# Discrimination of *Gentiana* and Its Related Species Using IR Spectroscopy Combined with Feature Selection and Stacked Generalization

**DOI:** 10.3390/molecules25061442

**Published:** 2020-03-23

**Authors:** Tao Shen, Hong Yu, Yuan-Zhong Wang

**Affiliations:** 1 Yunnan Herbal Laboratory, Institute of Herb Biotic Resources, School of Life and Sciences, Yunnan University, Kunming 650091, China; st_yxnu@126.com; 2 The International Joint Research Center for Sustainable Utilization of Cordyceps Bioresources in China (Yunnan) and Southeast Asia, Yunnan University, Kunming 650091, China; 3 College of Chemistry, Biological and Environment, Yuxi Normal University, Yu’xi 653100, China; 4 Medicinal Plants Research Institute, Yunnan Academy of Agricultural Sciences, Kunming 650200, China; boletus@126.com

**Keywords:** NIR, FT-MIR, species identification, *Gentiana*, chemometrics, feature selection, stacked generalization

## Abstract

*Gentiana*, which is one of the largest genera of Gentianoideae, most of which had potential pharmaceutical value, and applied to local traditional medical treatment. Because of the phytochemical diversity and difference of bioactive compounds among species, which makes it crucial to accurately identify authentic *Gentiana* species. In this paper, the feasibility of using the infrared spectroscopy technique combined with chemometrics analysis to identify *Gentiana* and its related species was studied. A total of 180 batches of raw spectral fingerprints were obtained from 18 species of *Gentiana* and *Tripterospermum* by near-infrared (NIR: 10,000–4000 cm^−1^) and Fourier transform mid-infrared (MIR: 4000–600 cm^−1^) spectrum. Firstly, principal component analysis (PCA) was utilized to explore the natural grouping of the 180 samples. Secondly, random forests (RF), support vector machine (SVM), and K-nearest neighbors (KNN) models were built while using full spectra (including 1487 NIR variables and 1214 FT-MIR variables, respectively). The MIR-SVM model had a higher classification accuracy rate than the other models that were based on the results of the calibration sets and prediction sets. The five feature selection strategies, VIP (variable importance in the projection), Boruta, GARF (genetic algorithm combined with random forest), GASVM (genetic algorithm combined with support vector machine), and Venn diagram calculation, were used to reduce the dimensions of the data variable in order to further reduce numbers of variables for modeling. Finally, 101 NIR and 73 FT-MIR bands were selected as the feature variables, respectively. Thirdly, stacking models were built based on the optimal spectral dataset. Most of the stacking models performed better than the full spectra-based models. RF and SVM (as base learners), combined with the SVM meta-classifier, was the optimal stacked generalization strategy. For the SG-Ven-MIR-SVM model, the accuracy (ACC) of the calibration set and validation set were both 100%. Sensitivity (SE), specificity (SP), efficiency (EFF), Matthews correlation coefficient (MCC), and Cohen’s kappa coefficient (K) were all 1, which showed that the model had the optimal authenticity identification performance. Those parameters indicated that stacked generalization combined with feature selection is probably an important technique for improving the classification model predictive accuracy and avoid overfitting. The study result can provide a valuable reference for the safety and effectiveness of the clinical application of medicinal *Gentiana*.

## 1. Introduction

Genus *Gentiana* is one of the largest groups in Gentianaceae, comprising 360 species that are widespread across Northwest of Africa, Europe, America, East of Australia, and Asia [1]. Many species of this genus have significant economic value and they are widely used by the food and pharmaceutical industries in the world [2,3,4]. In Europe, *G. lutea* (Yellow Gentian) are traditional materials for alcoholic bitter beverages and have a function of being appetite stimulating and improving digestion [4,5]. In Asia, *Gentiana* has a long history in use for medicine [2,3,6]. Places, including Iran, Mongolia, Japan and Korea have literature and details about nature and the of medicinal *Gentiana* plants found in these countries [3,7,8]. In China, species of *Gentiana* are diverse (about 248 species) and some of them have been an important part of traditional Chinese medicine (TCM) for a long time [1,9]. Approximattely 2000 years ago, Chinese Medicine monographs, “Shen Nong Ben Cao Jing”, had described and recorded function and medicinal value of Longdan (Gentianae Radix et Rhizoma: dried root and rhizome of *G rigescens*, *G. trifloral*, *G. manshurica* and *G. scabra*) and Qinjiao (Gentianae Macrophyllae Radix: dried root of *G. macrophylla*, *G. straminea*, *G. crassicaulis*, and *G. dahurica*) [10]. Presently, nine species of *Gentiana* have been recorded as the official drug of Pharmacopoeia of the People’s Republic of China (Ch.P. 2015 edition) [9]. But besides that, *G. cephalantha*, *G. davidii*, *G. loureirii*, *G. rubicunda G, lawrencei* var. *farreri*, and other species have been used as a popular herb in folk medicine and many other ethnomedicines for remedy digestive and respiratory illnesses [11,12,13]. *Gentiana* and its related species are extensively used for various health disorders due to the cheap price of traditional herbs [13]. These medicinal plants have always played an important role in the health care of local people, especially in the underdeveloped area of southwest China.

Chemical and pharmacological researches have indicated that the composition of bioactive compounds is diverse according to different *Gentiana* species [2,6]. Until now, more than 500 secondary metabolites have been isolated from approximately 60 species [2]. Those compounds, including iridoids, triterpenoids, flavonoids, alkaloids, and other types of secondary metabolites [2,14,15]. *Gentiana* species have different therapeutic properties and medicinal functions because of the complicated chemical profiles [2,13,14]. For example, *G. lute* and *G. rigescens* could be used as raw materials for the preparation of the therapeutic drug for Alzheimer’s disease because of neuritogenic compounds that were isolated from the two species [4,16,17]. Although *G. straminea* and *G. scabra* are rich in iridoids, chemical composition and traditional uses are different between the two species. *G. straminea* is used for treating rheumatic arthritis, while *G. scabra* is used for liver protection [6,14]. *G. rhodantha* and *G. rigescens* usually are often confused in traditional medicine markets in southwest China. In fact, the former is good at treating cough and other throat illnesses that are caused by fever, and, while the latter is used for chronic liver disease, inflammatory skin diseases, and clearing away heat [9,13]. Those cases showed that the identification of *Gentiana* species is crucial for keeping the clinical effect consistent and ensuring patients’ medication safety.

*Gentiana* species show extremely high morphological similarity and their Chinese names of species are often used in confusion in the market (see sample information). Furthermore, the powder of medicinal materials of *Gentiana* species is difficult for achieving the identification. Although pharmacognosy morphology identification or microscopic identification based on inner structural composition features and the inclusions of medicinal materials may be used for this purpose [9]; these works critically depend on personal experiences. In recent years, the researches regarding authenticity identification and discrimination of *Gentiana* and its relatives were focused on DNA barcoding, ISSR amplification, and other molecular identification technologies [18,19,20,21]. In addition, chromatographic and mass-spectrometric techniques were applied for species classification [10,22,23]. However, these methods need a complex process of extractions, tedious pretreatment, a great number of chemical reagents, waste time, and are expensive. A rapid, high-accurate, and green authenticity identification method needs to be established to ensure the effectiveness and safety of the clinical application of *Gentiana*. In the past few decades, ultraviolet-visible (UV-Vis), Raman, and infrared (IR) spectroscopic have gained the attention of various botany scientists and pharmacognosists [23,24,25,26]. Among them, near-infrared (NIR) and mid-infrared (MIR) spectroscopy are probably the most publicized technologies [27,28,29,30]. These two technologies can provide detailed structural information on sample properties and composition at the molecular level [31,32,33,34]. Like human fingerprints, the infrared spectrum of any substance has to be unique [31]. This is the reason for NIR and MIR spectral fingerprints can be applied to identify or classify different samples [31,32,33,34]. In the case of medicinal plants, chemical constituents and their ratios of biochemicals of different species can vary substantially [35,36]. The IR spectroscopy could be used for the identification of medicinal species because the corresponding spectral signals of these chemicals are highly specific [35,36]. Recently, successful species discrimination of *Dendrobium*, *Paris*, *Rhodiola*, *Ganoderma*, and the other genus based on IR spectroscopy has been reported [35,36,37,38].

In the process of spectral discrimination, it is necessary to establish a relationship between the chemical information and sample categories by chemometrics then to establish a classification model for the class identification of unknown samples [39]. Additionally, feature variable selection and model optimization strategy that are based on chemometrics are key steps during the model building [40]. From the literature, it can be found that a combination of variable selection methods and different algorithms could provide multifarious modeling strategies and most of them showed the superior ability for classification and identification [41,42]. With the development of modeling methods, Wolpert developed stacked generalization in the early 1990s [43]. This method combines multiple models together to produce a meta-model with equal or better classification performance than the constituent parts [43,44]. In theory, this modeling strategy belongs to the ensemble model, and its classification result might be better than any of the constituent sub-models [44,45]. For example, Shan’s research showed that the performance of an extreme learning machine model that was based on stacked generalization was more robust than the traditional model [46]. Sfakianakis’s research reported a similar finding [47]. Although stacked generalization might be an approach for improving model prediction accuracy and robustness, there was limited reporting of this method applied to medicinal plant research.

The aim of this research was (1) to investigate the application of NIR (near-infrared) and FT-MIR (Fourier transform mid-infrared) spectroscopies to the classification of medicinal *Gentiana* and its wild relatives; (2) to select the optimal bands that identify the differences among different species; and, (3) to examine the feasibility of using stacked generalization combined with infrared spectral data to identify *Gentiana* species. The results of the study may provide some basis for the safety and effectiveness utilization of medicinal *Gentiana* resources in China.

## 2. Results and Discussion

### 2.1. Spectral Fingerprint of NIR and FT-MIR

**Figure 1:**
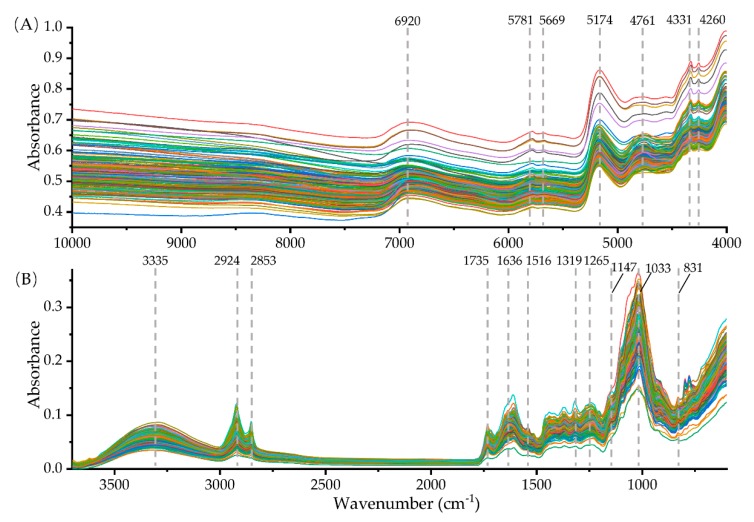
Raw near-infrared (NIR) (**A**) and Fourier transform mid-infrared (FT-MIR) (**B**) spectra of 180 samples of *G. rigescens* and its related species.

Figure 1 shows the raw NIR spectra and FT-MIR that were obtained from 180 samples of *G. rigescens* and their relatives. It can be seen from the raw NIR spectra that there are seven distinct absorption bands, which are located at 6920, 5781, 5669, 5174, 4761, 4331, and 4260 cm^−1^, respectively (Figure 1A). In the whole FT-MIR spectral range (Figure 1B), 3335, 2924, 2853, 1735, 1636, 1516, 1319, 1265, 1147, 1033, and 831 cm^−1^ appeared in all species.

**Figure 2:**
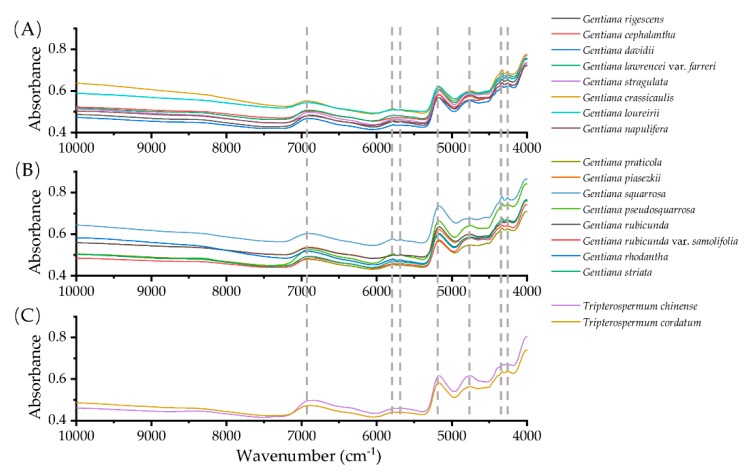
Averaged NIR spectra of 18 species of *Gentiana* (**A**), (**B**) and *Tripterospermum* species (**C**).

In the range of 7171–6514 cm^−1^, *G. rhodantha* is clearly different from the other two traditional medicinal *Gentiana* species. It is interesting that the NIR spectra of *T. chinense* and *T. cordatum* are similar to *G. rigescens* and *G. crassicaulis*. The spectral intensity of *G. davidii* at 4225 cm^−1^ was different from *G. rigescens* and *G. cephalantha* (Figure 2). In fact, the three species have similar plant morphology and *G. cephalantha* and *G. davidii* are primary alternative species of *G. rigescens* in remote rural of the southwest of China.

**Figure 3:**
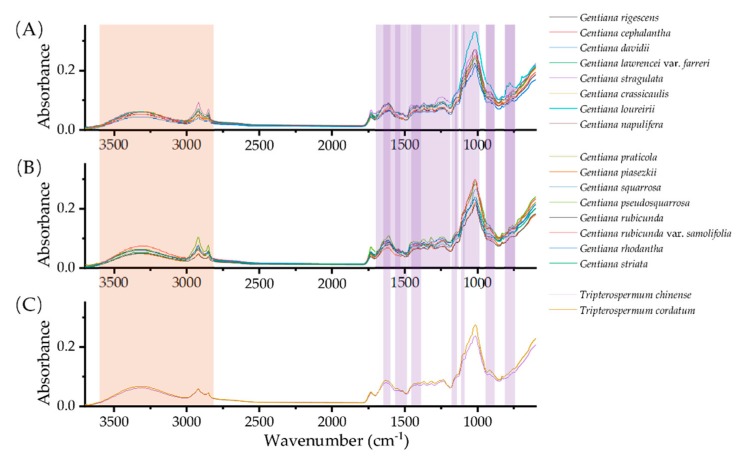
Averaged FT-MIR spectra of 18 *Gentiana* (**A**), (**B**), and *Tripterospermum* species (**C**).

The FT-MIR spectra of 18 species showed very similar band distributions in the whole spectral range of 3587–2827 cm^−1^, but there were differences in the relative intensities of the spectral absorption bands of samples in the range of 1780–600 cm^−1^ (Figure 3). For example, the huge spectral differences between the bands 1709–1531, 1478–1207, 1168–1130, 1114–1015, 948–883, and 822–740 cm^−1^ were observed among *G. rigescens, G. crassicaulis, G. rhodantha, G. davidii, G. pseudosquarrosa*, and *G. stragulata*. Obviously, the fingerprints of *Tripterospermum* species and *Gentiana* species were significantly different in the 1650–1600, 1579–1494, 1458–1393, 1164–1126, 1112–1090, 950–883, and 822–740 cm^−1^, respectively (Figure 3).

### 2.2. Exploratory Statistical Analysis

**Figure 4:**
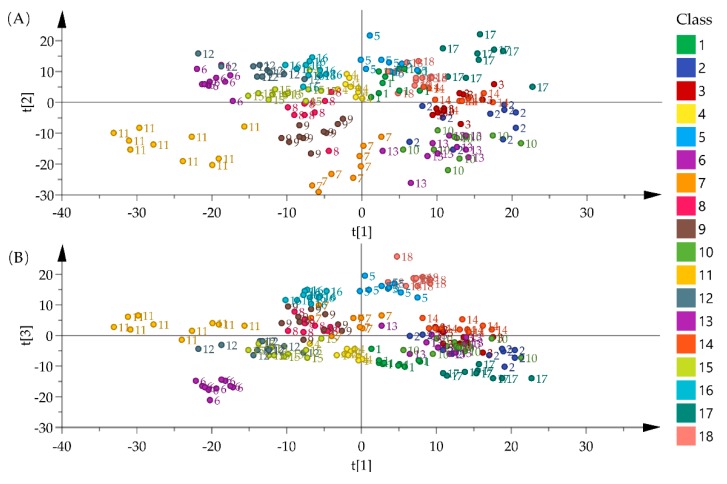
Score plots of PCA for 180 samples using NIR spectra after pretreatment (**A**) score plot of PC1 vs. PC2, (**B**) score plot of PC1 vs. PC3. The meaning of the codes (1–18) could be found in the sample information.

Before statistical analysis, all of the spectra datasets were pretreated by the second derivative and standard normal variate for improving visualization results. The score plots that were obtained after principal component analysis (PCA) on the NIR data set are shown in Figure 4. A faint clustering of samples was observed in the figure. The score-plot for PC1 vs. PC2 displays *G. squarrosa* (11) could be clearly separated from other species (Figure 4A). In Score-plot for PC1 vs. PC3, *G stragulata* (5) and *T. cordatum* (18) were clustered and samples from the *G. crassicaulis* (6) were more easily differentiated from other samples (Figure 4B).

**Figure 5:**
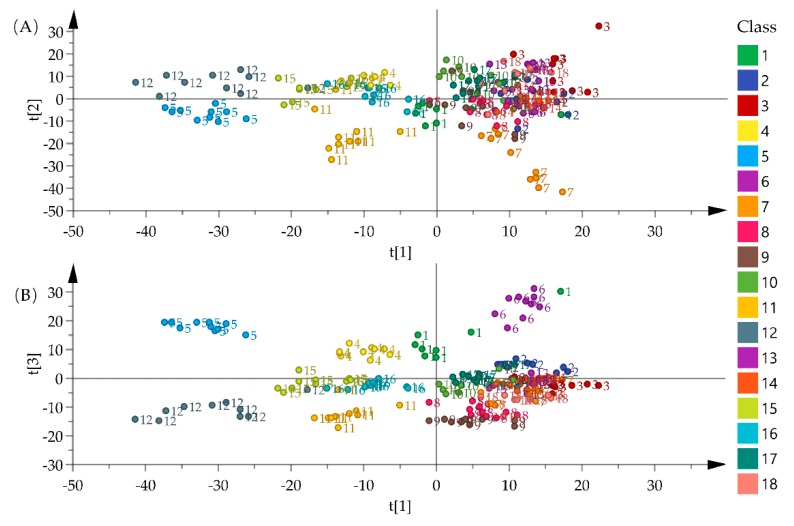
Score plots of PCA for 180 samples using FT-MIR spectra after pretreatment (**A**) score plot of PC1 vs. PC2, (**B**) score plot of PC1 vs. PC3. The meaning of the codes (1–18) could be found in the sample information.

Figure 5 shows score plots that were obtained by an application of PCA on the FT-MIR spectra data. According to the scatter plot of PC1 vs. PC2, *G. stragulata* (5) and *G. pseudosquarrosa* (12) were clustered. The samples of *G. lawrencei* var. *farreri* (4), *G. rhodantha* (15), and *G. striata* (16) were both located in the middle of the PC1 and PC2 axes. Most of the samples of *G. squarrosa* (11) were significantly different from other species and they were located on the negative side of PC1 and PC2. With the exception of the above species, all of the other species are grouped into one group (Figure 5A). From the scatter plot of PC1 vs. PC3. *G. stragulata* (5) and *G. crassicaulis* (6) were each separately clustered. Additionally, samples from the *G. squarrosa* (11) could be distinguished from those of the *G. pseudosquarrosa* (12) (Figure 5B).

The grouping results indicated a potential application value of NIR and FT-MIR fingerprint for the discrimination of medicinal *Gentiana* and its related species. Nonetheless, most of *Gentiana* species would be difficult to differentiate from one another, due to the overlap of their sample score. Hence, the application of supervised pattern recognition methods, such as random forest (RF), support vector machines (SVM), and k-nearest neighbors (KNN), for the development of classification models were required for enabling one to distinguish the samples.

### 2.3. Single Block Models for Sample Classification

#### 2.3.1. Classification Based on Full Spectra

In the section, all of the classification models were established by full spectra data (the total number of points in NIR and FT-MIR is 1487 and 1214, respectively) and 180 samples were separated into a calibration set (108 samples) and a validation set (72 samples) by the Kennard–Stone algorithm [48]. Six performance parameters, including sensitivity (SE), specificity (SP), efficiency (EFF), accuracy (ACC), Matthews correlation coefficient (MCC), and Cohen’s kappa coefficient (K), were applied to evaluate the identification ability of classification models [49,50]. Those parameters values range from 0 to 1, indicating a perfect classification when the values are 1 [49].

For RF models, model performance depends on the proper selection of the hyperparameters, which are *n_tree_* and *m_try_* [49]. Figures S1 and S2 show the suitable hyperparameters and variation of model mean misclassification error (MMCE) with different hyperparameters. The lower MMCE the hyperparameter was better [50]. Table 1 and Table 2 present classification accuracies rates in the calibration and validation data sets of 18 species that were obtained by NIR-RF and FT-MIR-RF models. For the two models, all of the samples in the calibration set were correctly classified. Additionally, the accuracy rates of validation sets were not less than 97.22%. Although the FT-MIR-RF model had higher total validation accuracy (94.44%), its SE, MCC, and EFF values of the validation set were lower than the NIR-RF model. Hence, the phenomenon of imbalance category recognition in the FT-MIR-RF model was worse (Table 1 and Table 2).

For the SVM models, the optimum kernel function (sigmoid, polynomial, and radial kernel) and the cost function were important for modeling [35,51]. Hyperparameter optimization results showed the linear kernel had lower MMCE value than sigmoid, polynomial, and radial kernel. Hence, the linear kernel was suitable for modeling (Figures S3 and S4). Subsequently, the cost function was optimized. And the most suitable values 5 and 0.05 were selected as the best cost function for the SVM models of NIR and FT-MIR, respectively (Figures S3 and S4). Table 3 and Table 4 present the major parameters of the calibration and validation sets for NIR-SVM and FT-MIR-SVM models. It could be seen that the samples of 18 species were better discriminated by using the FT-MIR data set. FT-MIR-SVM model achieved 100% total accuracy for the calibration set and validation sets.

Determining parameter *k* is critical for KNN [52]. Hence, this hyperparameter was optimized before modeling and the optimum *k* value for NIR and FT-MIR data set were both one (Figures S5 and S6). Table 5 and Table 6 present the classification accuracies rates in the calibration and validation data sets of 18 species obtained by NIR-KNN and FT-MIR-KNN models. Although the calibration set accuracy of the NIR-KNN model reached 100%, the total validation set accuracy was 88.89%. The performance of the FT-MIR-KNN model was better than the NIR-KNN model. Its total accuracy of the validation set was 94.44%. By comparison of validation set parameters (SE, SP, MCC, and EFF), it was clear that the performance of the KNN models was worse than RF and SVM models. Additionally, the highest classification accuracy was obtained with the use of the SVM combined with the FT-MIR data set.

#### 2.3.2. Feature Selection

**Figure 6:**
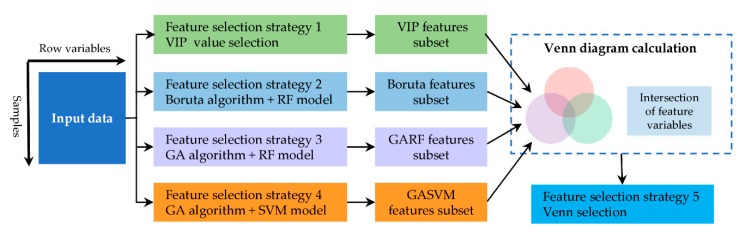
Feature selection strategies in the study.

**Figure 7:**
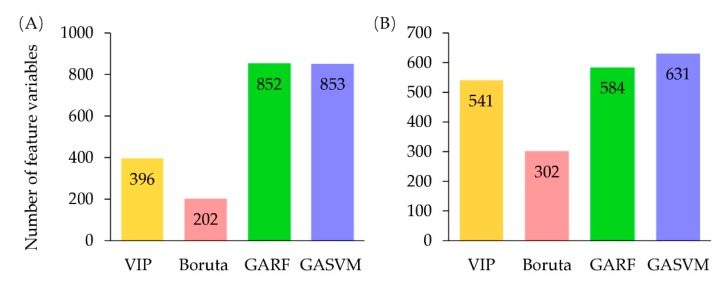
Size of feature variables of the four algorithms (**A**) feature selection of NIR spectroscopy, (**B**) feature selection of FT-MIR spectroscopy.

**Figure 8:**
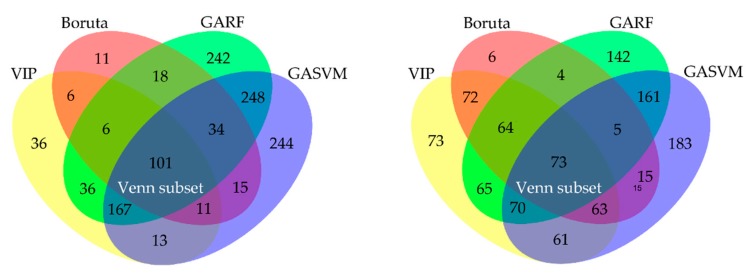
Venn diagram representing the overlap of the selected feature variables by variable importance in projection (VIP), Boruta, genetic algorithm combined with random forest (GARF), and genetic algorithm combined with support vector machine (GASVM) algorithms (**A**) Venn diagram calculate based on feature selection results of NIR variables, (**B**) Venn diagram calculate based on feature selection results of FT-MIR variables.

It is necessary to screen out the most relevant chemical information for classification with specific variables selection methods in order to improve the classifier performance. In the study, five methods were used to feature selection (Figure 6). Firstly, VIP (variable importance in projection), Boruta, GARF (genetic algorithm combined with random forest), and GASVM (genetic algorithm combined with support vector machine) were applied to select feature variables [49,50]. Secondly, the intersection of feature variables that were selected by these four algorithms was calculated and the result was the fifth approach of feature selection (Venn selection). Figure 7 displays the number of feature variables of each selection method. Further analysis by Venn diagram found that 101 NIR variables and 73 FT-MIR variables were common characteristic variables of the four selection methods, respectively (Figure 8). Those variables were 6.79% and 6.01% of the full NIR spectrum and full FT-MIR spectrum, respectively. In the final, 10 feature subsets were established. They were the VIP-NIR, Bor-NIR, GARF-NIR, GASVM-NIR, Ven-NIR, VIP-MIR, Bor-MIR, GARF-MIR, GASVM, and Ven-MIR subset.

Models of the RF, SVM, and KNN were established based on the optimal data sets of NIR and FT-MIR to verify the validity of the feature selection for improving modeling performance. Table 7, Table 8, and Tables S7–S36 show the recognition effect of each model for the calibration set and the prediction set.

Obviously, the use of the VIP-NIR and Ven-NIR data sets could produce better classification performance for all of the classifiers in comparison with using full spectrum information (Table 7). For the SVM classifier, its accuracy of the validation set increases to 98.61% with the use of feature variables that were selected by Boruta. However, there is a slight decrease in RF classifier performance with the use of the same feature variables. In addition, there is no improvement for classifiers’ performance when using GASVM. Overall, in the case of NIR models, the performance of the classifiers for different *Gentiana* species showed the best results when using SVM that was combined with Boruta or Venn feature selection.

For MIR spectral data (Table 8), the performance of the RF classifiers for the classification of samples shows acceptable results with maximum validation accuracies of 97.22% and 98.61% that were obtained using VIP-MIR and Ven-MIR data sets, respectively. Similar results have been achieved in the study of the KNN models. Although the validation accuracy of the Ven-MIR-SVM model was 98.61% and lower than the full spectra SVM model, but feature selection greatly reduced the SVM models’ variables and kept a good classification performance of models.

Comprehensive comparison modeling results, the optimal spectrum that was selected by Venn was effectively increasing the performance of the NIR and FT-MIR classification models. Additionally, Ven-NIR and Ven-MIR were the optimal data sets for further modeling. The 101 NIR variables and 73 FT-MIR spectral variables were the most important variables for the species discrimination (Figure 8, Table 7 and Table 8).

### 2.4. Model Stacking for Sample Classification

**Figure 9:**
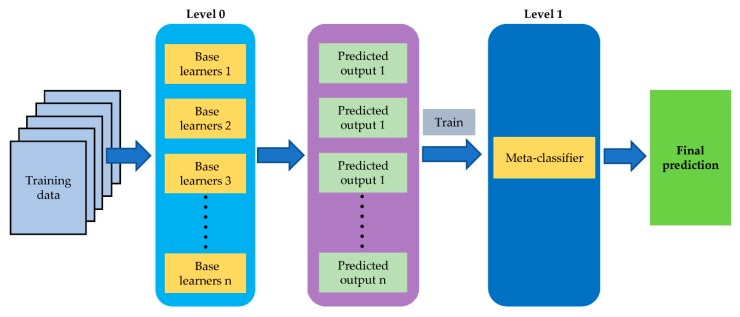
Stacked generalization in the study.

Although most of the models that were based on data sets of Ven-NIR and Ven-MIR had high accuracy, it is possible that stacked generalization could establish a model that had a better performance when compared to the individual classifiers. Through comparisons of tge classification results of Section 2.3.1 and Section 2.3.2, it could be found that RF and SVM appear to be the most effective of individual classifiers, realizing the highest classification rates in many cases when compared to KNN. Confusion matrices that correspond to Ven-NIR-RF, Ven-NIR-SVM, Ven-MIR-RF, and Ven-MIR-SVM shows that the predicted outputs of the two algorithms might be complementary (Supplementary Materials Tables S1–S36). All of the results suggest that the two learners would be the best combination of base learners. Accordingly, RF and SNV models as level-0 base learners were employed in our stacked generalization. Additionally, RF, SNV, and KNN algorithms were used at level-1 learners, respectively. In the final, a total of six scenarios were performed with stacking experiments (Table 9). Additonally, Figure 9 shows the schemes for stacked generalization.

For the Ven-NIR data set (101 variables), the best performing classifier was scenario A. The next best-performing classifiers were scenario B and C, respectively. For the Ven-MIR data set (73 variables), a model of scenario E showed the highest classification rates and second were scenario D and F (94.00% and 90.00% classification rate respectively). Comparing the performance of different stacking models (Table 9 and Supplementary Materials Tables S37–S42), SVM comes out to be the best algorithm at level-1. Additionally, the stacking model, based on the Ven-MIR data set, had the highest accuracy of calibrations and validations sets. The comprehensive analysis revealed that the SVM stacking model combined with the Ven-MIR data set had the best performance (SG-Ven-MIR-SVM).

### 2.5. Are Model Stacking Better than Data Fusion for Gentiana Species Discrimination?

Presently, the application of stacked generalization for establishing classification models of different medicinal plants or herbs is rather scarce. On the contrary, another modeling approach, data fusion strategy, has been widely used for classification and geographical origin traceability of herbs and foods [48,49,53,54]. Some researches stated that spectra data fusion, such as low-level and mid-level fusion strategies, could improve the discrimination capacity of the classification models and those strategies were usually more efficient than single spectroscopic techniques for modeling [48,49]. We select the Ven-MIR-SG-SVM model in the last section of the research to compare with six data fusion models on prediction accuracy and validate the advantage of stacked generalization in the classification of *Gentiana* species.

**Figure 10:**
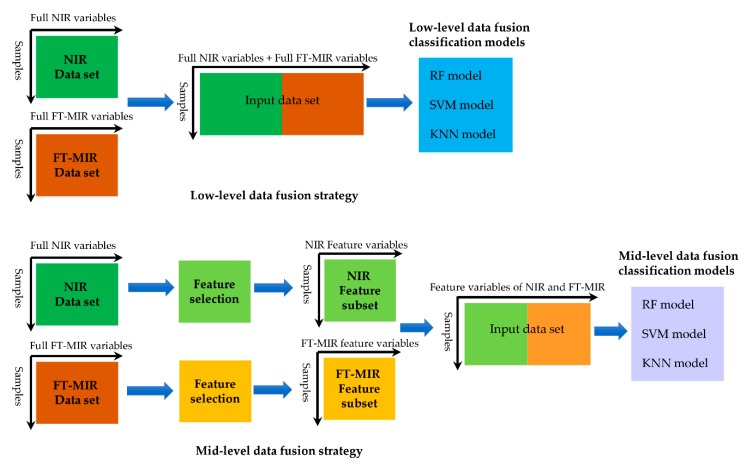
The low-level and mid-level data fusion strategies in the study.

In this study, the FT-MIR and NIR spectral signals were straightforwardly concatenated and they constitute a low-level fusion data set (a total of 2701 variables: the total number of the points in the both MIR and NIR spectra). The mid-level data fusion data set (174 variables) was made up of feature important variables from Ven-NIR (101 variables) and Ven-MIR (73 variables) subsets (Figure 10). Finally, the low- and mid-level data fusion matrices were used to establish the RF, SVM, and KNN models, respectively (Table 10 and Tables S43–S48). For low-level data fusion, the order of successful classification rates of three algorithms was as follows: SVM > RF and KNN. The SVM model resulted in a total accuracy of 100%. Additionlly, the validation set accuracy of RF and KNN were both 97.22%. In the case of mid-level fusion, the SVM model still achieved a total accuracy rate of 100%. In addition, the parameters of RF and KNN models that were based on feature fusion data set of FT-MIR and NIR spectra were higher than that of low-level data fusion.

The low and mid-level data fusion approach improved the discrimination capacity of the developed models to classify *Gentiana* samples, as shown in Table 10. Among the six classification models that were based on data fusion strategy, Low-SVM, Mid-RF, Mid-SVM, and Mid-KNN were the best performing model according to accuracy, kappa coefficient, and other indicators. When compared with these models, the performance of SG-Ven-MIR-SVM was as good as them (Table 9 and Table 10). The experimental results that were obtained from the two different modeling strategies showed that both model stacking and data fusion could result in a classification model with improved accuracy and enhanced robustness. Additionally, the strategy of stacked generalization could obtain efficient classification models that are as good as data fusion by fewer variables.

As we know, the data fusion (low-level and mid-level) approaches present a fusion of all variables or most important variables (feature variables) to create a model in order to exploit the synergy of the multispectral information to obtain an optimized model [53,54,55,56]. However, the calculation time might be higher when increasing variables. In contrast, stacked generalization reduces the calculation time and keeps fewer variables by combining several different classification algorithms into one meta-model [57,58,59]. In the case of discrimination of *Gentiana* and its relatives, only 73 variables used in the SG-Ven-MIR-SVM model, while low-level and mid-level data fusion models utilized 2701 and 174 variables for modeling, respectively. The variables number and modeling results indicated that the stacked generalization strategy is probably an important technique for improving species classification model predictive accuracy and avoiding overfitting.

## 3. Materials and Methods

### 3.1. Plant Material Collection

**Figure 11:**
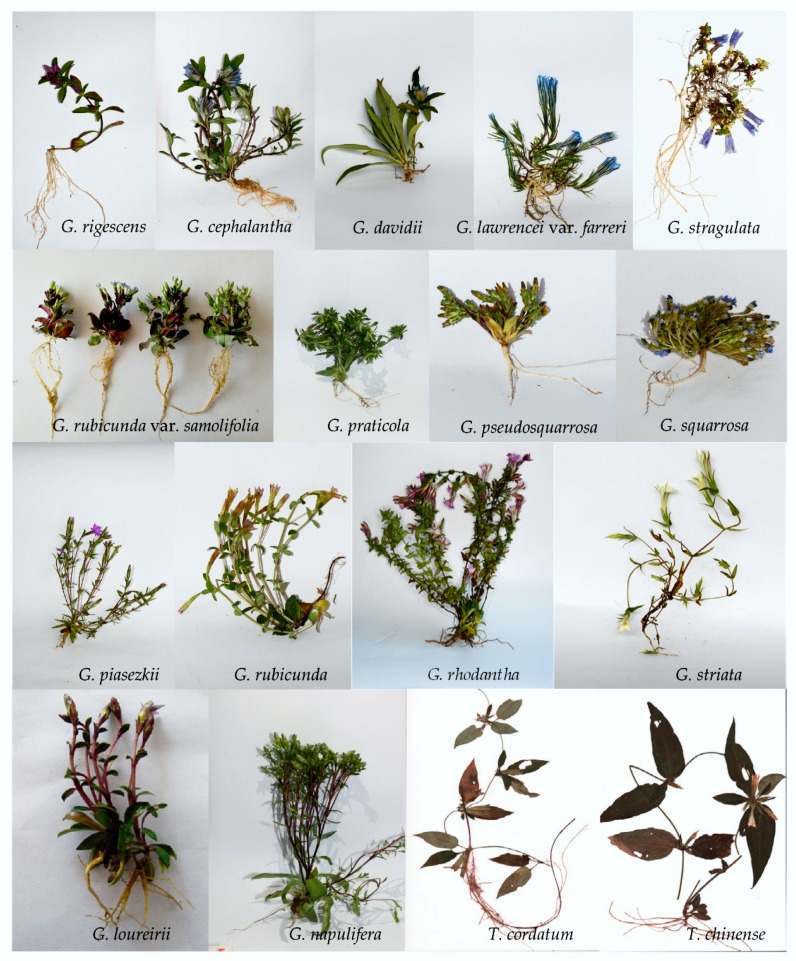
Medicinal *Gentiana* and its relatives in the study.

The 18 species used in the study belong to two genera (*Gentiana* and *Tripterospermum*) of Gentianaceae (Figure 11). All of the species were collected and identified during the flowering and fruiting time of 2018 and 2019. The voucher specimens of those plants were deposited in the College of Chemistry, Biological and Environment, Yuxi Normal University, Yu’xi, China. Their collection location is shown in Table 11 and medicinal use in southwest China was summarized in Table 12.

In the laboratory, the fresh materials were authenticated. Subsequently, the samples were wash cleaning and dried at 50 °C as soon as possible. The dried whole plant was broken into powder with high-speed disintegrator. Finally, 180 powder samples were collected (10 powder samples per species). All sample powders were screened through a 100-mesh stainless sieve to obtain same-sized particles. The powders after sieving were stored in dry zip-lock bags for a further spectra scan of NIR and FT-MIR.

### 3.2. Near Infrared (FT-NIR)

The samples were scanned in the Antaris II spectrometer (Thermo Fisher Scientific, Madison, WI, USA). Each powdered sample was scanned from 10,000 to 4000 cm^−1^ with a resolution of 4 cm^−1^ until 16 scans were averaged.

### 3.3. Fourier Transform Mid Infrared (FT-MIR)

**Figure 12:**
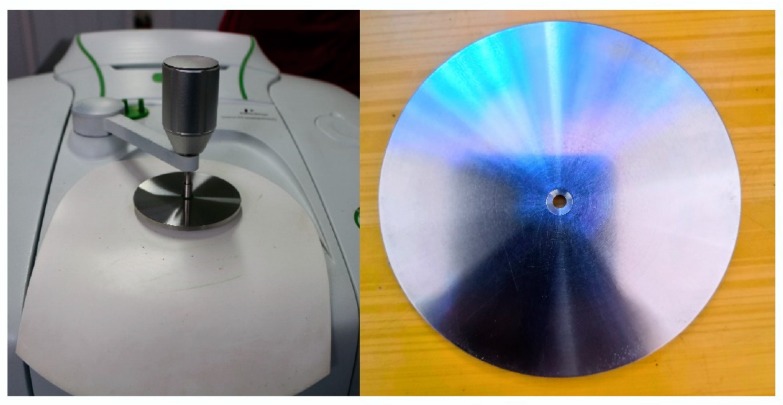
ZnSe ATR accessory (left) and the metal O-ring (right) in the study.

The FT-MIR spectrum was recorded using a FT-IR spectrometer (Perkin Elmer, Norwalk, CT, USA) that was equipped with a deuterated triglycine sulfate (DTGS) detector and a ZnSe ATR (attenuated total reflection) accessory (PIKE technologies, Inc. Madison, WI, USA). The spectral fingerprint of every sample was recorded bands from 4000–600 cm^−1^ while using a resolution of 4 cm^−1^ and an accumulation of 16 scans. The ATR accessory is equipped with a unique metal O-ring for sample holding in order to control the path length and thickness of the sample (Figure 12). In the beginning, the metal O-ring was placed on the reflection diamond of accessory, and then the sample powder was put on the central of O-ring metal. At last, a pressure tower on the top of the metal O-ring was used to press the powder tightly until a constant pressure (131 ± 1 bar on the scale of the micrometric pressure device) [60]. Before each measurement, a laboratory air spectrum was recorded and checked for remaining water and sample residues, as well as background deduction.

Spectrum signals from 2500 to 1800 cm^−1^ were not considered for further analysis due to strong crystal absorbance [61]. Furthermore, spectral regions that 4000–3700 cm^−1^ (baseline area and did not provide relevant information) and 682–653 cm^−1^ (disturbing absorption band of CO_2_) were excluded prior to chemometric analysis [62].

### 3.4. Statistical Analysis

The principal component analysis (PCA), unsupervised technique, has been widely applied in data dimension reduction and exploratory data analysis [37,63]. From PCA-loading analysis, we can also extract the characteristic variables, which lead to differences between the samples. Additionally, in general, the more important the band corresponding to the spectral variable, the larger PCA-loading value. In this study, PCA was applied to test whether the NIR and FT-MIR spectra fingerprint can result in a clustering of 180 samples and analyze the similarity and dissimilarity in spectra data between species, which might be useful for further understanding phytochemical diversity among different species. Furthermore, the results of PCA would provide reference information for the creation of classification models based while using the supervised technique.

Random forests (RF) or decision tree forests is an ensemble learning technique [64]. This algorithm is based on a combination of a large set of classification and regression trees [64]. After the ensemble of trees (the forest), each tree gives a classification. Finally, the model uses a vote to combine the trees’ predictions [64]. RF can handle extremely large datasets and deal with the “curse of dimensionality” well. Therefore, RF is robust to over-fitting, noise, and outliers, and always performs well in problems with a low feature ratio [65]. All of those indicate that RF is quite competitive relative to other ensemble learning techniques.

The support vector machine (SVM) algorithm is a non-parametric supervised classification [66]. Many previous studies have reported the theory and detailed mathematical explanation of this algorithm [67]. As one of the most robust and accurate data mining algorithms, SVM has been implemented in many programming languages, including R, MATLAB, and so on, which has led SVM to be adopted by a much wider audience. In recent years, SVM has successfully been applied to a number of applications, such as classification of species or geographical origin traceability of food [53,68,69]. It is important to note that SVM can achieve high classification accuracy whlie using a small number of training samples [56,67]. Additionally, it is also a suitable classifier for high-dimensional data [53,69].

The k-nearest neighbors (KNN) algorithm is a distance-based non-parametric discriminant technique [70]. As its name, this algorithm uses information regarding an example’s k-nearest neighbors to classify unlabeled examples and assign one of them to the most common class among the k-nearest neighbors [70]. KNN has been widely used in statistical applications and it has been one of the most successful supervised classification algorithms, especially for the task of multi-class classification [31,71].

Hyperparameters of RF (*n_tree_* and *m_try_*), SVM (kernel function and cost function), and KNN (*k*) were optimized by using Bayesian optimization of mlr package combined with the MMCE model [50]. The lower MMCE, the hyperparameter was better [50].

Feature selection (“optimal wavenumbers” for classification modeling) is a critical step in the modeling process [72]. There might be some irrelevant or noisy features in data sets because of the infrared techniques provide multivariate and non-specific signals [72,73]. Feature selection of NIR and FT-MIR subsets was based on five methods. The first four were VIP (features were selected by the PLS-DA combined with VIP value) [49], Boruta (features were selected by the Boruta algorithm) [49], GARF (features were selected by the genetic algorithm combined with RF model), and GASVM (features were selected by the genetic algorithm combined with SVM model) [50]. The last was Venn, which feature variables were the intersection of the results of the first four feature selection.

### 3.5. Model Stacking and Data Fusion

Stacked generalization (stacking) is one of the ensemble learning [43]. The essence of the method is combined predictions from a number of base learners (level 0 models) to generate a more powerful meta-model (level 1 models), with the aim of reducing the generalization error [43,44,45]. Hence, stacked generalization is an ensemble learning method with two or more levels models. The greatest advantage of stacked generalization is the free choice of base learners. Additionally, in general, the classification results of base learners might be complementarities and this combination might be helpful in improving the performance of the final meta-model [44]. Hence, investigating the best methods for constructing the ensemble classifiers was one focus of stacking.

In our study, the first level (level-0) of stacking model is composed of several weak classifiers (base learners 1, base learners 2, base learners 3, base learners n) [45]. Subsequently, the predicted probabilities of basic learners are used to train the second level model (final model) [45]. Figure 9 shows the schemes for stacked generalization.

Unlike stacked generalization, the data fusion strategy focus is on improving the model through best combine the subset. Most of the reported data fusion strategies include low-level data fusion and mid-level data fusion (feature-level data fusion) [48,53].

Low-level data fusion, as its name suggests, subsets are straightforwardly concatenated and reconstitute an independent data matrix. Subsequently, the new dataset is used to establish the classification models [53]. In the case of mid-level data fusion, classification models were established by a new data set, which were formed by concatenating the feature important variables from a subset by different feature selection algorithms [53]. In the research, the low- and mid-level data fusion strategies were considered. Additionally, Figure 10 shows the schemes for data fusion strategies.

### 3.6. Model Evaluation

The values of TP (Correctly identified samples of positive class), TN (correctly identified samples of negative class), FN (incorrectly identified samples of positive class), and FP (incorrectly identified samples of negative class) were calculated according to the confusion matrices of the classification models [49]. Subsequently, SE, SP, EFF, ACC, MCC, and K were calculated using Equations (1) to (6).

(1) $$\mathrm{ACC}=\frac{\left(\mathrm{TN}+\mathrm{TP}\right)}{\left(\mathrm{TP}+\mathrm{TN}+\mathrm{FP}+\mathrm{FN}\right)}$$

(2) $$\mathrm{SE}=\frac{\mathrm{TP}}{\left(\mathrm{TP}+\mathrm{FN}\right)}$$

(3) $$\mathrm{SP}=\frac{\mathrm{TN}}{\left(\mathrm{TN}+\mathrm{FP}\right)}$$

(4) $$\mathrm{EFF}=\sqrt{\mathrm{SE}\times \mathrm{SP}}$$

(5) $$\mathrm{MCC}=\frac{\left(\mathrm{TP}\times \mathrm{TN}-\mathrm{FP}\times \mathrm{FN}\right)}{\sqrt{\left(\mathrm{TP}+\mathrm{FP}\right)\left(\mathrm{TP}+\mathrm{FN}\right)\left(\mathrm{TN}+\mathrm{FP}\right)\left(\mathrm{TN}+\mathrm{FN}\right)}}$$

(6) $$\mathrm{kappa}=\frac{\left(\mathrm{Po}-\mathrm{Pe}\right)}{\left(1-\mathrm{Pe}\right)}$$

Po: observed agreement value, Pe: expected agreement value.

### 3.7. Software

ATR correction of the FT-MIR spectra was completed by OMNIC 9.7.7 software (Thermo Fisher Scientific, Madison, WI, USA). The other spectral data preprocessing (SNV and 2nd derivative), PCA, and VIP analysis were performed by SIMCA-P^+^ 14.0 Software (Umetrics AB, Umea, Sweden). In the study, a strategy of two levels stacked generalization was used and the models were developed with R [50]. Kennard–Stone algorithm was used to set the calibration sets and validation sets of all models (MATLAB, Version R 2017a, Mathworks, Natick, MA, USA). The RF, SVM, KNN technique, and feature selection of classification models were all implemented in R software (version 3.6.1, https://www.r-project.org/) base on randomForest, e1071, Boruta, mlr, and class package. The Venn diagrams were completed by the tools on BMKCloud (www.biocloud.net).

## 4. Conclusions

The results of this study indicated that NIR and FT-MIR spectroscopic techniques combined with chemometrics could successfully discriminate Chinese medicinal *Gentiana* and their related species. Exploratory data analysis showed the NIR and FT-MIR spectroscopy indirect reflection interspecific phytochemistry diversity of medicinal *Gentiana* among the genera level and species level. Hence, there was a potential application value of NIR and FT-MIR fingerprint for the identification of medicinal *Gentiana* and its related species. Subsequently, supervised methods of pattern recognition were used for further analysis of spectra data. Firstly, six classification models based on RF, SVM and KNN algorithms were built on the full spectra data set that was obtained by the NIR and FT-MIR spectroscopy technique, respectively. The FT-MIR-SVM model performed more effectively than other classification models. Five approaches were applied to select optimal wavenumbers in order to improve the performance of the models and filter irrelevant or noisy features in data sets. In the end, the stacking models were built by stacked generalization combined with NIR and FT-MIR feature data sets. The modeling results suggest that RF and SVM were the best combinations of base learners (level-0). When compared the performance of six stacking models, SVM comes out to be the best algorithm at level 1 and the stacking model using the Ven-MIR data set had the highest accuracy of calibrations and validation sets. In conclusion, stacked generalization combined with feature selection is probably an important technique for improving the classification model predictive accuracy and to avoid overfitting.

## Figures and Tables

**Table 1:** The major parameters of random forests (RF) model based on NIR full spectra data.

Class	Calibration Set					Validation Set				
	ACC (%)	SE	SP	MCC	EFF	ACC (%)	SE	SP	MCC	EFF
1	100.00	1.00	1.00	1.00	1.00	100.00	1.00	1.00	1.00	1.00
2	100.00	1.00	1.00	1.00	1.00	100.00	1.00	1.00	1.00	1.00
3	100.00	1.00	1.00	1.00	1.00	100.00	1.00	1.00	1.00	1.00
4	100.00	1.00	1.00	1.00	1.00	100.00	1.00	1.00	1.00	1.00
5	100.00	1.00	1.00	1.00	1.00	97.22	0.75	0.99	0.74	0.86
6	100.00	1.00	1.00	1.00	1.00	100.00	1.00	1.00	1.00	1.00
7	100.00	1.00	1.00	1.00	1.00	100.00	1.00	1.00	1.00	1.00
8	100.00	1.00	1.00	1.00	1.00	98.61	0.75	1.00	0.86	0.87
9	100.00	1.00	1.00	1.00	1.00	98.61	1.00	0.99	0.89	0.99
10	100.00	1.00	1.00	1.00	1.00	100.00	1.00	1.00	1.00	1.00
11	100.00	1.00	1.00	1.00	1.00	100.00	1.00	1.00	1.00	1.00
12	100.00	1.00	1.00	1.00	1.00	97.22	0.75	0.99	0.74	0.86
13	100.00	1.00	1.00	1.00	1.00	100.00	1.00	1.00	1.00	1.00
14	100.00	1.00	1.00	1.00	1.00	100.00	1.00	1.00	1.00	1.00
15	100.00	1.00	1.00	1.00	1.00	100.00	1.00	1.00	1.00	1.00
16	100.00	1.00	1.00	1.00	1.00	97.22	0.75	0.99	0.74	0.86
17	100.00	1.00	1.00	1.00	1.00	100.00	1.00	1.00	1.00	1.00
18	100.00	1.00	1.00	1.00	1.00	97.22	0.75	0.99	0.74	0.86

**Table 2:** The major parameters of RF model based on FT-MIR full spectra data.

Class	Calibration Set					Validation Set				
	ACC (%)	SE	SP	MCC	EFF	ACC (%)	SE	SP	MCC	EFF
1	100.00	1.00	1.00	1.00	1.00	100.00	1.00	1.00	1.00	1.00
2	100.00	1.00	1.00	1.00	1.00	100.00	1.00	1.00	1.00	1.00
3	100.00	1.00	1.00	1.00	1.00	100.00	1.00	1.00	1.00	1.00
4	100.00	1.00	1.00	1.00	1.00	100.00	1.00	1.00	1.00	1.00
5	100.00	1.00	1.00	1.00	1.00	100.00	1.00	1.00	1.00	1.00
6	100.00	1.00	1.00	1.00	1.00	100.00	1.00	1.00	1.00	1.00
7	100.00	1.00	1.00	1.00	1.00	100.00	1.00	1.00	1.00	1.00
8	100.00	1.00	1.00	1.00	1.00	98.61	0.75	1.00	0.86	0.87
9	100.00	1.00	1.00	1.00	1.00	98.61	1.00	0.99	0.89	0.99
10	100.00	1.00	1.00	1.00	1.00	98.61	1.00	0.99	0.89	0.99
11	100.00	1.00	1.00	1.00	1.00	98.61	1.00	0.99	0.89	0.99
12	100.00	1.00	1.00	1.00	1.00	98.61	0.75	1.00	0.86	0.87
13	100.00	1.00	1.00	1.00	1.00	100.00	1.00	1.00	1.00	1.00
14	100.00	1.00	1.00	1.00	1.00	100.00	1.00	1.00	1.00	1.00
15	100.00	1.00	1.00	1.00	1.00	98.61	1.00	0.99	0.89	0.99
16	100.00	1.00	1.00	1.00	1.00	97.22	0.50	1.00	0.70	0.71
17	100.00	1.00	1.00	1.00	1.00	100.00	1.00	1.00	1.00	1.00
18	100.00	1.00	1.00	1.00	1.00	100.00	1.00	1.00	1.00	1.00

**Table 3:** The major parameters of SVM model based on NIR full spectra data.

Class	Calibration Set					Validation Set				
	ACC (%)	SE	SP	MCC	EFF	ACC (%)	SE	SP	MCC	EFF
1	100.00	1.00	1.00	1.00	1.00	98.61	0.75	1.00	0.86	0.87
2	100.00	1.00	1.00	1.00	1.00	100.00	1.00	1.00	1.00	1.00
3	100.00	1.00	1.00	1.00	1.00	100.00	1.00	1.00	1.00	1.00
4	100.00	1.00	1.00	1.00	1.00	100.00	1.00	1.00	1.00	1.00
5	100.00	1.00	1.00	1.00	1.00	97.22	0.75	0.99	0.74	0.86
6	100.00	1.00	1.00	1.00	1.00	100.00	1.00	1.00	1.00	1.00
7	100.00	1.00	1.00	1.00	1.00	98.61	1.00	0.99	0.89	0.99
8	100.00	1.00	1.00	1.00	1.00	98.61	0.75	1.00	0.86	0.87
9	100.00	1.00	1.00	1.00	1.00	98.61	1.00	0.99	0.89	0.99
10	100.00	1.00	1.00	1.00	1.00	100.00	1.00	1.00	1.00	1.00
11	100.00	1.00	1.00	1.00	1.00	100.00	1.00	1.00	1.00	1.00
12	100.00	1.00	1.00	1.00	1.00	97.22	0.75	0.99	0.74	0.86
13	100.00	1.00	1.00	1.00	1.00	100.00	1.00	1.00	1.00	1.00
14	100.00	1.00	1.00	1.00	1.00	100.00	1.00	1.00	1.00	1.00
15	100.00	1.00	1.00	1.00	1.00	100.00	1.00	1.00	1.00	1.00
16	100.00	1.00	1.00	1.00	1.00	97.22	0.75	0.99	0.74	0.86
17	100.00	1.00	1.00	1.00	1.00	100.00	1.00	1.00	1.00	1.00
18	100.00	1.00	1.00	1.00	1.00	97.22	0.75	0.99	0.74	0.86

**Table 4:** The major parameters of SVM model based on FT-MIR full spectra data.

Class	Calibration Set					Validation Set				
	ACC (%)	SE	SP	MCC	EFF	ACC (%)	SE	SP	MCC	EFF
1	100.00	1.00	1.00	1.00	1.00	100.00	1.00	1.00	1.00	1.00
2	100.00	1.00	1.00	1.00	1.00	100.00	1.00	1.00	1.00	1.00
3	100.00	1.00	1.00	1.00	1.00	100.00	1.00	1.00	1.00	1.00
4	100.00	1.00	1.00	1.00	1.00	100.00	1.00	1.00	1.00	1.00
5	100.00	1.00	1.00	1.00	1.00	100.00	1.00	1.00	1.00	1.00
6	100.00	1.00	1.00	1.00	1.00	100.00	1.00	1.00	1.00	1.00
7	100.00	1.00	1.00	1.00	1.00	100.00	1.00	1.00	1.00	1.00
8	100.00	1.00	1.00	1.00	1.00	100.00	1.00	1.00	1.00	1.00
9	100.00	1.00	1.00	1.00	1.00	100.00	1.00	1.00	1.00	1.00
10	100.00	1.00	1.00	1.00	1.00	100.00	1.00	1.00	1.00	1.00
11	100.00	1.00	1.00	1.00	1.00	100.00	1.00	1.00	1.00	1.00
12	100.00	1.00	1.00	1.00	1.00	100.00	1.00	1.00	1.00	1.00
13	100.00	1.00	1.00	1.00	1.00	100.00	1.00	1.00	1.00	1.00
14	100.00	1.00	1.00	1.00	1.00	100.00	1.00	1.00	1.00	1.00
15	100.00	1.00	1.00	1.00	1.00	100.00	1.00	1.00	1.00	1.00
16	100.00	1.00	1.00	1.00	1.00	100.00	1.00	1.00	1.00	1.00
17	100.00	1.00	1.00	1.00	1.00	100.00	1.00	1.00	1.00	1.00
18	100.00	1.00	1.00	1.00	1.00	100.00	1.00	1.00	1.00	1.00

**Table 5:** The major parameters of K-nearest neighbors (KNN) model based on NIR full spectra data.

Class	Calibration Set					Validation Set				
	ACC (%)	SE	SP	MCC	EFF	ACC (%)	SE	SP	MCC	EFF
1	100.00	1.00	1.00	1.00	1.00	98.61	1.00	0.99	0.89	0.99
2	100.00	1.00	1.00	1.00	1.00	97.22	0.50	1.00	0.70	0.71
3	100.00	1.00	1.00	1.00	1.00	100.00	1.00	1.00	1.00	1.00
4	100.00	1.00	1.00	1.00	1.00	100.00	1.00	1.00	1.00	1.00
5	100.00	1.00	1.00	1.00	1.00	95.83	0.75	0.97	0.65	0.85
6	100.00	1.00	1.00	1.00	1.00	100.00	1.00	1.00	1.00	1.00
7	100.00	1.00	1.00	1.00	1.00	98.61	0.75	1.00	0.86	0.87
8	100.00	1.00	1.00	1.00	1.00	100.00	1.00	1.00	1.00	1.00
9	100.00	1.00	1.00	1.00	1.00	97.22	0.75	0.99	0.74	0.86
10	100.00	1.00	1.00	1.00	1.00	100.00	1.00	1.00	1.00	1.00
11	100.00	1.00	1.00	1.00	1.00	100.00	1.00	1.00	1.00	1.00
12	100.00	1.00	1.00	1.00	1.00	95.83	0.75	0.97	0.65	0.85
13	100.00	1.00	1.00	1.00	1.00	100.00	1.00	1.00	1.00	1.00
14	100.00	1.00	1.00	1.00	1.00	100.00	1.00	1.00	1.00	1.00
15	100.00	1.00	1.00	1.00	1.00	100.00	1.00	1.00	1.00	1.00
16	100.00	1.00	1.00	1.00	1.00	97.22	0.75	0.99	0.74	0.86
17	100.00	1.00	1.00	1.00	1.00	100.00	1.00	1.00	1.00	1.00
18	100.00	1.00	1.00	1.00	1.00	97.22	0.75	0.99	0.74	0.86

**Table 6:** The major parameters of KNN model based on FT-MIR full spectra data.

Class	Calibration Set					Validation Set				
	ACC (%)	SE	SP	MCC	EFF	ACC (%)	SE	SP	MCC	EFF
1	100.00	1.00	1.00	1.00	1.00	100.00	1.00	1.00	1.00	1.00
2	100.00	1.00	1.00	1.00	1.00	100.00	1.00	1.00	1.00	1.00
3	100.00	1.00	1.00	1.00	1.00	100.00	1.00	1.00	1.00	1.00
4	100.00	1.00	1.00	1.00	1.00	98.61	1.00	0.99	0.89	0.99
5	100.00	1.00	1.00	1.00	1.00	100.00	1.00	1.00	1.00	1.00
6	100.00	1.00	1.00	1.00	1.00	100.00	1.00	1.00	1.00	1.00
7	100.00	1.00	1.00	1.00	1.00	100.00	1.00	1.00	1.00	1.00
8	100.00	1.00	1.00	1.00	1.00	97.22	1.00	0.97	0.80	0.99
9	100.00	1.00	1.00	1.00	1.00	97.22	0.50	1.00	0.70	0.71
10	100.00	1.00	1.00	1.00	1.00	100.00	1.00	1.00	1.00	1.00
11	100.00	1.00	1.00	1.00	1.00	98.61	1.00	0.99	0.89	0.99
12	100.00	1.00	1.00	1.00	1.00	100.00	1.00	1.00	1.00	1.00
13	100.00	1.00	1.00	1.00	1.00	100.00	1.00	1.00	1.00	1.00
14	100.00	1.00	1.00	1.00	1.00	100.00	1.00	1.00	1.00	1.00
15	100.00	1.00	1.00	1.00	1.00	100.00	1.00	1.00	1.00	1.00
16	100.00	1.00	1.00	1.00	1.00	97.22	0.50	1.00	0.70	0.71
17	100.00	1.00	1.00	1.00	1.00	100.00	1.00	1.00	1.00	1.00
18	100.00	1.00	1.00	1.00	1.00	100.00	1.00	1.00	1.00	1.00

**Table 7:** The major parameters (accuracy and kappa) of classification models based on different NIR feature variables.

Model	Hyperparameters	Calibration Set	Validation Set	
		Total ACC (%)	Total ACC (%)	K
VIP-NIR-RF	*n_tree_* = 1774, *m_try_* = 14	100	97.22	0.97
Bor-NIR-RF	*n_tree_* = 452, *m_try_* = 11	100	91.67	0.91
GARF-NIR-RF	*n_tree_* = 678, *m_try_* = 22	100	91.67	0.91
GASVM-NIR-RF	*n_tree_* = 1763, *m_try_* = 34	100	91.67	0.91
Ven-NIR-RF	*n_tree_* = 1511, *m_try_* = 2	100	94.44	0.94
VIP-NIR-SVM	*kernel* = linear, *cost* = 0.01	100	97.22	0.97
Bor-NIR-SVM	*kernel* = linear, *cost* = 0.05	100	98.61	0.99
GARF-NIR-SVM	*kernel* = linear, *cost* = 0.1	100	93.06	0.93
GASVM-NIR-SVM	*kernel* = linear, *cost* = 0.05	100	91.67	0.91
Ven-NIR-SVM	*kernel* = linear, *cost* = 0.05	100	98.61	0.99
VIP-NIR-KNN	*k* = 1	100	95.83	0.96
Bor-NIR-KNN	*k* = 1	100	94.44	0.94
GARF-NIR-KNN	*k* = 1	100	87.50	0.87
GASVM-NIR-KNN	*k* = 1	100	88.89	0.88
Ven-NIR-KNN	*k* = 1	100	94.44	0.94

Note: VIP-NIR, Bor-NIR, GARF-NIR, GASVM-NIR and Ven-NIR were feature subsets of NIR extracted by VIP, Boruta, GARF, SVM and their common overlap variables.

**Table 8:** The major parameters (accuracy and kappa) of classification models based on different FT-MIR feature variables.

Model	Hyperparameter	Calibration Set	Validation Set	
		Total ACC (%)	Total ACC (%)	K
VIP-MIR-RF	*n_tree_* = 1334, *m_try_* = 23	100	97.22	0.97
Bor-MIR-RF	*n_tree_* = 1673, *m_try_* = 13	100	95.83	0.96
GARF-MIR-RF	*n_tree_* = 958, *m_try_* = 20	100	95.83	0.96
GASVM-MIR-RF	*n_tree_* = 297 *m_try_* = 31	100	94.44	0.94
Ven-MIR-RF	*n_tree_* = 190, *m_try_* = 10	100	98.61	0.99
VIP-MIR-SVM	*kernel* = linear, *cost* = 0.05	100	100	1.00
Bor-MIR-SVM	*kernel* = linear, *cost* = 0.5	100	100	1.00
GARF-MIR-SVM	*kernel* = linear, *cost* = 0.10	100	100	1.00
GASVM-MIR-SVM	*kernel* = linear, *cost* = 1.00	100	100	1.00
Ven-MIR-SVM	*kernel* = linear, *cost* = 1.00	100	98.61	0.99
VIP-MIR-KNN	*k* = 1	100	98.61	0.99
Bor-MIR-KNN	*k* = 1	100	97.22	0.97
GARF-MIR-KNN	*k* = 1	100	95.83	0.96
GASVM-MIR-KNN	*k* = 1	100	94.44	0.94
Ven-MIR-KNN	*k* = 1	100	97.22	0.97

Note: VIP-MIR, Bor-MIR, GARF-MIR, GASVM-MIR and Ven-MIR were feature subsets of FT-MIR extracted by VIP, Boruta, GARF, SVM, and their common overlap variables.

**Table 9:** The major parameters (accuracy and kappa) of the stacking models.

Scenario	Data Set	Model	Level 1	Calibration Set	Validation Set	
				Total ACC (%)	Total ACC (%)	K
A	Ven-NIR	SG-Ven-NIR- RF	RF	100.00	98.61	0.99
B	Ven-NIR	SG-Ven-NIR- SVM	SVM	100.00	97.22	0.97
C	Ven-NIR	SG-Ven-NIR- KNN	KNN	100.00	95.83	0.96
D	Ven-MIR	SG-Ven-MIR- RF	RF	100.00	94.44	0.94
E	Ven-MIR	SG-Ven-MIR- SVM	SVM	100.00	100.00	1.00
F	Ven-MIR	SG-Ven-MIR- KNN	KNN	100.00	90.28	0.90

Note: base learners (level-0) of all stacking models were RF and SNV models

**Table 10:** The major parameters (accuracy and kappa) of the data fusion models.

Data Fusion Strategy	Number of Variables	Models	Calibration Set	Validation Set	
			Total ACC (%)	Total ACC (%)	K
Low-level fusion	2701	Low-RF	100.00	97.22	0.97
Low-level fusion	2701	Low-SVM	100.00	100.00	1.00
Low-level fusion	2701	Low-KNN	100.00	97.22	0.97
Mid-level fusion	174	Mid-RF	100.00	100.00	1.00
Mid-level fusion	174	Mid-SVM	100.00	100.00	1.00
Mid-level fusion	174	Mid-KNN	100.00	100.00	1.00

**Table 11:** Source of 180 *Gentian* and *Tripterospermum* species samples.

Class	Genus	Species	Geographical Location
1	*Gentiana*	*G. rigescens*	Yongde, Lincang, Yunnan, China
2	*Gentiana*	*G. cephalantha*	Xuyong, Luzhou, Sichuan, China
3	*Gentiana*	*G. davidii*	Jianghua, Yongzhou, Hunan, China
4	*Gentiana*	*G. lawrencei* var. *farreri*	Songpan, Aba, Sichuan, China
5	*Gentiana*	*G. stragulata*	Songpan, Aba, Sichuan, China
6	*Gentiana*	*G. crassicaulis*	Lanping, Nujiang, Yunnan, China
7	*Gentiana*	*G. loureirii*	Jianghua, Yongzhou, Hunan, China
8	*Gentiana*	*G. napulifera*	Liping, QianDong-nan, Guizhou, China
9	*Gentiana*	*G. praticola*	Liping, QianDong-nan, Guizhou, China
10	*Gentiana*	*G. piasezkii*	Ningqiang, Hanzhong, Shaanxi, China
11	*Gentiana*	*G. squarrosa*	Songpan, Aba, Sichuan, China
12	*Gentiana*	*G. pseudosquarrosa*	Songpan, Aba, Sichuan, China
13	*Gentiana*	*G. rubicunda*	Xianfeng, Enshi, Hubei, China
14	*Gentiana*	*G. rubicunda* var. *samolifolia*	Wufeng, Yichang, Hubei, China
15	*Gentiana*	*G. rhodantha*	Nayong, Bijie, Guizhou, China
16	*Gentiana*	*G. striata*	Songpan, Aba, Sichuan, China
17	*Tripterospermum*	*T. chinense*	Tonggu, Yichun, Jiangxi, China
18	*Tripterospermum*	*T. cordatum*	Tonggu, Yichun, Jiangxi, China

**Table 12:** Sample information including their application in southwest of China.

Species	Chinese Name	Disease	Ch.P.
*G. rigescens*	Dian Longdan	heat-clearing, liver protection, icterohepatitis, Japanese encephalitis, cephalalgia, swelling and pain of eye [9,13]	listed (2015 edition) [9]
*G. cephalantha*	Tou hua Longdan	heat-clearing, icterohepatitis	unlisted
*G. davidii*	Wu ling Longdan	heat-clearing, urinary tract infection, conjunctivitis [13]	unlisted
*G. lawrencei* var. *farreri*	Xian ye Longdan	trachitis, cough, smallpox [13]	unlisted
*G. stragulata*	Shi e Longdan	none reported	unlisted
*G. crassicaulis*	Cu jing qin jiao	heat-clearing, icterohepatitis, hematochezia, rheumatism [9]	listed (2015 edition) [9]
*G. loureirii*	Hua nan Longdan	heat-clearing, icterohepatitis, diarrhea, swelling and pain of eye [13]	unlisted
*G. napulifera*	Fu gen Longdan	none reported	unlisted
*G. praticola*	Cao dian Longdan	heat-clearing, detumescence analgesic [13]	unlisted
*G. piasezkii*	Shan nan Longdan	none reported	unlisted
*G. squarrosa*	Lin ye Longdan	heat-clearing, acute appendicitis, swelling and pain of eye [13]	unlisted
*G. pseudosquarrosa*	Jia lin ye Longdan	none reported	unlisted
*G. rubicunda*	Shen hong Longdan	dyspepsia, bone fracture, snakebite, diminish inflammation [13]	unlisted
*G. rubicunda* var. *samolifolia*	Xiao fan lu ye Longdan	none reported	unlisted
*G. rhodantha*	Hong hua Longdan	heat-clearing, diminish inflammation, urinary tract infection, cold, icterohepatitis, diarrhea, scald [9,13]	listed (2015 edition) [9]
*G. striata*	Tiao wen Longdan	none reported	unlisted
*T. chinense*	Shuang hudie	heat-clearing, phthisis, pulmonary abscess, irregular menstruation [13]	unlisted
*T. cordatum*	E mei Shuang hudie	bone fracture [13]	unlisted

## Supplementary Materials

The following are available online. Figure S1. The *n_tree_* (left figure) and *m_try_* (right figure) screening of RF models based on Bayesian optimization methodology and NIR full spectra data, Figure S2. The *n_tree_* (left figure) and *m_try_* (right figure) screening of RF models based on Bayesian optimization methodology and FT-MIR full spectra data, Figure S3. The kernel (left figure) and cost (right figure) screening of SVM models based on Bayesian optimization methodology and NIR full spectra data, Figure S4. The *kernel* (left figure) and cost (right figure) screening of SVM models based on Bayesian optimization methodology and FT-MIR full spectra data, Figure S5. The *K* value screening of KNN models based on Bayesian optimization methodology, Figure S6. The *n_tree_* (left figure) and *m_try_* (right figure) screening of RF models based on Bayesian optimization methodology and NIR feature variables, Figure S7. The kernel (left figures) and cost (right figures) screening of SVM models based on Bayesian optimization methodology and NIR feature variables, Figure S8. The *K* value screening of KNN models based on Bayesian optimization methodology and NIR feature variables, Figure S9. The *n_tree_* (left figures) and *m_try_* (right figures) screening of RF models based on Bayesian optimization methodology and FT-MIR feature variables, Figure S10. The kernel (left figures) and cost (right figures) screening of SVM models based on Bayesian optimization methodology and FT-MIR feature variables, Figure S11. The *K* value screening of KNN models based on Bayesian optimization methodology and FT-MIR feature variables, Figure S12. The *n_tree_* (left figures) and *m_try_* (right figures) screening of RF models based on Bayesian optimization methodology and data fusion strategy, Figure S13. The kernel (left figures) and *cost* (right figures) screening of SVM models based on Bayesian optimization methodology and data fusion strategy, Figure S14. The *K* value screening of KNN models based on Bayesian optimization methodology and data fusion strategy, Table S1. Confusion matrixes of the calibration set and validation set of RF model based on NIR full spectra data, Table S2. Confusion matrixes of the calibration set and validation set of RF model based on FT-MIR full spectra data, Table S3. Confusion matrixes of the calibration set and validation set of SVM model based on NIR full spectra data, Table S4. Confusion matrixes of the calibration set and validation set of SVM model based on FT-MIR full spectra data, Table S5. Confusion matrixes of the calibration set and validation set of KNN model based on NIR full spectra data, Table S6. Confusion matrixes of the calibration set and validation set of KNN model based on FT-MIR full spectra data, Table S7. Confusion matrixes of the calibration set and validation set of VIP-NIR-RF, Table S8. Confusion matrixes of the calibration set and validation set of Bor-NIR-RF, Table S9. Confusion matrixes of the calibration set and validation set of GARF-NIR-RF, Table S10. Confusion matrixes of the calibration set and validation set of GASVM-NIR-RF, Table S11. Confusion matrixes of the calibration set and validation set of Ven-NIR-RF, Table S12. Confusion matrixes of the calibration set and validation set of VIP-NIR-SVM, Table S13. Confusion matrixes of the calibration set and validation set of Bor-NIR-SVM, Table S14. Confusion matrixes of the calibration set and validation set of GARF-NIR-SVM, Table S15. Confusion matrixes of the calibration set and validation set of GASVM-NIR-SVM, Table S16. Confusion matrixes of the calibration set and validation set of Ven-NIR-SVM, Table S17. Confusion matrixes of the calibration set and validation set of VIP-NIR-KNN, Table S18. Confusion matrixes of the calibration set and validation set of Bor-NIR-KNN, Table S19. Confusion matrixes of the calibration set and validation set of GARF-NIR-KNN, Table S20. Confusion matrixes of the calibration set and validation set of GASVM-NIR-KNN, Table S21. Confusion matrixes of the calibration set and validation set of Ven-NIR-KNN, Table S22. Confusion matrixes of the calibration set and validation set of VIP-MIR-RF, Table S23. Confusion matrixes of the calibration set and validation set of Bor-MIR-RF, Table S24. Confusion matrixes of the calibration set and validation set of GARF-MIR-RF, Table S25. Confusion matrixes of the calibration set and validation set of GASVM-MIR-RF, Table S26. Confusion matrixes of the calibration set and validation set of Ven-MIR-RF, Table S27. Confusion matrixes of the calibration set and validation set of VIP-MIR-SVM, Table S28. Confusion matrixes of the calibration set and validation set of Bor-MIR-SVM, Table S29. Confusion matrixes of the calibration set and validation set of GARF-MIR-SVM, Table S30. Confusion matrixes of the calibration set and validation set of GASVM-MIR-SVM, Table S31. Confusion matrixes of the calibration set and validation set of Ven-MIR-SVM, Table S32. Confusion matrixes of the calibration set and validation set of VIP-MIR-KNN, Table S33. Confusion matrixes of the calibration set and validation set of Bor-MIR-KNN, Table S34. Confusion matrixes of the calibration set and validation set of GARF-MIR-KNN, Table S35. Confusion matrixes of the calibration set and validation set of GASVM-MIR-KNN, Table S36. Confusion matrixes of the calibration set and validation set of Ven-MIR-KNN, Table S37. Confusion matrixes of the calibration set and validation set of SG-Ven-NIR-RF, Table S38. Confusion matrixes of the calibration set and validation set of SG-Ven-NIR-SVM, Table S39. Confusion matrixes of the calibration set and validation set of SG-Ven-NIR-KNN, Table S40. Confusion matrixes of the calibration set and validation set of SG-Ven-MIR-RF, Table S41. Confusion matrixes of the calibration set and validation set of SG-Ven-MIR-SVM, Table S42. Confusion matrixes of the calibration set and validation set of SG-Ven-MIR-KNN, Table S43. Confusion matrixes of the calibration set and validation set of Low-RF, Table S44. Confusion matrixes of the calibration set and validation set of Low-SVM, Table S45. Confusion matrixes of the calibration set and validation set of Low-KNN, Table S46. Confusion matrixes of the calibration set and validation set of Mid-RF, Table S47. Confusion matrixes of the calibration set and validation set of Mid-SVM, Table S48. Confusion matrixes of the calibration set and validation set of Mid-KNN.
