# Supplementary material for: Discrimination of Gentiana and Its Related Species Using IR Spectroscopy Combined with Feature Selection and Stacked Generalization
Source: Molecules. 2020 Mar 23;25(6):1442. doi: 10.3390/molecules25061442 (PMC7144467; doi:10.3390/molecules25061442)
Supplement: Supplementary file 1 [file molecules-25-01442-s001.pdf]

Discrimination of Chinese medicinal *Gentiana* and its related species using IR spectroscopy combined with feature selection and stacked generalization

Tao Shen [1, 2, 3], Hong Yu [1, 2*], Yuan-Zhong Wang [4]

[1] Yunnan Herbal Laboratory, Institute of Herb Biotic Resources, School of Life and Sciences, Yunnan University, Kunming 650091, China; st_yxnu@126.com
[2] The International Joint Research Center for Sustainable Utilization of Cordyceps Bioresources in China (Yunnan) and Southeast Asia, Yunnan University, Kunming 650091, China
[3] College of Chemistry, Biological and Environment, Yuxi Normal University, Yu'xi 653100, Yunnan, China
[4] Institute of Medicinal Plants, Yunnan Academy of Agricultural Sciences, Kunming 650200, China
* * Correspondence: hongyu@ynu.edu.cn (H.Y.); Tel: +86 13700676633

Figure captions

Figure S1. The n_{tree} (left figure) and m_{try} (right figure) screening of RF models based on Bayesian optimization methodology and NIR full spectra data

Figure S2. The n_{tree} (left figure) and m_{try} (right figure) screening of RF models based on Bayesian optimization methodology and FT-MIR full spectra data

Figure S3. The kernel (left figure) and cost (right figure) screening of SVM models based on Bayesian optimization methodology and NIR full spectra data

Figure S4. The kernel (left figure) and cost (right figure) screening of SVM models based on Bayesian optimization methodology and FT-MIR full spectra data

Figure S5. The *K* value screening of KNN models based on Bayesian optimization methodology

Figure S6. The n_{tree} (left figures) and m_{try} (right figures) screening of RF models based on Bayesian optimization methodology and NIR feature variables

Figure S7. The kernel (left figures) and cost (right figures) screening of SVM models based on Bayesian optimization methodology and NIR feature variables

Figure S8. The *K* value screening of KNN models based on Bayesian optimization methodology and NIR feature variables

Figure S9. The n_{tree} (left figures) and m_{try} (right figures) screening of RF models based on Bayesian optimization methodology and FT-MIR feature variables

Figure S10. The kernel (left figures) and cost (right figures) screening of SVM models based on Bayesian optimization methodology and FT-MIR feature variables

Figure S11. The *K* value screening of KNN models based on Bayesian optimization methodology and FT-MIR feature variables

Figure S12. The n_{tree} (left figures) and m_{try} (right figures) screening of RF models based on Bayesian optimization methodology and data fusion strategy

Figure S13. The kernel (left figures) and cost (right figures) screening of SVM models based on Bayesian optimization methodology and data fusion strategy

Figure S14. The *K* value screening of KNN models based on Bayesian optimization methodology and data fusion strategy

Table captions

Table S1. Confusion matrixes of the calibration set and validation set of RF model based on NIR full spectra data

Table S2. Confusion matrixes of the calibration set and validation set of RF model based on FT-MIR full spectra data

Table S3. Confusion matrixes of the calibration set and validation set of SVM model based on NIR full spectra data

Table S4. Confusion matrixes of the calibration set and validation set of SVM model based on FT-MIR full spectra data

Table S5. Confusion matrixes of the calibration set and validation set of KNN model based on NIR full spectra data

Table S6. Confusion matrixes of the calibration set and validation set of KNN model based on FT-MIR full spectra data

Table S7. Confusion matrixes of the calibration set and validation set of VIP-NIR-RF

Table S8. Confusion matrixes of the calibration set and validation set of Bor-NIR-RF

Table S9. Confusion matrixes of the calibration set and validation set of GARF-NIR-RF

Table S10. Confusion matrixes of the calibration set and validation set of GASVM-NIR-RF

Table S11. Confusion matrixes of the calibration set and validation set of Ven-NIR-RF

Table S12. Confusion matrixes of the calibration set and validation set of VIP-NIR-SVM

Table S13. Confusion matrixes of the calibration set and validation set of Bor-NIR-SVM

Table S14. Confusion matrixes of the calibration set and validation set of GARF-NIR-SVM

Table S15. Confusion matrixes of the calibration set and validation set of GASVM-NIR-SVM

Table S16. Confusion matrixes of the calibration set and validation set of Ven-NIR-SVM

Table S17. Confusion matrixes of the calibration set and validation set of VIP-NIR-KNN

Table S18. Confusion matrixes of the calibration set and validation set of Bor-NIR-KNN

Table S19. Confusion matrixes of the calibration set and validation set of GARF-NIR-KNN

Table S20. Confusion matrixes of the calibration set and validation set of GASVM-NIR-KNN

Table S21. Confusion matrixes of the calibration set and validation set of Ven-NIR-KNN

Table S22. Confusion matrixes of the calibration set and validation set of VIP-MIR-RF

Table S23. Confusion matrixes of the calibration set and validation set of Bor-MIR-RF

Table S24. Confusion matrixes of the calibration set and validation set of GARF-MIR-RF

Table S25. Confusion matrixes of the calibration set and validation set of GASVM-MIR-RF

Table S26. Confusion matrixes of the calibration set and validation set of Ven-MIR-RF

Table S27. Confusion matrixes of the calibration set and validation set of VIP-MIR-SVM

Table S28. Confusion matrixes of the calibration set and validation set of Bor-MIR-SVM

Table S29. Confusion matrixes of the calibration set and validation set of GARF-MIR-SVM

Table S30. Confusion matrixes of the calibration set and validation set of GASVM-MIR-SVM
Table S31. Confusion matrixes of the calibration set and validation set of Ven-MIR-SVM
Table S32. Confusion matrixes of the calibration set and validation set of VIP-MIR-KNN
Table S33. Confusion matrixes of the calibration set and validation set of Bor-MIR-KNN
Table S34. Confusion matrixes of the calibration set and validation set of GARF-MIR-KNN
Table S35. Confusion matrixes of the calibration set and validation set of GASVM-MIR-KNN
Table S36. Confusion matrixes of the calibration set and validation set of Ven-MIR-KNN

Table S37. Confusion matrixes of the calibration set and validation set of SG-Ven-NIR-RF
Table S38. Confusion matrixes of the calibration set and validation set of SG-Ven-NIR-SVM
Table S39. Confusion matrixes of the calibration set and validation set of SG-Ven-NIR-KNN
Table S40. Confusion matrixes of the calibration set and validation set of SG-Ven-MIR-RF
Table S41. Confusion matrixes of the calibration set and validation set of SG-Ven-MIR-SVM
Table S42. Confusion matrixes of the calibration set and validation set of SG-Ven-MIR-KNN
Table S43. Confusion matrixes of the calibration set and validation set of Low-RF
Table S44. Confusion matrixes of the calibration set and validation set of Low-SVM
Table S45. Confusion matrixes of the calibration set and validation set of Low-KNN
Table S46. Confusion matrixes of the calibration set and validation set of Mid-RF
Table S47. Confusion matrixes of the calibration set and validation set of Mid-SVM
Table S48. Confusion matrixes of the calibration set and validation set of Mid-KNN

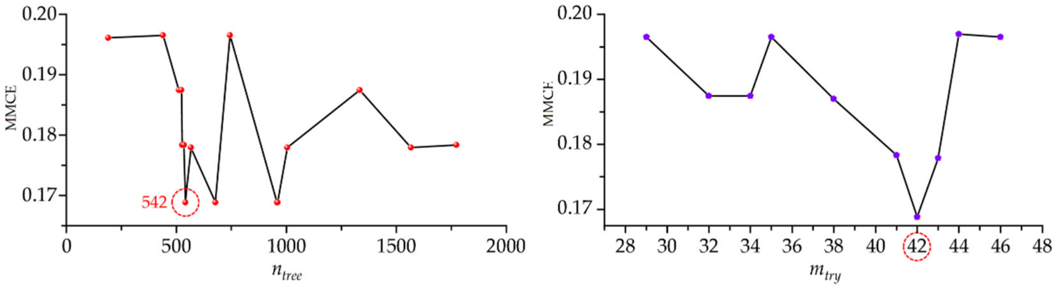

Figure S1. The n_{tree} (left figure) and m_{try} (right figure) screening of RF models based on Bayesian optimization methodology and NIR full spectra data (The optimum value of hyperparameter was in the red circle)

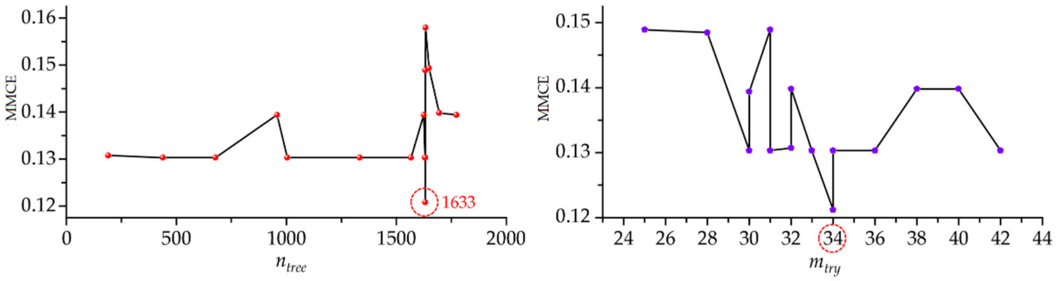

Figure S2. The n_{tree} (left figure) and m_{try} (right figure) screening of RF models based on Bayesian optimization methodology and FT-MIR full spectra data (The optimum value of hyperparameter are shown in the red circle)

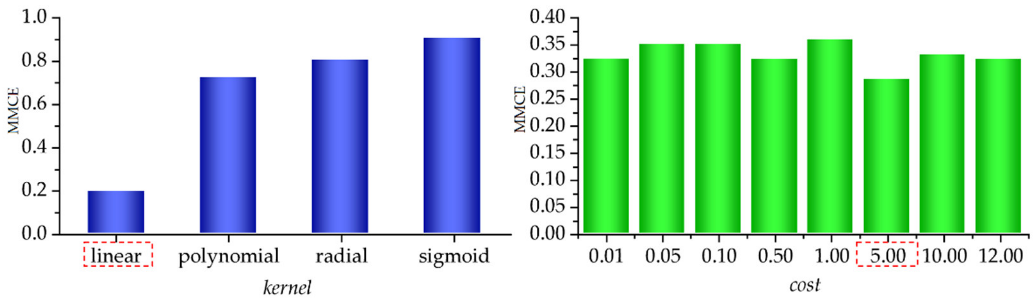

Figure S3. The kernel (left figure) and cost (right figure) screening of SVM models based on Bayesian optimization methodology and NIR full spectra data (The optimum value of hyperparameter are shown in the red box)

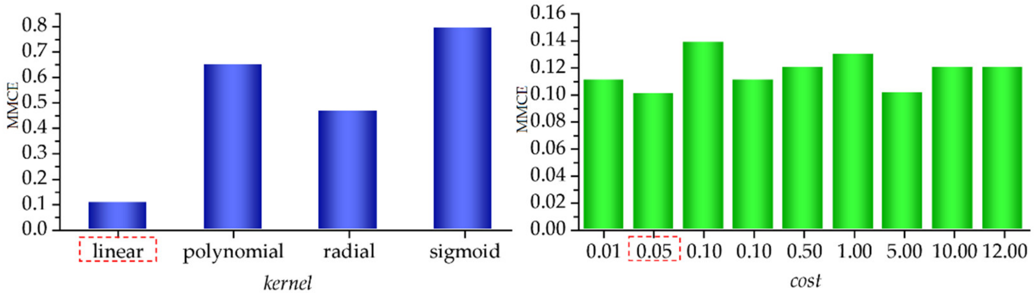

Figure S4. The kernel (left figure) and cost (right figure) screening of SVM models based on Bayesian optimization methodology and FT-MIR full spectra data (The optimum value of hyperparameter are shown in the red box)

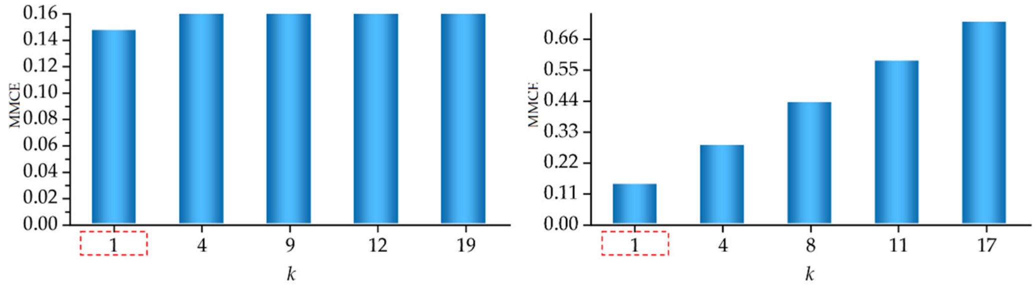

Figure S5. The *K* value (the left figure was NIR data set and the right figure was FT-MIR data set) screening of KNN models based on Bayesian optimization methodology (The optimum value of hyperparameter are shown in the red box)

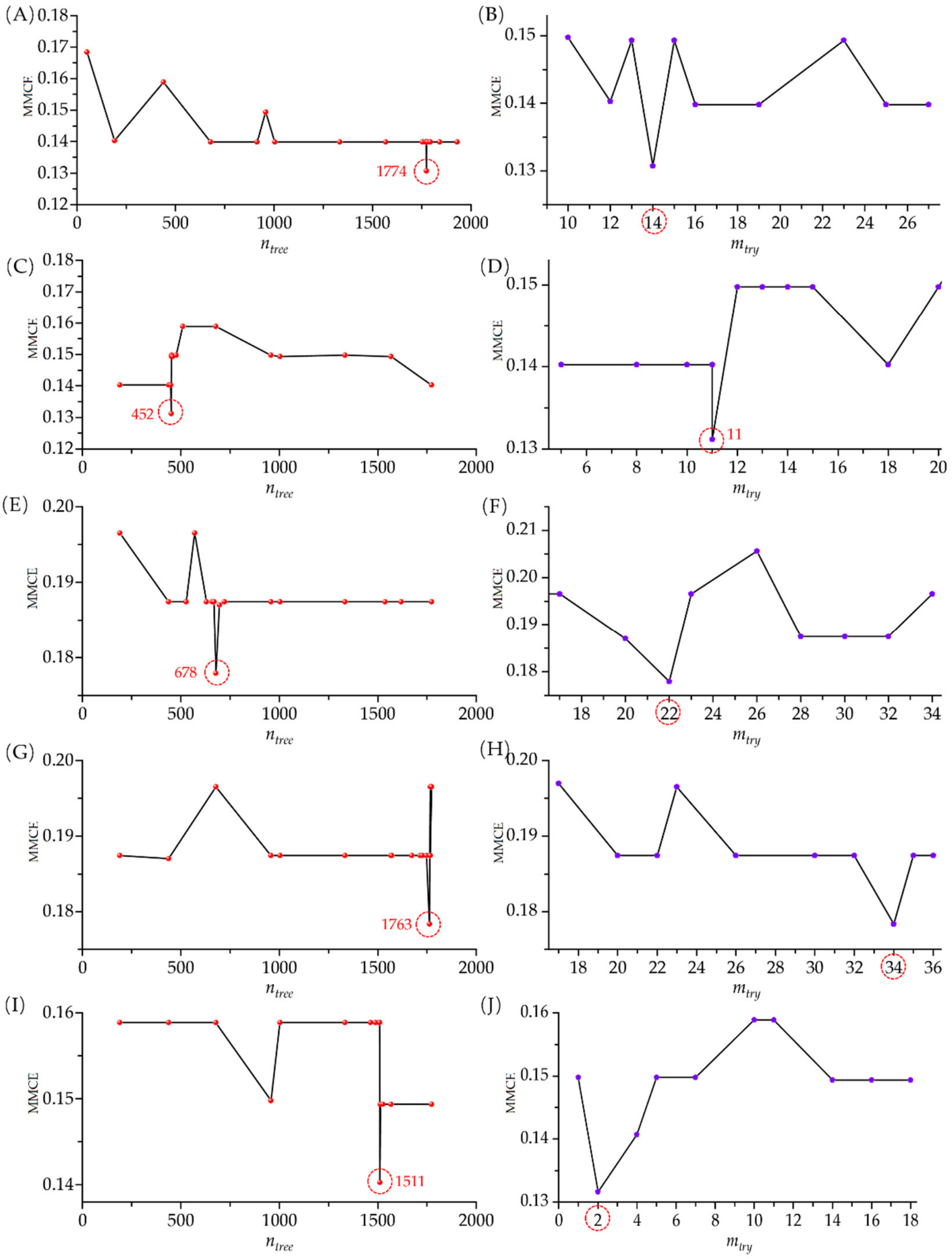

Figure S6. The n_{tree} (left figures) and m_{try} (right figures) screening of RF models based on Bayesian optimization methodology and NIR feature variables (The optimum value of hyperparameter are shown in the red circle)

A, B: VIP-NIR-RF model; C, D: Bor-NIR-RF model; E, F: GARF-NIR-RF model; G, H: GASVM-NIR-RF model; I, J: Ven-NIR-RF model.

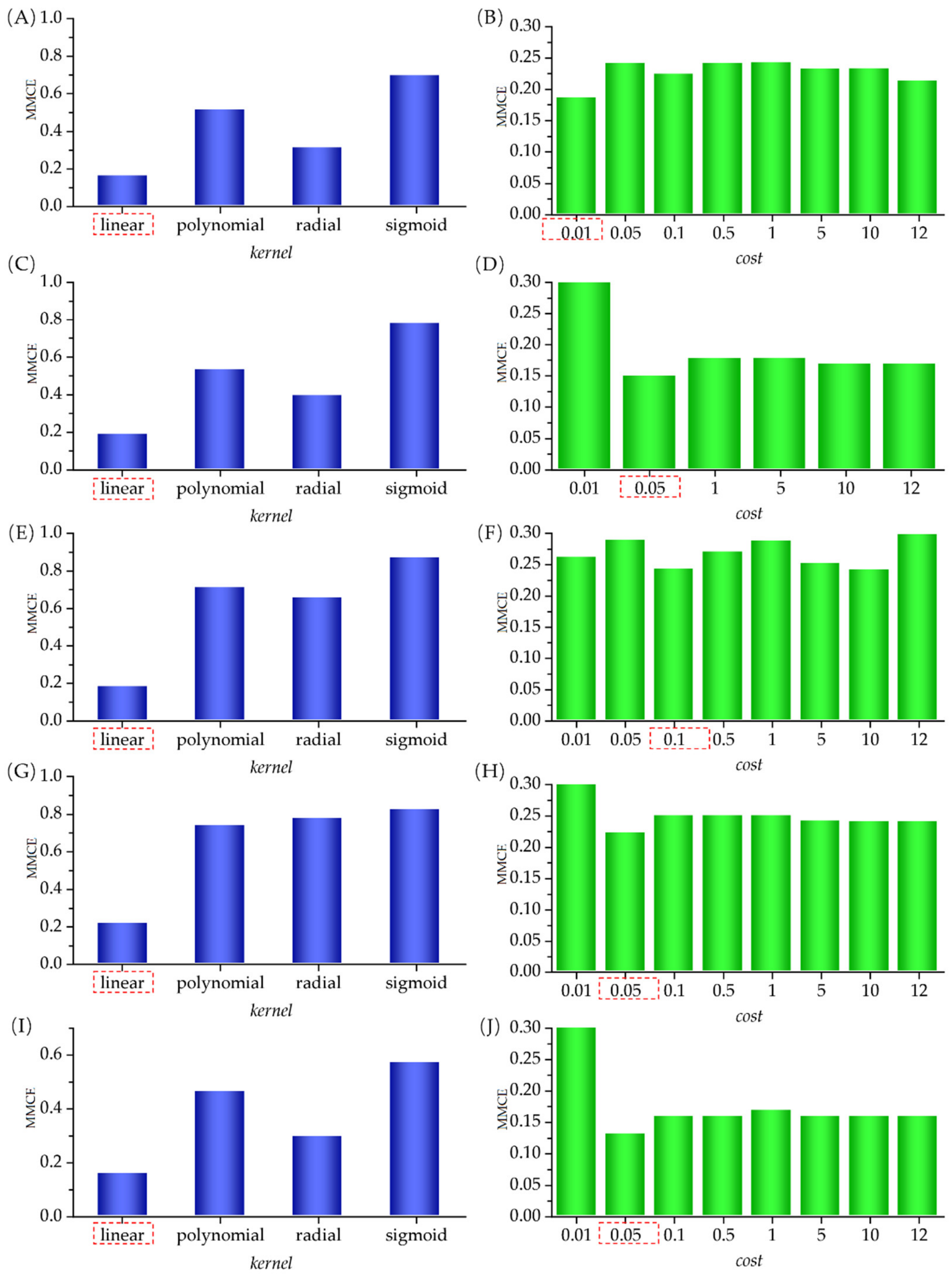

Figure S7. The kernel (left figures) and cost (right figures) screening of SVM models based on Bayesian optimization methodology and NIR feature variables (The optimum value of hyperparameter are shown in the red box)

A, B: VIP-NIR-SVM model; C, D: Bor-NIR-SVM model; E, F: GARF-NIR-SVM model; G, H: GASVM-NIR-SVM model; I, J: Ven-NIR-SVM model.

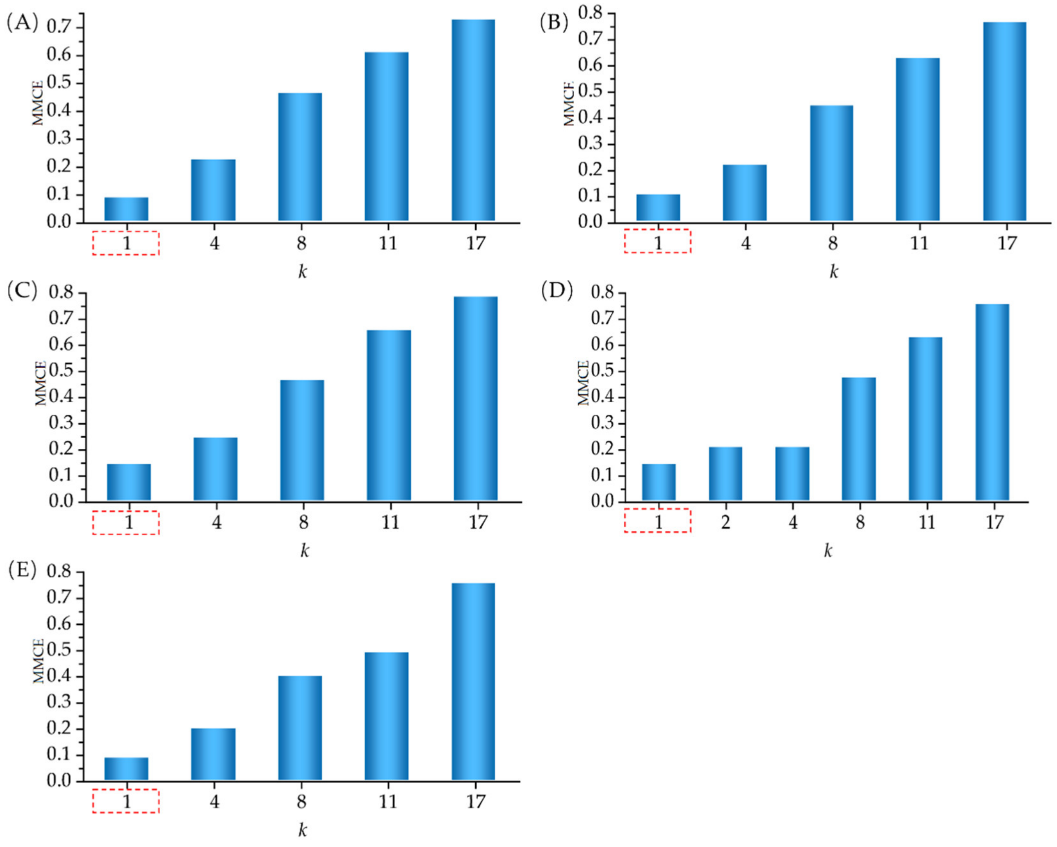

Figure S8. The *K* value screening of KNN models based on Bayesian optimization methodology and NIR feature variables (The optimum value of hyperparameter are shown in the red box)

A: VIP-NIR-KNN model; B: Bor-NIR-KNN model; C: GARF-NIR-KNN model; D: GASVM-NIR-KNN model; E: Ven-NIR-KNN model.

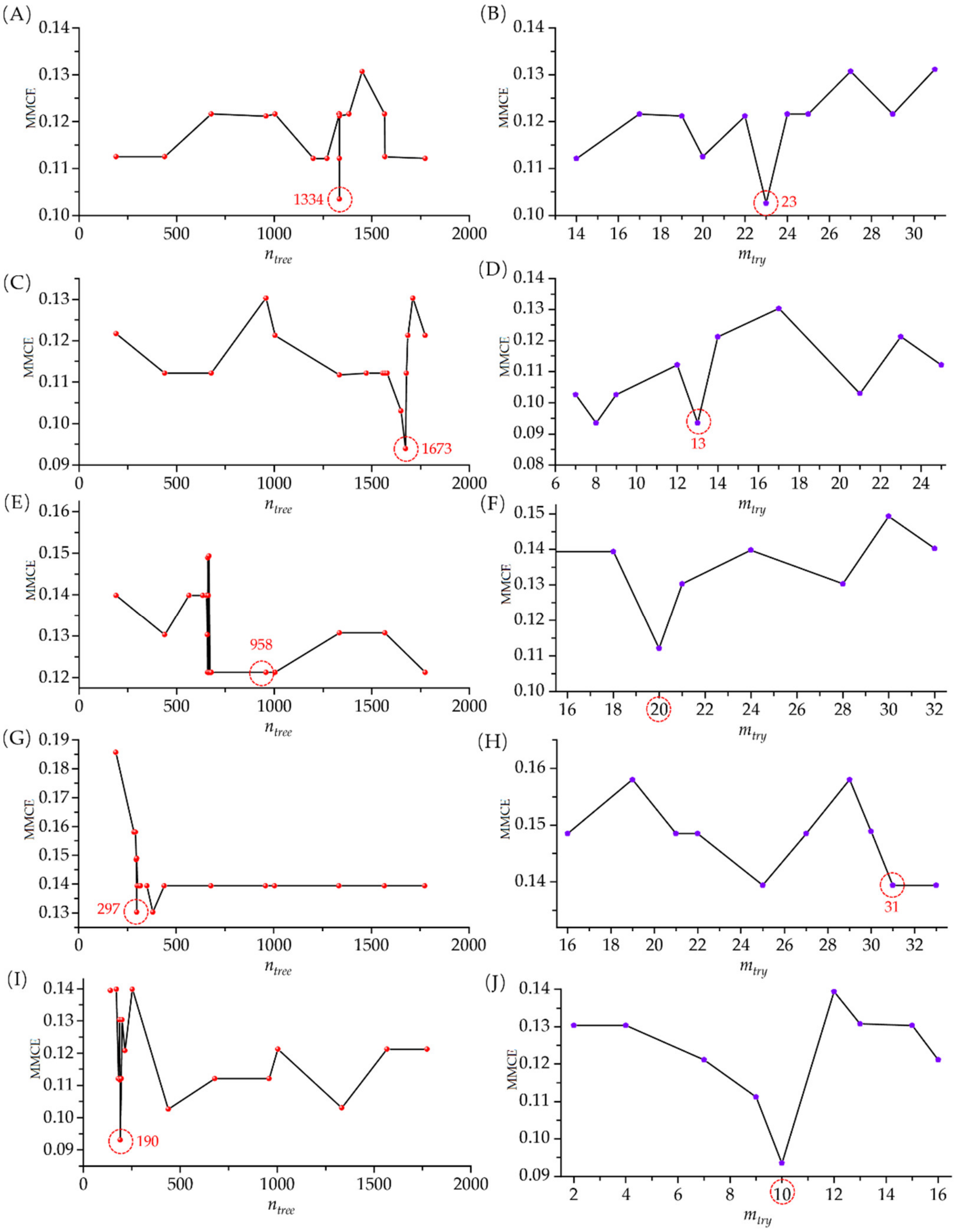

Figure S9. The n_{tree} (left figures) and m_{try} (right figures) screening of RF models based on Bayesian optimization methodology and FT-MIR feature variables (The optimum value of hyperparameter are shown in the red circle)

A, B: VIP-MIR-RF model; C, D: Bor-MIR-RF model; E, F: GARF-MIR-RF model; G, H: GASVM-MIR-RF model; I, J: Ven-MIR-RF model.

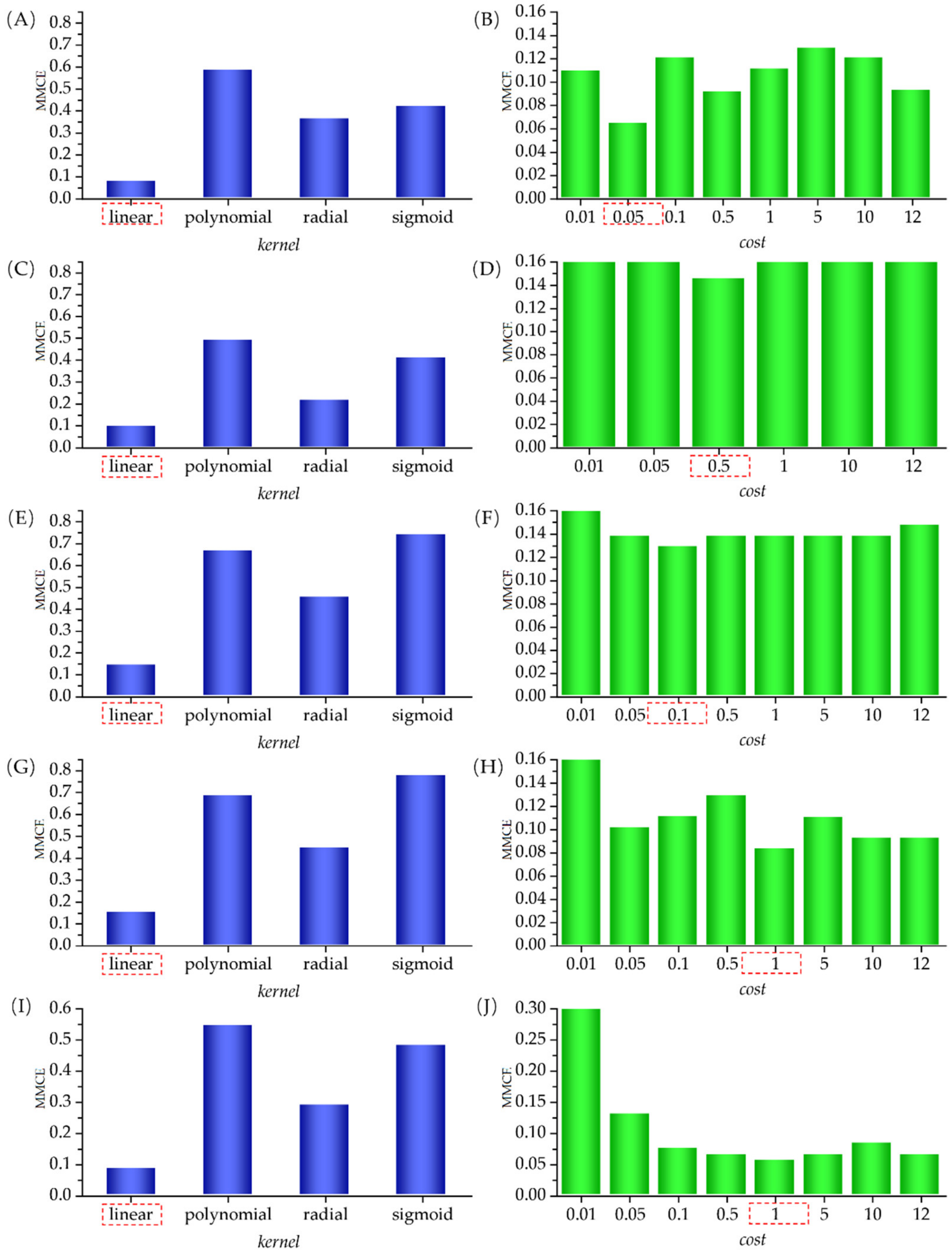

Figure S10. The kernel (left figures) and cost (right figures) screening of SVM models based on Bayesian optimization methodology and FT-MIR feature variables (The optimum value of hyperparameter are shown in the red box)

A, B: VIP-MIR-SVM model; C, D: Bor-MIR-SVM model; E, F: GARF-MIR-SVM model; G, H: GASVM-MIR-SVM model; I, J: Ven-MIR-SVM model.

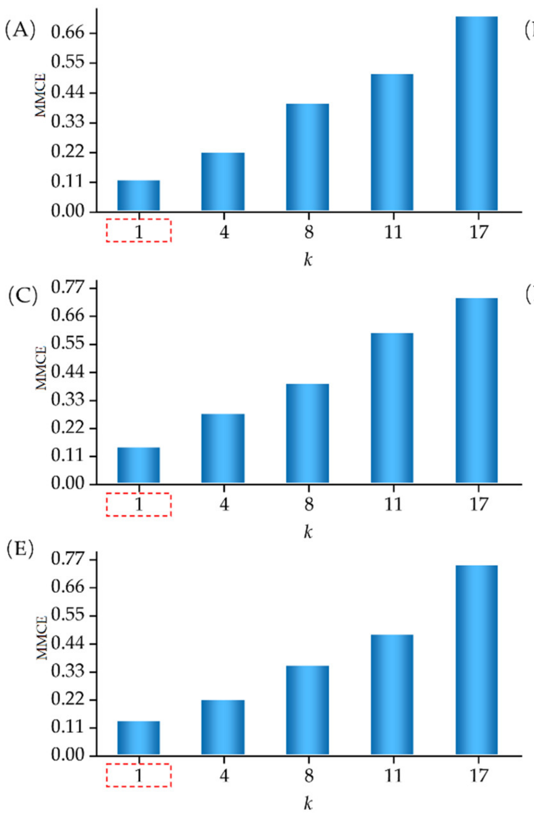

Figure S11. The *K* value screening of KNN models based on Bayesian optimization methodology and FT-MIR feature variables (The optimum value of hyperparameter are shown in the red box)

A: VIP-MIR-KNN model; B: Bor-MIR-KNN model; C: GARF-MIR-KNN model; D: GASVM-MIR-KNN model; E: Ven-MIR-KNN model.

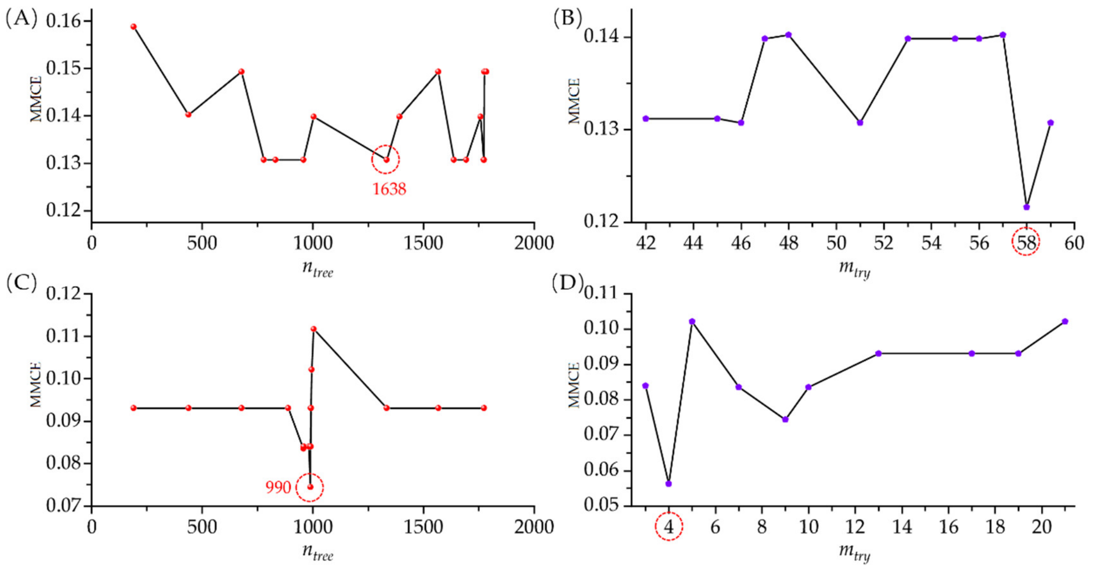

Figure S12. The n_{tree} (left figures) and m_{try} (right figures) screening of RF models based on Bayesian optimization methodology and data fusion strategy (The optimum value of hyperparameter are shown in the red circle)

A, B: low-level fusion model; C, D: mid-level fusion model.

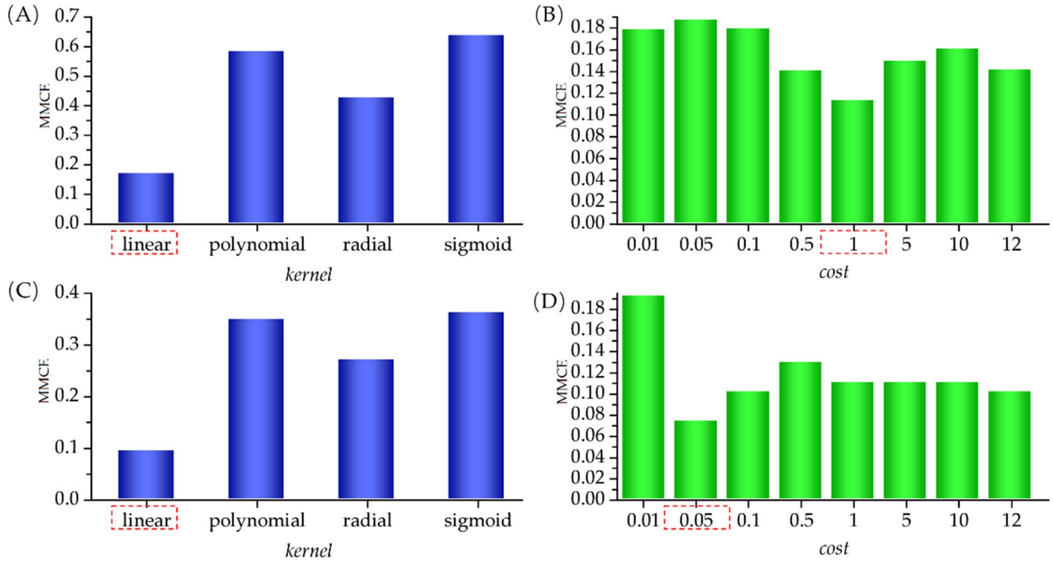

Figure S13. The kernel (left figures) and cost (right figures) screening of SVM models based on Bayesian optimization methodology and data fusion strategy (The optimum value of hyperparameter are shown in the red box)

A, B: low-level fusion model; C, D: mid-level fusion model.

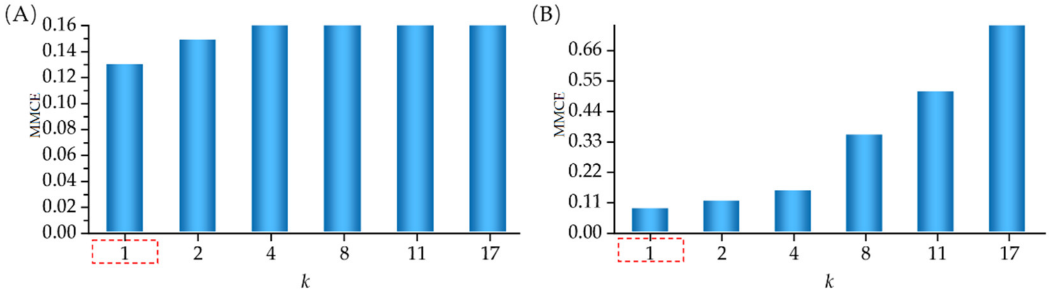

Figure S14. The *K* value screening of KNN models based on Bayesian optimization methodology and data fusion strategy (The optimum value of hyperparameter are shown in the red box)

A: low-level fusion model; B: mid-level fusion model.

Table S1. Confusion matrixes of the calibration set and validation set of RF model based on NIR full spectra data

Calibration set

Class	1	2	3	4	5	6	7	8	9	10	11	12	13	14	15	16	17	18	TP	TN	FP	FN
1	6	0	0	0	0	0	0	0	0	0	0	0	0	0	0	0	0	0	6	102	0	0
2	0	6	0	0	0	0	0	0	0	0	0	0	0	0	0	0	0	0	6	102	0	0
3	0	0	6	0	0	0	0	0	0	0	0	0	0	0	0	0	0	0	6	102	0	0
4	0	0	0	6	0	0	0	0	0	0	0	0	0	0	0	0	0	0	6	102	0	0
5	0	0	0	0	6	0	0	0	0	0	0	0	0	0	0	0	0	0	6	102	0	0
6	0	0	0	0	0	6	0	0	0	0	0	0	0	0	0	0	0	0	6	102	0	0
7	0	0	0	0	0	0	6	0	0	0	0	0	0	0	0	0	0	0	6	102	0	0
8	0	0	0	0	0	0	0	6	0	0	0	0	0	0	0	0	0	0	6	102	0	0
9	0	0	0	0	0	0	0	0	6	0	0	0	0	0	0	0	0	0	6	102	0	0
10	0	0	0	0	0	0	0	0	0	6	0	0	0	0	0	0	0	0	6	102	0	0
11	0	0	0	0	0	0	0	0	0	0	6	0	0	0	0	0	0	0	6	102	0	0
12	0	0	0	0	0	0	0	0	0	0	0	6	0	0	0	0	0	0	6	102	0	0
13	0	0	0	0	0	0	0	0	0	0	0	0	6	0	0	0	0	0	6	102	0	0
14	0	0	0	0	0	0	0	0	0	0	0	0	0	6	0	0	0	0	6	102	0	0
15	0	0	0	0	0	0	0	0	0	0	0	0	0	0	6	0	0	0	6	102	0	0
16	0	0	0	0	0	0	0	0	0	0	0	0	0	0	0	6	0	0	6	102	0	0
17	0	0	0	0	0	0	0	0	0	0	0	0	0	0	0	0	6	0	6	102	0	0
18	0	0	0	0	0	0	0	0	0	0	0	0	0	0	0	0	0	6	6	102	0	0

Validation set

Class	1	2	3	4	5	6	7	8	9	10	11	12	13	14	15	16	17	18	TP	TN	FP	FN
1	4	0	0	0	0	0	0	0	0	0	0	0	0	0	0	0	0	0	4	68	0	0
2	0	4	0	0	0	0	0	0	0	0	0	0	0	0	0	0	0	0	4	68	0	0
3	0	0	4	0	0	0	0	0	0	0	0	0	0	0	0	0	0	0	4	68	0	0
4	0	0	0	4	0	0	0	0	0	0	0	0	0	0	0	0	0	0	4	68	0	0
5	0	0	0	0	3	0	0	0	0	0	0	0	0	0	0	0	0	1	3	67	1	1
6	0	0	0	0	0	4	0	0	0	0	0	0	0	0	0	0	0	0	4	68	0	0
7	0	0	0	0	0	0	4	0	0	0	0	0	0	0	0	0	0	0	4	68	0	0
8	0	0	0	0	0	0	0	3	1	0	0	0	0	0	0	0	0	0	3	68	0	1
9	0	0	0	0	0	0	0	0	4	0	0	0	0	0	0	0	0	0	4	67	1	0
10	0	0	0	0	0	0	0	0	0	4	0	0	0	0	0	0	0	0	4	68	0	0
11	0	0	0	0	0	0	0	0	0	0	4	0	0	0	0	0	0	0	4	68	0	0
12	0	0	0	0	0	0	0	0	0	0	0	3	0	0	0	1	0	0	3	67	1	1
13	0	0	0	0	0	0	0	0	0	0	0	0	4	0	0	0	0	0	4	68	0	0
14	0	0	0	0	0	0	0	0	0	0	0	0	0	4	0	0	0	0	4	68	0	0
15	0	0	0	0	0	0	0	0	0	0	0	0	0	0	4	0	0	0	4	68	0	0
16	0	0	0	0	0	0	0	0	0	0	0	1	0	0	0	3	0	0	3	67	1	1
17	0	0	0	0	0	0	0	0	0	0	0	0	0	0	0	0	4	0	4	68	0	0
18	0	0	0	0	1	0	0	0	0	0	0	0	0	0	0	0	0	3	3	67	1	1

Table S2. Confusion matrixes of the calibration set and validation set of RF model based on FT-MIR full spectra data

Calibration set

Class	1	2	3	4	5	6	7	8	9	10	11	12	13	14	15	16	17	18	TP	TN	FP	FN
1	6	0	0	0	0	0	0	0	0	0	0	0	0	0	0	0	0	0	6	102	0	0
2	0	6	0	0	0	0	0	0	0	0	0	0	0	0	0	0	0	0	6	102	0	0
3	0	0	6	0	0	0	0	0	0	0	0	0	0	0	0	0	0	0	6	102	0	0
4	0	0	0	6	0	0	0	0	0	0	0	0	0	0	0	0	0	0	6	102	0	0
5	0	0	0	0	6	0	0	0	0	0	0	0	0	0	0	0	0	0	6	102	0	0
6	0	0	0	0	0	6	0	0	0	0	0	0	0	0	0	0	0	0	6	102	0	0
7	0	0	0	0	0	0	6	0	0	0	0	0	0	0	0	0	0	0	6	102	0	0
8	0	0	0	0	0	0	0	6	0	0	0	0	0	0	0	0	0	0	6	102	0	0
9	0	0	0	0	0	0	0	0	6	0	0	0	0	0	0	0	0	0	6	102	0	0
10	0	0	0	0	0	0	0	0	0	6	0	0	0	0	0	0	0	0	6	102	0	0
11	0	0	0	0	0	0	0	0	0	0	6	0	0	0	0	0	0	0	6	102	0	0
12	0	0	0	0	0	0	0	0	0	0	0	6	0	0	0	0	0	0	6	102	0	0
13	0	0	0	0	0	0	0	0	0	0	0	0	6	0	0	0	0	0	6	102	0	0
14	0	0	0	0	0	0	0	0	0	0	0	0	0	6	0	0	0	0	6	102	0	0
15	0	0	0	0	0	0	0	0	0	0	0	0	0	0	6	0	0	0	6	102	0	0
16	0	0	0	0	0	0	0	0	0	0	0	0	0	0	0	6	0	0	6	102	0	0
17	0	0	0	0	0	0	0	0	0	0	0	0	0	0	0	0	6	0	6	102	0	0
18	0	0	0	0	0	0	0	0	0	0	0	0	0	0	0	0	0	6	6	102	0	0

Validation set

Class	1	2	3	4	5	6	7	8	9	10	11	12	13	14	15	16	17	18	TP	TN	FP	FN
1	4	0	0	0	0	0	0	0	0	0	0	0	0	0	0	0	0	0	4	68	0	0
2	0	4	0	0	0	0	0	0	0	0	0	0	0	0	0	0	0	0	4	68	0	0
3	0	0	4	0	0	0	0	0	0	0	0	0	0	0	0	0	0	0	4	68	0	0
4	0	0	0	4	0	0	0	0	0	0	0	0	0	0	0	0	0	0	4	68	0	0
5	0	0	0	0	4	0	0	0	0	0	0	0	0	0	0	0	0	0	4	68	0	0
6	0	0	0	0	0	4	0	0	0	0	0	0	0	0	0	0	0	0	4	68	0	0
7	0	0	0	0	0	0	4	0	0	0	0	0	0	0	0	0	0	0	4	68	0	0
8	0	0	0	0	0	0	0	3	1	0	0	0	0	0	0	0	0	0	3	68	0	1
9	0	0	0	0	0	0	0	0	4	0	0	0	0	0	0	0	0	0	4	67	1	0
10	0	0	0	0	0	0	0	0	0	4	0	0	0	0	0	0	0	0	4	67	1	0
11	0	0	0	0	0	0	0	0	0	0	4	0	0	0	0	0	0	0	4	67	1	0
12	0	0	0	0	0	0	0	0	0	0	1	3	0	0	0	0	0	0	3	68	0	1
13	0	0	0	0	0	0	0	0	0	0	0	0	4	0	0	0	0	0	4	68	0	0
14	0	0	0	0	0	0	0	0	0	0	0	0	0	4	0	0	0	0	4	68	0	0
15	0	0	0	0	0	0	0	0	0	0	0	0	0	0	4	0	0	0	4	67	1	0
16	0	0	0	0	0	0	0	0	0	1	0	0	0	0	1	2	0	0	2	68	0	2
17	0	0	0	0	0	0	0	0	0	0	0	0	0	0	0	0	4	0	4	68	0	0
18	0	0	0	0	0	0	0	0	0	0	0	0	0	0	0	0	0	4	4	68	0	0

Table S3. Confusion matrixes of the calibration set and validation set of SVM model based on NIR full spectra data

Calibration set

Class	1	2	3	4	5	6	7	8	9	10	11	12	13	14	15	16	17	18	TP	TN	FP	FN
1	6	0	0	0	0	0	0	0	0	0	0	0	0	0	0	0	0	0	6	102	0	0
2	0	6	0	0	0	0	0	0	0	0	0	0	0	0	0	0	0	0	6	102	0	0
3	0	0	6	0	0	0	0	0	0	0	0	0	0	0	0	0	0	0	6	102	0	0
4	0	0	0	6	0	0	0	0	0	0	0	0	0	0	0	0	0	0	6	102	0	0
5	0	0	0	0	6	0	0	0	0	0	0	0	0	0	0	0	0	0	6	102	0	0
6	0	0	0	0	0	6	0	0	0	0	0	0	0	0	0	0	0	0	6	102	0	0
7	0	0	0	0	0	0	6	0	0	0	0	0	0	0	0	0	0	0	6	102	0	0
8	0	0	0	0	0	0	0	6	0	0	0	0	0	0	0	0	0	0	6	102	0	0
9	0	0	0	0	0	0	0	0	6	0	0	0	0	0	0	0	0	0	6	102	0	0
10	0	0	0	0	0	0	0	0	0	6	0	0	0	0	0	0	0	0	6	102	0	0
11	0	0	0	0	0	0	0	0	0	0	6	0	0	0	0	0	0	0	6	102	0	0
12	0	0	0	0	0	0	0	0	0	0	0	6	0	0	0	0	0	0	6	102	0	0
13	0	0	0	0	0	0	0	0	0	0	0	0	6	0	0	0	0	0	6	102	0	0
14	0	0	0	0	0	0	0	0	0	0	0	0	0	6	0	0	0	0	6	102	0	0
15	0	0	0	0	0	0	0	0	0	0	0	0	0	0	6	0	0	0	6	102	0	0
16	0	0	0	0	0	0	0	0	0	0	0	0	0	0	0	6	0	0	6	102	0	0
17	0	0	0	0	0	0	0	0	0	0	0	0	0	0	0	0	6	0	6	102	0	0
18	0	0	0	0	0	0	0	0	0	0	0	0	0	0	0	0	0	6	6	102	0	0

Validation set

Class	1	2	3	4	5	6	7	8	9	10	11	12	13	14	15	16	17	18	TP	TN	FP	FN
1	3	0	0	0	0	0	1	0	0	0	0	0	0	0	0	0	0	0	3	68	0	1
2	0	4	0	0	0	0	0	0	0	0	0	0	0	0	0	0	0	0	4	68	0	0
3	0	0	4	0	0	0	0	0	0	0	0	0	0	0	0	0	0	0	4	68	0	0
4	0	0	0	4	0	0	0	0	0	0	0	0	0	0	0	0	0	0	4	68	0	0
5	0	0	0	0	3	0	0	0	0	0	0	0	0	0	0	0	0	1	3	67	1	1
6	0	0	0	0	0	4	0	0	0	0	0	0	0	0	0	0	0	0	4	68	0	0
7	0	0	0	0	0	0	4	0	0	0	0	0	0	0	0	0	0	0	4	67	1	0
8	0	0	0	0	0	0	0	3	1	0	0	0	0	0	0	0	0	0	3	68	0	1
9	0	0	0	0	0	0	0	0	4	0	0	0	0	0	0	0	0	0	4	67	1	0
10	0	0	0	0	0	0	0	0	0	4	0	0	0	0	0	0	0	0	4	68	0	0
11	0	0	0	0	0	0	0	0	0	0	4	0	0	0	0	0	0	0	4	68	0	0
12	0	0	0	0	0	0	0	0	0	0	0	3	0	0	0	1	0	0	3	67	1	1
13	0	0	0	0	0	0	0	0	0	0	0	0	4	0	0	0	0	0	4	68	0	0
14	0	0	0	0	0	0	0	0	0	0	0	0	0	4	0	0	0	0	4	68	0	0
15	0	0	0	0	0	0	0	0	0	0	0	0	0	0	4	0	0	0	4	68	0	0
16	0	0	0	0	0	0	0	0	0	0	0	1	0	0	0	3	0	0	3	67	1	1
17	0	0	0	0	0	0	0	0	0	0	0	0	0	0	0	0	4	0	4	68	0	0
18	0	0	0	0	1	0	0	0	0	0	0	0	0	0	0	0	0	3	3	67	1	1

Table S4. Confusion matrixes of the calibration set and validation set of SVM model based on FT-MIR full spectra data

Calibration set

Class	1	2	3	4	5	6	7	8	9	10	11	12	13	14	15	16	17	18	TP	TN	FP	FN
1	6	0	0	0	0	0	0	0	0	0	0	0	0	0	0	0	0	0	6	102	0	0
2	0	6	0	0	0	0	0	0	0	0	0	0	0	0	0	0	0	0	6	102	0	0
3	0	0	6	0	0	0	0	0	0	0	0	0	0	0	0	0	0	0	6	102	0	0
4	0	0	0	6	0	0	0	0	0	0	0	0	0	0	0	0	0	0	6	102	0	0
5	0	0	0	0	6	0	0	0	0	0	0	0	0	0	0	0	0	0	6	102	0	0
6	0	0	0	0	0	6	0	0	0	0	0	0	0	0	0	0	0	0	6	102	0	0
7	0	0	0	0	0	0	6	0	0	0	0	0	0	0	0	0	0	0	6	102	0	0
8	0	0	0	0	0	0	0	6	0	0	0	0	0	0	0	0	0	0	6	102	0	0
9	0	0	0	0	0	0	0	0	6	0	0	0	0	0	0	0	0	0	6	102	0	0
10	0	0	0	0	0	0	0	0	0	6	0	0	0	0	0	0	0	0	6	102	0	0
11	0	0	0	0	0	0	0	0	0	0	6	0	0	0	0	0	0	0	6	102	0	0
12	0	0	0	0	0	0	0	0	0	0	0	6	0	0	0	0	0	0	6	102	0	0
13	0	0	0	0	0	0	0	0	0	0	0	0	6	0	0	0	0	0	6	102	0	0
14	0	0	0	0	0	0	0	0	0	0	0	0	0	6	0	0	0	0	6	102	0	0
15	0	0	0	0	0	0	0	0	0	0	0	0	0	0	6	0	0	0	6	102	0	0
16	0	0	0	0	0	0	0	0	0	0	0	0	0	0	0	6	0	0	6	102	0	0
17	0	0	0	0	0	0	0	0	0	0	0	0	0	0	0	0	6	0	6	102	0	0
18	0	0	0	0	0	0	0	0	0	0	0	0	0	0	0	0	0	6	6	102	0	0

Validation set

Class	1	2	3	4	5	6	7	8	9	10	11	12	13	14	15	16	17	18	TP	TN	FP	FN
1	4	0	0	0	0	0	0	0	0	0	0	0	0	0	0	0	0	0	4	68	0	0
2	0	4	0	0	0	0	0	0	0	0	0	0	0	0	0	0	0	0	4	68	0	0
3	0	0	4	0	0	0	0	0	0	0	0	0	0	0	0	0	0	0	4	68	0	0
4	0	0	0	4	0	0	0	0	0	0	0	0	0	0	0	0	0	0	4	68	0	0
5	0	0	0	0	4	0	0	0	0	0	0	0	0	0	0	0	0	0	4	68	0	0
6	0	0	0	0	0	4	0	0	0	0	0	0	0	0	0	0	0	0	4	68	0	0
7	0	0	0	0	0	0	4	0	0	0	0	0	0	0	0	0	0	0	4	68	0	0
8	0	0	0	0	0	0	0	4	0	0	0	0	0	0	0	0	0	0	4	68	0	0
9	0	0	0	0	0	0	0	0	4	0	0	0	0	0	0	0	0	0	4	68	0	0
10	0	0	0	0	0	0	0	0	0	4	0	0	0	0	0	0	0	0	4	68	0	0
11	0	0	0	0	0	0	0	0	0	0	4	0	0	0	0	0	0	0	4	68	0	0
12	0	0	0	0	0	0	0	0	0	0	0	4	0	0	0	0	0	0	4	68	0	0
13	0	0	0	0	0	0	0	0	0	0	0	0	4	0	0	0	0	0	4	68	0	0
14	0	0	0	0	0	0	0	0	0	0	0	0	0	4	0	0	0	0	4	68	0	0
15	0	0	0	0	0	0	0	0	0	0	0	0	0	0	4	0	0	0	4	68	0	0
16	0	0	0	0	0	0	0	0	0	0	0	0	0	0	0	4	0	0	4	68	0	0
17	0	0	0	0	0	0	0	0	0	0	0	0	0	0	0	0	4	0	4	68	0	0
18	0	0	0	0	0	0	0	0	0	0	0	0	0	0	0	0	0	4	4	68	0	0

Table S5. Confusion matrixes of the calibration set and validation set of KNN model based on NIR full spectra data

Calibration set

Class	1	2	3	4	5	6	7	8	9	10	11	12	13	14	15	16	17	18	TP	TN	FP	FN
1	6	0	0	0	0	0	0	0	0	0	0	0	0	0	0	0	0	0	6	102	0	0
2	0	6	0	0	0	0	0	0	0	0	0	0	0	0	0	0	0	0	6	102	0	0
3	0	0	6	0	0	0	0	0	0	0	0	0	0	0	0	0	0	0	6	102	0	0
4	0	0	0	6	0	0	0	0	0	0	0	0	0	0	0	0	0	0	6	102	0	0
5	0	0	0	0	6	0	0	0	0	0	0	0	0	0	0	0	0	0	6	102	0	0
6	0	0	0	0	0	6	0	0	0	0	0	0	0	0	0	0	0	0	6	102	0	0
7	0	0	0	0	0	0	6	0	0	0	0	0	0	0	0	0	0	0	6	102	0	0
8	0	0	0	0	0	0	0	6	0	0	0	0	0	0	0	0	0	0	6	102	0	0
9	0	0	0	0	0	0	0	0	6	0	0	0	0	0	0	0	0	0	6	102	0	0
10	0	0	0	0	0	0	0	0	0	6	0	0	0	0	0	0	0	0	6	102	0	0
11	0	0	0	0	0	0	0	0	0	0	6	0	0	0	0	0	0	0	6	102	0	0
12	0	0	0	0	0	0	0	0	0	0	0	6	0	0	0	0	0	0	6	102	0	0
13	0	0	0	0	0	0	0	0	0	0	0	0	6	0	0	0	0	0	6	102	0	0
14	0	0	0	0	0	0	0	0	0	0	0	0	0	6	0	0	0	0	6	102	0	0
15	0	0	0	0	0	0	0	0	0	0	0	0	0	0	6	0	0	0	6	102	0	0
16	0	0	0	0	0	0	0	0	0	0	0	0	0	0	0	6	0	0	6	102	0	0
17	0	0	0	0	0	0	0	0	0	0	0	0	0	0	0	0	6	0	6	102	0	0
18	0	0	0	0	0	0	0	0	0	0	0	0	0	0	0	0	0	6	6	102	0	0

Validation set

Class	1	2	3	4	5	6	7	8	9	10	11	12	13	14	15	16	17	18	TP	TN	FP	FN
1	4	0	0	0	0	0	0	0	0	0	0	0	0	0	0	0	0	0	4	67	1	0
2	1	2	0	0	1	0	0	0	0	0	0	0	0	0	0	0	0	0	2	68	0	2
3	0	0	4	0	0	0	0	0	0	0	0	0	0	0	0	0	0	0	4	68	0	0
4	0	0	0	4	0	0	0	0	0	0	0	0	0	0	0	0	0	0	4	68	0	0
5	0	0	0	0	3	0	0	0	0	0	0	0	0	0	0	0	0	1	3	66	2	1
6	0	0	0	0	0	4	0	0	0	0	0	0	0	0	0	0	0	0	4	68	0	0
7	0	0	0	0	0	0	3	0	1	0	0	0	0	0	0	0	0	0	3	68	0	1
8	0	0	0	0	0	0	0	4	0	0	0	0	0	0	0	0	0	0	4	68	0	0
9	0	0	0	0	0	0	0	0	3	0	0	1	0	0	0	0	0	0	3	67	1	1
10	0	0	0	0	0	0	0	0	0	4	0	0	0	0	0	0	0	0	4	68	0	0
11	0	0	0	0	0	0	0	0	0	0	4	0	0	0	0	0	0	0	4	68	0	0
12	0	0	0	0	0	0	0	0	0	0	0	3	0	0	0	1	0	0	3	66	2	1
13	0	0	0	0	0	0	0	0	0	0	0	0	4	0	0	0	0	0	4	68	0	0
14	0	0	0	0	0	0	0	0	0	0	0	0	0	4	0	0	0	0	4	68	0	0
15	0	0	0	0	0	0	0	0	0	0	0	0	0	0	4	0	0	0	4	68	0	0
16	0	0	0	0	0	0	0	0	0	0	0	1	0	0	0	3	0	0	3	67	1	1
17	0	0	0	0	0	0	0	0	0	0	0	0	0	0	0	0	4	0	4	68	0	0
18	0	0	0	0	1	0	0	0	0	0	0	0	0	0	0	0	0	3	3	67	1	1

Table S6. Confusion matrixes of the calibration set and validation set of KNN model based on FT-MIR full spectra data

Calibration set

Class	1	2	3	4	5	6	7	8	9	10	11	12	13	14	15	16	17	18	TP	TN	FP	FN
1	6	0	0	0	0	0	0	0	0	0	0	0	0	0	0	0	0	0	6	102	0	0
2	0	6	0	0	0	0	0	0	0	0	0	0	0	0	0	0	0	0	6	102	0	0
3	0	0	6	0	0	0	0	0	0	0	0	0	0	0	0	0	0	0	6	102	0	0
4	0	0	0	6	0	0	0	0	0	0	0	0	0	0	0	0	0	0	6	102	0	0
5	0	0	0	0	6	0	0	0	0	0	0	0	0	0	0	0	0	0	6	102	0	0
6	0	0	0	0	0	6	0	0	0	0	0	0	0	0	0	0	0	0	6	102	0	0
7	0	0	0	0	0	0	6	0	0	0	0	0	0	0	0	0	0	0	6	102	0	0
8	0	0	0	0	0	0	0	6	0	0	0	0	0	0	0	0	0	0	6	102	0	0
9	0	0	0	0	0	0	0	0	6	0	0	0	0	0	0	0	0	0	6	102	0	0
10	0	0	0	0	0	0	0	0	0	6	0	0	0	0	0	0	0	0	6	102	0	0
11	0	0	0	0	0	0	0	0	0	0	6	0	0	0	0	0	0	0	6	102	0	0
12	0	0	0	0	0	0	0	0	0	0	0	6	0	0	0	0	0	0	6	102	0	0
13	0	0	0	0	0	0	0	0	0	0	0	0	6	0	0	0	0	0	6	102	0	0
14	0	0	0	0	0	0	0	0	0	0	0	0	0	6	0	0	0	0	6	102	0	0
15	0	0	0	0	0	0	0	0	0	0	0	0	0	0	6	0	0	0	6	102	0	0
16	0	0	0	0	0	0	0	0	0	0	0	0	0	0	0	6	0	0	6	102	0	0
17	0	0	0	0	0	0	0	0	0	0	0	0	0	0	0	0	6	0	6	102	0	0
18	0	0	0	0	0	0	0	0	0	0	0	0	0	0	0	0	0	6	6	102	0	0

Validation set

Class	1	2	3	4	5	6	7	8	9	10	11	12	13	14	15	16	17	18	TP	TN	FP	FN
1	4	0	0	0	0	0	0	0	0	0	0	0	0	0	0	0	0	0	4	68	0	0
2	0	4	0	0	0	0	0	0	0	0	0	0	0	0	0	0	0	0	4	68	0	0
3	0	0	4	0	0	0	0	0	0	0	0	0	0	0	0	0	0	0	4	68	0	0
4	0	0	0	4	0	0	0	0	0	0	0	0	0	0	0	0	0	0	4	67	1	0
5	0	0	0	0	4	0	0	0	0	0	0	0	0	0	0	0	0	0	4	68	0	0
6	0	0	0	0	0	4	0	0	0	0	0	0	0	0	0	0	0	0	4	68	0	0
7	0	0	0	0	0	0	4	0	0	0	0	0	0	0	0	0	0	0	4	68	0	0
8	0	0	0	0	0	0	0	4	0	0	0	0	0	0	0	0	0	0	4	66	2	0
9	0	0	0	0	0	0	0	2	2	0	0	0	0	0	0	0	0	0	2	68	0	2
10	0	0	0	0	0	0	0	0	0	4	0	0	0	0	0	0	0	0	4	68	0	0
11	0	0	0	0	0	0	0	0	0	0	4	0	0	0	0	0	0	0	4	67	1	0
12	0	0	0	0	0	0	0	0	0	0	0	4	0	0	0	0	0	0	4	68	0	0
13	0	0	0	0	0	0	0	0	0	0	0	0	4	0	0	0	0	0	4	68	0	0
14	0	0	0	0	0	0	0	0	0	0	0	0	0	4	0	0	0	0	4	68	0	0
15	0	0	0	0	0	0	0	0	0	0	0	0	0	0	4	0	0	0	4	68	0	0
16	0	0	0	1	0	0	0	0	0	0	1	0	0	0	0	2	0	0	2	68	0	2
17	0	0	0	0	0	0	0	0	0	0	0	0	0	0	0	0	4	0	4	68	0	0
18	0	0	0	0	0	0	0	0	0	0	0	0	0	0	0	0	0	4	4	68	0	0

Table S7. Confusion matrixes of the calibration set and validation set of VIP-NIR-RF

Calibration set																											
**Class**	**1**	**2**	**3**	**4**	**5**	**6**	**7**	**8**	**9**	**10**	**11**	**12**	**13**	**14**	**15**	**16**	**17**	**18**	**TP**	**TN**	**FP**	**FN**	**ACC**	**SE**	**SP**	**MCC**	**EFF**
1	6	0	0	0	0	0	0	0	0	0	0	0	0	0	0	0	0	0	6	102	0	0	100.00	1.00	1.00	1.00	1.00
2	0	6	0	0	0	0	0	0	0	0	0	0	0	0	0	0	0	0	6	102	0	0	100.00	1.00	1.00	1.00	1.00
3	0	0	6	0	0	0	0	0	0	0	0	0	0	0	0	0	0	0	6	102	0	0	100.00	1.00	1.00	1.00	1.00
4	0	0	0	6	0	0	0	0	0	0	0	0	0	0	0	0	0	0	6	102	0	0	100.00	1.00	1.00	1.00	1.00
5	0	0	0	0	6	0	0	0	0	0	0	0	0	0	0	0	0	0	6	102	0	0	100.00	1.00	1.00	1.00	1.00
6	0	0	0	0	0	6	0	0	0	0	0	0	0	0	0	0	0	0	6	102	0	0	100.00	1.00	1.00	1.00	1.00
7	0	0	0	0	0	0	6	0	0	0	0	0	0	0	0	0	0	0	6	102	0	0	100.00	1.00	1.00	1.00	1.00
8	0	0	0	0	0	0	0	6	0	0	0	0	0	0	0	0	0	0	6	102	0	0	100.00	1.00	1.00	1.00	1.00
9	0	0	0	0	0	0	0	0	6	0	0	0	0	0	0	0	0	0	6	102	0	0	100.00	1.00	1.00	1.00	1.00
10	0	0	0	0	0	0	0	0	0	6	0	0	0	0	0	0	0	0	6	102	0	0	100.00	1.00	1.00	1.00	1.00
11	0	0	0	0	0	0	0	0	0	0	6	0	0	0	0	0	0	0	6	102	0	0	100.00	1.00	1.00	1.00	1.00
12	0	0	0	0	0	0	0	0	0	0	0	6	0	0	0	0	0	0	6	102	0	0	100.00	1.00	1.00	1.00	1.00
13	0	0	0	0	0	0	0	0	0	0	0	0	6	0	0	0	0	0	6	102	0	0	100.00	1.00	1.00	1.00	1.00
14	0	0	0	0	0	0	0	0	0	0	0	0	0	6	0	0	0	0	6	102	0	0	100.00	1.00	1.00	1.00	1.00
15	0	0	0	0	0	0	0	0	0	0	0	0	0	0	6	0	0	0	6	102	0	0	100.00	1.00	1.00	1.00	1.00
16	0	0	0	0	0	0	0	0	0	0	0	0	0	0	0	6	0	0	6	102	0	0	100.00	1.00	1.00	1.00	1.00
17	0	0	0	0	0	0	0	0	0	0	0	0	0	0	0	0	6	0	6	102	0	0	100.00	1.00	1.00	1.00	1.00
18	0	0	0	0	0	0	0	0	0	0	0	0	0	0	0	0	0	6	6	102	0	0	100.00	1.00	1.00	1.00	1.00

Validation set																											
**Class**	**1**	**2**	**3**	**4**	**5**	**6**	**7**	**8**	**9**	**10**	**11**	**12**	**13**	**14**	**15**	**16**	**17**	**18**	**TP**	**TN**	**FP**	**FN**	**ACC**	**SE**	**SP**	**MCC**	**EFF**
1	4	0	0	0	0	0	0	0	0	0	0	0	0	0	0	0	0	0	4	68	0	0	100.00	1.00	1.00	1.00	1.00
2	0	4	0	0	0	0	0	0	0	0	0	0	0	0	0	0	0	0	4	68	0	0	100.00	1.00	1.00	1.00	1.00
3	0	0	4	0	0	0	0	0	0	0	0	0	0	0	0	0	0	0	4	68	0	0	100.00	1.00	1.00	1.00	1.00
4	0	0	0	4	0	0	0	0	0	0	0	0	0	0	0	0	0	0	4	68	0	0	100.00	1.00	1.00	1.00	1.00
5	0	0	0	0	3	0	0	0	0	0	0	0	0	0	0	0	0	1	3	67	1	1	97.22	0.75	0.99	0.74	0.86
6	0	0	0	0	0	4	0	0	0	0	0	0	0	0	0	0	0	0	4	68	0	0	100.00	1.00	1.00	1.00	1.00
7	0	0	0	0	0	0	4	0	0	0	0	0	0	0	0	0	0	0	4	68	0	0	100.00	1.00	1.00	1.00	1.00
8	0	0	0	0	0	0	0	4	0	0	0	0	0	0	0	0	0	0	4	68	0	0	100.00	1.00	1.00	1.00	1.00
9	0	0	0	0	0	0	0	0	4	0	0	0	0	0	0	0	0	0	4	68	0	0	100.00	1.00	1.00	1.00	1.00
10	0	0	0	0	0	0	0	0	0	4	0	0	0	0	0	0	0	0	4	68	0	0	100.00	1.00	1.00	1.00	1.00
11	0	0	0	0	0	0	0	0	0	0	4	0	0	0	0	0	0	0	4	68	0	0	100.00	1.00	1.00	1.00	1.00
12	0	0	0	0	0	0	0	0	0	0	0	4	0	0	0	0	0	0	4	68	0	0	100.00	1.00	1.00	1.00	1.00
13	0	0	0	0	0	0	0	0	0	0	0	0	4	0	0	0	0	0	4	68	0	0	100.00	1.00	1.00	1.00	1.00
14	0	0	0	0	0	0	0	0	0	0	0	0	0	4	0	0	0	0	4	68	0	0	100.00	1.00	1.00	1.00	1.00
15	0	0	0	0	0	0	0	0	0	0	0	0	0	0	4	0	0	0	4	68	0	0	100.00	1.00	1.00	1.00	1.00
16	0	0	0	0	0	0	0	0	0	0	0	0	0	0	0	4	0	0	4	68	0	0	100.00	1.00	1.00	1.00	1.00
17	0	0	0	0	0	0	0	0	0	0	0	0	0	0	0	0	4	0	4	68	0	0	100.00	1.00	1.00	1.00	1.00
18	0	0	0	0	1	0	0	0	0	0	0	0	0	0	0	0	0	3	3	67	1	1	97.22	0.75	0.99	0.74	0.86

Table S8. Confusion matrixes of the calibration set and validation set of Bor-NIR-RF

Calibration set																												
**Class**	**1**	**2**	**3**	**4**	**5**	**6**	**7**	**8**	**9**	**10**	**11**	**12**	**13**	**14**	**15**	**16**	**17**	**18**	**TP**	**TN**	**FP**	**FN**	**ACC**	**SE**	**SP**	**MCC**	**EFF**
1	6	0	0	0	0	0	0	0	0	0	0	0	0	0	0	0	0	0	6	102	0	0	100.00	1.00	1.00	1.00	1.00
2	0	6	0	0	0	0	0	0	0	0	0	0	0	0	0	0	0	0	6	102	0	0	100.00	1.00	1.00	1.00	1.00
3	0	0	6	0	0	0	0	0	0	0	0	0	0	0	0	0	0	0	6	102	0	0	100.00	1.00	1.00	1.00	1.00
4	0	0	0	6	0	0	0	0	0	0	0	0	0	0	0	0	0	0	6	102	0	0	100.00	1.00	1.00	1.00	1.00
5	0	0	0	0	6	0	0	0	0	0	0	0	0	0	0	0	0	0	6	102	0	0	100.00	1.00	1.00	1.00	1.00
6	0	0	0	0	0	6	0	0	0	0	0	0	0	0	0	0	0	0	6	102	0	0	100.00	1.00	1.00	1.00	1.00
7	0	0	0	0	0	0	6	0	0	0	0	0	0	0	0	0	0	0	6	102	0	0	100.00	1.00	1.00	1.00	1.00
8	0	0	0	0	0	0	0	6	0	0	0	0	0	0	0	0	0	0	6	102	0	0	100.00	1.00	1.00	1.00	1.00
9	0	0	0	0	0	0	0	0	6	0	0	0	0	0	0	0	0	0	6	102	0	0	100.00	1.00	1.00	1.00	1.00
10	0	0	0	0	0	0	0	0	0	6	0	0	0	0	0	0	0	0	6	102	0	0	100.00	1.00	1.00	1.00	1.00
11	0	0	0	0	0	0	0	0	0	0	6	0	0	0	0	0	0	0	6	102	0	0	100.00	1.00	1.00	1.00	1.00
12	0	0	0	0	0	0	0	0	0	0	0	6	0	0	0	0	0	0	6	102	0	0	100.00	1.00	1.00	1.00	1.00
13	0	0	0	0	0	0	0	0	0	0	0	0	6	0	0	0	0	0	6	102	0	0	100.00	1.00	1.00	1.00	1.00
14	0	0	0	0	0	0	0	0	0	0	0	0	0	6	0	0	0	0	6	102	0	0	100.00	1.00	1.00	1.00	1.00
15	0	0	0	0	0	0	0	0	0	0	0	0	0	0	6	0	0	0	6	102	0	0	100.00	1.00	1.00	1.00	1.00
16	0	0	0	0	0	0	0	0	0	0	0	0	0	0	0	6	0	0	6	102	0	0	100.00	1.00	1.00	1.00	1.00
17	0	0	0	0	0	0	0	0	0	0	0	0	0	0	0	0	6	0	6	102	0	0	100.00	1.00	1.00	1.00	1.00
18	0	0	0	0	0	0	0	0	0	0	0	0	0	0	0	0	0	6	6	102	0	0	100.00	1.00	1.00	1.00	1.00

Validation set																											
**Class**	**1**	**2**	**3**	**4**	**5**	**6**	**7**	**8**	**9**	**10**	**11**	**12**	**13**	**14**	**15**	**16**	**17**	**18**	**TP**	**TN**	**FP**	**FN**	**ACC**	**SE**	**SP**	**MCC**	**EFF**
1	4	0	0	0	0	0	0	0	0	0	0	0	0	0	0	0	0	0	4	68	0	0	100.00	1.00	1.00	1.00	1.00
2	0	3	0	0	0	0	0	0	0	1	0	0	0	0	0	0	0	0	3	68	0	1	98.61	0.75	1.00	0.86	0.87
3	0	0	4	0	0	0	0	0	0	0	0	0	0	0	0	0	0	0	4	68	0	0	100.00	1.00	1.00	1.00	1.00
4	0	0	0	4	0	0	0	0	0	0	0	0	0	0	0	0	0	0	4	68	0	0	100.00	1.00	1.00	1.00	1.00
5	0	0	0	0	3	0	0	0	0	0	0	0	0	0	0	0	0	1	3	67	1	1	97.22	0.75	0.99	0.74	0.86
6	0	0	0	0	0	4	0	0	0	0	0	0	0	0	0	0	0	0	4	68	0	0	100.00	1.00	1.00	1.00	1.00
7	0	0	0	0	0	0	2	0	2	0	0	0	0	0	0	0	0	0	2	68	0	2	97.22	0.50	1.00	0.70	0.71
8	0	0	0	0	0	0	0	4	0	0	0	0	0	0	0	0	0	0	4	68	0	0	100.00	1.00	1.00	1.00	1.00
9	0	0	0	0	0	0	0	0	4	0	0	0	0	0	0	0	0	0	4	66	2	0	97.22	1.00	0.97	0.80	0.99
10	0	0	0	0	0	0	0	0	0	4	0	0	0	0	0	0	0	0	4	67	1	0	98.61	1.00	0.99	0.89	0.99
11	0	0	0	0	0	0	0	0	0	0	4	0	0	0	0	0	0	0	4	68	0	0	100.00	1.00	1.00	1.00	1.00
12	0	0	0	0	0	0	0	0	0	0	0	4	0	0	0	0	0	0	4	67	1	0	98.61	1.00	0.99	0.89	0.99
13	0	0	0	0	0	0	0	0	0	0	0	0	4	0	0	0	0	0	4	68	0	0	100.00	1.00	1.00	1.00	1.00
14	0	0	0	0	0	0	0	0	0	0	0	0	0	4	0	0	0	0	4	68	0	0	100.00	1.00	1.00	1.00	1.00
15	0	0	0	0	0	0	0	0	0	0	0	0	0	0	4	0	0	0	4	68	0	0	100.00	1.00	1.00	1.00	1.00
16	0	0	0	0	0	0	0	0	0	0	0	1	0	0	0	3	0	0	3	68	0	1	98.61	0.75	1.00	0.86	0.87
17	0	0	0	0	0	0	0	0	0	0	0	0	0	0	0	0	4	0	4	68	0	0	100.00	1.00	1.00	1.00	1.00
18	0	0	0	0	1	0	0	0	0	0	0	0	0	0	0	0	0	3	3	67	1	1	97.22	0.75	0.99	0.74	0.86

Table S9. Confusion matrixes of the calibration set and validation set of GARF-NIR-RF

Calibration set																											
**Class**	**1**	**2**	**3**	**4**	**5**	**6**	**7**	**8**	**9**	**10**	**11**	**12**	**13**	**14**	**15**	**16**	**17**	**18**	**TP**	**TN**	**FP**	**FN**	**ACC**	**SE**	**SP**	**MCC**	**EFF**
1	6	0	0	0	0	0	0	0	0	0	0	0	0	0	0	0	0	0	6	102	0	0	100.00	1.00	1.00	1.00	1.00
2	0	6	0	0	0	0	0	0	0	0	0	0	0	0	0	0	0	0	6	102	0	0	100.00	1.00	1.00	1.00	1.00
3	0	0	6	0	0	0	0	0	0	0	0	0	0	0	0	0	0	0	6	102	0	0	100.00	1.00	1.00	1.00	1.00
4	0	0	0	6	0	0	0	0	0	0	0	0	0	0	0	0	0	0	6	102	0	0	100.00	1.00	1.00	1.00	1.00
5	0	0	0	0	6	0	0	0	0	0	0	0	0	0	0	0	0	0	6	102	0	0	100.00	1.00	1.00	1.00	1.00
6	0	0	0	0	0	6	0	0	0	0	0	0	0	0	0	0	0	0	6	102	0	0	100.00	1.00	1.00	1.00	1.00
7	0	0	0	0	0	0	6	0	0	0	0	0	0	0	0	0	0	0	6	102	0	0	100.00	1.00	1.00	1.00	1.00
8	0	0	0	0	0	0	0	6	0	0	0	0	0	0	0	0	0	0	6	102	0	0	100.00	1.00	1.00	1.00	1.00
9	0	0	0	0	0	0	0	0	6	0	0	0	0	0	0	0	0	0	6	102	0	0	100.00	1.00	1.00	1.00	1.00
10	0	0	0	0	0	0	0	0	0	6	0	0	0	0	0	0	0	0	6	102	0	0	100.00	1.00	1.00	1.00	1.00
11	0	0	0	0	0	0	0	0	0	0	6	0	0	0	0	0	0	0	6	102	0	0	100.00	1.00	1.00	1.00	1.00
12	0	0	0	0	0	0	0	0	0	0	0	6	0	0	0	0	0	0	6	102	0	0	100.00	1.00	1.00	1.00	1.00
13	0	0	0	0	0	0	0	0	0	0	0	0	6	0	0	0	0	0	6	102	0	0	100.00	1.00	1.00	1.00	1.00
14	0	0	0	0	0	0	0	0	0	0	0	0	0	6	0	0	0	0	6	102	0	0	100.00	1.00	1.00	1.00	1.00
15	0	0	0	0	0	0	0	0	0	0	0	0	0	0	6	0	0	0	6	102	0	0	100.00	1.00	1.00	1.00	1.00
16	0	0	0	0	0	0	0	0	0	0	0	0	0	0	0	6	0	0	6	102	0	0	100.00	1.00	1.00	1.00	1.00
17	0	0	0	0	0	0	0	0	0	0	0	0	0	0	0	0	6	0	6	102	0	0	100.00	1.00	1.00	1.00	1.00
18	0	0	0	0	0	0	0	0	0	0	0	0	0	0	0	0	0	6	6	102	0	0	100.00	1.00	1.00	1.00	1.00

Validation set

Class	1	2	3	4	5	6	7	8	9	10	11	12	13	14	15	16	17	18	TP	TN	FP	FN	ACC	SE	SP	MCC	EFF
1	4	0	0	0	0	0	0	0	0	0	0	0	0	0	0	0	0	0	4	68	0	0	100.00	1.00	1.00	1.00	1.00
2	0	4	0	0	0	0	0	0	0	0	0	0	0	0	0	0	0	0	4	68	0	0	100.00	1.00	1.00	1.00	1.00
3	0	0	4	0	0	0	0	0	0	0	0	0	0	0	0	0	0	0	4	68	0	0	100.00	1.00	1.00	1.00	1.00
4	0	0	0	4	0	0	0	0	0	0	0	0	0	0	0	0	0	0	4	67	1	0	98.61	1.00	0.99	0.89	0.99
5	0	0	0	0	3	0	0	0	0	0	0	0	0	0	0	0	0	1	3	67	1	1	97.22	0.75	0.99	0.74	0.86
6	0	0	0	0	0	4	0	0	0	0	0	0	0	0	0	0	0	0	4	68	0	0	100.00	1.00	1.00	1.00	1.00
7	0	0	0	0	0	0	2	0	2	0	0	0	0	0	0	0	0	0	2	68	0	2	97.22	0.50	1.00	0.70	0.71
8	0	0	0	0	0	0	0	4	0	0	0	0	0	0	0	0	0	0	4	68	0	0	100.00	1.00	1.00	1.00	1.00
9	0	0	0	0	0	0	0	0	4	0	0	0	0	0	0	0	0	0	4	66	2	0	97.22	1.00	0.97	0.80	0.99
10	0	0	0	0	0	0	0	0	0	4	0	0	0	0	0	0	0	0	4	68	0	0	100.00	1.00	1.00	1.00	1.00
11	0	0	0	0	0	0	0	0	0	0	4	0	0	0	0	0	0	0	4	68	0	0	100.00	1.00	1.00	1.00	1.00
12	0	0	0	1	0	0	0	0	0	0	0	3	0	0	0	0	0	0	3	67	1	1	97.22	0.75	0.99	0.74	0.86
13	0	0	0	0	0	0	0	0	0	0	0	0	4	0	0	0	0	0	4	68	0	0	100.00	1.00	1.00	1.00	1.00
14	0	0	0	0	0	0	0	0	0	0	0	0	0	4	0	0	0	0	4	68	0	0	100.00	1.00	1.00	1.00	1.00
15	0	0	0	0	0	0	0	0	0	0	0	0	0	0	4	0	0	0	4	68	0	0	100.00	1.00	1.00	1.00	1.00
16	0	0	0	0	0	0	0	0	0	0	0	1	0	0	0	3	0	0	3	68	0	1	98.61	0.75	1.00	0.86	0.87
17	0	0	0	0	0	0	0	0	0	0	0	0	0	0	0	0	4	0	4	68	0	0	100.00	1.00	1.00	1.00	1.00
18	0	0	0	0	1	0	0	0	0	0	0	0	0	0	0	0	0	3	3	67	1	1	97.22	0.75	0.99	0.74	0.86

Table S10. Confusion matrixes of the calibration set and validation set of GASVM-NIR-RF

Calibration set																												
**Class**	**1**	**2**	**3**	**4**	**5**	**6**	**7**	**8**	**9**	**10**	**11**	**12**	**13**	**14**	**15**	**16**	**17**	**18**	**TP**	**TN**	**FP**	**FN**	**ACC**	**SE**	**SP**	**MCC**	**EFF**
1	6	0	0	0	0	0	0	0	0	0	0	0	0	0	0	0	0	0	6	102	0	0	100.00	1.00	1.00	1.00	1.00
2	0	6	0	0	0	0	0	0	0	0	0	0	0	0	0	0	0	0	6	102	0	0	100.00	1.00	1.00	1.00	1.00
3	0	0	6	0	0	0	0	0	0	0	0	0	0	0	0	0	0	0	6	102	0	0	100.00	1.00	1.00	1.00	1.00
4	0	0	0	6	0	0	0	0	0	0	0	0	0	0	0	0	0	0	6	102	0	0	100.00	1.00	1.00	1.00	1.00
5	0	0	0	0	6	0	0	0	0	0	0	0	0	0	0	0	0	0	6	102	0	0	100.00	1.00	1.00	1.00	1.00
6	0	0	0	0	0	6	0	0	0	0	0	0	0	0	0	0	0	0	6	102	0	0	100.00	1.00	1.00	1.00	1.00
7	0	0	0	0	0	0	6	0	0	0	0	0	0	0	0	0	0	0	6	102	0	0	100.00	1.00	1.00	1.00	1.00
8	0	0	0	0	0	0	0	6	0	0	0	0	0	0	0	0	0	0	6	102	0	0	100.00	1.00	1.00	1.00	1.00
9	0	0	0	0	0	0	0	0	6	0	0	0	0	0	0	0	0	0	6	102	0	0	100.00	1.00	1.00	1.00	1.00
10	0	0	0	0	0	0	0	0	0	6	0	0	0	0	0	0	0	0	6	102	0	0	100.00	1.00	1.00	1.00	1.00
11	0	0	0	0	0	0	0	0	0	0	6	0	0	0	0	0	0	0	6	102	0	0	100.00	1.00	1.00	1.00	1.00
12	0	0	0	0	0	0	0	0	0	0	0	6	0	0	0	0	0	0	6	102	0	0	100.00	1.00	1.00	1.00	1.00
13	0	0	0	0	0	0	0	0	0	0	0	0	6	0	0	0	0	0	6	102	0	0	100.00	1.00	1.00	1.00	1.00
14	0	0	0	0	0	0	0	0	0	0	0	0	0	6	0	0	0	0	6	102	0	0	100.00	1.00	1.00	1.00	1.00
15	0	0	0	0	0	0	0	0	0	0	0	0	0	0	6	0	0	0	6	102	0	0	100.00	1.00	1.00	1.00	1.00
16	0	0	0	0	0	0	0	0	0	0	0	0	0	0	0	6	0	0	6	102	0	0	100.00	1.00	1.00	1.00	1.00
17	0	0	0	0	0	0	0	0	0	0	0	0	0	0	0	0	6	0	6	102	0	0	100.00	1.00	1.00	1.00	1.00
18	0	0	0	0	0	0	0	0	0	0	0	0	0	0	0	0	0	6	6	102	0	0	100.00	1.00	1.00	1.00	1.00

	Validation set																										
**Class**	**1**	**2**	**3**	**4**	**5**	**6**	**7**	**8**	**9**	**10**	**11**	**12**	**13**	**14**	**15**	**16**	**17**	**18**	**TP**	**TN**	**FP**	**FN**	**ACC**	**SE**	**SP**	**MCC**	**EFF**
1	4	0	0	0	0	0	0	0	0	0	0	0	0	0	0	0	0	0	4	68	0	0	100.00	1.00	1.00	1.00	1.00
2	0	4	0	0	0	0	0	0	0	0	0	0	0	0	0	0	0	0	4	68	0	0	100.00	1.00	1.00	1.00	1.00
3	0	0	4	0	0	0	0	0	0	0	0	0	0	0	0	0	0	0	4	68	0	0	100.00	1.00	1.00	1.00	1.00
4	0	0	0	4	0	0	0	0	0	0	0	0	0	0	0	0	0	0	4	67	1	0	98.61	1.00	0.99	0.89	0.99
5	0	0	0	0	3	0	0	0	0	0	0	0	0	0	0	0	0	1	3	67	1	1	97.22	0.75	0.99	0.74	0.86
6	0	0	0	0	0	4	0	0	0	0	0	0	0	0	0	0	0	0	4	68	0	0	100.00	1.00	1.00	1.00	1.00
7	0	0	0	0	0	0	3	0	1	0	0	0	0	0	0	0	0	0	3	68	0	1	98.61	0.75	1.00	0.86	0.87
8	0	0	0	0	0	0	0	3	1	0	0	0	0	0	0	0	0	0	3	68	0	1	98.61	0.75	1.00	0.86	0.87
9	0	0	0	0	0	0	0	0	4	0	0	0	0	0	0	0	0	0	4	66	2	0	97.22	1.00	0.97	0.80	0.99
10	0	0	0	0	0	0	0	0	0	4	0	0	0	0	0	0	0	0	4	68	0	0	100.00	1.00	1.00	1.00	1.00
11	0	0	0	0	0	0	0	0	0	0	4	0	0	0	0	0	0	0	4	68	0	0	100.00	1.00	1.00	1.00	1.00
12	0	0	0	1	0	0	0	0	0	0	0	3	0	0	0	0	0	0	3	67	1	1	97.22	0.75	0.99	0.74	0.86
13	0	0	0	0	0	0	0	0	0	0	0	0	4	0	0	0	0	0	4	68	0	0	100.00	1.00	1.00	1.00	1.00
14	0	0	0	0	0	0	0	0	0	0	0	0	0	4	0	0	0	0	4	68	0	0	100.00	1.00	1.00	1.00	1.00
15	0	0	0	0	0	0	0	0	0	0	0	0	0	0	4	0	0	0	4	68	0	0	100.00	1.00	1.00	1.00	1.00
16	0	0	0	0	0	0	0	0	0	0	0	1	0	0	0	3	0	0	3	68	0	1	98.61	0.75	1.00	0.86	0.87
17	0	0	0	0	0	0	0	0	0	0	0	0	0	0	0	0	4	0	4	68	0	0	100.00	1.00	1.00	1.00	1.00
18	0	0	0	0	1	0	0	0	0	0	0	0	0	0	0	0	0	3	3	67	1	1	97.22	0.75	0.99	0.74	0.86

Table S11. Confusion matrixes of the calibration set and validation set of Ven-NIR-RF

Calibration set																											
**Class**	**1**	**2**	**3**	**4**	**5**	**6**	**7**	**8**	**9**	**10**	**11**	**12**	**13**	**14**	**15**	**16**	**17**	**18**	**TP**	**TN**	**FP**	**FN**	**ACC**	**SE**	**SP**	**MCC**	**EFF**
1	6	0	0	0	0	0	0	0	0	0	0	0	0	0	0	0	0	0	6	102	0	0	100.00	1.00	1.00	1.00	1.00
2	0	6	0	0	0	0	0	0	0	0	0	0	0	0	0	0	0	0	6	102	0	0	100.00	1.00	1.00	1.00	1.00
3	0	0	6	0	0	0	0	0	0	0	0	0	0	0	0	0	0	0	6	102	0	0	100.00	1.00	1.00	1.00	1.00
4	0	0	0	6	0	0	0	0	0	0	0	0	0	0	0	0	0	0	6	102	0	0	100.00	1.00	1.00	1.00	1.00
5	0	0	0	0	6	0	0	0	0	0	0	0	0	0	0	0	0	0	6	102	0	0	100.00	1.00	1.00	1.00	1.00
6	0	0	0	0	0	6	0	0	0	0	0	0	0	0	0	0	0	0	6	102	0	0	100.00	1.00	1.00	1.00	1.00
7	0	0	0	0	0	0	6	0	0	0	0	0	0	0	0	0	0	0	6	102	0	0	100.00	1.00	1.00	1.00	1.00
8	0	0	0	0	0	0	0	6	0	0	0	0	0	0	0	0	0	0	6	102	0	0	100.00	1.00	1.00	1.00	1.00
9	0	0	0	0	0	0	0	0	6	0	0	0	0	0	0	0	0	0	6	102	0	0	100.00	1.00	1.00	1.00	1.00
10	0	0	0	0	0	0	0	0	0	6	0	0	0	0	0	0	0	0	6	102	0	0	100.00	1.00	1.00	1.00	1.00
11	0	0	0	0	0	0	0	0	0	0	6	0	0	0	0	0	0	0	6	102	0	0	100.00	1.00	1.00	1.00	1.00
12	0	0	0	0	0	0	0	0	0	0	0	6	0	0	0	0	0	0	6	102	0	0	100.00	1.00	1.00	1.00	1.00
13	0	0	0	0	0	0	0	0	0	0	0	0	6	0	0	0	0	0	6	102	0	0	100.00	1.00	1.00	1.00	1.00
14	0	0	0	0	0	0	0	0	0	0	0	0	0	6	0	0	0	0	6	102	0	0	100.00	1.00	1.00	1.00	1.00
15	0	0	0	0	0	0	0	0	0	0	0	0	0	0	6	0	0	0	6	102	0	0	100.00	1.00	1.00	1.00	1.00
16	0	0	0	0	0	0	0	0	0	0	0	0	0	0	0	6	0	0	6	102	0	0	100.00	1.00	1.00	1.00	1.00
17	0	0	0	0	0	0	0	0	0	0	0	0	0	0	0	0	6	0	6	102	0	0	100.00	1.00	1.00	1.00	1.00
18	0	0	0	0	0	0	0	0	0	0	0	0	0	0	0	0	0	6	6	102	0	0	100.00	1.00	1.00	1.00	1.00

Validation set																												
**Class**	**1**	**2**	**3**	**4**	**5**	**6**	**7**	**8**	**9**	**10**	**11**	**12**	**13**	**14**	**15**	**16**	**17**	**18**	**TP**	**TN**	**FP**	**FN**	**ACC**	**SE**	**SP**	**MCC**	**EFF**
1	4	0	0	0	0	0	0	0	0	0	0	0	0	0	0	0	0	0	4	68	0	0	100.00	1.00	1.00	1.00	1.00
2	0	4	0	0	0	0	0	0	0	0	0	0	0	0	0	0	0	0	4	68	0	0	100.00	1.00	1.00	1.00	1.00
3	0	0	4	0	0	0	0	0	0	0	0	0	0	0	0	0	0	0	4	68	0	0	100.00	1.00	1.00	1.00	1.00
4	0	0	0	4	0	0	0	0	0	0	0	0	0	0	0	0	0	0	4	68	0	0	100.00	1.00	1.00	1.00	1.00
5	0	0	0	0	3	0	0	0	0	0	0	0	0	0	0	0	0	1	3	67	1	1	97.22	0.75	0.99	0.74	0.86
6	0	0	0	0	0	4	0	0	0	0	0	0	0	0	0	0	0	0	4	68	0	0	100.00	1.00	1.00	1.00	1.00
7	0	0	0	0	0	0	3	0	1	0	0	0	0	0	0	0	0	0	3	68	0	1	98.61	0.75	1.00	0.86	0.87
8	0	0	0	0	0	0	0	4	0	0	0	0	0	0	0	0	0	0	4	68	0	0	100.00	1.00	1.00	1.00	1.00
9	0	0	0	0	0	0	0	0	4	0	0	0	0	0	0	0	0	0	4	67	1	0	98.61	1.00	0.99	0.89	0.99
10	0	0	0	0	0	0	0	0	0	4	0	0	0	0	0	0	0	0	4	68	0	0	100.00	1.00	1.00	1.00	1.00
11	0	0	0	0	0	0	0	0	0	0	4	0	0	0	0	0	0	0	4	68	0	0	100.00	1.00	1.00	1.00	1.00
12	0	0	0	0	0	0	0	0	0	0	0	4	0	0	0	0	0	0	4	67	1	0	98.61	1.00	0.99	0.89	0.99
13	0	0	0	0	0	0	0	0	0	0	0	0	4	0	0	0	0	0	4	68	0	0	100.00	1.00	1.00	1.00	1.00
14	0	0	0	0	0	0	0	0	0	0	0	0	0	4	0	0	0	0	4	68	0	0	100.00	1.00	1.00	1.00	1.00
15	0	0	0	0	0	0	0	0	0	0	0	0	0	0	4	0	0	0	4	68	0	0	100.00	1.00	1.00	1.00	1.00
16	0	0	0	0	0	0	0	0	0	0	0	1	0	0	0	3	0	0	3	68	0	1	98.61	0.75	1.00	0.86	0.87
17	0	0	0	0	0	0	0	0	0	0	0	0	0	0	0	0	4	0	4	68	0	0	100.00	1.00	1.00	1.00	1.00
18	0	0	0	0	1	0	0	0	0	0	0	0	0	0	0	0	0	3	3	67	1	1	97.22	0.75	0.99	0.74	0.86

Table S12. Confusion matrixes of the calibration set and validation set of VIP-NIR-SVM

**Calibration set**																											
**Class**	**1**	**2**	**3**	**4**	**5**	**6**	**7**	**8**	**9**	**10**	**11**	**12**	**13**	**14**	**15**	**16**	**17**	**18**	**TP**	**TN**	**FP**	**FN**	**ACC**	**SE**	**SP**	**MCC**	**EFF**
1	6	0	0	0	0	0	0	0	0	0	0	0	0	0	0	0	0	0	6	102	0	0	100.00	1.00	1.00	1.00	1.00
2	0	6	0	0	0	0	0	0	0	0	0	0	0	0	0	0	0	0	6	102	0	0	100.00	1.00	1.00	1.00	1.00
3	0	0	6	0	0	0	0	0	0	0	0	0	0	0	0	0	0	0	6	102	0	0	100.00	1.00	1.00	1.00	1.00
4	0	0	0	6	0	0	0	0	0	0	0	0	0	0	0	0	0	0	6	102	0	0	100.00	1.00	1.00	1.00	1.00
5	0	0	0	0	6	0	0	0	0	0	0	0	0	0	0	0	0	0	6	102	0	0	100.00	1.00	1.00	1.00	1.00
6	0	0	0	0	0	6	0	0	0	0	0	0	0	0	0	0	0	0	6	102	0	0	100.00	1.00	1.00	1.00	1.00
7	0	0	0	0	0	0	6	0	0	0	0	0	0	0	0	0	0	0	6	102	0	0	100.00	1.00	1.00	1.00	1.00
8	0	0	0	0	0	0	0	6	0	0	0	0	0	0	0	0	0	0	6	102	0	0	100.00	1.00	1.00	1.00	1.00
9	0	0	0	0	0	0	0	0	6	0	0	0	0	0	0	0	0	0	6	102	0	0	100.00	1.00	1.00	1.00	1.00
10	0	0	0	0	0	0	0	0	0	6	0	0	0	0	0	0	0	0	6	102	0	0	100.00	1.00	1.00	1.00	1.00
11	0	0	0	0	0	0	0	0	0	0	6	0	0	0	0	0	0	0	6	102	0	0	100.00	1.00	1.00	1.00	1.00
12	0	0	0	0	0	0	0	0	0	0	0	6	0	0	0	0	0	0	6	102	0	0	100.00	1.00	1.00	1.00	1.00
13	0	0	0	0	0	0	0	0	0	0	0	0	6	0	0	0	0	0	6	102	0	0	100.00	1.00	1.00	1.00	1.00
14	0	0	0	0	0	0	0	0	0	0	0	0	0	6	0	0	0	0	6	102	0	0	100.00	1.00	1.00	1.00	1.00
15	0	0	0	0	0	0	0	0	0	0	0	0	0	0	6	0	0	0	6	102	0	0	100.00	1.00	1.00	1.00	1.00
16	0	0	0	0	0	0	0	0	0	0	0	0	0	0	0	6	0	0	6	102	0	0	100.00	1.00	1.00	1.00	1.00
17	0	0	0	0	0	0	0	0	0	0	0	0	0	0	0	0	6	0	6	102	0	0	100.00	1.00	1.00	1.00	1.00
18	0	0	0	0	0	0	0	0	0	0	0	0	0	0	0	0	0	6	6	102	0	0	100.00	1.00	1.00	1.00	1.00

Validation set																												
**Class**	**1**	**2**	**3**	**4**	**5**	**6**	**7**	**8**	**9**	**10**	**11**	**12**	**13**	**14**	**15**	**16**	**17**	**18**	**TP**	**TN**	**FP**	**FN**	**ACC**	**SE**	**SP**	**MCC**	**EFF**
1	4	0	0	0	0	0	0	0	0	0	0	0	0	0	0	0	0	0	4	68	0	0	100.00	1.00	1.00	1.00	1.00
2	0	4	0	0	0	0	0	0	0	0	0	0	0	0	0	0	0	0	4	68	0	0	100.00	1.00	1.00	1.00	1.00
3	0	0	4	0	0	0	0	0	0	0	0	0	0	0	0	0	0	0	4	68	0	0	100.00	1.00	1.00	1.00	1.00
4	0	0	0	4	0	0	0	0	0	0	0	0	0	0	0	0	0	0	4	68	0	0	100.00	1.00	1.00	1.00	1.00
5	0	0	0	0	3	0	0	0	0	0	0	0	0	0	0	0	0	1	3	67	1	1	97.22	0.75	0.99	0.74	0.86
6	0	0	0	0	0	4	0	0	0	0	0	0	0	0	0	0	0	0	4	68	0	0	100.00	1.00	1.00	1.00	1.00
7	0	0	0	0	0	0	4	0	0	0	0	0	0	0	0	0	0	0	4	68	0	0	100.00	1.00	1.00	1.00	1.00
8	0	0	0	0	0	0	0	4	0	0	0	0	0	0	0	0	0	0	4	68	0	0	100.00	1.00	1.00	1.00	1.00
9	0	0	0	0	0	0	0	0	4	0	0	0	0	0	0	0	0	0	4	68	0	0	100.00	1.00	1.00	1.00	1.00
10	0	0	0	0	0	0	0	0	0	4	0	0	0	0	0	0	0	0	4	68	0	0	100.00	1.00	1.00	1.00	1.00
11	0	0	0	0	0	0	0	0	0	0	4	0	0	0	0	0	0	0	4	68	0	0	100.00	1.00	1.00	1.00	1.00
12	0	0	0	0	0	0	0	0	0	0	0	4	0	0	0	0	0	0	4	68	0	0	100.00	1.00	1.00	1.00	1.00
13	0	0	0	0	0	0	0	0	0	0	0	0	4	0	0	0	0	0	4	68	0	0	100.00	1.00	1.00	1.00	1.00
14	0	0	0	0	0	0	0	0	0	0	0	0	0	4	0	0	0	0	4	68	0	0	100.00	1.00	1.00	1.00	1.00
15	0	0	0	0	0	0	0	0	0	0	0	0	0	0	4	0	0	0	4	68	0	0	100.00	1.00	1.00	1.00	1.00
16	0	0	0	0	0	0	0	0	0	0	0	0	0	0	0	4	0	0	4	68	0	0	100.00	1.00	1.00	1.00	1.00
17	0	0	0	0	0	0	0	0	0	0	0	0	0	0	0	0	4	0	4	68	0	0	100.00	1.00	1.00	1.00	1.00
18	0	0	0	0	1	0	0	0	0	0	0	0	0	0	0	0	0	3	3	67	1	1	97.22	0.75	0.99	0.74	0.86

Table S13. Confusion matrixes of the calibration set and validation set of Bor-NIR-SVM

Calibration set																											
**Class**	**1**	**2**	**3**	**4**	**5**	**6**	**7**	**8**	**9**	**10**	**11**	**12**	**13**	**14**	**15**	**16**	**17**	**18**	**TP**	**TN**	**FP**	**FN**	**ACC**	**SE**	**SP**	**MCC**	**EFF**
1	6	0	0	0	0	0	0	0	0	0	0	0	0	0	0	0	0	0	6	102	0	0	100.00	1.00	1.00	1.00	1.00
2	0	6	0	0	0	0	0	0	0	0	0	0	0	0	0	0	0	0	6	102	0	0	100.00	1.00	1.00	1.00	1.00
3	0	0	6	0	0	0	0	0	0	0	0	0	0	0	0	0	0	0	6	102	0	0	100.00	1.00	1.00	1.00	1.00
4	0	0	0	6	0	0	0	0	0	0	0	0	0	0	0	0	0	0	6	102	0	0	100.00	1.00	1.00	1.00	1.00
5	0	0	0	0	6	0	0	0	0	0	0	0	0	0	0	0	0	0	6	102	0	0	100.00	1.00	1.00	1.00	1.00
6	0	0	0	0	0	6	0	0	0	0	0	0	0	0	0	0	0	0	6	102	0	0	100.00	1.00	1.00	1.00	1.00
7	0	0	0	0	0	0	6	0	0	0	0	0	0	0	0	0	0	0	6	102	0	0	100.00	1.00	1.00	1.00	1.00
8	0	0	0	0	0	0	0	6	0	0	0	0	0	0	0	0	0	0	6	102	0	0	100.00	1.00	1.00	1.00	1.00
9	0	0	0	0	0	0	0	0	6	0	0	0	0	0	0	0	0	0	6	102	0	0	100.00	1.00	1.00	1.00	1.00
10	0	0	0	0	0	0	0	0	0	6	0	0	0	0	0	0	0	0	6	102	0	0	100.00	1.00	1.00	1.00	1.00
11	0	0	0	0	0	0	0	0	0	0	6	0	0	0	0	0	0	0	6	102	0	0	100.00	1.00	1.00	1.00	1.00
12	0	0	0	0	0	0	0	0	0	0	0	6	0	0	0	0	0	0	6	102	0	0	100.00	1.00	1.00	1.00	1.00
13	0	0	0	0	0	0	0	0	0	0	0	0	6	0	0	0	0	0	6	102	0	0	100.00	1.00	1.00	1.00	1.00
14	0	0	0	0	0	0	0	0	0	0	0	0	0	6	0	0	0	0	6	102	0	0	100.00	1.00	1.00	1.00	1.00
15	0	0	0	0	0	0	0	0	0	0	0	0	0	0	6	0	0	0	6	102	0	0	100.00	1.00	1.00	1.00	1.00
16	0	0	0	0	0	0	0	0	0	0	0	0	0	0	0	6	0	0	6	102	0	0	100.00	1.00	1.00	1.00	1.00
17	0	0	0	0	0	0	0	0	0	0	0	0	0	0	0	0	6	0	6	102	0	0	100.00	1.00	1.00	1.00	1.00
18	0	0	0	0	0	0	0	0	0	0	0	0	0	0	0	0	0	6	6	102	0	0	100.00	1.00	1.00	1.00	1.00

Validation set																												
**Class**	**1**	**2**	**3**	**4**	**5**	**6**	**7**	**8**	**9**	**10**	**11**	**12**	**13**	**14**	**15**	**16**	**17**	**18**	**TP**	**TN**	**FP**	**FN**	**ACC**	**SE**	**SP**	**MCC**	**EFF**
1	4	0	0	0	0	0	0	0	0	0	0	0	0	0	0	0	0	0	4	68	0	0	100.00	1.00	1.00	1.00	1.00
2	0	4	0	0	0	0	0	0	0	0	0	0	0	0	0	0	0	0	4	68	0	0	100.00	1.00	1.00	1.00	1.00
3	0	0	4	0	0	0	0	0	0	0	0	0	0	0	0	0	0	0	4	68	0	0	100.00	1.00	1.00	1.00	1.00
4	0	0	0	4	0	0	0	0	0	0	0	0	0	0	0	0	0	0	4	68	0	0	100.00	1.00	1.00	1.00	1.00
5	0	0	0	0	4	0	0	0	0	0	0	0	0	0	0	0	0	0	4	67	1	0	98.61	1.00	0.99	0.89	0.99
6	0	0	0	0	0	4	0	0	0	0	0	0	0	0	0	0	0	0	4	68	0	0	100.00	1.00	1.00	1.00	1.00
7	0	0	0	0	0	0	4	0	0	0	0	0	0	0	0	0	0	0	4	68	0	0	100.00	1.00	1.00	1.00	1.00
8	0	0	0	0	0	0	0	4	0	0	0	0	0	0	0	0	0	0	4	68	0	0	100.00	1.00	1.00	1.00	1.00
9	0	0	0	0	0	0	0	0	4	0	0	0	0	0	0	0	0	0	4	68	0	0	100.00	1.00	1.00	1.00	1.00
10	0	0	0	0	0	0	0	0	0	4	0	0	0	0	0	0	0	0	4	68	0	0	100.00	1.00	1.00	1.00	1.00
11	0	0	0	0	0	0	0	0	0	0	4	0	0	0	0	0	0	0	4	68	0	0	100.00	1.00	1.00	1.00	1.00
12	0	0	0	0	0	0	0	0	0	0	0	4	0	0	0	0	0	0	4	68	0	0	100.00	1.00	1.00	1.00	1.00
13	0	0	0	0	0	0	0	0	0	0	0	0	4	0	0	0	0	0	4	68	0	0	100.00	1.00	1.00	1.00	1.00
14	0	0	0	0	0	0	0	0	0	0	0	0	0	4	0	0	0	0	4	68	0	0	100.00	1.00	1.00	1.00	1.00
15	0	0	0	0	0	0	0	0	0	0	0	0	0	0	4	0	0	0	4	68	0	0	100.00	1.00	1.00	1.00	1.00
16	0	0	0	0	0	0	0	0	0	0	0	0	0	0	0	4	0	0	4	68	0	0	100.00	1.00	1.00	1.00	1.00
17	0	0	0	0	0	0	0	0	0	0	0	0	0	0	0	0	4	0	4	68	0	0	100.00	1.00	1.00	1.00	1.00
18	0	0	0	0	1	0	0	0	0	0	0	0	0	0	0	0	0	3	3	68	0	1	98.61	0.75	1.00	0.86	0.87

Table S14. Confusion matrixes of the calibration set and validation set of GARF-NIR-SVM

Calibration set																											
**Class**	**1**	**2**	**3**	**4**	**5**	**6**	**7**	**8**	**9**	**10**	**11**	**12**	**13**	**14**	**15**	**16**	**17**	**18**	**TP**	**TN**	**FP**	**FN**	**ACC**	**SE**	**SP**	**MCC**	**EFF**
1	6	0	0	0	0	0	0	0	0	0	0	0	0	0	0	0	0	0	6	102	0	0	100.00	1.00	1.00	1.00	1.00
2	0	6	0	0	0	0	0	0	0	0	0	0	0	0	0	0	0	0	6	102	0	0	100.00	1.00	1.00	1.00	1.00
3	0	0	6	0	0	0	0	0	0	0	0	0	0	0	0	0	0	0	6	102	0	0	100.00	1.00	1.00	1.00	1.00
4	0	0	0	6	0	0	0	0	0	0	0	0	0	0	0	0	0	0	6	102	0	0	100.00	1.00	1.00	1.00	1.00
5	0	0	0	0	6	0	0	0	0	0	0	0	0	0	0	0	0	0	6	102	0	0	100.00	1.00	1.00	1.00	1.00
6	0	0	0	0	0	6	0	0	0	0	0	0	0	0	0	0	0	0	6	102	0	0	100.00	1.00	1.00	1.00	1.00
7	0	0	0	0	0	0	6	0	0	0	0	0	0	0	0	0	0	0	6	102	0	0	100.00	1.00	1.00	1.00	1.00
8	0	0	0	0	0	0	0	6	0	0	0	0	0	0	0	0	0	0	6	102	0	0	100.00	1.00	1.00	1.00	1.00
9	0	0	0	0	0	0	0	0	6	0	0	0	0	0	0	0	0	0	6	102	0	0	100.00	1.00	1.00	1.00	1.00
10	0	0	0	0	0	0	0	0	0	6	0	0	0	0	0	0	0	0	6	102	0	0	100.00	1.00	1.00	1.00	1.00
11	0	0	0	0	0	0	0	0	0	0	6	0	0	0	0	0	0	0	6	102	0	0	100.00	1.00	1.00	1.00	1.00
12	0	0	0	0	0	0	0	0	0	0	0	6	0	0	0	0	0	0	6	102	0	0	100.00	1.00	1.00	1.00	1.00
13	0	0	0	0	0	0	0	0	0	0	0	0	6	0	0	0	0	0	6	102	0	0	100.00	1.00	1.00	1.00	1.00
14	0	0	0	0	0	0	0	0	0	0	0	0	0	6	0	0	0	0	6	102	0	0	100.00	1.00	1.00	1.00	1.00
15	0	0	0	0	0	0	0	0	0	0	0	0	0	0	6	0	0	0	6	102	0	0	100.00	1.00	1.00	1.00	1.00
16	0	0	0	0	0	0	0	0	0	0	0	0	0	0	0	6	0	0	6	102	0	0	100.00	1.00	1.00	1.00	1.00
17	0	0	0	0	0	0	0	0	0	0	0	0	0	0	0	0	6	0	6	102	0	0	100.00	1.00	1.00	1.00	1.00
18	0	0	0	0	0	0	0	0	0	0	0	0	0	0	0	0	0	6	6	102	0	0	100.00	1.00	1.00	1.00	1.00

Validation set																												
**Class**	**1**	**2**	**3**	**4**	**5**	**6**	**7**	**8**	**9**	**10**	**11**	**12**	**13**	**14**	**15**	**16**	**17**	**18**	**TP**	**TN**	**FP**	**FN**	**ACC**	**SE**	**SP**	**MCC**	**EFF**
1	3	0	0	0	0	0	1	0	0	0	0	0	0	0	0	0	0	0	3	68	0	1	98.61	0.75	1.00	0.86	0.87
2	0	4	0	0	0	0	0	0	0	0	0	0	0	0	0	0	0	0	4	68	0	0	100.00	1.00	1.00	1.00	1.00
3	0	0	4	0	0	0	0	0	0	0	0	0	0	0	0	0	0	0	4	68	0	0	100.00	1.00	1.00	1.00	1.00
4	0	0	0	4	0	0	0	0	0	0	0	0	0	0	0	0	0	0	4	68	0	0	100.00	1.00	1.00	1.00	1.00
5	0	0	0	0	3	0	0	0	0	0	0	0	0	0	0	0	0	1	3	67	1	1	97.22	0.75	0.99	0.74	0.86
6	0	0	0	0	0	4	0	0	0	0	0	0	0	0	0	0	0	0	4	68	0	0	100.00	1.00	1.00	1.00	1.00
7	0	0	0	0	0	0	4	0	0	0	0	0	0	0	0	0	0	0	4	67	1	0	98.61	1.00	0.99	0.89	0.99
8	0	0	0	0	0	0	0	3	1	0	0	0	0	0	0	0	0	0	3	68	0	1	98.61	0.75	1.00	0.86	0.87
9	0	0	0	0	0	0	0	0	4	0	0	0	0	0	0	0	0	0	4	67	1	0	98.61	1.00	0.99	0.89	0.99
10	0	0	0	0	0	0	0	0	0	4	0	0	0	0	0	0	0	0	4	68	0	0	100.00	1.00	1.00	1.00	1.00
11	0	0	0	0	0	0	0	0	0	0	4	0	0	0	0	0	0	0	4	68	0	0	100.00	1.00	1.00	1.00	1.00
12	0	0	0	0	0	0	0	0	0	0	0	3	0	0	0	1	0	0	3	68	0	1	98.61	0.75	1.00	0.86	0.87
13	0	0	0	0	0	0	0	0	0	0	0	0	4	0	0	0	0	0	4	68	0	0	100.00	1.00	1.00	1.00	1.00
14	0	0	0	0	0	0	0	0	0	0	0	0	0	4	0	0	0	0	4	68	0	0	100.00	1.00	1.00	1.00	1.00
15	0	0	0	0	0	0	0	0	0	0	0	0	0	0	4	0	0	0	4	68	0	0	100.00	1.00	1.00	1.00	1.00
16	0	0	0	0	0	0	0	0	0	0	0	0	0	0	0	4	0	0	4	67	1	0	98.61	1.00	0.99	0.89	0.99
17	0	0	0	0	0	0	0	0	0	0	0	0	0	0	0	0	4	0	4	68	0	0	100.00	1.00	1.00	1.00	1.00
18	0	0	0	0	1	0	0	0	0	0	0	0	0	0	0	0	0	3	3	67	1	1	97.22	0.75	0.99	0.74	0.86

Table S15. Confusion matrixes of the calibration set and validation set of GASVM-NIR-SVM

Calibration set																												
**Class**	**1**	**2**	**3**	**4**	**5**	**6**	**7**	**8**	**9**	**10**	**11**	**12**	**13**	**14**	**15**	**16**	**17**	**18**	**TP**	**TN**	**FP**	**FN**	**ACC**	**SE**	**SP**	**MCC**	**EFF**
1	6	0	0	0	0	0	0	0	0	0	0	0	0	0	0	0	0	0	6	102	0	0	100.00	1.00	1.00	1.00	1.00
2	0	6	0	0	0	0	0	0	0	0	0	0	0	0	0	0	0	0	6	102	0	0	100.00	1.00	1.00	1.00	1.00
3	0	0	6	0	0	0	0	0	0	0	0	0	0	0	0	0	0	0	6	102	0	0	100.00	1.00	1.00	1.00	1.00
4	0	0	0	6	0	0	0	0	0	0	0	0	0	0	0	0	0	0	6	102	0	0	100.00	1.00	1.00	1.00	1.00
5	0	0	0	0	6	0	0	0	0	0	0	0	0	0	0	0	0	0	6	102	0	0	100.00	1.00	1.00	1.00	1.00
6	0	0	0	0	0	6	0	0	0	0	0	0	0	0	0	0	0	0	6	102	0	0	100.00	1.00	1.00	1.00	1.00
7	0	0	0	0	0	0	6	0	0	0	0	0	0	0	0	0	0	0	6	102	0	0	100.00	1.00	1.00	1.00	1.00
8	0	0	0	0	0	0	0	6	0	0	0	0	0	0	0	0	0	0	6	102	0	0	100.00	1.00	1.00	1.00	1.00
9	0	0	0	0	0	0	0	0	6	0	0	0	0	0	0	0	0	0	6	102	0	0	100.00	1.00	1.00	1.00	1.00
10	0	0	0	0	0	0	0	0	0	6	0	0	0	0	0	0	0	0	6	102	0	0	100.00	1.00	1.00	1.00	1.00
11	0	0	0	0	0	0	0	0	0	0	6	0	0	0	0	0	0	0	6	102	0	0	100.00	1.00	1.00	1.00	1.00
12	0	0	0	0	0	0	0	0	0	0	0	6	0	0	0	0	0	0	6	102	0	0	100.00	1.00	1.00	1.00	1.00
13	0	0	0	0	0	0	0	0	0	0	0	0	6	0	0	0	0	0	6	102	0	0	100.00	1.00	1.00	1.00	1.00
14	0	0	0	0	0	0	0	0	0	0	0	0	0	6	0	0	0	0	6	102	0	0	100.00	1.00	1.00	1.00	1.00
15	0	0	0	0	0	0	0	0	0	0	0	0	0	0	6	0	0	0	6	102	0	0	100.00	1.00	1.00	1.00	1.00
16	0	0	0	0	0	0	0	0	0	0	0	0	0	0	0	6	0	0	6	102	0	0	100.00	1.00	1.00	1.00	1.00
17	0	0	0	0	0	0	0	0	0	0	0	0	0	0	0	0	6	0	6	102	0	0	100.00	1.00	1.00	1.00	1.00
18	0	0	0	0	0	0	0	0	0	0	0	0	0	0	0	0	0	6	6	102	0	0	100.00	1.00	1.00	1.00	1.00

Validation set																												
**Class**	**1**	**2**	**3**	**4**	**5**	**6**	**7**	**8**	**9**	**10**	**11**	**12**	**13**	**14**	**15**	**16**	**17**	**18**	**TP**	**TN**	**FP**	**FN**	**ACC**	**SE**	**SP**	**MCC**	**EFF**
1	3	0	0	0	0	0	1	0	0	0	0	0	0	0	0	0	0	0	3	68	0	1	98.61	0.75	1.00	0.86	0.87
2	0	4	0	0	0	0	0	0	0	0	0	0	0	0	0	0	0	0	4	68	0	0	100.00	1.00	1.00	1.00	1.00
3	0	0	4	0	0	0	0	0	0	0	0	0	0	0	0	0	0	0	4	68	0	0	100.00	1.00	1.00	1.00	1.00
4	0	0	0	4	0	0	0	0	0	0	0	0	0	0	0	0	0	0	4	68	0	0	100.00	1.00	1.00	1.00	1.00
5	0	0	0	0	3	0	0	0	0	0	0	0	0	0	0	0	0	1	3	67	1	1	97.22	0.75	0.99	0.74	0.86
6	0	0	0	0	0	4	0	0	0	0	0	0	0	0	0	0	0	0	4	68	0	0	100.00	1.00	1.00	1.00	1.00
7	0	0	0	0	0	0	4	0	0	0	0	0	0	0	0	0	0	0	4	67	1	0	98.61	1.00	0.99	0.89	0.99
8	0	0	0	0	0	0	0	3	1	0	0	0	0	0	0	0	0	0	3	68	0	1	98.61	0.75	1.00	0.86	0.87
9	0	0	0	0	0	0	0	0	4	0	0	0	0	0	0	0	0	0	4	67	1	0	98.61	1.00	0.99	0.89	0.99
10	0	0	0	0	0	0	0	0	0	4	0	0	0	0	0	0	0	0	4	68	0	0	100.00	1.00	1.00	1.00	1.00
11	0	0	0	0	0	0	0	0	0	0	4	0	0	0	0	0	0	0	4	68	0	0	100.00	1.00	1.00	1.00	1.00
12	0	0	0	0	0	0	0	0	0	0	0	3	0	0	0	1	0	0	3	67	1	1	97.22	0.75	0.99	0.74	0.86
13	0	0	0	0	0	0	0	0	0	0	0	0	4	0	0	0	0	0	4	68	0	0	100.00	1.00	1.00	1.00	1.00
14	0	0	0	0	0	0	0	0	0	0	0	0	0	4	0	0	0	0	4	68	0	0	100.00	1.00	1.00	1.00	1.00
15	0	0	0	0	0	0	0	0	0	0	0	0	0	0	4	0	0	0	4	68	0	0	100.00	1.00	1.00	1.00	1.00
16	0	0	0	0	0	0	0	0	0	0	0	1	0	0	0	3	0	0	3	67	1	1	97.22	0.75	0.99	0.74	0.86
17	0	0	0	0	0	0	0	0	0	0	0	0	0	0	0	0	4	0	4	68	0	0	100.00	1.00	1.00	1.00	1.00
18	0	0	0	0	1	0	0	0	0	0	0	0	0	0	0	0	0	3	3	67	1	1	97.22	0.75	0.99	0.74	0.86

Table S16. Confusion matrixes of the calibration set and validation set of Ven-NIR-SVM

Calibration set																											
**Class**	**1**	**2**	**3**	**4**	**5**	**6**	**7**	**8**	**9**	**10**	**11**	**12**	**13**	**14**	**15**	**16**	**17**	**18**	**TP**	**TN**	**FP**	**FN**	**ACC**	**SE**	**SP**	**MCC**	**EFF**
1	6	0	0	0	0	0	0	0	0	0	0	0	0	0	0	0	0	0	6	102	0	0	100.00	1.00	1.00	1.00	1.00
2	0	6	0	0	0	0	0	0	0	0	0	0	0	0	0	0	0	0	6	102	0	0	100.00	1.00	1.00	1.00	1.00
3	0	0	6	0	0	0	0	0	0	0	0	0	0	0	0	0	0	0	6	102	0	0	100.00	1.00	1.00	1.00	1.00
4	0	0	0	6	0	0	0	0	0	0	0	0	0	0	0	0	0	0	6	102	0	0	100.00	1.00	1.00	1.00	1.00
5	0	0	0	0	6	0	0	0	0	0	0	0	0	0	0	0	0	0	6	102	0	0	100.00	1.00	1.00	1.00	1.00
6	0	0	0	0	0	6	0	0	0	0	0	0	0	0	0	0	0	0	6	102	0	0	100.00	1.00	1.00	1.00	1.00
7	0	0	0	0	0	0	6	0	0	0	0	0	0	0	0	0	0	0	6	102	0	0	100.00	1.00	1.00	1.00	1.00
8	0	0	0	0	0	0	0	6	0	0	0	0	0	0	0	0	0	0	6	102	0	0	100.00	1.00	1.00	1.00	1.00
9	0	0	0	0	0	0	0	0	6	0	0	0	0	0	0	0	0	0	6	102	0	0	100.00	1.00	1.00	1.00	1.00
10	0	0	0	0	0	0	0	0	0	6	0	0	0	0	0	0	0	0	6	102	0	0	100.00	1.00	1.00	1.00	1.00
11	0	0	0	0	0	0	0	0	0	0	6	0	0	0	0	0	0	0	6	102	0	0	100.00	1.00	1.00	1.00	1.00
12	0	0	0	0	0	0	0	0	0	0	0	6	0	0	0	0	0	0	6	102	0	0	100.00	1.00	1.00	1.00	1.00
13	0	0	0	0	0	0	0	0	0	0	0	0	6	0	0	0	0	0	6	102	0	0	100.00	1.00	1.00	1.00	1.00
14	0	0	0	0	0	0	0	0	0	0	0	0	0	6	0	0	0	0	6	102	0	0	100.00	1.00	1.00	1.00	1.00
15	0	0	0	0	0	0	0	0	0	0	0	0	0	0	6	0	0	0	6	102	0	0	100.00	1.00	1.00	1.00	1.00
16	0	0	0	0	0	0	0	0	0	0	0	0	0	0	0	6	0	0	6	102	0	0	100.00	1.00	1.00	1.00	1.00
17	0	0	0	0	0	0	0	0	0	0	0	0	0	0	0	0	6	0	6	102	0	0	100.00	1.00	1.00	1.00	1.00
18	0	0	0	0	0	0	0	0	0	0	0	0	0	0	0	0	0	6	6	102	0	0	100.00	1.00	1.00	1.00	1.00

Validation set																												
**Class**	**1**	**2**	**3**	**4**	**5**	**6**	**7**	**8**	**9**	**10**	**11**	**12**	**13**	**14**	**15**	**16**	**17**	**18**	**TP**	**TN**	**FP**	**FN**	**ACC**	**SE**	**SP**	**MCC**	**EFF**
1	4	0	0	0	0	0	0	0	0	0	0	0	0	0	0	0	0	0	4	68	0	0	100.00	1.00	1.00	1.00	1.00
2	0	4	0	0	0	0	0	0	0	0	0	0	0	0	0	0	0	0	4	68	0	0	100.00	1.00	1.00	1.00	1.00
3	0	0	4	0	0	0	0	0	0	0	0	0	0	0	0	0	0	0	4	68	0	0	100.00	1.00	1.00	1.00	1.00
4	0	0	0	4	0	0	0	0	0	0	0	0	0	0	0	0	0	0	4	68	0	0	100.00	1.00	1.00	1.00	1.00
5	0	0	0	0	4	0	0	0	0	0	0	0	0	0	0	0	0	0	4	67	1	0	98.61	1.00	0.99	0.89	0.99
6	0	0	0	0	0	4	0	0	0	0	0	0	0	0	0	0	0	0	4	68	0	0	100.00	1.00	1.00	1.00	1.00
7	0	0	0	0	0	0	4	0	0	0	0	0	0	0	0	0	0	0	4	68	0	0	100.00	1.00	1.00	1.00	1.00
8	0	0	0	0	0	0	0	4	0	0	0	0	0	0	0	0	0	0	4	68	0	0	100.00	1.00	1.00	1.00	1.00
9	0	0	0	0	0	0	0	0	4	0	0	0	0	0	0	0	0	0	4	68	0	0	100.00	1.00	1.00	1.00	1.00
10	0	0	0	0	0	0	0	0	0	4	0	0	0	0	0	0	0	0	4	68	0	0	100.00	1.00	1.00	1.00	1.00
11	0	0	0	0	0	0	0	0	0	0	4	0	0	0	0	0	0	0	4	68	0	0	100.00	1.00	1.00	1.00	1.00
12	0	0	0	0	0	0	0	0	0	0	0	4	0	0	0	0	0	0	4	68	0	0	100.00	1.00	1.00	1.00	1.00
13	0	0	0	0	0	0	0	0	0	0	0	0	4	0	0	0	0	0	4	68	0	0	100.00	1.00	1.00	1.00	1.00
14	0	0	0	0	0	0	0	0	0	0	0	0	0	4	0	0	0	0	4	68	0	0	100.00	1.00	1.00	1.00	1.00
15	0	0	0	0	0	0	0	0	0	0	0	0	0	0	4	0	0	0	4	68	0	0	100.00	1.00	1.00	1.00	1.00
16	0	0	0	0	0	0	0	0	0	0	0	0	0	0	0	4	0	0	4	68	0	0	100.00	1.00	1.00	1.00	1.00
17	0	0	0	0	0	0	0	0	0	0	0	0	0	0	0	0	4	0	4	68	0	0	100.00	1.00	1.00	1.00	1.00
18	0	0	0	0	1	0	0	0	0	0	0	0	0	0	0	0	0	3	3	68	0	1	98.61	0.75	1.00	0.86	0.87

Table S17. Confusion matrixes of the calibration set and validation set of VIP-NIR-KNN

Calibration set																											
**Class**	**1**	**2**	**3**	**4**	**5**	**6**	**7**	**8**	**9**	**10**	**11**	**12**	**13**	**14**	**15**	**16**	**17**	**18**	**TP**	**TN**	**FP**	**FN**	**ACC**	**SE**	**SP**	**MCC**	**EFF**
1	6	0	0	0	0	0	0	0	0	0	0	0	0	0	0	0	0	0	6	102	0	0	100.00	1.00	1.00	1.00	1.00
2	0	6	0	0	0	0	0	0	0	0	0	0	0	0	0	0	0	0	6	102	0	0	100.00	1.00	1.00	1.00	1.00
3	0	0	6	0	0	0	0	0	0	0	0	0	0	0	0	0	0	0	6	102	0	0	100.00	1.00	1.00	1.00	1.00
4	0	0	0	6	0	0	0	0	0	0	0	0	0	0	0	0	0	0	6	102	0	0	100.00	1.00	1.00	1.00	1.00
5	0	0	0	0	6	0	0	0	0	0	0	0	0	0	0	0	0	0	6	102	0	0	100.00	1.00	1.00	1.00	1.00
6	0	0	0	0	0	6	0	0	0	0	0	0	0	0	0	0	0	0	6	102	0	0	100.00	1.00	1.00	1.00	1.00
7	0	0	0	0	0	0	6	0	0	0	0	0	0	0	0	0	0	0	6	102	0	0	100.00	1.00	1.00	1.00	1.00
8	0	0	0	0	0	0	0	6	0	0	0	0	0	0	0	0	0	0	6	102	0	0	100.00	1.00	1.00	1.00	1.00
9	0	0	0	0	0	0	0	0	6	0	0	0	0	0	0	0	0	0	6	102	0	0	100.00	1.00	1.00	1.00	1.00
10	0	0	0	0	0	0	0	0	0	6	0	0	0	0	0	0	0	0	6	102	0	0	100.00	1.00	1.00	1.00	1.00
11	0	0	0	0	0	0	0	0	0	0	6	0	0	0	0	0	0	0	6	102	0	0	100.00	1.00	1.00	1.00	1.00
12	0	0	0	0	0	0	0	0	0	0	0	6	0	0	0	0	0	0	6	102	0	0	100.00	1.00	1.00	1.00	1.00
13	0	0	0	0	0	0	0	0	0	0	0	0	6	0	0	0	0	0	6	102	0	0	100.00	1.00	1.00	1.00	1.00
14	0	0	0	0	0	0	0	0	0	0	0	0	0	6	0	0	0	0	6	102	0	0	100.00	1.00	1.00	1.00	1.00
15	0	0	0	0	0	0	0	0	0	0	0	0	0	0	6	0	0	0	6	102	0	0	100.00	1.00	1.00	1.00	1.00
16	0	0	0	0	0	0	0	0	0	0	0	0	0	0	0	6	0	0	6	102	0	0	100.00	1.00	1.00	1.00	1.00
17	0	0	0	0	0	0	0	0	0	0	0	0	0	0	0	0	6	0	6	102	0	0	100.00	1.00	1.00	1.00	1.00
18	0	0	0	0	0	0	0	0	0	0	0	0	0	0	0	0	0	6	6	102	0	0	100.00	1.00	1.00	1.00	1.00

Validation set																												
**Class**	**1**	**2**	**3**	**4**	**5**	**6**	**7**	**8**	**9**	**10**	**11**	**12**	**13**	**14**	**15**	**16**	**17**	**18**	**TP**	**TN**	**FP**	**FN**	**ACC**	**SE**	**SP**	**MCC**	**EFF**
1	4	0	0	0	0	0	0	0	0	0	0	0	0	0	0	0	0	0	4	67	1	0	98.61	1.00	0.99	0.89	0.99
2	1	3	0	0	0	0	0	0	0	0	0	0	0	0	0	0	0	0	3	68	0	1	98.61	0.75	1.00	0.86	0.87
3	0	0	4	0	0	0	0	0	0	0	0	0	0	0	0	0	0	0	4	67	1	0	98.61	1.00	0.99	0.89	0.99
4	0	0	0	4	0	0	0	0	0	0	0	0	0	0	0	0	0	0	4	68	0	0	100.00	1.00	1.00	1.00	1.00
5	0	0	0	0	4	0	0	0	0	0	0	0	0	0	0	0	0	0	4	68	0	0	100.00	1.00	1.00	1.00	1.00
6	0	0	0	0	0	4	0	0	0	0	0	0	0	0	0	0	0	0	4	68	0	0	100.00	1.00	1.00	1.00	1.00
7	0	0	0	0	0	0	4	0	0	0	0	0	0	0	0	0	0	0	4	68	0	0	100.00	1.00	1.00	1.00	1.00
8	0	0	0	0	0	0	0	4	0	0	0	0	0	0	0	0	0	0	4	68	0	0	100.00	1.00	1.00	1.00	1.00
9	0	0	0	0	0	0	0	0	3	0	0	1	0	0	0	0	0	0	3	68	0	1	98.61	0.75	1.00	0.86	0.87
10	0	0	0	0	0	0	0	0	0	4	0	0	0	0	0	0	0	0	4	68	0	0	100.00	1.00	1.00	1.00	1.00
11	0	0	0	0	0	0	0	0	0	0	4	0	0	0	0	0	0	0	4	68	0	0	100.00	1.00	1.00	1.00	1.00
12	0	0	0	0	0	0	0	0	0	0	0	4	0	0	0	0	0	0	4	67	1	0	98.61	1.00	0.99	0.89	0.99
13	0	0	0	0	0	0	0	0	0	0	0	0	4	0	0	0	0	0	4	68	0	0	100.00	1.00	1.00	1.00	1.00
14	0	0	0	0	0	0	0	0	0	0	0	0	0	4	0	0	0	0	4	68	0	0	100.00	1.00	1.00	1.00	1.00
15	0	0	0	0	0	0	0	0	0	0	0	0	0	0	4	0	0	0	4	68	0	0	100.00	1.00	1.00	1.00	1.00
16	0	0	0	0	0	0	0	0	0	0	0	0	0	0	0	4	0	0	4	68	0	0	100.00	1.00	1.00	1.00	1.00
17	0	0	0	0	0	0	0	0	0	0	0	0	0	0	0	0	4	0	4	68	0	0	100.00	1.00	1.00	1.00	1.00
18	0	0	1	0	0	0	0	0	0	0	0	0	0	0	0	0	0	3	3	68	0	1	98.61	0.75	1.00	0.86	0.87

Table S18. Confusion matrixes of the calibration set and validation set of Bor-NIR-KNN

Calibration set																											
**Class**	**1**	**2**	**3**	**4**	**5**	**6**	**7**	**8**	**9**	**10**	**11**	**12**	**13**	**14**	**15**	**16**	**17**	**18**	**TP**	**TN**	**FP**	**FN**	**ACC**	**SE**	**SP**	**MCC**	**EFF**
1	6	0	0	0	0	0	0	0	0	0	0	0	0	0	0	0	0	0	6	102	0	0	100.00	1.00	1.00	1.00	1.00
2	0	6	0	0	0	0	0	0	0	0	0	0	0	0	0	0	0	0	6	102	0	0	100.00	1.00	1.00	1.00	1.00
3	0	0	6	0	0	0	0	0	0	0	0	0	0	0	0	0	0	0	6	102	0	0	100.00	1.00	1.00	1.00	1.00
4	0	0	0	6	0	0	0	0	0	0	0	0	0	0	0	0	0	0	6	102	0	0	100.00	1.00	1.00	1.00	1.00
5	0	0	0	0	6	0	0	0	0	0	0	0	0	0	0	0	0	0	6	102	0	0	100.00	1.00	1.00	1.00	1.00
6	0	0	0	0	0	6	0	0	0	0	0	0	0	0	0	0	0	0	6	102	0	0	100.00	1.00	1.00	1.00	1.00
7	0	0	0	0	0	0	6	0	0	0	0	0	0	0	0	0	0	0	6	102	0	0	100.00	1.00	1.00	1.00	1.00
8	0	0	0	0	0	0	0	6	0	0	0	0	0	0	0	0	0	0	6	102	0	0	100.00	1.00	1.00	1.00	1.00
9	0	0	0	0	0	0	0	0	6	0	0	0	0	0	0	0	0	0	6	102	0	0	100.00	1.00	1.00	1.00	1.00
10	0	0	0	0	0	0	0	0	0	6	0	0	0	0	0	0	0	0	6	102	0	0	100.00	1.00	1.00	1.00	1.00
11	0	0	0	0	0	0	0	0	0	0	6	0	0	0	0	0	0	0	6	102	0	0	100.00	1.00	1.00	1.00	1.00
12	0	0	0	0	0	0	0	0	0	0	0	6	0	0	0	0	0	0	6	102	0	0	100.00	1.00	1.00	1.00	1.00
13	0	0	0	0	0	0	0	0	0	0	0	0	6	0	0	0	0	0	6	102	0	0	100.00	1.00	1.00	1.00	1.00
14	0	0	0	0	0	0	0	0	0	0	0	0	0	6	0	0	0	0	6	102	0	0	100.00	1.00	1.00	1.00	1.00
15	0	0	0	0	0	0	0	0	0	0	0	0	0	0	6	0	0	0	6	102	0	0	100.00	1.00	1.00	1.00	1.00
16	0	0	0	0	0	0	0	0	0	0	0	0	0	0	0	6	0	0	6	102	0	0	100.00	1.00	1.00	1.00	1.00
17	0	0	0	0	0	0	0	0	0	0	0	0	0	0	0	0	6	0	6	102	0	0	100.00	1.00	1.00	1.00	1.00
18	0	0	0	0	0	0	0	0	0	0	0	0	0	0	0	0	0	6	6	102	0	0	100.00	1.00	1.00	1.00	1.00

Validation set																											
**Class**	**1**	**2**	**3**	**4**	**5**	**6**	**7**	**8**	**9**	**10**	**11**	**12**	**13**	**14**	**15**	**16**	**17**	**18**	**TP**	**TN**	**FP**	**FN**	**ACC**	**SE**	**SP**	**MCC**	**EFF**
1	4	0	0	0	0	0	0	0	0	0	0	0	0	0	0	0	0	0	4	67	1	0	98.61	1.00	0.99	0.89	0.99
2	1	2	0	0	1	0	0	0	0	0	0	0	0	0	0	0	0	0	2	68	0	2	97.22	0.50	1.00	0.70	0.71
3	0	0	4	0	0	0	0	0	0	0	0	0	0	0	0	0	0	0	4	68	0	0	100.00	1.00	1.00	1.00	1.00
4	0	0	0	4	0	0	0	0	0	0	0	0	0	0	0	0	0	0	4	68	0	0	100.00	1.00	1.00	1.00	1.00
5	0	0	0	0	4	0	0	0	0	0	0	0	0	0	0	0	0	0	4	66	2	0	97.22	1.00	0.97	0.80	0.99
6	0	0	0	0	0	4	0	0	0	0	0	0	0	0	0	0	0	0	4	68	0	0	100.00	1.00	1.00	1.00	1.00
7	0	0	0	0	0	0	3	0	1	0	0	0	0	0	0	0	0	0	3	68	0	1	98.61	0.75	1.00	0.86	0.87
8	0	0	0	0	0	0	0	4	0	0	0	0	0	0	0	0	0	0	4	68	0	0	100.00	1.00	1.00	1.00	1.00
9	0	0	0	0	0	0	0	0	4	0	0	0	0	0	0	0	0	0	4	67	1	0	98.61	1.00	0.99	0.89	0.99
10	0	0	0	0	0	0	0	0	0	4	0	0	0	0	0	0	0	0	4	68	0	0	100.00	1.00	1.00	1.00	1.00
11	0	0	0	0	0	0	0	0	0	0	4	0	0	0	0	0	0	0	4	68	0	0	100.00	1.00	1.00	1.00	1.00
12	0	0	0	0	0	0	0	0	0	0	0	4	0	0	0	0	0	0	4	68	0	0	100.00	1.00	1.00	1.00	1.00
13	0	0	0	0	0	0	0	0	0	0	0	0	4	0	0	0	0	0	4	68	0	0	100.00	1.00	1.00	1.00	1.00
14	0	0	0	0	0	0	0	0	0	0	0	0	0	4	0	0	0	0	4	68	0	0	100.00	1.00	1.00	1.00	1.00
15	0	0	0	0	0	0	0	0	0	0	0	0	0	0	4	0	0	0	4	68	0	0	100.00	1.00	1.00	1.00	1.00
16	0	0	0	0	0	0	0	0	0	0	0	0	0	0	0	4	0	0	4	68	0	0	100.00	1.00	1.00	1.00	1.00
17	0	0	0	0	0	0	0	0	0	0	0	0	0	0	0	0	4	0	4	68	0	0	100.00	1.00	1.00	1.00	1.00
18	0	0	0	0	1	0	0	0	0	0	0	0	0	0	0	0	0	3	3	68	0	1	98.61	0.75	1.00	0.86	0.87

Table S19. Confusion matrixes of the calibration set and validation set of GARF-NIR-KNN

Calibration set																											
**Class**	**1**	**2**	**3**	**4**	**5**	**6**	**7**	**8**	**9**	**10**	**11**	**12**	**13**	**14**	**15**	**16**	**17**	**18**	**TP**	**TN**	**FP**	**FN**	**ACC**	**SE**	**SP**	**MCC**	**EFF**
1	6	0	0	0	0	0	0	0	0	0	0	0	0	0	0	0	0	0	6	102	0	0	100.00	1.00	1.00	1.00	1.00
2	0	6	0	0	0	0	0	0	0	0	0	0	0	0	0	0	0	0	6	102	0	0	100.00	1.00	1.00	1.00	1.00
3	0	0	6	0	0	0	0	0	0	0	0	0	0	0	0	0	0	0	6	102	0	0	100.00	1.00	1.00	1.00	1.00
4	0	0	0	6	0	0	0	0	0	0	0	0	0	0	0	0	0	0	6	102	0	0	100.00	1.00	1.00	1.00	1.00
5	0	0	0	0	6	0	0	0	0	0	0	0	0	0	0	0	0	0	6	102	0	0	100.00	1.00	1.00	1.00	1.00
6	0	0	0	0	0	6	0	0	0	0	0	0	0	0	0	0	0	0	6	102	0	0	100.00	1.00	1.00	1.00	1.00
7	0	0	0	0	0	0	6	0	0	0	0	0	0	0	0	0	0	0	6	102	0	0	100.00	1.00	1.00	1.00	1.00
8	0	0	0	0	0	0	0	6	0	0	0	0	0	0	0	0	0	0	6	102	0	0	100.00	1.00	1.00	1.00	1.00
9	0	0	0	0	0	0	0	0	6	0	0	0	0	0	0	0	0	0	6	102	0	0	100.00	1.00	1.00	1.00	1.00
10	0	0	0	0	0	0	0	0	0	6	0	0	0	0	0	0	0	0	6	102	0	0	100.00	1.00	1.00	1.00	1.00
11	0	0	0	0	0	0	0	0	0	0	6	0	0	0	0	0	0	0	6	102	0	0	100.00	1.00	1.00	1.00	1.00
12	0	0	0	0	0	0	0	0	0	0	0	6	0	0	0	0	0	0	6	102	0	0	100.00	1.00	1.00	1.00	1.00
13	0	0	0	0	0	0	0	0	0	0	0	0	6	0	0	0	0	0	6	102	0	0	100.00	1.00	1.00	1.00	1.00
14	0	0	0	0	0	0	0	0	0	0	0	0	0	6	0	0	0	0	6	102	0	0	100.00	1.00	1.00	1.00	1.00
15	0	0	0	0	0	0	0	0	0	0	0	0	0	0	6	0	0	0	6	102	0	0	100.00	1.00	1.00	1.00	1.00
16	0	0	0	0	0	0	0	0	0	0	0	0	0	0	0	6	0	0	6	102	0	0	100.00	1.00	1.00	1.00	1.00
17	0	0	0	0	0	0	0	0	0	0	0	0	0	0	0	0	6	0	6	102	0	0	100.00	1.00	1.00	1.00	1.00
18	0	0	0	0	0	0	0	0	0	0	0	0	0	0	0	0	0	6	6	102	0	0	100.00	1.00	1.00	1.00	1.00

Validation set																											
**Class**	**1**	**2**	**3**	**4**	**5**	**6**	**7**	**8**	**9**	**10**	**11**	**12**	**13**	**14**	**15**	**16**	**17**	**18**	**TP**	**TN**	**FP**	**FN**	**ACC**	**SE**	**SP**	**MCC**	**EFF**
1	4	0	0	0	0	0	0	0	0	0	0	0	0	0	0	0	0	0	4	67	1	0	98.61	1.00	0.99	0.89	0.99
2	1	2	0	0	1	0	0	0	0	0	0	0	0	0	0	0	0	0	2	68	0	2	97.22	0.50	1.00	0.70	0.71
3	0	0	4	0	0	0	0	0	0	0	0	0	0	0	0	0	0	0	4	68	0	0	100.00	1.00	1.00	1.00	1.00
4	0	0	0	4	0	0	0	0	0	0	0	0	0	0	0	0	0	0	4	68	0	0	100.00	1.00	1.00	1.00	1.00
5	0	0	0	0	3	0	0	0	0	0	0	0	0	0	0	0	0	1	3	67	1	1	97.22	0.75	0.99	0.74	0.86
6	0	0	0	0	0	4	0	0	0	0	0	0	0	0	0	0	0	0	4	68	0	0	100.00	1.00	1.00	1.00	1.00
7	0	0	0	0	0	0	3	0	1	0	0	0	0	0	0	0	0	0	3	68	0	1	98.61	0.75	1.00	0.86	0.87
8	0	0	0	0	0	0	0	4	0	0	0	0	0	0	0	0	0	0	4	68	0	0	100.00	1.00	1.00	1.00	1.00
9	0	0	0	0	0	0	0	0	2	0	0	2	0	0	0	0	0	0	2	67	1	2	95.83	0.50	0.99	0.56	0.70
10	0	0	0	0	0	0	0	0	0	4	0	0	0	0	0	0	0	0	4	67	1	0	98.61	1.00	0.99	0.89	0.99
11	0	0	0	0	0	0	0	0	0	0	4	0	0	0	0	0	0	0	4	68	0	0	100.00	1.00	1.00	1.00	1.00
12	0	0	0	0	0	0	0	0	0	0	0	3	0	0	0	1	0	0	3	65	3	1	94.44	0.75	0.96	0.59	0.85
13	0	0	0	0	0	0	0	0	0	0	0	0	4	0	0	0	0	0	4	68	0	0	100.00	1.00	1.00	1.00	1.00
14	0	0	0	0	0	0	0	0	0	0	0	0	0	4	0	0	0	0	4	68	0	0	100.00	1.00	1.00	1.00	1.00
15	0	0	0	0	0	0	0	0	0	0	0	0	0	0	4	0	0	0	4	68	0	0	100.00	1.00	1.00	1.00	1.00
16	0	0	0	0	0	0	0	0	0	0	0	1	0	0	0	3	0	0	3	67	1	1	97.22	0.75	0.99	0.74	0.86
17	0	0	0	0	0	0	0	0	0	0	0	0	0	0	0	0	4	0	4	68	0	0	100.00	1.00	1.00	1.00	1.00
18	0	0	0	0	0	0	0	0	0	1	0	0	0	0	0	0	0	3	3	67	1	1	97.22	0.75	0.99	0.74	0.86

Table S20. Confusion matrixes of the calibration set and validation set of GASVM-NIR-KNN

Calibration set																											
**Class**	**1**	**2**	**3**	**4**	**5**	**6**	**7**	**8**	**9**	**10**	**11**	**12**	**13**	**14**	**15**	**16**	**17**	**18**	**TP**	**TN**	**FP**	**FN**	**ACC**	**SE**	**SP**	**MCC**	**EFF**
1	6	0	0	0	0	0	0	0	0	0	0	0	0	0	0	0	0	0	6	102	0	0	100.00	1.00	1.00	1.00	1.00
2	0	6	0	0	0	0	0	0	0	0	0	0	0	0	0	0	0	0	6	102	0	0	100.00	1.00	1.00	1.00	1.00
3	0	0	6	0	0	0	0	0	0	0	0	0	0	0	0	0	0	0	6	102	0	0	100.00	1.00	1.00	1.00	1.00
4	0	0	0	6	0	0	0	0	0	0	0	0	0	0	0	0	0	0	6	102	0	0	100.00	1.00	1.00	1.00	1.00
5	0	0	0	0	6	0	0	0	0	0	0	0	0	0	0	0	0	0	6	102	0	0	100.00	1.00	1.00	1.00	1.00
6	0	0	0	0	0	6	0	0	0	0	0	0	0	0	0	0	0	0	6	102	0	0	100.00	1.00	1.00	1.00	1.00
7	0	0	0	0	0	0	6	0	0	0	0	0	0	0	0	0	0	0	6	102	0	0	100.00	1.00	1.00	1.00	1.00
8	0	0	0	0	0	0	0	6	0	0	0	0	0	0	0	0	0	0	6	102	0	0	100.00	1.00	1.00	1.00	1.00
9	0	0	0	0	0	0	0	0	6	0	0	0	0	0	0	0	0	0	6	102	0	0	100.00	1.00	1.00	1.00	1.00
10	0	0	0	0	0	0	0	0	0	6	0	0	0	0	0	0	0	0	6	102	0	0	100.00	1.00	1.00	1.00	1.00
11	0	0	0	0	0	0	0	0	0	0	6	0	0	0	0	0	0	0	6	102	0	0	100.00	1.00	1.00	1.00	1.00
12	0	0	0	0	0	0	0	0	0	0	0	6	0	0	0	0	0	0	6	102	0	0	100.00	1.00	1.00	1.00	1.00
13	0	0	0	0	0	0	0	0	0	0	0	0	6	0	0	0	0	0	6	102	0	0	100.00	1.00	1.00	1.00	1.00
14	0	0	0	0	0	0	0	0	0	0	0	0	0	6	0	0	0	0	6	102	0	0	100.00	1.00	1.00	1.00	1.00
15	0	0	0	0	0	0	0	0	0	0	0	0	0	0	6	0	0	0	6	102	0	0	100.00	1.00	1.00	1.00	1.00
16	0	0	0	0	0	0	0	0	0	0	0	0	0	0	0	6	0	0	6	102	0	0	100.00	1.00	1.00	1.00	1.00
17	0	0	0	0	0	0	0	0	0	0	0	0	0	0	0	0	6	0	6	102	0	0	100.00	1.00	1.00	1.00	1.00
18	0	0	0	0	0	0	0	0	0	0	0	0	0	0	0	0	0	6	6	102	0	0	100.00	1.00	1.00	1.00	1.00

Validation set																												
**Class**	**1**	**2**	**3**	**4**	**5**	**6**	**7**	**8**	**9**	**10**	**11**	**12**	**13**	**14**	**15**	**16**	**17**	**18**	**TP**	**TN**	**FP**	**FN**	**ACC**	**SE**	**SP**	**MCC**	**EFF**
1	4	0	0	0	0	0	0	0	0	0	0	0	0	0	0	0	0	0	4	67	1	0	98.61	1.00	0.99	0.89	0.99
2	1	2	0	0	1	0	0	0	0	0	0	0	0	0	0	0	0	0	2	68	0	2	97.22	0.50	1.00	0.70	0.71
3	0	0	4	0	0	0	0	0	0	0	0	0	0	0	0	0	0	0	4	68	0	0	100.00	1.00	1.00	1.00	1.00
4	0	0	0	4	0	0	0	0	0	0	0	0	0	0	0	0	0	0	4	68	0	0	100.00	1.00	1.00	1.00	1.00
5	0	0	0	0	3	0	0	0	0	0	0	0	0	0	0	0	0	1	3	66	2	1	95.83	0.75	0.97	0.65	0.85
6	0	0	0	0	0	4	0	0	0	0	0	0	0	0	0	0	0	0	4	68	0	0	100.00	1.00	1.00	1.00	1.00
7	0	0	0	0	0	0	3	0	1	0	0	0	0	0	0	0	0	0	3	68	0	1	98.61	0.75	1.00	0.86	0.87
8	0	0	0	0	0	0	0	4	0	0	0	0	0	0	0	0	0	0	4	68	0	0	100.00	1.00	1.00	1.00	1.00
9	0	0	0	0	0	0	0	0	3	0	0	1	0	0	0	0	0	0	3	67	1	1	97.22	0.75	0.99	0.74	0.86
10	0	0	0	0	0	0	0	0	0	4	0	0	0	0	0	0	0	0	4	68	0	0	100.00	1.00	1.00	1.00	1.00
11	0	0	0	0	0	0	0	0	0	0	4	0	0	0	0	0	0	0	4	68	0	0	100.00	1.00	1.00	1.00	1.00
12	0	0	0	0	0	0	0	0	0	0	0	3	0	0	0	1	0	0	3	66	2	1	95.83	0.75	0.97	0.65	0.85
13	0	0	0	0	0	0	0	0	0	0	0	0	4	0	0	0	0	0	4	68	0	0	100.00	1.00	1.00	1.00	1.00
14	0	0	0	0	0	0	0	0	0	0	0	0	0	4	0	0	0	0	4	68	0	0	100.00	1.00	1.00	1.00	1.00
15	0	0	0	0	0	0	0	0	0	0	0	0	0	0	4	0	0	0	4	68	0	0	100.00	1.00	1.00	1.00	1.00
16	0	0	0	0	0	0	0	0	0	0	0	1	0	0	0	3	0	0	3	67	1	1	97.22	0.75	0.99	0.74	0.86
17	0	0	0	0	0	0	0	0	0	0	0	0	0	0	0	0	4	0	4	68	0	0	100.00	1.00	1.00	1.00	1.00
18	0	0	0	0	1	0	0	0	0	0	0	0	0	0	0	0	0	3	3	67	1	1	97.22	0.75	0.99	0.74	0.86

Table S21. Confusion matrixes of the calibration set and validation set of Ven-NIR-KNN

Calibration set																											
**Class**	**1**	**2**	**3**	**4**	**5**	**6**	**7**	**8**	**9**	**10**	**11**	**12**	**13**	**14**	**15**	**16**	**17**	**18**	**TP**	**TN**	**FP**	**FN**	**ACC**	**SE**	**SP**	**MCC**	**EFF**
1	6	0	0	0	0	0	0	0	0	0	0	0	0	0	0	0	0	0	6	102	0	0	100.00	1.00	1.00	1.00	1.00
2	0	6	0	0	0	0	0	0	0	0	0	0	0	0	0	0	0	0	6	102	0	0	100.00	1.00	1.00	1.00	1.00
3	0	0	6	0	0	0	0	0	0	0	0	0	0	0	0	0	0	0	6	102	0	0	100.00	1.00	1.00	1.00	1.00
4	0	0	0	6	0	0	0	0	0	0	0	0	0	0	0	0	0	0	6	102	0	0	100.00	1.00	1.00	1.00	1.00
5	0	0	0	0	6	0	0	0	0	0	0	0	0	0	0	0	0	0	6	102	0	0	100.00	1.00	1.00	1.00	1.00
6	0	0	0	0	0	6	0	0	0	0	0	0	0	0	0	0	0	0	6	102	0	0	100.00	1.00	1.00	1.00	1.00
7	0	0	0	0	0	0	6	0	0	0	0	0	0	0	0	0	0	0	6	102	0	0	100.00	1.00	1.00	1.00	1.00
8	0	0	0	0	0	0	0	6	0	0	0	0	0	0	0	0	0	0	6	102	0	0	100.00	1.00	1.00	1.00	1.00
9	0	0	0	0	0	0	0	0	6	0	0	0	0	0	0	0	0	0	6	102	0	0	100.00	1.00	1.00	1.00	1.00
10	0	0	0	0	0	0	0	0	0	6	0	0	0	0	0	0	0	0	6	102	0	0	100.00	1.00	1.00	1.00	1.00
11	0	0	0	0	0	0	0	0	0	0	6	0	0	0	0	0	0	0	6	102	0	0	100.00	1.00	1.00	1.00	1.00
12	0	0	0	0	0	0	0	0	0	0	0	6	0	0	0	0	0	0	6	102	0	0	100.00	1.00	1.00	1.00	1.00
13	0	0	0	0	0	0	0	0	0	0	0	0	6	0	0	0	0	0	6	102	0	0	100.00	1.00	1.00	1.00	1.00
14	0	0	0	0	0	0	0	0	0	0	0	0	0	6	0	0	0	0	6	102	0	0	100.00	1.00	1.00	1.00	1.00
15	0	0	0	0	0	0	0	0	0	0	0	0	0	0	6	0	0	0	6	102	0	0	100.00	1.00	1.00	1.00	1.00
16	0	0	0	0	0	0	0	0	0	0	0	0	0	0	0	6	0	0	6	102	0	0	100.00	1.00	1.00	1.00	1.00
17	0	0	0	0	0	0	0	0	0	0	0	0	0	0	0	0	6	0	6	102	0	0	100.00	1.00	1.00	1.00	1.00
18	0	0	0	0	0	0	0	0	0	0	0	0	0	0	0	0	0	6	6	102	0	0	100.00	1.00	1.00	1.00	1.00

Validation set																											
**Class**	**1**	**2**	**3**	**4**	**5**	**6**	**7**	**8**	**9**	**10**	**11**	**12**	**13**	**14**	**15**	**16**	**17**	**18**	**TP**	**TN**	**FP**	**FN**	**ACC**	**SE**	**SP**	**MCC**	**EFF**
1	4	0	0	0	0	0	0	0	0	0	0	0	0	0	0	0	0	0	4	67	1	0	98.61	1.00	0.99	0.89	0.99
2	1	3	0	0	0	0	0	0	0	0	0	0	0	0	0	0	0	0	3	68	0	1	98.61	0.75	1.00	0.86	0.87
3	0	0	4	0	0	0	0	0	0	0	0	0	0	0	0	0	0	0	4	68	0	0	100.00	1.00	1.00	1.00	1.00
4	0	0	0	4	0	0	0	0	0	0	0	0	0	0	0	0	0	0	4	68	0	0	100.00	1.00	1.00	1.00	1.00
5	0	0	0	0	4	0	0	0	0	0	0	0	0	0	0	0	0	0	4	67	1	0	98.61	1.00	0.99	0.89	0.99
6	0	0	0	0	0	4	0	0	0	0	0	0	0	0	0	0	0	0	4	68	0	0	100.00	1.00	1.00	1.00	1.00
7	0	0	0	0	0	0	3	0	1	0	0	0	0	0	0	0	0	0	3	68	0	1	98.61	0.75	1.00	0.86	0.87
8	0	0	0	0	0	0	0	4	0	0	0	0	0	0	0	0	0	0	4	68	0	0	100.00	1.00	1.00	1.00	1.00
9	0	0	0	0	0	0	0	0	3	0	0	1	0	0	0	0	0	0	3	67	1	1	97.22	0.75	0.99	0.74	0.86
10	0	0	0	0	0	0	0	0	0	4	0	0	0	0	0	0	0	0	4	68	0	0	100.00	1.00	1.00	1.00	1.00
11	0	0	0	0	0	0	0	0	0	0	4	0	0	0	0	0	0	0	4	68	0	0	100.00	1.00	1.00	1.00	1.00
12	0	0	0	0	0	0	0	0	0	0	0	4	0	0	0	0	0	0	4	67	1	0	98.61	1.00	0.99	0.89	0.99
13	0	0	0	0	0	0	0	0	0	0	0	0	4	0	0	0	0	0	4	68	0	0	100.00	1.00	1.00	1.00	1.00
14	0	0	0	0	0	0	0	0	0	0	0	0	0	4	0	0	0	0	4	68	0	0	100.00	1.00	1.00	1.00	1.00
15	0	0	0	0	0	0	0	0	0	0	0	0	0	0	4	0	0	0	4	68	0	0	100.00	1.00	1.00	1.00	1.00
16	0	0	0	0	0	0	0	0	0	0	0	0	0	0	0	4	0	0	4	68	0	0	100.00	1.00	1.00	1.00	1.00
17	0	0	0	0	0	0	0	0	0	0	0	0	0	0	0	0	4	0	4	68	0	0	100.00	1.00	1.00	1.00	1.00
18	0	0	0	0	1	0	0	0	0	0	0	0	0	0	0	0	0	3	3	68	0	1	98.61	0.75	1.00	0.86	0.87

Table S22. Confusion matrixes of the calibration set and validation set of VIP-MIR-RF

Calibration set																												
**Class**	**1**	**2**	**3**	**4**	**5**	**6**	**7**	**8**	**9**	**10**	**11**	**12**	**13**	**14**	**15**	**16**	**17**	**18**	**TP**	**TN**	**FP**	**FN**	**ACC**	**SE**	**SP**	**MCC**	**EFF**
1	6	0	0	0	0	0	0	0	0	0	0	0	0	0	0	0	0	0	6	102	0	0	100.00	1.00	1.00	1.00	1.00
2	0	6	0	0	0	0	0	0	0	0	0	0	0	0	0	0	0	0	6	102	0	0	100.00	1.00	1.00	1.00	1.00
3	0	0	6	0	0	0	0	0	0	0	0	0	0	0	0	0	0	0	6	102	0	0	100.00	1.00	1.00	1.00	1.00
4	0	0	0	6	0	0	0	0	0	0	0	0	0	0	0	0	0	0	6	102	0	0	100.00	1.00	1.00	1.00	1.00
5	0	0	0	0	6	0	0	0	0	0	0	0	0	0	0	0	0	0	6	102	0	0	100.00	1.00	1.00	1.00	1.00
6	0	0	0	0	0	6	0	0	0	0	0	0	0	0	0	0	0	0	6	102	0	0	100.00	1.00	1.00	1.00	1.00
7	0	0	0	0	0	0	6	0	0	0	0	0	0	0	0	0	0	0	6	102	0	0	100.00	1.00	1.00	1.00	1.00
8	0	0	0	0	0	0	0	6	0	0	0	0	0	0	0	0	0	0	6	102	0	0	100.00	1.00	1.00	1.00	1.00
9	0	0	0	0	0	0	0	0	6	0	0	0	0	0	0	0	0	0	6	102	0	0	100.00	1.00	1.00	1.00	1.00
10	0	0	0	0	0	0	0	0	0	6	0	0	0	0	0	0	0	0	6	102	0	0	100.00	1.00	1.00	1.00	1.00
11	0	0	0	0	0	0	0	0	0	0	6	0	0	0	0	0	0	0	6	102	0	0	100.00	1.00	1.00	1.00	1.00
12	0	0	0	0	0	0	0	0	0	0	0	6	0	0	0	0	0	0	6	102	0	0	100.00	1.00	1.00	1.00	1.00
13	0	0	0	0	0	0	0	0	0	0	0	0	6	0	0	0	0	0	6	102	0	0	100.00	1.00	1.00	1.00	1.00
14	0	0	0	0	0	0	0	0	0	0	0	0	0	6	0	0	0	0	6	102	0	0	100.00	1.00	1.00	1.00	1.00
15	0	0	0	0	0	0	0	0	0	0	0	0	0	0	6	0	0	0	6	102	0	0	100.00	1.00	1.00	1.00	1.00
16	0	0	0	0	0	0	0	0	0	0	0	0	0	0	0	6	0	0	6	102	0	0	100.00	1.00	1.00	1.00	1.00
17	0	0	0	0	0	0	0	0	0	0	0	0	0	0	0	0	6	0	6	102	0	0	100.00	1.00	1.00	1.00	1.00
18	0	0	0	0	0	0	0	0	0	0	0	0	0	0	0	0	0	6	6	102	0	0	100.00	1.00	1.00	1.00	1.00

Validation set																												
**Class**	**1**	**2**	**3**	**4**	**5**	**6**	**7**	**8**	**9**	**10**	**11**	**12**	**13**	**14**	**15**	**16**	**17**	**18**	**TP**	**TN**	**FP**	**FN**	**ACC**	**SE**	**SP**	**MCC**	**EFF**
1	4	0	0	0	0	0	0	0	0	0	0	0	0	0	0	0	0	0	4	68	0	0	100.00	1.00	1.00	1.00	1.00
2	0	4	0	0	0	0	0	0	0	0	0	0	0	0	0	0	0	0	4	68	0	0	100.00	1.00	1.00	1.00	1.00
3	0	0	4	0	0	0	0	0	0	0	0	0	0	0	0	0	0	0	4	68	0	0	100.00	1.00	1.00	1.00	1.00
4	0	0	0	4	0	0	0	0	0	0	0	0	0	0	0	0	0	0	4	68	0	0	100.00	1.00	1.00	1.00	1.00
5	0	0	0	0	4	0	0	0	0	0	0	0	0	0	0	0	0	0	4	68	0	0	100.00	1.00	1.00	1.00	1.00
6	0	0	0	0	0	4	0	0	0	0	0	0	0	0	0	0	0	0	4	68	0	0	100.00	1.00	1.00	1.00	1.00
7	0	0	0	0	0	0	4	0	0	0	0	0	0	0	0	0	0	0	4	68	0	0	100.00	1.00	1.00	1.00	1.00
8	0	0	0	0	0	0	0	3	1	0	0	0	0	0	0	0	0	0	3	68	0	1	98.61	0.75	1.00	0.86	0.87
9	0	0	0	0	0	0	0	0	4	0	0	0	0	0	0	0	0	0	4	67	1	0	98.61	1.00	0.99	0.89	0.99
10	0	0	0	0	0	0	0	0	0	4	0	0	0	0	0	0	0	0	4	68	0	0	100.00	1.00	1.00	1.00	1.00
11	0	0	0	0	0	0	0	0	0	0	4	0	0	0	0	0	0	0	4	67	1	0	98.61	1.00	0.99	0.89	0.99
12	0	0	0	0	0	0	0	0	0	0	1	3	0	0	0	0	0	0	3	68	0	1	98.61	0.75	1.00	0.86	0.87
13	0	0	0	0	0	0	0	0	0	0	0	0	4	0	0	0	0	0	4	68	0	0	100.00	1.00	1.00	1.00	1.00
14	0	0	0	0	0	0	0	0	0	0	0	0	0	4	0	0	0	0	4	68	0	0	100.00	1.00	1.00	1.00	1.00
15	0	0	0	0	0	0	0	0	0	0	0	0	0	0	4	0	0	0	4	68	0	0	100.00	1.00	1.00	1.00	1.00
16	0	0	0	0	0	0	0	0	0	0	0	0	0	0	0	4	0	0	4	68	0	0	100.00	1.00	1.00	1.00	1.00
17	0	0	0	0	0	0	0	0	0	0	0	0	0	0	0	0	4	0	4	68	0	0	100.00	1.00	1.00	1.00	1.00
18	0	0	0	0	0	0	0	0	0	0	0	0	0	0	0	0	0	4	4	68	0	0	100.00	1.00	1.00	1.00	1.00

Table S23. Confusion matrixes of the calibration set and validation set of Bor-MIR-RF

Calibration set																											
**Class**	**1**	**2**	**3**	**4**	**5**	**6**	**7**	**8**	**9**	**10**	**11**	**12**	**13**	**14**	**15**	**16**	**17**	**18**	**TP**	**TN**	**FP**	**FN**	**ACC**	**SE**	**SP**	**MCC**	**EFF**
1	6	0	0	0	0	0	0	0	0	0	0	0	0	0	0	0	0	0	6	102	0	0	100.00	1.00	1.00	1.00	1.00
2	0	6	0	0	0	0	0	0	0	0	0	0	0	0	0	0	0	0	6	102	0	0	100.00	1.00	1.00	1.00	1.00
3	0	0	6	0	0	0	0	0	0	0	0	0	0	0	0	0	0	0	6	102	0	0	100.00	1.00	1.00	1.00	1.00
4	0	0	0	6	0	0	0	0	0	0	0	0	0	0	0	0	0	0	6	102	0	0	100.00	1.00	1.00	1.00	1.00
5	0	0	0	0	6	0	0	0	0	0	0	0	0	0	0	0	0	0	6	102	0	0	100.00	1.00	1.00	1.00	1.00
6	0	0	0	0	0	6	0	0	0	0	0	0	0	0	0	0	0	0	6	102	0	0	100.00	1.00	1.00	1.00	1.00
7	0	0	0	0	0	0	6	0	0	0	0	0	0	0	0	0	0	0	6	102	0	0	100.00	1.00	1.00	1.00	1.00
8	0	0	0	0	0	0	0	6	0	0	0	0	0	0	0	0	0	0	6	102	0	0	100.00	1.00	1.00	1.00	1.00
9	0	0	0	0	0	0	0	0	6	0	0	0	0	0	0	0	0	0	6	102	0	0	100.00	1.00	1.00	1.00	1.00
10	0	0	0	0	0	0	0	0	0	6	0	0	0	0	0	0	0	0	6	102	0	0	100.00	1.00	1.00	1.00	1.00
11	0	0	0	0	0	0	0	0	0	0	6	0	0	0	0	0	0	0	6	102	0	0	100.00	1.00	1.00	1.00	1.00
12	0	0	0	0	0	0	0	0	0	0	0	6	0	0	0	0	0	0	6	102	0	0	100.00	1.00	1.00	1.00	1.00
13	0	0	0	0	0	0	0	0	0	0	0	0	6	0	0	0	0	0	6	102	0	0	100.00	1.00	1.00	1.00	1.00
14	0	0	0	0	0	0	0	0	0	0	0	0	0	6	0	0	0	0	6	102	0	0	100.00	1.00	1.00	1.00	1.00
15	0	0	0	0	0	0	0	0	0	0	0	0	0	0	6	0	0	0	6	102	0	0	100.00	1.00	1.00	1.00	1.00
16	0	0	0	0	0	0	0	0	0	0	0	0	0	0	0	6	0	0	6	102	0	0	100.00	1.00	1.00	1.00	1.00
17	0	0	0	0	0	0	0	0	0	0	0	0	0	0	0	0	6	0	6	102	0	0	100.00	1.00	1.00	1.00	1.00
18	0	0	0	0	0	0	0	0	0	0	0	0	0	0	0	0	0	6	6	102	0	0	100.00	1.00	1.00	1.00	1.00

Validation set																												
**Class**	**1**	**2**	**3**	**4**	**5**	**6**	**7**	**8**	**9**	**10**	**11**	**12**	**13**	**14**	**15**	**16**	**17**	**18**	**TP**	**TN**	**FP**	**FN**	**ACC**	**SE**	**SP**	**MCC**	**EFF**
1	4	0	0	0	0	0	0	0	0	0	0	0	0	0	0	0	0	0	4	68	0	0	100.00	1.00	1.00	1.00	1.00
2	0	4	0	0	0	0	0	0	0	0	0	0	0	0	0	0	0	0	4	68	0	0	100.00	1.00	1.00	1.00	1.00
3	0	0	4	0	0	0	0	0	0	0	0	0	0	0	0	0	0	0	4	68	0	0	100.00	1.00	1.00	1.00	1.00
4	0	0	0	4	0	0	0	0	0	0	0	0	0	0	0	0	0	0	4	68	0	0	100.00	1.00	1.00	1.00	1.00
5	0	0	0	0	4	0	0	0	0	0	0	0	0	0	0	0	0	0	4	68	0	0	100.00	1.00	1.00	1.00	1.00
6	0	0	0	0	0	4	0	0	0	0	0	0	0	0	0	0	0	0	4	68	0	0	100.00	1.00	1.00	1.00	1.00
7	0	0	0	0	0	0	4	0	0	0	0	0	0	0	0	0	0	0	4	68	0	0	100.00	1.00	1.00	1.00	1.00
8	0	0	0	0	0	0	0	3	1	0	0	0	0	0	0	0	0	0	3	68	0	1	98.61	0.75	1.00	0.86	0.87
9	0	0	0	0	0	0	0	0	4	0	0	0	0	0	0	0	0	0	4	67	1	0	98.61	1.00	0.99	0.89	0.99
10	0	0	0	0	0	0	0	0	0	4	0	0	0	0	0	0	0	0	4	68	0	0	100.00	1.00	1.00	1.00	1.00
11	0	0	0	0	0	0	0	0	0	0	4	0	0	0	0	0	0	0	4	67	1	0	98.61	1.00	0.99	0.89	0.99
12	0	0	0	0	0	0	0	0	0	0	1	3	0	0	0	0	0	0	3	68	0	1	98.61	0.75	1.00	0.86	0.87
13	0	0	0	0	0	0	0	0	0	0	0	0	4	0	0	0	0	0	4	68	0	0	100.00	1.00	1.00	1.00	1.00
14	0	0	0	0	0	0	0	0	0	0	0	0	0	4	0	0	0	0	4	68	0	0	100.00	1.00	1.00	1.00	1.00
15	0	0	0	0	0	0	0	0	0	0	0	0	0	0	4	0	0	0	4	67	1	0	98.61	1.00	0.99	0.89	0.99
16	0	0	0	0	0	0	0	0	0	0	0	0	0	0	1	3	0	0	3	68	0	1	98.61	0.75	1.00	0.86	0.87
17	0	0	0	0	0	0	0	0	0	0	0	0	0	0	0	0	4	0	4	68	0	0	100.00	1.00	1.00	1.00	1.00
18	0	0	0	0	0	0	0	0	0	0	0	0	0	0	0	0	0	4	4	68	0	0	100.00	1.00	1.00	1.00	1.00

Table S24. Confusion matrixes of the calibration set and validation set of GARF-MIR-RF

Calibration set																												
**Class**	**1**	**2**	**3**	**4**	**5**	**6**	**7**	**8**	**9**	**10**	**11**	**12**	**13**	**14**	**15**	**16**	**17**	**18**	**TP**	**TN**	**FP**	**FN**	**ACC**	**SE**	**SP**	**MCC**	**EFF**
1	6	0	0	0	0	0	0	0	0	0	0	0	0	0	0	0	0	0	6	102	0	0	100.00	1.00	1.00	1.00	1.00
2	0	6	0	0	0	0	0	0	0	0	0	0	0	0	0	0	0	0	6	102	0	0	100.00	1.00	1.00	1.00	1.00
3	0	0	6	0	0	0	0	0	0	0	0	0	0	0	0	0	0	0	6	102	0	0	100.00	1.00	1.00	1.00	1.00
4	0	0	0	6	0	0	0	0	0	0	0	0	0	0	0	0	0	0	6	102	0	0	100.00	1.00	1.00	1.00	1.00
5	0	0	0	0	6	0	0	0	0	0	0	0	0	0	0	0	0	0	6	102	0	0	100.00	1.00	1.00	1.00	1.00
6	0	0	0	0	0	6	0	0	0	0	0	0	0	0	0	0	0	0	6	102	0	0	100.00	1.00	1.00	1.00	1.00
7	0	0	0	0	0	0	6	0	0	0	0	0	0	0	0	0	0	0	6	102	0	0	100.00	1.00	1.00	1.00	1.00
8	0	0	0	0	0	0	0	6	0	0	0	0	0	0	0	0	0	0	6	102	0	0	100.00	1.00	1.00	1.00	1.00
9	0	0	0	0	0	0	0	0	6	0	0	0	0	0	0	0	0	0	6	102	0	0	100.00	1.00	1.00	1.00	1.00
10	0	0	0	0	0	0	0	0	0	6	0	0	0	0	0	0	0	0	6	102	0	0	100.00	1.00	1.00	1.00	1.00
11	0	0	0	0	0	0	0	0	0	0	6	0	0	0	0	0	0	0	6	102	0	0	100.00	1.00	1.00	1.00	1.00
12	0	0	0	0	0	0	0	0	0	0	0	6	0	0	0	0	0	0	6	102	0	0	100.00	1.00	1.00	1.00	1.00
13	0	0	0	0	0	0	0	0	0	0	0	0	6	0	0	0	0	0	6	102	0	0	100.00	1.00	1.00	1.00	1.00
14	0	0	0	0	0	0	0	0	0	0	0	0	0	6	0	0	0	0	6	102	0	0	100.00	1.00	1.00	1.00	1.00
15	0	0	0	0	0	0	0	0	0	0	0	0	0	0	6	0	0	0	6	102	0	0	100.00	1.00	1.00	1.00	1.00
16	0	0	0	0	0	0	0	0	0	0	0	0	0	0	0	6	0	0	6	102	0	0	100.00	1.00	1.00	1.00	1.00
17	0	0	0	0	0	0	0	0	0	0	0	0	0	0	0	0	6	0	6	102	0	0	100.00	1.00	1.00	1.00	1.00
18	0	0	0	0	0	0	0	0	0	0	0	0	0	0	0	0	0	6	6	102	0	0	100.00	1.00	1.00	1.00	1.00

Validation set																												
**Class**	**1**	**2**	**3**	**4**	**5**	**6**	**7**	**8**	**9**	**10**	**11**	**12**	**13**	**14**	**15**	**16**	**17**	**18**	**TP**	**TN**	**FP**	**FN**	**ACC**	**SE**	**SP**	**MCC**	**EFF**
1	4	0	0	0	0	0	0	0	0	0	0	0	0	0	0	0	0	0	4	68	0	0	100.00	1.00	1.00	1.00	1.00
2	0	4	0	0	0	0	0	0	0	0	0	0	0	0	0	0	0	0	4	68	0	0	100.00	1.00	1.00	1.00	1.00
3	0	0	4	0	0	0	0	0	0	0	0	0	0	0	0	0	0	0	4	68	0	0	100.00	1.00	1.00	1.00	1.00
4	0	0	0	4	0	0	0	0	0	0	0	0	0	0	0	0	0	0	4	68	0	0	100.00	1.00	1.00	1.00	1.00
5	0	0	0	0	4	0	0	0	0	0	0	0	0	0	0	0	0	0	4	68	0	0	100.00	1.00	1.00	1.00	1.00
6	0	0	0	0	0	4	0	0	0	0	0	0	0	0	0	0	0	0	4	68	0	0	100.00	1.00	1.00	1.00	1.00
7	0	0	0	0	0	0	4	0	0	0	0	0	0	0	0	0	0	0	4	68	0	0	100.00	1.00	1.00	1.00	1.00
8	0	0	0	0	0	0	0	3	1	0	0	0	0	0	0	0	0	0	3	68	0	1	98.61	0.75	1.00	0.86	0.87
9	0	0	0	0	0	0	0	0	4	0	0	0	0	0	0	0	0	0	4	67	1	0	98.61	1.00	0.99	0.89	0.99
10	0	0	0	0	0	0	0	0	0	4	0	0	0	0	0	0	0	0	4	67	1	0	98.61	1.00	0.99	0.89	0.99
11	0	0	0	0	0	0	0	0	0	0	4	0	0	0	0	0	0	0	4	67	1	0	98.61	1.00	0.99	0.89	0.99
12	0	0	0	0	0	0	0	0	0	0	1	3	0	0	0	0	0	0	3	68	0	1	98.61	0.75	1.00	0.86	0.87
13	0	0	0	0	0	0	0	0	0	0	0	0	4	0	0	0	0	0	4	68	0	0	100.00	1.00	1.00	1.00	1.00
14	0	0	0	0	0	0	0	0	0	0	0	0	0	4	0	0	0	0	4	68	0	0	100.00	1.00	1.00	1.00	1.00
15	0	0	0	0	0	0	0	0	0	0	0	0	0	0	4	0	0	0	4	68	0	0	100.00	1.00	1.00	1.00	1.00
16	0	0	0	0	0	0	0	0	0	1	0	0	0	0	0	3	0	0	3	68	0	1	98.61	0.75	1.00	0.86	0.87
17	0	0	0	0	0	0	0	0	0	0	0	0	0	0	0	0	4	0	4	68	0	0	100.00	1.00	1.00	1.00	1.00
18	0	0	0	0	0	0	0	0	0	0	0	0	0	0	0	0	0	4	4	68	0	0	100.00	1.00	1.00	1.00	1.00

Table S25. Confusion matrixes of the calibration set and validation set of GASVM-MIR-RF

Calibration set																												
**Class**	**1**	**2**	**3**	**4**	**5**	**6**	**7**	**8**	**9**	**10**	**11**	**12**	**13**	**14**	**15**	**16**	**17**	**18**	**TP**	**TN**	**FP**	**FN**	**ACC**	**SE**	**SP**	**MCC**	**EFF**
1	6	0	0	0	0	0	0	0	0	0	0	0	0	0	0	0	0	0	6	102	0	0	100.00	1.00	1.00	1.00	1.00
2	0	6	0	0	0	0	0	0	0	0	0	0	0	0	0	0	0	0	6	102	0	0	100.00	1.00	1.00	1.00	1.00
3	0	0	6	0	0	0	0	0	0	0	0	0	0	0	0	0	0	0	6	102	0	0	100.00	1.00	1.00	1.00	1.00
4	0	0	0	6	0	0	0	0	0	0	0	0	0	0	0	0	0	0	6	102	0	0	100.00	1.00	1.00	1.00	1.00
5	0	0	0	0	6	0	0	0	0	0	0	0	0	0	0	0	0	0	6	102	0	0	100.00	1.00	1.00	1.00	1.00
6	0	0	0	0	0	6	0	0	0	0	0	0	0	0	0	0	0	0	6	102	0	0	100.00	1.00	1.00	1.00	1.00
7	0	0	0	0	0	0	6	0	0	0	0	0	0	0	0	0	0	0	6	102	0	0	100.00	1.00	1.00	1.00	1.00
8	0	0	0	0	0	0	0	6	0	0	0	0	0	0	0	0	0	0	6	102	0	0	100.00	1.00	1.00	1.00	1.00
9	0	0	0	0	0	0	0	0	6	0	0	0	0	0	0	0	0	0	6	102	0	0	100.00	1.00	1.00	1.00	1.00
10	0	0	0	0	0	0	0	0	0	6	0	0	0	0	0	0	0	0	6	102	0	0	100.00	1.00	1.00	1.00	1.00
11	0	0	0	0	0	0	0	0	0	0	6	0	0	0	0	0	0	0	6	102	0	0	100.00	1.00	1.00	1.00	1.00
12	0	0	0	0	0	0	0	0	0	0	0	6	0	0	0	0	0	0	6	102	0	0	100.00	1.00	1.00	1.00	1.00
13	0	0	0	0	0	0	0	0	0	0	0	0	6	0	0	0	0	0	6	102	0	0	100.00	1.00	1.00	1.00	1.00
14	0	0	0	0	0	0	0	0	0	0	0	0	0	6	0	0	0	0	6	102	0	0	100.00	1.00	1.00	1.00	1.00
15	0	0	0	0	0	0	0	0	0	0	0	0	0	0	6	0	0	0	6	102	0	0	100.00	1.00	1.00	1.00	1.00
16	0	0	0	0	0	0	0	0	0	0	0	0	0	0	0	6	0	0	6	102	0	0	100.00	1.00	1.00	1.00	1.00
17	0	0	0	0	0	0	0	0	0	0	0	0	0	0	0	0	6	0	6	102	0	0	100.00	1.00	1.00	1.00	1.00
18	0	0	0	0	0	0	0	0	0	0	0	0	0	0	0	0	0	6	6	102	0	0	100.00	1.00	1.00	1.00	1.00

Validation set																												
**Class**	**1**	**2**	**3**	**4**	**5**	**6**	**7**	**8**	**9**	**10**	**11**	**12**	**13**	**14**	**15**	**16**	**17**	**18**	**TP**	**TN**	**FP**	**FN**	**ACC**	**SE**	**SP**	**MCC**	**EFF**
1	4	0	0	0	0	0	0	0	0	0	0	0	0	0	0	0	0	0	4	68	0	0	100.00	1.00	1.00	1.00	1.00
2	0	4	0	0	0	0	0	0	0	0	0	0	0	0	0	0	0	0	4	68	0	0	100.00	1.00	1.00	1.00	1.00
3	0	0	4	0	0	0	0	0	0	0	0	0	0	0	0	0	0	0	4	68	0	0	100.00	1.00	1.00	1.00	1.00
4	0	0	0	4	0	0	0	0	0	0	0	0	0	0	0	0	0	0	4	68	0	0	100.00	1.00	1.00	1.00	1.00
5	0	0	0	0	4	0	0	0	0	0	0	0	0	0	0	0	0	0	4	68	0	0	100.00	1.00	1.00	1.00	1.00
6	0	0	0	0	0	4	0	0	0	0	0	0	0	0	0	0	0	0	4	68	0	0	100.00	1.00	1.00	1.00	1.00
7	0	0	0	0	0	0	4	0	0	0	0	0	0	0	0	0	0	0	4	68	0	0	100.00	1.00	1.00	1.00	1.00
8	0	0	0	0	0	0	0	3	1	0	0	0	0	0	0	0	0	0	3	68	0	1	98.61	0.75	1.00	0.86	0.87
9	0	0	0	0	0	0	0	0	4	0	0	0	0	0	0	0	0	0	4	67	1	0	98.61	1.00	0.99	0.89	0.99
10	0	0	0	0	0	0	0	0	0	4	0	0	0	0	0	0	0	0	4	67	1	0	98.61	1.00	0.99	0.89	0.99
11	0	0	0	0	0	0	0	0	0	0	4	0	0	0	0	0	0	0	4	67	1	0	98.61	1.00	0.99	0.89	0.99
12	0	0	0	0	0	0	0	0	0	0	1	3	0	0	0	0	0	0	3	68	0	1	98.61	0.75	1.00	0.86	0.87
13	0	0	0	0	0	0	0	0	0	0	0	0	4	0	0	0	0	0	4	68	0	0	100.00	1.00	1.00	1.00	1.00
14	0	0	0	0	0	0	0	0	0	0	0	0	0	4	0	0	0	0	4	68	0	0	100.00	1.00	1.00	1.00	1.00
15	0	0	0	0	0	0	0	0	0	0	0	0	0	0	4	0	0	0	4	67	1	0	98.61	1.00	0.99	0.89	0.99
16	0	0	0	0	0	0	0	0	0	1	0	0	0	0	1	2	0	0	2	68	0	2	97.22	0.50	1.00	0.70	0.71
17	0	0	0	0	0	0	0	0	0	0	0	0	0	0	0	0	4	0	4	68	0	0	100.00	1.00	1.00	1.00	1.00
18	0	0	0	0	0	0	0	0	0	0	0	0	0	0	0	0	0	4	4	68	0	0	100.00	1.00	1.00	1.00	1.00

Table S26. Confusion matrixes of the calibration set and validation set of Ven-MIR-RF

Calibration set																											
**Class**	**1**	**2**	**3**	**4**	**5**	**6**	**7**	**8**	**9**	**10**	**11**	**12**	**13**	**14**	**15**	**16**	**17**	**18**	**TP**	**TN**	**FP**	**FN**	**ACC**	**SE**	**SP**	**MCC**	**EFF**
1	6	0	0	0	0	0	0	0	0	0	0	0	0	0	0	0	0	0	6	102	0	0	100.00	1.00	1.00	1.00	1.00
2	0	6	0	0	0	0	0	0	0	0	0	0	0	0	0	0	0	0	6	102	0	0	100.00	1.00	1.00	1.00	1.00
3	0	0	6	0	0	0	0	0	0	0	0	0	0	0	0	0	0	0	6	102	0	0	100.00	1.00	1.00	1.00	1.00
4	0	0	0	6	0	0	0	0	0	0	0	0	0	0	0	0	0	0	6	102	0	0	100.00	1.00	1.00	1.00	1.00
5	0	0	0	0	6	0	0	0	0	0	0	0	0	0	0	0	0	0	6	102	0	0	100.00	1.00	1.00	1.00	1.00
6	0	0	0	0	0	6	0	0	0	0	0	0	0	0	0	0	0	0	6	102	0	0	100.00	1.00	1.00	1.00	1.00
7	0	0	0	0	0	0	6	0	0	0	0	0	0	0	0	0	0	0	6	102	0	0	100.00	1.00	1.00	1.00	1.00
8	0	0	0	0	0	0	0	6	0	0	0	0	0	0	0	0	0	0	6	102	0	0	100.00	1.00	1.00	1.00	1.00
9	0	0	0	0	0	0	0	0	6	0	0	0	0	0	0	0	0	0	6	102	0	0	100.00	1.00	1.00	1.00	1.00
10	0	0	0	0	0	0	0	0	0	6	0	0	0	0	0	0	0	0	6	102	0	0	100.00	1.00	1.00	1.00	1.00
11	0	0	0	0	0	0	0	0	0	0	6	0	0	0	0	0	0	0	6	102	0	0	100.00	1.00	1.00	1.00	1.00
12	0	0	0	0	0	0	0	0	0	0	0	6	0	0	0	0	0	0	6	102	0	0	100.00	1.00	1.00	1.00	1.00
13	0	0	0	0	0	0	0	0	0	0	0	0	6	0	0	0	0	0	6	102	0	0	100.00	1.00	1.00	1.00	1.00
14	0	0	0	0	0	0	0	0	0	0	0	0	0	6	0	0	0	0	6	102	0	0	100.00	1.00	1.00	1.00	1.00
15	0	0	0	0	0	0	0	0	0	0	0	0	0	0	6	0	0	0	6	102	0	0	100.00	1.00	1.00	1.00	1.00
16	0	0	0	0	0	0	0	0	0	0	0	0	0	0	0	6	0	0	6	102	0	0	100.00	1.00	1.00	1.00	1.00
17	0	0	0	0	0	0	0	0	0	0	0	0	0	0	0	0	6	0	6	102	0	0	100.00	1.00	1.00	1.00	1.00
18	0	0	0	0	0	0	0	0	0	0	0	0	0	0	0	0	0	6	6	102	0	0	100.00	1.00	1.00	1.00	1.00

Validation set																												
**Class**	**1**	**2**	**3**	**4**	**5**	**6**	**7**	**8**	**9**	**10**	**11**	**12**	**13**	**14**	**15**	**16**	**17**	**18**	**TP**	**TN**	**FP**	**FN**	**ACC**	**SE**	**SP**	**MCC**	**EFF**
1	4	0	0	0	0	0	0	0	0	0	0	0	0	0	0	0	0	0	4	68	0	0	100.00	1.00	1.00	1.00	1.00
2	0	4	0	0	0	0	0	0	0	0	0	0	0	0	0	0	0	0	4	68	0	0	100.00	1.00	1.00	1.00	1.00
3	0	0	4	0	0	0	0	0	0	0	0	0	0	0	0	0	0	0	4	68	0	0	100.00	1.00	1.00	1.00	1.00
4	0	0	0	4	0	0	0	0	0	0	0	0	0	0	0	0	0	0	4	68	0	0	100.00	1.00	1.00	1.00	1.00
5	0	0	0	0	4	0	0	0	0	0	0	0	0	0	0	0	0	0	4	68	0	0	100.00	1.00	1.00	1.00	1.00
6	0	0	0	0	0	4	0	0	0	0	0	0	0	0	0	0	0	0	4	68	0	0	100.00	1.00	1.00	1.00	1.00
7	0	0	0	0	0	0	4	0	0	0	0	0	0	0	0	0	0	0	4	68	0	0	100.00	1.00	1.00	1.00	1.00
8	0	0	0	0	0	0	0	4	0	0	0	0	0	0	0	0	0	0	4	68	0	0	100.00	1.00	1.00	1.00	1.00
9	0	0	0	0	0	0	0	0	4	0	0	0	0	0	0	0	0	0	4	68	0	0	100.00	1.00	1.00	1.00	1.00
10	0	0	0	0	0	0	0	0	0	4	0	0	0	0	0	0	0	0	4	68	0	0	100.00	1.00	1.00	1.00	1.00
11	0	0	0	0	0	0	0	0	0	0	4	0	0	0	0	0	0	0	4	67	1	0	98.61	1.00	0.99	0.89	0.99
12	0	0	0	0	0	0	0	0	0	0	1	3	0	0	0	0	0	0	3	68	0	1	98.61	0.75	1.00	0.86	0.87
13	0	0	0	0	0	0	0	0	0	0	0	0	4	0	0	0	0	0	4	68	0	0	100.00	1.00	1.00	1.00	1.00
14	0	0	0	0	0	0	0	0	0	0	0	0	0	4	0	0	0	0	4	68	0	0	100.00	1.00	1.00	1.00	1.00
15	0	0	0	0	0	0	0	0	0	0	0	0	0	0	4	0	0	0	4	68	0	0	100.00	1.00	1.00	1.00	1.00
16	0	0	0	0	0	0	0	0	0	0	0	0	0	0	0	4	0	0	4	68	0	0	100.00	1.00	1.00	1.00	1.00
17	0	0	0	0	0	0	0	0	0	0	0	0	0	0	0	0	4	0	4	68	0	0	100.00	1.00	1.00	1.00	1.00
18	0	0	0	0	0	0	0	0	0	0	0	0	0	0	0	0	0	4	4	68	0	0	100.00	1.00	1.00	1.00	1.00

Table S27. Confusion matrixes of the calibration set and validation set of VIP-MIR-SVM

Calibration set																											
**Class**	**1**	**2**	**3**	**4**	**5**	**6**	**7**	**8**	**9**	**10**	**11**	**12**	**13**	**14**	**15**	**16**	**17**	**18**	**TP**	**TN**	**FP**	**FN**	**ACC**	**SE**	**SP**	**MCC**	**EFF**
1	6	0	0	0	0	0	0	0	0	0	0	0	0	0	0	0	0	0	6	102	0	0	100.00	1.00	1.00	1.00	1.00
2	0	6	0	0	0	0	0	0	0	0	0	0	0	0	0	0	0	0	6	102	0	0	100.00	1.00	1.00	1.00	1.00
3	0	0	6	0	0	0	0	0	0	0	0	0	0	0	0	0	0	0	6	102	0	0	100.00	1.00	1.00	1.00	1.00
4	0	0	0	6	0	0	0	0	0	0	0	0	0	0	0	0	0	0	6	102	0	0	100.00	1.00	1.00	1.00	1.00
5	0	0	0	0	6	0	0	0	0	0	0	0	0	0	0	0	0	0	6	102	0	0	100.00	1.00	1.00	1.00	1.00
6	0	0	0	0	0	6	0	0	0	0	0	0	0	0	0	0	0	0	6	102	0	0	100.00	1.00	1.00	1.00	1.00
7	0	0	0	0	0	0	6	0	0	0	0	0	0	0	0	0	0	0	6	102	0	0	100.00	1.00	1.00	1.00	1.00
8	0	0	0	0	0	0	0	6	0	0	0	0	0	0	0	0	0	0	6	102	0	0	100.00	1.00	1.00	1.00	1.00
9	0	0	0	0	0	0	0	0	6	0	0	0	0	0	0	0	0	0	6	102	0	0	100.00	1.00	1.00	1.00	1.00
10	0	0	0	0	0	0	0	0	0	6	0	0	0	0	0	0	0	0	6	102	0	0	100.00	1.00	1.00	1.00	1.00
11	0	0	0	0	0	0	0	0	0	0	6	0	0	0	0	0	0	0	6	102	0	0	100.00	1.00	1.00	1.00	1.00
12	0	0	0	0	0	0	0	0	0	0	0	6	0	0	0	0	0	0	6	102	0	0	100.00	1.00	1.00	1.00	1.00
13	0	0	0	0	0	0	0	0	0	0	0	0	6	0	0	0	0	0	6	102	0	0	100.00	1.00	1.00	1.00	1.00
14	0	0	0	0	0	0	0	0	0	0	0	0	0	6	0	0	0	0	6	102	0	0	100.00	1.00	1.00	1.00	1.00
15	0	0	0	0	0	0	0	0	0	0	0	0	0	0	6	0	0	0	6	102	0	0	100.00	1.00	1.00	1.00	1.00
16	0	0	0	0	0	0	0	0	0	0	0	0	0	0	0	6	0	0	6	102	0	0	100.00	1.00	1.00	1.00	1.00
17	0	0	0	0	0	0	0	0	0	0	0	0	0	0	0	0	6	0	6	102	0	0	100.00	1.00	1.00	1.00	1.00
18	0	0	0	0	0	0	0	0	0	0	0	0	0	0	0	0	0	6	6	102	0	0	100.00	1.00	1.00	1.00	1.00

Validation set																											
**Class**	**1**	**2**	**3**	**4**	**5**	**6**	**7**	**8**	**9**	**10**	**11**	**12**	**13**	**14**	**15**	**16**	**17**	**18**	**TP**	**TN**	**FP**	**FN**	**ACC**	**SE**	**SP**	**MCC**	**EFF**
1	4	0	0	0	0	0	0	0	0	0	0	0	0	0	0	0	0	0	4	68	0	0	100.00	1.00	1.00	1.00	1.00
2	0	4	0	0	0	0	0	0	0	0	0	0	0	0	0	0	0	0	4	68	0	0	100.00	1.00	1.00	1.00	1.00
3	0	0	4	0	0	0	0	0	0	0	0	0	0	0	0	0	0	0	4	68	0	0	100.00	1.00	1.00	1.00	1.00
4	0	0	0	4	0	0	0	0	0	0	0	0	0	0	0	0	0	0	4	68	0	0	100.00	1.00	1.00	1.00	1.00
5	0	0	0	0	4	0	0	0	0	0	0	0	0	0	0	0	0	0	4	68	0	0	100.00	1.00	1.00	1.00	1.00
6	0	0	0	0	0	4	0	0	0	0	0	0	0	0	0	0	0	0	4	68	0	0	100.00	1.00	1.00	1.00	1.00
7	0	0	0	0	0	0	4	0	0	0	0	0	0	0	0	0	0	0	4	68	0	0	100.00	1.00	1.00	1.00	1.00
8	0	0	0	0	0	0	0	4	0	0	0	0	0	0	0	0	0	0	4	68	0	0	100.00	1.00	1.00	1.00	1.00
9	0	0	0	0	0	0	0	0	4	0	0	0	0	0	0	0	0	0	4	68	0	0	100.00	1.00	1.00	1.00	1.00
10	0	0	0	0	0	0	0	0	0	4	0	0	0	0	0	0	0	0	4	68	0	0	100.00	1.00	1.00	1.00	1.00
11	0	0	0	0	0	0	0	0	0	0	4	0	0	0	0	0	0	0	4	68	0	0	100.00	1.00	1.00	1.00	1.00
12	0	0	0	0	0	0	0	0	0	0	0	4	0	0	0	0	0	0	4	68	0	0	100.00	1.00	1.00	1.00	1.00
13	0	0	0	0	0	0	0	0	0	0	0	0	4	0	0	0	0	0	4	68	0	0	100.00	1.00	1.00	1.00	1.00
14	0	0	0	0	0	0	0	0	0	0	0	0	0	4	0	0	0	0	4	68	0	0	100.00	1.00	1.00	1.00	1.00
15	0	0	0	0	0	0	0	0	0	0	0	0	0	0	4	0	0	0	4	68	0	0	100.00	1.00	1.00	1.00	1.00
16	0	0	0	0	0	0	0	0	0	0	0	0	0	0	0	4	0	0	4	68	0	0	100.00	1.00	1.00	1.00	1.00
17	0	0	0	0	0	0	0	0	0	0	0	0	0	0	0	0	4	0	4	68	0	0	100.00	1.00	1.00	1.00	1.00
18	0	0	0	0	0	0	0	0	0	0	0	0	0	0	0	0	0	4	4	68	0	0	100.00	1.00	1.00	1.00	1.00

Table S28. Confusion matrixes of the calibration set and validation set of Bor-MIR-SVM

Calibration set																											
**Class**	**1**	**2**	**3**	**4**	**5**	**6**	**7**	**8**	**9**	**10**	**11**	**12**	**13**	**14**	**15**	**16**	**17**	**18**	**TP**	**TN**	**FP**	**FN**	**ACC**	**SE**	**SP**	**MCC**	**EFF**
1	6	0	0	0	0	0	0	0	0	0	0	0	0	0	0	0	0	0	6	102	0	0	100.00	1.00	1.00	1.00	1.00
2	0	6	0	0	0	0	0	0	0	0	0	0	0	0	0	0	0	0	6	102	0	0	100.00	1.00	1.00	1.00	1.00
3	0	0	6	0	0	0	0	0	0	0	0	0	0	0	0	0	0	0	6	102	0	0	100.00	1.00	1.00	1.00	1.00
4	0	0	0	6	0	0	0	0	0	0	0	0	0	0	0	0	0	0	6	102	0	0	100.00	1.00	1.00	1.00	1.00
5	0	0	0	0	6	0	0	0	0	0	0	0	0	0	0	0	0	0	6	102	0	0	100.00	1.00	1.00	1.00	1.00
6	0	0	0	0	0	6	0	0	0	0	0	0	0	0	0	0	0	0	6	102	0	0	100.00	1.00	1.00	1.00	1.00
7	0	0	0	0	0	0	6	0	0	0	0	0	0	0	0	0	0	0	6	102	0	0	100.00	1.00	1.00	1.00	1.00
8	0	0	0	0	0	0	0	6	0	0	0	0	0	0	0	0	0	0	6	102	0	0	100.00	1.00	1.00	1.00	1.00
9	0	0	0	0	0	0	0	0	6	0	0	0	0	0	0	0	0	0	6	102	0	0	100.00	1.00	1.00	1.00	1.00
10	0	0	0	0	0	0	0	0	0	6	0	0	0	0	0	0	0	0	6	102	0	0	100.00	1.00	1.00	1.00	1.00
11	0	0	0	0	0	0	0	0	0	0	6	0	0	0	0	0	0	0	6	102	0	0	100.00	1.00	1.00	1.00	1.00
12	0	0	0	0	0	0	0	0	0	0	0	6	0	0	0	0	0	0	6	102	0	0	100.00	1.00	1.00	1.00	1.00
13	0	0	0	0	0	0	0	0	0	0	0	0	6	0	0	0	0	0	6	102	0	0	100.00	1.00	1.00	1.00	1.00
14	0	0	0	0	0	0	0	0	0	0	0	0	0	6	0	0	0	0	6	102	0	0	100.00	1.00	1.00	1.00	1.00
15	0	0	0	0	0	0	0	0	0	0	0	0	0	0	6	0	0	0	6	102	0	0	100.00	1.00	1.00	1.00	1.00
16	0	0	0	0	0	0	0	0	0	0	0	0	0	0	0	6	0	0	6	102	0	0	100.00	1.00	1.00	1.00	1.00
17	0	0	0	0	0	0	0	0	0	0	0	0	0	0	0	0	6	0	6	102	0	0	100.00	1.00	1.00	1.00	1.00
18	0	0	0	0	0	0	0	0	0	0	0	0	0	0	0	0	0	6	6	102	0	0	100.00	1.00	1.00	1.00	1.00

Validation set																												
**Class**	**1**	**2**	**3**	**4**	**5**	**6**	**7**	**8**	**9**	**10**	**11**	**12**	**13**	**14**	**15**	**16**	**17**	**18**	**TP**	**TN**	**FP**	**FN**	**ACC**	**SE**	**SP**	**MCC**	**EFF**
1	4	0	0	0	0	0	0	0	0	0	0	0	0	0	0	0	0	0	4	68	0	0	100.00	1.00	1.00	1.00	1.00
2	0	4	0	0	0	0	0	0	0	0	0	0	0	0	0	0	0	0	4	68	0	0	100.00	1.00	1.00	1.00	1.00
3	0	0	4	0	0	0	0	0	0	0	0	0	0	0	0	0	0	0	4	68	0	0	100.00	1.00	1.00	1.00	1.00
4	0	0	0	4	0	0	0	0	0	0	0	0	0	0	0	0	0	0	4	68	0	0	100.00	1.00	1.00	1.00	1.00
5	0	0	0	0	4	0	0	0	0	0	0	0	0	0	0	0	0	0	4	68	0	0	100.00	1.00	1.00	1.00	1.00
6	0	0	0	0	0	4	0	0	0	0	0	0	0	0	0	0	0	0	4	68	0	0	100.00	1.00	1.00	1.00	1.00
7	0	0	0	0	0	0	4	0	0	0	0	0	0	0	0	0	0	0	4	68	0	0	100.00	1.00	1.00	1.00	1.00
8	0	0	0	0	0	0	0	4	0	0	0	0	0	0	0	0	0	0	4	68	0	0	100.00	1.00	1.00	1.00	1.00
9	0	0	0	0	0	0	0	0	4	0	0	0	0	0	0	0	0	0	4	68	0	0	100.00	1.00	1.00	1.00	1.00
10	0	0	0	0	0	0	0	0	0	4	0	0	0	0	0	0	0	0	4	68	0	0	100.00	1.00	1.00	1.00	1.00
11	0	0	0	0	0	0	0	0	0	0	4	0	0	0	0	0	0	0	4	68	0	0	100.00	1.00	1.00	1.00	1.00
12	0	0	0	0	0	0	0	0	0	0	0	4	0	0	0	0	0	0	4	68	0	0	100.00	1.00	1.00	1.00	1.00
13	0	0	0	0	0	0	0	0	0	0	0	0	4	0	0	0	0	0	4	68	0	0	100.00	1.00	1.00	1.00	1.00
14	0	0	0	0	0	0	0	0	0	0	0	0	0	4	0	0	0	0	4	68	0	0	100.00	1.00	1.00	1.00	1.00
15	0	0	0	0	0	0	0	0	0	0	0	0	0	0	4	0	0	0	4	68	0	0	100.00	1.00	1.00	1.00	1.00
16	0	0	0	0	0	0	0	0	0	0	0	0	0	0	0	4	0	0	4	68	0	0	100.00	1.00	1.00	1.00	1.00
17	0	0	0	0	0	0	0	0	0	0	0	0	0	0	0	0	4	0	4	68	0	0	100.00	1.00	1.00	1.00	1.00
18	0	0	0	0	0	0	0	0	0	0	0	0	0	0	0	0	0	4	4	68	0	0	100.00	1.00	1.00	1.00	1.00

Table S29. Confusion matrixes of the calibration set and validation set of GARF-MIR-SVM

Calibration set																												
**Class**	**1**	**2**	**3**	**4**	**5**	**6**	**7**	**8**	**9**	**10**	**11**	**12**	**13**	**14**	**15**	**16**	**17**	**18**	**TP**	**TN**	**FP**	**FN**	**ACC**	**SE**	**SP**	**MCC**	**EFF**
1	6	0	0	0	0	0	0	0	0	0	0	0	0	0	0	0	0	0	6	102	0	0	100.00	1.00	1.00	1.00	1.00
2	0	6	0	0	0	0	0	0	0	0	0	0	0	0	0	0	0	0	6	102	0	0	100.00	1.00	1.00	1.00	1.00
3	0	0	6	0	0	0	0	0	0	0	0	0	0	0	0	0	0	0	6	102	0	0	100.00	1.00	1.00	1.00	1.00
4	0	0	0	6	0	0	0	0	0	0	0	0	0	0	0	0	0	0	6	102	0	0	100.00	1.00	1.00	1.00	1.00
5	0	0	0	0	6	0	0	0	0	0	0	0	0	0	0	0	0	0	6	102	0	0	100.00	1.00	1.00	1.00	1.00
6	0	0	0	0	0	6	0	0	0	0	0	0	0	0	0	0	0	0	6	102	0	0	100.00	1.00	1.00	1.00	1.00
7	0	0	0	0	0	0	6	0	0	0	0	0	0	0	0	0	0	0	6	102	0	0	100.00	1.00	1.00	1.00	1.00
8	0	0	0	0	0	0	0	6	0	0	0	0	0	0	0	0	0	0	6	102	0	0	100.00	1.00	1.00	1.00	1.00
9	0	0	0	0	0	0	0	0	6	0	0	0	0	0	0	0	0	0	6	102	0	0	100.00	1.00	1.00	1.00	1.00
10	0	0	0	0	0	0	0	0	0	6	0	0	0	0	0	0	0	0	6	102	0	0	100.00	1.00	1.00	1.00	1.00
11	0	0	0	0	0	0	0	0	0	0	6	0	0	0	0	0	0	0	6	102	0	0	100.00	1.00	1.00	1.00	1.00
12	0	0	0	0	0	0	0	0	0	0	0	6	0	0	0	0	0	0	6	102	0	0	100.00	1.00	1.00	1.00	1.00
13	0	0	0	0	0	0	0	0	0	0	0	0	6	0	0	0	0	0	6	102	0	0	100.00	1.00	1.00	1.00	1.00
14	0	0	0	0	0	0	0	0	0	0	0	0	0	6	0	0	0	0	6	102	0	0	100.00	1.00	1.00	1.00	1.00
15	0	0	0	0	0	0	0	0	0	0	0	0	0	0	6	0	0	0	6	102	0	0	100.00	1.00	1.00	1.00	1.00
16	0	0	0	0	0	0	0	0	0	0	0	0	0	0	0	6	0	0	6	102	0	0	100.00	1.00	1.00	1.00	1.00
17	0	0	0	0	0	0	0	0	0	0	0	0	0	0	0	0	6	0	6	102	0	0	100.00	1.00	1.00	1.00	1.00
18	0	0	0	0	0	0	0	0	0	0	0	0	0	0	0	0	0	6	6	102	0	0	100.00	1.00	1.00	1.00	1.00

Validation set																											
**Class**	**1**	**2**	**3**	**4**	**5**	**6**	**7**	**8**	**9**	**10**	**11**	**12**	**13**	**14**	**15**	**16**	**17**	**18**	**TP**	**TN**	**FP**	**FN**	**ACC**	**SE**	**SP**	**MCC**	**EFF**
1	4	0	0	0	0	0	0	0	0	0	0	0	0	0	0	0	0	0	4	68	0	0	100.00	1.00	1.00	1.00	1.00
2	0	4	0	0	0	0	0	0	0	0	0	0	0	0	0	0	0	0	4	68	0	0	100.00	1.00	1.00	1.00	1.00
3	0	0	4	0	0	0	0	0	0	0	0	0	0	0	0	0	0	0	4	68	0	0	100.00	1.00	1.00	1.00	1.00
4	0	0	0	4	0	0	0	0	0	0	0	0	0	0	0	0	0	0	4	68	0	0	100.00	1.00	1.00	1.00	1.00
5	0	0	0	0	4	0	0	0	0	0	0	0	0	0	0	0	0	0	4	68	0	0	100.00	1.00	1.00	1.00	1.00
6	0	0	0	0	0	4	0	0	0	0	0	0	0	0	0	0	0	0	4	68	0	0	100.00	1.00	1.00	1.00	1.00
7	0	0	0	0	0	0	4	0	0	0	0	0	0	0	0	0	0	0	4	68	0	0	100.00	1.00	1.00	1.00	1.00
8	0	0	0	0	0	0	0	4	0	0	0	0	0	0	0	0	0	0	4	68	0	0	100.00	1.00	1.00	1.00	1.00
9	0	0	0	0	0	0	0	0	4	0	0	0	0	0	0	0	0	0	4	68	0	0	100.00	1.00	1.00	1.00	1.00
10	0	0	0	0	0	0	0	0	0	4	0	0	0	0	0	0	0	0	4	68	0	0	100.00	1.00	1.00	1.00	1.00
11	0	0	0	0	0	0	0	0	0	0	4	0	0	0	0	0	0	0	4	68	0	0	100.00	1.00	1.00	1.00	1.00
12	0	0	0	0	0	0	0	0	0	0	0	4	0	0	0	0	0	0	4	68	0	0	100.00	1.00	1.00	1.00	1.00
13	0	0	0	0	0	0	0	0	0	0	0	0	4	0	0	0	0	0	4	68	0	0	100.00	1.00	1.00	1.00	1.00
14	0	0	0	0	0	0	0	0	0	0	0	0	0	4	0	0	0	0	4	68	0	0	100.00	1.00	1.00	1.00	1.00
15	0	0	0	0	0	0	0	0	0	0	0	0	0	0	4	0	0	0	4	68	0	0	100.00	1.00	1.00	1.00	1.00
16	0	0	0	0	0	0	0	0	0	0	0	0	0	0	0	4	0	0	4	68	0	0	100.00	1.00	1.00	1.00	1.00
17	0	0	0	0	0	0	0	0	0	0	0	0	0	0	0	0	4	0	4	68	0	0	100.00	1.00	1.00	1.00	1.00
18	0	0	0	0	0	0	0	0	0	0	0	0	0	0	0	0	0	4	4	68	0	0	100.00	1.00	1.00	1.00	1.00

Table S30. Confusion matrixes of the calibration set and validation set of GASVM-MIR-SVM

Calibration set																												
**Class**	**1**	**2**	**3**	**4**	**5**	**6**	**7**	**8**	**9**	**10**	**11**	**12**	**13**	**14**	**15**	**16**	**17**	**18**	**TP**	**TN**	**FP**	**FN**	**ACC**	**SE**	**SP**	**MCC**	**EFF**
1	6	0	0	0	0	0	0	0	0	0	0	0	0	0	0	0	0	0	6	102	0	0	100.00	1.00	1.00	1.00	1.00
2	0	6	0	0	0	0	0	0	0	0	0	0	0	0	0	0	0	0	6	102	0	0	100.00	1.00	1.00	1.00	1.00
3	0	0	6	0	0	0	0	0	0	0	0	0	0	0	0	0	0	0	6	102	0	0	100.00	1.00	1.00	1.00	1.00
4	0	0	0	6	0	0	0	0	0	0	0	0	0	0	0	0	0	0	6	102	0	0	100.00	1.00	1.00	1.00	1.00
5	0	0	0	0	6	0	0	0	0	0	0	0	0	0	0	0	0	0	6	102	0	0	100.00	1.00	1.00	1.00	1.00
6	0	0	0	0	0	6	0	0	0	0	0	0	0	0	0	0	0	0	6	102	0	0	100.00	1.00	1.00	1.00	1.00
7	0	0	0	0	0	0	6	0	0	0	0	0	0	0	0	0	0	0	6	102	0	0	100.00	1.00	1.00	1.00	1.00
8	0	0	0	0	0	0	0	6	0	0	0	0	0	0	0	0	0	0	6	102	0	0	100.00	1.00	1.00	1.00	1.00
9	0	0	0	0	0	0	0	0	6	0	0	0	0	0	0	0	0	0	6	102	0	0	100.00	1.00	1.00	1.00	1.00
10	0	0	0	0	0	0	0	0	0	6	0	0	0	0	0	0	0	0	6	102	0	0	100.00	1.00	1.00	1.00	1.00
11	0	0	0	0	0	0	0	0	0	0	6	0	0	0	0	0	0	0	6	102	0	0	100.00	1.00	1.00	1.00	1.00
12	0	0	0	0	0	0	0	0	0	0	0	6	0	0	0	0	0	0	6	102	0	0	100.00	1.00	1.00	1.00	1.00
13	0	0	0	0	0	0	0	0	0	0	0	0	6	0	0	0	0	0	6	102	0	0	100.00	1.00	1.00	1.00	1.00
14	0	0	0	0	0	0	0	0	0	0	0	0	0	6	0	0	0	0	6	102	0	0	100.00	1.00	1.00	1.00	1.00
15	0	0	0	0	0	0	0	0	0	0	0	0	0	0	6	0	0	0	6	102	0	0	100.00	1.00	1.00	1.00	1.00
16	0	0	0	0	0	0	0	0	0	0	0	0	0	0	0	6	0	0	6	102	0	0	100.00	1.00	1.00	1.00	1.00
17	0	0	0	0	0	0	0	0	0	0	0	0	0	0	0	0	6	0	6	102	0	0	100.00	1.00	1.00	1.00	1.00
18	0	0	0	0	0	0	0	0	0	0	0	0	0	0	0	0	0	6	6	102	0	0	100.00	1.00	1.00	1.00	1.00

Validation set																												
**Class**	**1**	**2**	**3**	**4**	**5**	**6**	**7**	**8**	**9**	**10**	**11**	**12**	**13**	**14**	**15**	**16**	**17**	**18**	**TP**	**TN**	**FP**	**FN**	**ACC**	**SE**	**SP**	**MCC**	**EFF**
1	4	0	0	0	0	0	0	0	0	0	0	0	0	0	0	0	0	0	4	68	0	0	100.00	1.00	1.00	1.00	1.00
2	0	4	0	0	0	0	0	0	0	0	0	0	0	0	0	0	0	0	4	68	0	0	100.00	1.00	1.00	1.00	1.00
3	0	0	4	0	0	0	0	0	0	0	0	0	0	0	0	0	0	0	4	68	0	0	100.00	1.00	1.00	1.00	1.00
4	0	0	0	4	0	0	0	0	0	0	0	0	0	0	0	0	0	0	4	68	0	0	100.00	1.00	1.00	1.00	1.00
5	0	0	0	0	4	0	0	0	0	0	0	0	0	0	0	0	0	0	4	68	0	0	100.00	1.00	1.00	1.00	1.00
6	0	0	0	0	0	4	0	0	0	0	0	0	0	0	0	0	0	0	4	68	0	0	100.00	1.00	1.00	1.00	1.00
7	0	0	0	0	0	0	4	0	0	0	0	0	0	0	0	0	0	0	4	68	0	0	100.00	1.00	1.00	1.00	1.00
8	0	0	0	0	0	0	0	4	0	0	0	0	0	0	0	0	0	0	4	68	0	0	100.00	1.00	1.00	1.00	1.00
9	0	0	0	0	0	0	0	0	4	0	0	0	0	0	0	0	0	0	4	68	0	0	100.00	1.00	1.00	1.00	1.00
10	0	0	0	0	0	0	0	0	0	4	0	0	0	0	0	0	0	0	4	68	0	0	100.00	1.00	1.00	1.00	1.00
11	0	0	0	0	0	0	0	0	0	0	4	0	0	0	0	0	0	0	4	68	0	0	100.00	1.00	1.00	1.00	1.00
12	0	0	0	0	0	0	0	0	0	0	0	4	0	0	0	0	0	0	4	68	0	0	100.00	1.00	1.00	1.00	1.00
13	0	0	0	0	0	0	0	0	0	0	0	0	4	0	0	0	0	0	4	68	0	0	100.00	1.00	1.00	1.00	1.00
14	0	0	0	0	0	0	0	0	0	0	0	0	0	4	0	0	0	0	4	68	0	0	100.00	1.00	1.00	1.00	1.00
15	0	0	0	0	0	0	0	0	0	0	0	0	0	0	4	0	0	0	4	68	0	0	100.00	1.00	1.00	1.00	1.00
16	0	0	0	0	0	0	0	0	0	0	0	0	0	0	0	4	0	0	4	68	0	0	100.00	1.00	1.00	1.00	1.00
17	0	0	0	0	0	0	0	0	0	0	0	0	0	0	0	0	4	0	4	68	0	0	100.00	1.00	1.00	1.00	1.00
18	0	0	0	0	0	0	0	0	0	0	0	0	0	0	0	0	0	4	4	68	0	0	100.00	1.00	1.00	1.00	1.00

Table S31. Confusion matrixes of the calibration set and validation set of Ven-MIR-SVM

Calibration set																											
**Class**	**1**	**2**	**3**	**4**	**5**	**6**	**7**	**8**	**9**	**10**	**11**	**12**	**13**	**14**	**15**	**16**	**17**	**18**	**TP**	**TN**	**FP**	**FN**	**ACC**	**SE**	**SP**	**MCC**	**EFF**
1	6	0	0	0	0	0	0	0	0	0	0	0	0	0	0	0	0	0	6	102	0	0	100.00	1.00	1.00	1.00	1.00
2	0	6	0	0	0	0	0	0	0	0	0	0	0	0	0	0	0	0	6	102	0	0	100.00	1.00	1.00	1.00	1.00
3	0	0	6	0	0	0	0	0	0	0	0	0	0	0	0	0	0	0	6	102	0	0	100.00	1.00	1.00	1.00	1.00
4	0	0	0	6	0	0	0	0	0	0	0	0	0	0	0	0	0	0	6	102	0	0	100.00	1.00	1.00	1.00	1.00
5	0	0	0	0	6	0	0	0	0	0	0	0	0	0	0	0	0	0	6	102	0	0	100.00	1.00	1.00	1.00	1.00
6	0	0	0	0	0	6	0	0	0	0	0	0	0	0	0	0	0	0	6	102	0	0	100.00	1.00	1.00	1.00	1.00
7	0	0	0	0	0	0	6	0	0	0	0	0	0	0	0	0	0	0	6	102	0	0	100.00	1.00	1.00	1.00	1.00
8	0	0	0	0	0	0	0	6	0	0	0	0	0	0	0	0	0	0	6	102	0	0	100.00	1.00	1.00	1.00	1.00
9	0	0	0	0	0	0	0	0	6	0	0	0	0	0	0	0	0	0	6	102	0	0	100.00	1.00	1.00	1.00	1.00
10	0	0	0	0	0	0	0	0	0	6	0	0	0	0	0	0	0	0	6	102	0	0	100.00	1.00	1.00	1.00	1.00
11	0	0	0	0	0	0	0	0	0	0	6	0	0	0	0	0	0	0	6	102	0	0	100.00	1.00	1.00	1.00	1.00
12	0	0	0	0	0	0	0	0	0	0	0	6	0	0	0	0	0	0	6	102	0	0	100.00	1.00	1.00	1.00	1.00
13	0	0	0	0	0	0	0	0	0	0	0	0	6	0	0	0	0	0	6	102	0	0	100.00	1.00	1.00	1.00	1.00
14	0	0	0	0	0	0	0	0	0	0	0	0	0	6	0	0	0	0	6	102	0	0	100.00	1.00	1.00	1.00	1.00
15	0	0	0	0	0	0	0	0	0	0	0	0	0	0	6	0	0	0	6	102	0	0	100.00	1.00	1.00	1.00	1.00
16	0	0	0	0	0	0	0	0	0	0	0	0	0	0	0	6	0	0	6	102	0	0	100.00	1.00	1.00	1.00	1.00
17	0	0	0	0	0	0	0	0	0	0	0	0	0	0	0	0	6	0	6	102	0	0	100.00	1.00	1.00	1.00	1.00
18	0	0	0	0	0	0	0	0	0	0	0	0	0	0	0	0	0	6	6	102	0	0	100.00	1.00	1.00	1.00	1.00

Validation set																											
**Class**	**1**	**2**	**3**	**4**	**5**	**6**	**7**	**8**	**9**	**10**	**11**	**12**	**13**	**14**	**15**	**16**	**17**	**18**	**TP**	**TN**	**FP**	**FN**	**ACC**	**SE**	**SP**	**MCC**	**EFF**
1	4	0	0	0	0	0	0	0	0	0	0	0	0	0	0	0	0	0	4	68	0	0	100.00	1.00	1.00	1.00	1.00
2	0	4	0	0	0	0	0	0	0	0	0	0	0	0	0	0	0	0	4	68	0	0	100.00	1.00	1.00	1.00	1.00
3	0	0	4	0	0	0	0	0	0	0	0	0	0	0	0	0	0	0	4	68	0	0	100.00	1.00	1.00	1.00	1.00
4	0	0	0	4	0	0	0	0	0	0	0	0	0	0	0	0	0	0	4	68	0	0	100.00	1.00	1.00	1.00	1.00
5	0	0	0	0	4	0	0	0	0	0	0	0	0	0	0	0	0	0	4	68	0	0	100.00	1.00	1.00	1.00	1.00
6	0	0	0	0	0	4	0	0	0	0	0	0	0	0	0	0	0	0	4	68	0	0	100.00	1.00	1.00	1.00	1.00
7	0	0	0	0	0	0	4	0	0	0	0	0	0	0	0	0	0	0	4	68	0	0	100.00	1.00	1.00	1.00	1.00
8	0	0	0	0	0	0	0	4	0	0	0	0	0	0	0	0	0	0	4	67	1	0	98.61	1.00	0.99	0.89	0.99
9	0	0	0	0	0	0	0	1	3	0	0	0	0	0	0	0	0	0	3	68	0	1	98.61	0.75	1.00	0.86	0.87
10	0	0	0	0	0	0	0	0	0	4	0	0	0	0	0	0	0	0	4	68	0	0	100.00	1.00	1.00	1.00	1.00
11	0	0	0	0	0	0	0	0	0	0	4	0	0	0	0	0	0	0	4	68	0	0	100.00	1.00	1.00	1.00	1.00
12	0	0	0	0	0	0	0	0	0	0	0	4	0	0	0	0	0	0	4	68	0	0	100.00	1.00	1.00	1.00	1.00
13	0	0	0	0	0	0	0	0	0	0	0	0	4	0	0	0	0	0	4	68	0	0	100.00	1.00	1.00	1.00	1.00
14	0	0	0	0	0	0	0	0	0	0	0	0	0	4	0	0	0	0	4	68	0	0	100.00	1.00	1.00	1.00	1.00
15	0	0	0	0	0	0	0	0	0	0	0	0	0	0	4	0	0	0	4	68	0	0	100.00	1.00	1.00	1.00	1.00
16	0	0	0	0	0	0	0	0	0	0	0	0	0	0	0	4	0	0	4	68	0	0	100.00	1.00	1.00	1.00	1.00
17	0	0	0	0	0	0	0	0	0	0	0	0	0	0	0	0	4	0	4	68	0	0	100.00	1.00	1.00	1.00	1.00
18	0	0	0	0	0	0	0	0	0	0	0	0	0	0	0	0	0	4	4	68	0	0	100.00	1.00	1.00	1.00	1.00

Table S32. Confusion matrixes of the calibration set and validation set of VIP-MIR-KNN

Calibration set																												
**Class**	**1**	**2**	**3**	**4**	**5**	**6**	**7**	**8**	**9**	**10**	**11**	**12**	**13**	**14**	**15**	**16**	**17**	**18**	**TP**	**TN**	**FP**	**FN**	**ACC**	**SE**	**SP**	**MCC**	**EFF**
1	6	0	0	0	0	0	0	0	0	0	0	0	0	0	0	0	0	0	6	102	0	0	100.00	1.00	1.00	1.00	1.00
2	0	6	0	0	0	0	0	0	0	0	0	0	0	0	0	0	0	0	6	102	0	0	100.00	1.00	1.00	1.00	1.00
3	0	0	6	0	0	0	0	0	0	0	0	0	0	0	0	0	0	0	6	102	0	0	100.00	1.00	1.00	1.00	1.00
4	0	0	0	6	0	0	0	0	0	0	0	0	0	0	0	0	0	0	6	102	0	0	100.00	1.00	1.00	1.00	1.00
5	0	0	0	0	6	0	0	0	0	0	0	0	0	0	0	0	0	0	6	102	0	0	100.00	1.00	1.00	1.00	1.00
6	0	0	0	0	0	6	0	0	0	0	0	0	0	0	0	0	0	0	6	102	0	0	100.00	1.00	1.00	1.00	1.00
7	0	0	0	0	0	0	6	0	0	0	0	0	0	0	0	0	0	0	6	102	0	0	100.00	1.00	1.00	1.00	1.00
8	0	0	0	0	0	0	0	6	0	0	0	0	0	0	0	0	0	0	6	102	0	0	100.00	1.00	1.00	1.00	1.00
9	0	0	0	0	0	0	0	0	6	0	0	0	0	0	0	0	0	0	6	102	0	0	100.00	1.00	1.00	1.00	1.00
10	0	0	0	0	0	0	0	0	0	6	0	0	0	0	0	0	0	0	6	102	0	0	100.00	1.00	1.00	1.00	1.00
11	0	0	0	0	0	0	0	0	0	0	6	0	0	0	0	0	0	0	6	102	0	0	100.00	1.00	1.00	1.00	1.00
12	0	0	0	0	0	0	0	0	0	0	0	6	0	0	0	0	0	0	6	102	0	0	100.00	1.00	1.00	1.00	1.00
13	0	0	0	0	0	0	0	0	0	0	0	0	6	0	0	0	0	0	6	102	0	0	100.00	1.00	1.00	1.00	1.00
14	0	0	0	0	0	0	0	0	0	0	0	0	0	6	0	0	0	0	6	102	0	0	100.00	1.00	1.00	1.00	1.00
15	0	0	0	0	0	0	0	0	0	0	0	0	0	0	6	0	0	0	6	102	0	0	100.00	1.00	1.00	1.00	1.00
16	0	0	0	0	0	0	0	0	0	0	0	0	0	0	0	6	0	0	6	102	0	0	100.00	1.00	1.00	1.00	1.00
17	0	0	0	0	0	0	0	0	0	0	0	0	0	0	0	0	6	0	6	102	0	0	100.00	1.00	1.00	1.00	1.00
18	0	0	0	0	0	0	0	0	0	0	0	0	0	0	0	0	0	6	6	102	0	0	100.00	1.00	1.00	1.00	1.00

Validation set																												
**Class**	**1**	**2**	**3**	**4**	**5**	**6**	**7**	**8**	**9**	**10**	**11**	**12**	**13**	**14**	**15**	**16**	**17**	**18**	**TP**	**TN**	**FP**	**FN**	**ACC**	**SE**	**SP**	**MCC**	**EFF**
1	4	0	0	0	0	0	0	0	0	0	0	0	0	0	0	0	0	0	4	68	0	0	100.00	1.00	1.00	1.00	1.00
2	0	4	0	0	0	0	0	0	0	0	0	0	0	0	0	0	0	0	4	68	0	0	100.00	1.00	1.00	1.00	1.00
3	0	0	4	0	0	0	0	0	0	0	0	0	0	0	0	0	0	0	4	68	0	0	100.00	1.00	1.00	1.00	1.00
4	0	0	0	4	0	0	0	0	0	0	0	0	0	0	0	0	0	0	4	68	0	0	100.00	1.00	1.00	1.00	1.00
5	0	0	0	0	4	0	0	0	0	0	0	0	0	0	0	0	0	0	4	68	0	0	100.00	1.00	1.00	1.00	1.00
6	0	0	0	0	0	4	0	0	0	0	0	0	0	0	0	0	0	0	4	68	0	0	100.00	1.00	1.00	1.00	1.00
7	0	0	0	0	0	0	4	0	0	0	0	0	0	0	0	0	0	0	4	68	0	0	100.00	1.00	1.00	1.00	1.00
8	0	0	0	0	0	0	0	4	0	0	0	0	0	0	0	0	0	0	4	67	1	0	98.61	1.00	0.99	0.89	0.99
9	0	0	0	0	0	0	0	1	3	0	0	0	0	0	0	0	0	0	3	68	0	1	98.61	0.75	1.00	0.86	0.87
10	0	0	0	0	0	0	0	0	0	4	0	0	0	0	0	0	0	0	4	68	0	0	100.00	1.00	1.00	1.00	1.00
11	0	0	0	0	0	0	0	0	0	0	4	0	0	0	0	0	0	0	4	68	0	0	100.00	1.00	1.00	1.00	1.00
12	0	0	0	0	0	0	0	0	0	0	0	4	0	0	0	0	0	0	4	68	0	0	100.00	1.00	1.00	1.00	1.00
13	0	0	0	0	0	0	0	0	0	0	0	0	4	0	0	0	0	0	4	68	0	0	100.00	1.00	1.00	1.00	1.00
14	0	0	0	0	0	0	0	0	0	0	0	0	0	4	0	0	0	0	4	68	0	0	100.00	1.00	1.00	1.00	1.00
15	0	0	0	0	0	0	0	0	0	0	0	0	0	0	4	0	0	0	4	68	0	0	100.00	1.00	1.00	1.00	1.00
16	0	0	0	0	0	0	0	0	0	0	0	0	0	0	0	4	0	0	4	68	0	0	100.00	1.00	1.00	1.00	1.00
17	0	0	0	0	0	0	0	0	0	0	0	0	0	0	0	0	4	0	4	68	0	0	100.00	1.00	1.00	1.00	1.00
18	0	0	0	0	0	0	0	0	0	0	0	0	0	0	0	0	0	4	4	68	0	0	100.00	1.00	1.00	1.00	1.00

Table S33. Confusion matrixes of the calibration set and validation set of Bor-MIR-KNN

Calibration set																												
**Class**	**1**	**2**	**3**	**4**	**5**	**6**	**7**	**8**	**9**	**10**	**11**	**12**	**13**	**14**	**15**	**16**	**17**	**18**	**TP**	**TN**	**FP**	**FN**	**ACC**	**SE**	**SP**	**MCC**	**EFF**
1	6	0	0	0	0	0	0	0	0	0	0	0	0	0	0	0	0	0	6	102	0	0	100.00	1.00	1.00	1.00	1.00
2	0	6	0	0	0	0	0	0	0	0	0	0	0	0	0	0	0	0	6	102	0	0	100.00	1.00	1.00	1.00	1.00
3	0	0	6	0	0	0	0	0	0	0	0	0	0	0	0	0	0	0	6	102	0	0	100.00	1.00	1.00	1.00	1.00
4	0	0	0	6	0	0	0	0	0	0	0	0	0	0	0	0	0	0	6	102	0	0	100.00	1.00	1.00	1.00	1.00
5	0	0	0	0	6	0	0	0	0	0	0	0	0	0	0	0	0	0	6	102	0	0	100.00	1.00	1.00	1.00	1.00
6	0	0	0	0	0	6	0	0	0	0	0	0	0	0	0	0	0	0	6	102	0	0	100.00	1.00	1.00	1.00	1.00
7	0	0	0	0	0	0	6	0	0	0	0	0	0	0	0	0	0	0	6	102	0	0	100.00	1.00	1.00	1.00	1.00
8	0	0	0	0	0	0	0	6	0	0	0	0	0	0	0	0	0	0	6	102	0	0	100.00	1.00	1.00	1.00	1.00
9	0	0	0	0	0	0	0	0	6	0	0	0	0	0	0	0	0	0	6	102	0	0	100.00	1.00	1.00	1.00	1.00
10	0	0	0	0	0	0	0	0	0	6	0	0	0	0	0	0	0	0	6	102	0	0	100.00	1.00	1.00	1.00	1.00
11	0	0	0	0	0	0	0	0	0	0	6	0	0	0	0	0	0	0	6	102	0	0	100.00	1.00	1.00	1.00	1.00
12	0	0	0	0	0	0	0	0	0	0	0	6	0	0	0	0	0	0	6	102	0	0	100.00	1.00	1.00	1.00	1.00
13	0	0	0	0	0	0	0	0	0	0	0	0	6	0	0	0	0	0	6	102	0	0	100.00	1.00	1.00	1.00	1.00
14	0	0	0	0	0	0	0	0	0	0	0	0	0	6	0	0	0	0	6	102	0	0	100.00	1.00	1.00	1.00	1.00
15	0	0	0	0	0	0	0	0	0	0	0	0	0	0	6	0	0	0	6	102	0	0	100.00	1.00	1.00	1.00	1.00
16	0	0	0	0	0	0	0	0	0	0	0	0	0	0	0	6	0	0	6	102	0	0	100.00	1.00	1.00	1.00	1.00
17	0	0	0	0	0	0	0	0	0	0	0	0	0	0	0	0	6	0	6	102	0	0	100.00	1.00	1.00	1.00	1.00
18	0	0	0	0	0	0	0	0	0	0	0	0	0	0	0	0	0	6	6	102	0	0	100.00	1.00	1.00	1.00	1.00

Validation set

Class	1	2	3	4	5	6	7	8	9	10	11	12	13	14	15	16	17	18	TP	TN	FP	FN	ACC	SE	SP	MCC	EFF
1	4	0	0	0	0	0	0	0	0	0	0	0	0	0	0	0	0	0	4	68	0	0	100.00	1.00	1.00	1.00	1.00
2	0	4	0	0	0	0	0	0	0	0	0	0	0	0	0	0	0	0	4	68	0	0	100.00	1.00	1.00	1.00	1.00
3	0	0	4	0	0	0	0	0	0	0	0	0	0	0	0	0	0	0	4	68	0	0	100.00	1.00	1.00	1.00	1.00
4	0	0	0	4	0	0	0	0	0	0	0	0	0	0	0	0	0	0	4	68	0	0	100.00	1.00	1.00	1.00	1.00
5	0	0	0	0	4	0	0	0	0	0	0	0	0	0	0	0	0	0	4	68	0	0	100.00	1.00	1.00	1.00	1.00
6	0	0	0	0	0	4	0	0	0	0	0	0	0	0	0	0	0	0	4	68	0	0	100.00	1.00	1.00	1.00	1.00
7	0	0	0	0	0	0	4	0	0	0	0	0	0	0	0	0	0	0	4	68	0	0	100.00	1.00	1.00	1.00	1.00
8	0	0	0	0	0	0	0	4	0	0	0	0	0	0	0	0	0	0	4	66	2	0	97.22	1.00	0.97	0.80	0.99
9	0	0	0	0	0	0	0	2	2	0	0	0	0	0	0	0	0	0	2	68	0	2	97.22	0.50	1.00	0.70	0.71
10	0	0	0	0	0	0	0	0	0	4	0	0	0	0	0	0	0	0	4	68	0	0	100.00	1.00	1.00	1.00	1.00
11	0	0	0	0	0	0	0	0	0	0	4	0	0	0	0	0	0	0	4	68	0	0	100.00	1.00	1.00	1.00	1.00
12	0	0	0	0	0	0	0	0	0	0	0	4	0	0	0	0	0	0	4	68	0	0	100.00	1.00	1.00	1.00	1.00
13	0	0	0	0	0	0	0	0	0	0	0	0	4	0	0	0	0	0	4	68	0	0	100.00	1.00	1.00	1.00	1.00
14	0	0	0	0	0	0	0	0	0	0	0	0	0	4	0	0	0	0	4	68	0	0	100.00	1.00	1.00	1.00	1.00
15	0	0	0	0	0	0	0	0	0	0	0	0	0	0	4	0	0	0	4	68	0	0	100.00	1.00	1.00	1.00	1.00
16	0	0	0	0	0	0	0	0	0	0	0	0	0	0	0	4	0	0	4	68	0	0	100.00	1.00	1.00	1.00	1.00
17	0	0	0	0	0	0	0	0	0	0	0	0	0	0	0	0	4	0	4	68	0	0	100.00	1.00	1.00	1.00	1.00
18	0	0	0	0	0	0	0	0	0	0	0	0	0	0	0	0	0	4	4	68	0	0	100.00	1.00	1.00	1.00	1.00

Table S34. Confusion matrixes of the calibration set and validation set of GARF-MIR-KNN

Calibration set																											
**Class**	**1**	**2**	**3**	**4**	**5**	**6**	**7**	**8**	**9**	**10**	**11**	**12**	**13**	**14**	**15**	**16**	**17**	**18**	**TP**	**TN**	**FP**	**FN**	**ACC**	**SE**	**SP**	**MCC**	**EFF**
1	6	0	0	0	0	0	0	0	0	0	0	0	0	0	0	0	0	0	6	102	0	0	100.00	1.00	1.00	1.00	1.00
2	0	6	0	0	0	0	0	0	0	0	0	0	0	0	0	0	0	0	6	102	0	0	100.00	1.00	1.00	1.00	1.00
3	0	0	6	0	0	0	0	0	0	0	0	0	0	0	0	0	0	0	6	102	0	0	100.00	1.00	1.00	1.00	1.00
4	0	0	0	6	0	0	0	0	0	0	0	0	0	0	0	0	0	0	6	102	0	0	100.00	1.00	1.00	1.00	1.00
5	0	0	0	0	6	0	0	0	0	0	0	0	0	0	0	0	0	0	6	102	0	0	100.00	1.00	1.00	1.00	1.00
6	0	0	0	0	0	6	0	0	0	0	0	0	0	0	0	0	0	0	6	102	0	0	100.00	1.00	1.00	1.00	1.00
7	0	0	0	0	0	0	6	0	0	0	0	0	0	0	0	0	0	0	6	102	0	0	100.00	1.00	1.00	1.00	1.00
8	0	0	0	0	0	0	0	6	0	0	0	0	0	0	0	0	0	0	6	102	0	0	100.00	1.00	1.00	1.00	1.00
9	0	0	0	0	0	0	0	0	6	0	0	0	0	0	0	0	0	0	6	102	0	0	100.00	1.00	1.00	1.00	1.00
10	0	0	0	0	0	0	0	0	0	6	0	0	0	0	0	0	0	0	6	102	0	0	100.00	1.00	1.00	1.00	1.00
11	0	0	0	0	0	0	0	0	0	0	6	0	0	0	0	0	0	0	6	102	0	0	100.00	1.00	1.00	1.00	1.00
12	0	0	0	0	0	0	0	0	0	0	0	6	0	0	0	0	0	0	6	102	0	0	100.00	1.00	1.00	1.00	1.00
13	0	0	0	0	0	0	0	0	0	0	0	0	6	0	0	0	0	0	6	102	0	0	100.00	1.00	1.00	1.00	1.00
14	0	0	0	0	0	0	0	0	0	0	0	0	0	6	0	0	0	0	6	102	0	0	100.00	1.00	1.00	1.00	1.00
15	0	0	0	0	0	0	0	0	0	0	0	0	0	0	6	0	0	0	6	102	0	0	100.00	1.00	1.00	1.00	1.00
16	0	0	0	0	0	0	0	0	0	0	0	0	0	0	0	6	0	0	6	102	0	0	100.00	1.00	1.00	1.00	1.00
17	0	0	0	0	0	0	0	0	0	0	0	0	0	0	0	0	6	0	6	102	0	0	100.00	1.00	1.00	1.00	1.00
18	0	0	0	0	0	0	0	0	0	0	0	0	0	0	0	0	0	6	6	102	0	0	100.00	1.00	1.00	1.00	1.00

Validation set																												
Class	1	2	3	4	5	6	7	8	9	10	11	12	13	14	15	16	17	18	TP	TN	FP	FN	ACC	SE	SP	MCC	EFF
1	4	0	0	0	0	0	0	0	0	0	0	0	0	0	0	0	0	0	4	68	0	0	100.00	1.00	1.00	1.00	1.00
2	0	4	0	0	0	0	0	0	0	0	0	0	0	0	0	0	0	0	4	68	0	0	100.00	1.00	1.00	1.00	1.00
3	0	0	4	0	0	0	0	0	0	0	0	0	0	0	0	0	0	0	4	68	0	0	100.00	1.00	1.00	1.00	1.00
4	0	0	0	4	0	0	0	0	0	0	0	0	0	0	0	0	0	0	4	68	0	0	100.00	1.00	1.00	1.00	1.00
5	0	0	0	0	4	0	0	0	0	0	0	0	0	0	0	0	0	0	4	68	0	0	100.00	1.00	1.00	1.00	1.00
6	0	0	0	0	0	4	0	0	0	0	0	0	0	0	0	0	0	0	4	68	0	0	100.00	1.00	1.00	1.00	1.00
7	0	0	0	0	0	0	4	0	0	0	0	0	0	0	0	0	0	0	4	68	0	0	100.00	1.00	1.00	1.00	1.00
8	0	0	0	0	0	0	0	4	0	0	0	0	0	0	0	0	0	0	4	66	2	0	97.22	1.00	0.97	0.80	0.99
9	0	0	0	0	0	0	0	2	2	0	0	0	0	0	0	0	0	0	2	68	0	2	97.22	0.50	1.00	0.70	0.71
10	0	0	0	0	0	0	0	0	0	4	0	0	0	0	0	0	0	0	4	68	0	0	100.00	1.00	1.00	1.00	1.00
11	0	0	0	0	0	0	0	0	0	0	4	0	0	0	0	0	0	0	4	67	1	0	98.61	1.00	0.99	0.89	0.99
12	0	0	0	0	0	0	0	0	0	0	0	4	0	0	0	0	0	0	4	68	0	0	100.00	1.00	1.00	1.00	1.00
13	0	0	0	0	0	0	0	0	0	0	0	0	4	0	0	0	0	0	4	68	0	0	100.00	1.00	1.00	1.00	1.00
14	0	0	0	0	0	0	0	0	0	0	0	0	0	4	0	0	0	0	4	68	0	0	100.00	1.00	1.00	1.00	1.00
15	0	0	0	0	0	0	0	0	0	0	0	0	0	0	4	0	0	0	4	68	0	0	100.00	1.00	1.00	1.00	1.00
16	0	0	0	0	0	0	0	0	0	0	1	0	0	0	0	3	0	0	3	68	0	1	98.61	0.75	1.00	0.86	0.87
17	0	0	0	0	0	0	0	0	0	0	0	0	0	0	0	0	4	0	4	68	0	0	100.00	1.00	1.00	1.00	1.00
18	0	0	0	0	0	0	0	0	0	0	0	0	0	0	0	0	0	4	4	68	0	0	100.00	1.00	1.00	1.00	1.00

Table S35. Confusion matrixes of the calibration set and validation set of GASVM-MIR-KNN

Calibration set																											
**Class**	**1**	**2**	**3**	**4**	**5**	**6**	**7**	**8**	**9**	**10**	**11**	**12**	**13**	**14**	**15**	**16**	**17**	**18**	**TP**	**TN**	**FP**	**FN**	**ACC**	**SE**	**SP**	**MCC**	**EFF**
1	6	0	0	0	0	0	0	0	0	0	0	0	0	0	0	0	0	0	6	102	0	0	100.00	1.00	1.00	1.00	1.00
2	0	6	0	0	0	0	0	0	0	0	0	0	0	0	0	0	0	0	6	102	0	0	100.00	1.00	1.00	1.00	1.00
3	0	0	6	0	0	0	0	0	0	0	0	0	0	0	0	0	0	0	6	102	0	0	100.00	1.00	1.00	1.00	1.00
4	0	0	0	6	0	0	0	0	0	0	0	0	0	0	0	0	0	0	6	102	0	0	100.00	1.00	1.00	1.00	1.00
5	0	0	0	0	6	0	0	0	0	0	0	0	0	0	0	0	0	0	6	102	0	0	100.00	1.00	1.00	1.00	1.00
6	0	0	0	0	0	6	0	0	0	0	0	0	0	0	0	0	0	0	6	102	0	0	100.00	1.00	1.00	1.00	1.00
7	0	0	0	0	0	0	6	0	0	0	0	0	0	0	0	0	0	0	6	102	0	0	100.00	1.00	1.00	1.00	1.00
8	0	0	0	0	0	0	0	6	0	0	0	0	0	0	0	0	0	0	6	102	0	0	100.00	1.00	1.00	1.00	1.00
9	0	0	0	0	0	0	0	0	6	0	0	0	0	0	0	0	0	0	6	102	0	0	100.00	1.00	1.00	1.00	1.00
10	0	0	0	0	0	0	0	0	0	6	0	0	0	0	0	0	0	0	6	102	0	0	100.00	1.00	1.00	1.00	1.00
11	0	0	0	0	0	0	0	0	0	0	6	0	0	0	0	0	0	0	6	102	0	0	100.00	1.00	1.00	1.00	1.00
12	0	0	0	0	0	0	0	0	0	0	0	6	0	0	0	0	0	0	6	102	0	0	100.00	1.00	1.00	1.00	1.00
13	0	0	0	0	0	0	0	0	0	0	0	0	6	0	0	0	0	0	6	102	0	0	100.00	1.00	1.00	1.00	1.00
14	0	0	0	0	0	0	0	0	0	0	0	0	0	6	0	0	0	0	6	102	0	0	100.00	1.00	1.00	1.00	1.00
15	0	0	0	0	0	0	0	0	0	0	0	0	0	0	6	0	0	0	6	102	0	0	100.00	1.00	1.00	1.00	1.00
16	0	0	0	0	0	0	0	0	0	0	0	0	0	0	0	6	0	0	6	102	0	0	100.00	1.00	1.00	1.00	1.00
17	0	0	0	0	0	0	0	0	0	0	0	0	0	0	0	0	6	0	6	102	0	0	100.00	1.00	1.00	1.00	1.00
18	0	0	0	0	0	0	0	0	0	0	0	0	0	0	0	0	0	6	6	102	0	0	100.00	1.00	1.00	1.00	1.00

Validation set																											
**Class**	**1**	**2**	**3**	**4**	**5**	**6**	**7**	**8**	**9**	**10**	**11**	**12**	**13**	**14**	**15**	**16**	**17**	**18**	**TP**	**TN**	**FP**	**FN**	**ACC**	**SE**	**SP**	**MCC**	**EFF**
1	4	0	0	0	0	0	0	0	0	0	0	0	0	0	0	0	0	0	4	68	0	0	100.00	1.00	1.00	1.00	1.00
2	0	4	0	0	0	0	0	0	0	0	0	0	0	0	0	0	0	0	4	68	0	0	100.00	1.00	1.00	1.00	1.00
3	0	0	4	0	0	0	0	0	0	0	0	0	0	0	0	0	0	0	4	68	0	0	100.00	1.00	1.00	1.00	1.00
4	0	0	0	4	0	0	0	0	0	0	0	0	0	0	0	0	0	0	4	67	1	0	98.61	1.00	0.99	0.89	0.99
5	0	0	0	0	4	0	0	0	0	0	0	0	0	0	0	0	0	0	4	68	0	0	100.00	1.00	1.00	1.00	1.00
6	0	0	0	0	0	4	0	0	0	0	0	0	0	0	0	0	0	0	4	68	0	0	100.00	1.00	1.00	1.00	1.00
7	0	0	0	0	0	0	4	0	0	0	0	0	0	0	0	0	0	0	4	68	0	0	100.00	1.00	1.00	1.00	1.00
8	0	0	0	0	0	0	0	4	0	0	0	0	0	0	0	0	0	0	4	65	3	0	95.83	1.00	0.96	0.74	0.98
9	0	0	0	0	0	0	0	3	1	0	0	0	0	0	0	0	0	0	1	68	0	3	95.83	0.25	1.00	0.49	0.50
10	0	0	0	0	0	0	0	0	0	4	0	0	0	0	0	0	0	0	4	68	0	0	100.00	1.00	1.00	1.00	1.00
11	0	0	0	0	0	0	0	0	0	0	4	0	0	0	0	0	0	0	4	68	0	0	100.00	1.00	1.00	1.00	1.00
12	0	0	0	0	0	0	0	0	0	0	0	4	0	0	0	0	0	0	4	68	0	0	100.00	1.00	1.00	1.00	1.00
13	0	0	0	0	0	0	0	0	0	0	0	0	4	0	0	0	0	0	4	68	0	0	100.00	1.00	1.00	1.00	1.00
14	0	0	0	0	0	0	0	0	0	0	0	0	0	4	0	0	0	0	4	68	0	0	100.00	1.00	1.00	1.00	1.00
15	0	0	0	0	0	0	0	0	0	0	0	0	0	0	4	0	0	0	4	68	0	0	100.00	1.00	1.00	1.00	1.00
16	0	0	0	1	0	0	0	0	0	0	0	0	0	0	0	3	0	0	3	68	0	1	98.61	0.75	1.00	0.86	0.87
17	0	0	0	0	0	0	0	0	0	0	0	0	0	0	0	0	4	0	4	68	0	0	100.00	1.00	1.00	1.00	1.00
18	0	0	0	0	0	0	0	0	0	0	0	0	0	0	0	0	0	4	4	68	0	0	100.00	1.00	1.00	1.00	1.00

Table S36. Confusion matrixes of the calibration set and validation set of Ven-MIR-KNN

Calibration set																											
**Class**	**1**	**2**	**3**	**4**	**5**	**6**	**7**	**8**	**9**	**10**	**11**	**12**	**13**	**14**	**15**	**16**	**17**	**18**	**TP**	**TN**	**FP**	**FN**	**ACC**	**SE**	**SP**	**MCC**	**EFF**
1	6	0	0	0	0	0	0	0	0	0	0	0	0	0	0	0	0	0	6	102	0	0	100.00	1.00	1.00	1.00	1.00
2	0	6	0	0	0	0	0	0	0	0	0	0	0	0	0	0	0	0	6	102	0	0	100.00	1.00	1.00	1.00	1.00
3	0	0	6	0	0	0	0	0	0	0	0	0	0	0	0	0	0	0	6	102	0	0	100.00	1.00	1.00	1.00	1.00
4	0	0	0	6	0	0	0	0	0	0	0	0	0	0	0	0	0	0	6	102	0	0	100.00	1.00	1.00	1.00	1.00
5	0	0	0	0	6	0	0	0	0	0	0	0	0	0	0	0	0	0	6	102	0	0	100.00	1.00	1.00	1.00	1.00
6	0	0	0	0	0	6	0	0	0	0	0	0	0	0	0	0	0	0	6	102	0	0	100.00	1.00	1.00	1.00	1.00
7	0	0	0	0	0	0	6	0	0	0	0	0	0	0	0	0	0	0	6	102	0	0	100.00	1.00	1.00	1.00	1.00
8	0	0	0	0	0	0	0	6	0	0	0	0	0	0	0	0	0	0	6	102	0	0	100.00	1.00	1.00	1.00	1.00
9	0	0	0	0	0	0	0	0	6	0	0	0	0	0	0	0	0	0	6	102	0	0	100.00	1.00	1.00	1.00	1.00
10	0	0	0	0	0	0	0	0	0	6	0	0	0	0	0	0	0	0	6	102	0	0	100.00	1.00	1.00	1.00	1.00
11	0	0	0	0	0	0	0	0	0	0	6	0	0	0	0	0	0	0	6	102	0	0	100.00	1.00	1.00	1.00	1.00
12	0	0	0	0	0	0	0	0	0	0	0	6	0	0	0	0	0	0	6	102	0	0	100.00	1.00	1.00	1.00	1.00
13	0	0	0	0	0	0	0	0	0	0	0	0	6	0	0	0	0	0	6	102	0	0	100.00	1.00	1.00	1.00	1.00
14	0	0	0	0	0	0	0	0	0	0	0	0	0	6	0	0	0	0	6	102	0	0	100.00	1.00	1.00	1.00	1.00
15	0	0	0	0	0	0	0	0	0	0	0	0	0	0	6	0	0	0	6	102	0	0	100.00	1.00	1.00	1.00	1.00
16	0	0	0	0	0	0	0	0	0	0	0	0	0	0	0	6	0	0	6	102	0	0	100.00	1.00	1.00	1.00	1.00
17	0	0	0	0	0	0	0	0	0	0	0	0	0	0	0	0	6	0	6	102	0	0	100.00	1.00	1.00	1.00	1.00
18	0	0	0	0	0	0	0	0	0	0	0	0	0	0	0	0	0	6	6	102	0	0	100.00	1.00	1.00	1.00	1.00

Validation set																												
**Class**	**1**	**2**	**3**	**4**	**5**	**6**	**7**	**8**	**9**	**10**	**11**	**12**	**13**	**14**	**15**	**16**	**17**	**18**	**TP**	**TN**	**FP**	**FN**	**ACC**	**SE**	**SP**	**MCC**	**EFF**
1	4	0	0	0	0	0	0	0	0	0	0	0	0	0	0	0	0	0	4	68	0	0	100.00	1.00	1.00	1.00	1.00
2	0	4	0	0	0	0	0	0	0	0	0	0	0	0	0	0	0	0	4	68	0	0	100.00	1.00	1.00	1.00	1.00
3	0	0	4	0	0	0	0	0	0	0	0	0	0	0	0	0	0	0	4	68	0	0	100.00	1.00	1.00	1.00	1.00
4	0	0	0	4	0	0	0	0	0	0	0	0	0	0	0	0	0	0	4	68	0	0	100.00	1.00	1.00	1.00	1.00
5	0	0	0	0	4	0	0	0	0	0	0	0	0	0	0	0	0	0	4	68	0	0	100.00	1.00	1.00	1.00	1.00
6	0	0	0	0	0	4	0	0	0	0	0	0	0	0	0	0	0	0	4	68	0	0	100.00	1.00	1.00	1.00	1.00
7	0	0	0	0	0	0	4	0	0	0	0	0	0	0	0	0	0	0	4	68	0	0	100.00	1.00	1.00	1.00	1.00
8	0	0	0	0	0	0	0	4	0	0	0	0	0	0	0	0	0	0	4	66	2	0	97.22	1.00	0.97	0.80	0.99
9	0	0	0	0	0	0	0	2	2	0	0	0	0	0	0	0	0	0	2	68	0	2	97.22	0.50	1.00	0.70	0.71
10	0	0	0	0	0	0	0	0	0	4	0	0	0	0	0	0	0	0	4	68	0	0	100.00	1.00	1.00	1.00	1.00
11	0	0	0	0	0	0	0	0	0	0	4	0	0	0	0	0	0	0	4	68	0	0	100.00	1.00	1.00	1.00	1.00
12	0	0	0	0	0	0	0	0	0	0	0	4	0	0	0	0	0	0	4	68	0	0	100.00	1.00	1.00	1.00	1.00
13	0	0	0	0	0	0	0	0	0	0	0	0	4	0	0	0	0	0	4	68	0	0	100.00	1.00	1.00	1.00	1.00
14	0	0	0	0	0	0	0	0	0	0	0	0	0	4	0	0	0	0	4	68	0	0	100.00	1.00	1.00	1.00	1.00
15	0	0	0	0	0	0	0	0	0	0	0	0	0	0	4	0	0	0	4	68	0	0	100.00	1.00	1.00	1.00	1.00
16	0	0	0	0	0	0	0	0	0	0	0	0	0	0	0	4	0	0	4	68	0	0	100.00	1.00	1.00	1.00	1.00
17	0	0	0	0	0	0	0	0	0	0	0	0	0	0	0	0	4	0	4	68	0	0	100.00	1.00	1.00	1.00	1.00
18	0	0	0	0	0	0	0	0	0	0	0	0	0	0	0	0	0	4	4	68	0	0	100.00	1.00	1.00	1.00	1.00

Table S37. Confusion matrixes of the calibration set and validation set of SG-Ven-NIR- RF

Calibration set																											
**Class**	**1**	**2**	**3**	**4**	**5**	**6**	**7**	**8**	**9**	**10**	**11**	**12**	**13**	**14**	**15**	**16**	**17**	**18**	**TP**	**TN**	**FP**	**FN**	**ACC**	**SE**	**SP**	**MCC**	**EFF**
1	6	0	0	0	0	0	0	0	0	0	0	0	0	0	0	0	0	0	6	102	0	0	100.00	1.00	1.00	1.00	1.00
2	0	6	0	0	0	0	0	0	0	0	0	0	0	0	0	0	0	0	6	102	0	0	100.00	1.00	1.00	1.00	1.00
3	0	0	6	0	0	0	0	0	0	0	0	0	0	0	0	0	0	0	6	102	0	0	100.00	1.00	1.00	1.00	1.00
4	0	0	0	6	0	0	0	0	0	0	0	0	0	0	0	0	0	0	6	102	0	0	100.00	1.00	1.00	1.00	1.00
5	0	0	0	0	6	0	0	0	0	0	0	0	0	0	0	0	0	0	6	102	0	0	100.00	1.00	1.00	1.00	1.00
6	0	0	0	0	0	6	0	0	0	0	0	0	0	0	0	0	0	0	6	102	0	0	100.00	1.00	1.00	1.00	1.00
7	0	0	0	0	0	0	6	0	0	0	0	0	0	0	0	0	0	0	6	102	0	0	100.00	1.00	1.00	1.00	1.00
8	0	0	0	0	0	0	0	6	0	0	0	0	0	0	0	0	0	0	6	102	0	0	100.00	1.00	1.00	1.00	1.00
9	0	0	0	0	0	0	0	0	6	0	0	0	0	0	0	0	0	0	6	102	0	0	100.00	1.00	1.00	1.00	1.00
10	0	0	0	0	0	0	0	0	0	6	0	0	0	0	0	0	0	0	6	102	0	0	100.00	1.00	1.00	1.00	1.00
11	0	0	0	0	0	0	0	0	0	0	6	0	0	0	0	0	0	0	6	102	0	0	100.00	1.00	1.00	1.00	1.00
12	0	0	0	0	0	0	0	0	0	0	0	6	0	0	0	0	0	0	6	102	0	0	100.00	1.00	1.00	1.00	1.00
13	0	0	0	0	0	0	0	0	0	0	0	0	6	0	0	0	0	0	6	102	0	0	100.00	1.00	1.00	1.00	1.00
14	0	0	0	0	0	0	0	0	0	0	0	0	0	6	0	0	0	0	6	102	0	0	100.00	1.00	1.00	1.00	1.00
15	0	0	0	0	0	0	0	0	0	0	0	0	0	0	6	0	0	0	6	102	0	0	100.00	1.00	1.00	1.00	1.00
16	0	0	0	0	0	0	0	0	0	0	0	0	0	0	0	6	0	0	6	102	0	0	100.00	1.00	1.00	1.00	1.00
17	0	0	0	0	0	0	0	0	0	0	0	0	0	0	0	0	6	0	6	102	0	0	100.00	1.00	1.00	1.00	1.00
18	0	0	0	0	0	0	0	0	0	0	0	0	0	0	0	0	0	6	6	102	0	0	100.00	1.00	1.00	1.00	1.00

	Validation set																										
**Class**	**1**	**2**	**3**	**4**	**5**	**6**	**7**	**8**	**9**	**10**	**11**	**12**	**13**	**14**	**15**	**16**	**17**	**18**	**TP**	**TN**	**FP**	**FN**	**ACC**	**SE**	**SP**	**MCC**	**EFF**
1	4	0	0	0	0	0	0	0	0	0	0	0	0	0	0	0	0	0	4	68	0	0	100.00	1.00	1.00	1.00	1.00
2	0	4	0	0	0	0	0	0	0	0	0	0	0	0	0	0	0	0	4	68	0	0	100.00	1.00	1.00	1.00	1.00
3	0	0	4	0	0	0	0	0	0	0	0	0	0	0	0	0	0	0	4	68	0	0	100.00	1.00	1.00	1.00	1.00
4	0	0	0	4	0	0	0	0	0	0	0	0	0	0	0	0	0	0	4	68	0	0	100.00	1.00	1.00	1.00	1.00
5	0	0	0	0	4	0	0	0	0	0	0	0	0	0	0	0	0	0	4	67	1	0	98.61	1.00	0.99	0.89	0.99
6	0	0	0	0	0	4	0	0	0	0	0	0	0	0	0	0	0	0	4	68	0	0	100.00	1.00	1.00	1.00	1.00
7	0	0	0	0	0	0	4	0	0	0	0	0	0	0	0	0	0	0	4	68	0	0	100.00	1.00	1.00	1.00	1.00
8	0	0	0	0	0	0	0	4	0	0	0	0	0	0	0	0	0	0	4	68	0	0	100.00	1.00	1.00	1.00	1.00
9	0	0	0	0	0	0	0	0	4	0	0	0	0	0	0	0	0	0	4	68	0	0	100.00	1.00	1.00	1.00	1.00
10	0	0	0	0	0	0	0	0	0	4	0	0	0	0	0	0	0	0	4	68	0	0	100.00	1.00	1.00	1.00	1.00
11	0	0	0	0	0	0	0	0	0	0	4	0	0	0	0	0	0	0	4	68	0	0	100.00	1.00	1.00	1.00	1.00
12	0	0	0	0	0	0	0	0	0	0	0	4	0	0	0	0	0	0	4	68	0	0	100.00	1.00	1.00	1.00	1.00
13	0	0	0	0	0	0	0	0	0	0	0	0	4	0	0	0	0	0	4	68	0	0	100.00	1.00	1.00	1.00	1.00
14	0	0	0	0	0	0	0	0	0	0	0	0	0	4	0	0	0	0	4	68	0	0	100.00	1.00	1.00	1.00	1.00
15	0	0	0	0	0	0	0	0	0	0	0	0	0	0	4	0	0	0	4	68	0	0	100.00	1.00	1.00	1.00	1.00
16	0	0	0	0	0	0	0	0	0	0	0	0	0	0	0	4	0	0	4	68	0	0	100.00	1.00	1.00	1.00	1.00
17	0	0	0	0	0	0	0	0	0	0	0	0	0	0	0	0	4	0	4	68	0	0	100.00	1.00	1.00	1.00	1.00
18	0	0	0	0	1	0	0	0	0	0	0	0	0	0	0	0	0	3	3	68	0	1	98.61	0.75	1.00	0.86	0.87

Table S38. Confusion matrixes of the calibration set and validation set of SG-Ven-NIR- SVM

Calibration set																											
**Class**	**1**	**2**	**3**	**4**	**5**	**6**	**7**	**8**	**9**	**10**	**11**	**12**	**13**	**14**	**15**	**16**	**17**	**18**	**TP**	**TN**	**FP**	**FN**	**ACC**	**SE**	**SP**	**MCC**	**EFF**
1	6	0	0	0	0	0	0	0	0	0	0	0	0	0	0	0	0	0	6	102	0	0	100.00	1.00	1.00	1.00	1.00
2	0	6	0	0	0	0	0	0	0	0	0	0	0	0	0	0	0	0	6	102	0	0	100.00	1.00	1.00	1.00	1.00
3	0	0	6	0	0	0	0	0	0	0	0	0	0	0	0	0	0	0	6	102	0	0	100.00	1.00	1.00	1.00	1.00
4	0	0	0	6	0	0	0	0	0	0	0	0	0	0	0	0	0	0	6	102	0	0	100.00	1.00	1.00	1.00	1.00
5	0	0	0	0	6	0	0	0	0	0	0	0	0	0	0	0	0	0	6	102	0	0	100.00	1.00	1.00	1.00	1.00
6	0	0	0	0	0	6	0	0	0	0	0	0	0	0	0	0	0	0	6	102	0	0	100.00	1.00	1.00	1.00	1.00
7	0	0	0	0	0	0	6	0	0	0	0	0	0	0	0	0	0	0	6	102	0	0	100.00	1.00	1.00	1.00	1.00
8	0	0	0	0	0	0	0	6	0	0	0	0	0	0	0	0	0	0	6	102	0	0	100.00	1.00	1.00	1.00	1.00
9	0	0	0	0	0	0	0	0	6	0	0	0	0	0	0	0	0	0	6	102	0	0	100.00	1.00	1.00	1.00	1.00
10	0	0	0	0	0	0	0	0	0	6	0	0	0	0	0	0	0	0	6	102	0	0	100.00	1.00	1.00	1.00	1.00
11	0	0	0	0	0	0	0	0	0	0	6	0	0	0	0	0	0	0	6	102	0	0	100.00	1.00	1.00	1.00	1.00
12	0	0	0	0	0	0	0	0	0	0	0	6	0	0	0	0	0	0	6	102	0	0	100.00	1.00	1.00	1.00	1.00
13	0	0	0	0	0	0	0	0	0	0	0	0	6	0	0	0	0	0	6	102	0	0	100.00	1.00	1.00	1.00	1.00
14	0	0	0	0	0	0	0	0	0	0	0	0	0	6	0	0	0	0	6	102	0	0	100.00	1.00	1.00	1.00	1.00
15	0	0	0	0	0	0	0	0	0	0	0	0	0	0	6	0	0	0	6	102	0	0	100.00	1.00	1.00	1.00	1.00
16	0	0	0	0	0	0	0	0	0	0	0	0	0	0	0	6	0	0	6	102	0	0	100.00	1.00	1.00	1.00	1.00
17	0	0	0	0	0	0	0	0	0	0	0	0	0	0	0	0	6	0	6	102	0	0	100.00	1.00	1.00	1.00	1.00
18	0	0	0	0	0	0	0	0	0	0	0	0	0	0	0	0	0	6	6	102	0	0	100.00	1.00	1.00	1.00	1.00

Validation set																											
**Class**	**1**	**2**	**3**	**4**	**5**	**6**	**7**	**8**	**9**	**10**	**11**	**12**	**13**	**14**	**15**	**16**	**17**	**18**	**TP**	**TN**	**FP**	**FN**	**ACC**	**SE**	**SP**	**MCC**	**EFF**
1	4	0	0	0	0	0	0	0	0	0	0	0	0	0	0	0	0	0	4	68	0	0	100.00	1.00	1.00	1.00	1.00
2	0	4	0	0	0	0	0	0	0	0	0	0	0	0	0	0	0	0	4	68	0	0	100.00	1.00	1.00	1.00	1.00
3	0	0	4	0	0	0	0	0	0	0	0	0	0	0	0	0	0	0	4	68	0	0	100.00	1.00	1.00	1.00	1.00
4	0	0	0	4	0	0	0	0	0	0	0	0	0	0	0	0	0	0	4	68	0	0	100.00	1.00	1.00	1.00	1.00
5	0	0	0	0	3	0	0	0	0	0	0	0	0	0	0	0	0	1	3	67	1	1	97.22	0.75	0.99	0.74	0.86
6	0	0	0	0	0	4	0	0	0	0	0	0	0	0	0	0	0	0	4	68	0	0	100.00	1.00	1.00	1.00	1.00
7	0	0	0	0	0	0	4	0	0	0	0	0	0	0	0	0	0	0	4	68	0	0	100.00	1.00	1.00	1.00	1.00
8	0	0	0	0	0	0	0	4	0	0	0	0	0	0	0	0	0	0	4	68	0	0	100.00	1.00	1.00	1.00	1.00
9	0	0	0	0	0	0	0	0	4	0	0	0	0	0	0	0	0	0	4	68	0	0	100.00	1.00	1.00	1.00	1.00
10	0	0	0	0	0	0	0	0	0	4	0	0	0	0	0	0	0	0	4	68	0	0	100.00	1.00	1.00	1.00	1.00
11	0	0	0	0	0	0	0	0	0	0	4	0	0	0	0	0	0	0	4	68	0	0	100.00	1.00	1.00	1.00	1.00
12	0	0	0	0	0	0	0	0	0	0	0	4	0	0	0	0	0	0	4	68	0	0	100.00	1.00	1.00	1.00	1.00
13	0	0	0	0	0	0	0	0	0	0	0	0	4	0	0	0	0	0	4	68	0	0	100.00	1.00	1.00	1.00	1.00
14	0	0	0	0	0	0	0	0	0	0	0	0	0	4	0	0	0	0	4	68	0	0	100.00	1.00	1.00	1.00	1.00
15	0	0	0	0	0	0	0	0	0	0	0	0	0	0	4	0	0	0	4	68	0	0	100.00	1.00	1.00	1.00	1.00
16	0	0	0	0	0	0	0	0	0	0	0	0	0	0	0	4	0	0	4	68	0	0	100.00	1.00	1.00	1.00	1.00
17	0	0	0	0	0	0	0	0	0	0	0	0	0	0	0	0	4	0	4	68	0	0	100.00	1.00	1.00	1.00	1.00
18	0	0	0	0	1	0	0	0	0	0	0	0	0	0	0	0	0	3	3	67	1	1	97.22	0.75	0.99	0.74	0.86

Table S39. Confusion matrixes of the calibration set and validation set of SG-Ven-NIR- KNN

Calibration set																												
**Class**	**1**	**2**	**3**	**4**	**5**	**6**	**7**	**8**	**9**	**10**	**11**	**12**	**13**	**14**	**15**	**16**	**17**	**18**	**TP**	**TN**	**FP**	**FN**	**ACC**	**SE**	**SP**	**MCC**	**EFF**
1	6	0	0	0	0	0	0	0	0	0	0	0	0	0	0	0	0	0	6	102	0	0	100.00	1.00	1.00	1.00	1.00
2	0	6	0	0	0	0	0	0	0	0	0	0	0	0	0	0	0	0	6	102	0	0	100.00	1.00	1.00	1.00	1.00
3	0	0	6	0	0	0	0	0	0	0	0	0	0	0	0	0	0	0	6	102	0	0	100.00	1.00	1.00	1.00	1.00
4	0	0	0	6	0	0	0	0	0	0	0	0	0	0	0	0	0	0	6	102	0	0	100.00	1.00	1.00	1.00	1.00
5	0	0	0	0	6	0	0	0	0	0	0	0	0	0	0	0	0	0	6	102	0	0	100.00	1.00	1.00	1.00	1.00
6	0	0	0	0	0	6	0	0	0	0	0	0	0	0	0	0	0	0	6	102	0	0	100.00	1.00	1.00	1.00	1.00
7	0	0	0	0	0	0	6	0	0	0	0	0	0	0	0	0	0	0	6	102	0	0	100.00	1.00	1.00	1.00	1.00
8	0	0	0	0	0	0	0	6	0	0	0	0	0	0	0	0	0	0	6	102	0	0	100.00	1.00	1.00	1.00	1.00
9	0	0	0	0	0	0	0	0	6	0	0	0	0	0	0	0	0	0	6	102	0	0	100.00	1.00	1.00	1.00	1.00
10	0	0	0	0	0	0	0	0	0	6	0	0	0	0	0	0	0	0	6	102	0	0	100.00	1.00	1.00	1.00	1.00
11	0	0	0	0	0	0	0	0	0	0	6	0	0	0	0	0	0	0	6	102	0	0	100.00	1.00	1.00	1.00	1.00
12	0	0	0	0	0	0	0	0	0	0	0	6	0	0	0	0	0	0	6	102	0	0	100.00	1.00	1.00	1.00	1.00
13	0	0	0	0	0	0	0	0	0	0	0	0	6	0	0	0	0	0	6	102	0	0	100.00	1.00	1.00	1.00	1.00
14	0	0	0	0	0	0	0	0	0	0	0	0	0	6	0	0	0	0	6	102	0	0	100.00	1.00	1.00	1.00	1.00
15	0	0	0	0	0	0	0	0	0	0	0	0	0	0	6	0	0	0	6	102	0	0	100.00	1.00	1.00	1.00	1.00
16	0	0	0	0	0	0	0	0	0	0	0	0	0	0	0	6	0	0	6	102	0	0	100.00	1.00	1.00	1.00	1.00
17	0	0	0	0	0	0	0	0	0	0	0	0	0	0	0	0	6	0	6	102	0	0	100.00	1.00	1.00	1.00	1.00
18	0	0	0	0	0	0	0	0	0	0	0	0	0	0	0	0	0	6	6	102	0	0	100.00	1.00	1.00	1.00	1.00

Validation set																												
**Class**	**1**	**2**	**3**	**4**	**5**	**6**	**7**	**8**	**9**	**10**	**11**	**12**	**13**	**14**	**15**	**16**	**17**	**18**	**TP**	**TN**	**FP**	**FN**	**ACC**	**SE**	**SP**	**MCC**	**EFF**
1	4	0	0	0	0	0	0	0	0	0	0	0	0	0	0	0	0	0	4	68	0	0	100.00	1.00	1.00	1.00	1.00
2	0	4	0	0	0	0	0	0	0	0	0	0	0	0	0	0	0	0	4	68	0	0	100.00	1.00	1.00	1.00	1.00
3	0	0	4	0	0	0	0	0	0	0	0	0	0	0	0	0	0	0	4	68	0	0	100.00	1.00	1.00	1.00	1.00
4	0	0	0	4	0	0	0	0	0	0	0	0	0	0	0	0	0	0	4	68	0	0	100.00	1.00	1.00	1.00	1.00
5	0	0	0	0	3	0	0	0	0	0	0	0	0	0	0	0	0	1	3	67	1	1	97.22	0.75	0.99	0.74	0.86
6	0	0	0	0	0	4	0	0	0	0	0	0	0	0	0	0	0	0	4	68	0	0	100.00	1.00	1.00	1.00	1.00
7	0	0	0	0	0	0	4	0	0	0	0	0	0	0	0	0	0	0	4	68	0	0	100.00	1.00	1.00	1.00	1.00
8	0	0	0	0	0	0	0	4	0	0	0	0	0	0	0	0	0	0	4	68	0	0	100.00	1.00	1.00	1.00	1.00
9	0	0	0	0	0	0	0	0	4	0	0	0	0	0	0	0	0	0	4	68	0	0	100.00	1.00	1.00	1.00	1.00
10	0	0	0	0	0	0	0	0	0	4	0	0	0	0	0	0	0	0	4	68	0	0	100.00	1.00	1.00	1.00	1.00
11	0	0	0	0	0	0	0	0	0	0	4	0	0	0	0	0	0	0	4	68	0	0	100.00	1.00	1.00	1.00	1.00
12	0	0	0	0	0	0	0	0	0	0	0	4	0	0	0	0	0	0	4	67	1	0	98.61	1.00	0.99	0.89	0.99
13	0	0	0	0	0	0	0	0	0	0	0	0	4	0	0	0	0	0	4	68	0	0	100.00	1.00	1.00	1.00	1.00
14	0	0	0	0	0	0	0	0	0	0	0	0	0	4	0	0	0	0	4	68	0	0	100.00	1.00	1.00	1.00	1.00
15	0	0	0	0	0	0	0	0	0	0	0	0	0	0	4	0	0	0	4	68	0	0	100.00	1.00	1.00	1.00	1.00
16	0	0	0	0	0	0	0	0	0	0	0	1	0	0	0	3	0	0	3	68	0	1	98.61	0.75	1.00	0.86	0.87
17	0	0	0	0	0	0	0	0	0	0	0	0	0	0	0	0	4	0	4	68	0	0	100.00	1.00	1.00	1.00	1.00
18	0	0	0	0	1	0	0	0	0	0	0	0	0	0	0	0	0	3	3	67	1	1	97.22	0.75	0.99	0.74	0.86

Table S40. Confusion matrixes of the calibration set and validation set of SG-Ven-MIR- RF

Calibration set																												
**Class**	**1**	**2**	**3**	**4**	**5**	**6**	**7**	**8**	**9**	**10**	**11**	**12**	**13**	**14**	**15**	**16**	**17**	**18**	**TP**	**TN**	**FP**	**FN**	**ACC**	**SE**	**SP**	**MCC**	**EFF**
1	6	0	0	0	0	0	0	0	0	0	0	0	0	0	0	0	0	0	6	102	0	0	100.00	1.00	1.00	1.00	1.00
2	0	6	0	0	0	0	0	0	0	0	0	0	0	0	0	0	0	0	6	102	0	0	100.00	1.00	1.00	1.00	1.00
3	0	0	6	0	0	0	0	0	0	0	0	0	0	0	0	0	0	0	6	102	0	0	100.00	1.00	1.00	1.00	1.00
4	0	0	0	6	0	0	0	0	0	0	0	0	0	0	0	0	0	0	6	102	0	0	100.00	1.00	1.00	1.00	1.00
5	0	0	0	0	6	0	0	0	0	0	0	0	0	0	0	0	0	0	6	102	0	0	100.00	1.00	1.00	1.00	1.00
6	0	0	0	0	0	6	0	0	0	0	0	0	0	0	0	0	0	0	6	102	0	0	100.00	1.00	1.00	1.00	1.00
7	0	0	0	0	0	0	6	0	0	0	0	0	0	0	0	0	0	0	6	102	0	0	100.00	1.00	1.00	1.00	1.00
8	0	0	0	0	0	0	0	6	0	0	0	0	0	0	0	0	0	0	6	102	0	0	100.00	1.00	1.00	1.00	1.00
9	0	0	0	0	0	0	0	0	6	0	0	0	0	0	0	0	0	0	6	102	0	0	100.00	1.00	1.00	1.00	1.00
10	0	0	0	0	0	0	0	0	0	6	0	0	0	0	0	0	0	0	6	102	0	0	100.00	1.00	1.00	1.00	1.00
11	0	0	0	0	0	0	0	0	0	0	6	0	0	0	0	0	0	0	6	102	0	0	100.00	1.00	1.00	1.00	1.00
12	0	0	0	0	0	0	0	0	0	0	0	6	0	0	0	0	0	0	6	102	0	0	100.00	1.00	1.00	1.00	1.00
13	0	0	0	0	0	0	0	0	0	0	0	0	6	0	0	0	0	0	6	102	0	0	100.00	1.00	1.00	1.00	1.00
14	0	0	0	0	0	0	0	0	0	0	0	0	0	6	0	0	0	0	6	102	0	0	100.00	1.00	1.00	1.00	1.00
15	0	0	0	0	0	0	0	0	0	0	0	0	0	0	6	0	0	0	6	102	0	0	100.00	1.00	1.00	1.00	1.00
16	0	0	0	0	0	0	0	0	0	0	0	0	0	0	0	6	0	0	6	102	0	0	100.00	1.00	1.00	1.00	1.00
17	0	0	0	0	0	0	0	0	0	0	0	0	0	0	0	0	6	0	6	102	0	0	100.00	1.00	1.00	1.00	1.00
18	0	0	0	0	0	0	0	0	0	0	0	0	0	0	0	0	0	6	6	102	0	0	100.00	1.00	1.00	1.00	1.00

Validation set																												
**Class**	**1**	**2**	**3**	**4**	**5**	**6**	**7**	**8**	**9**	**10**	**11**	**12**	**13**	**14**	**15**	**16**	**17**	**18**	**TP**	**TN**	**FP**	**FN**	**ACC**	**SE**	**SP**	**MCC**	**EFF**
1	4	0	0	0	0	0	0	0	0	0	0	0	0	0	0	0	0	0	4	67	1	0	98.61	1.00	0.99	0.89	0.99
2	0	4	0	0	0	0	0	0	0	0	0	0	0	0	0	0	0	0	4	68	0	0	100.00	1.00	1.00	1.00	1.00
3	0	0	4	0	0	0	0	0	0	0	0	0	0	0	0	0	0	0	4	68	0	0	100.00	1.00	1.00	1.00	1.00
4	0	0	0	4	0	0	0	0	0	0	0	0	0	0	0	0	0	0	4	68	0	0	100.00	1.00	1.00	1.00	1.00
5	0	0	0	0	4	0	0	0	0	0	0	0	0	0	0	0	0	0	4	68	0	0	100.00	1.00	1.00	1.00	1.00
6	1	0	0	0	0	3	0	0	0	0	0	0	0	0	0	0	0	0	3	68	0	1	98.61	0.75	1.00	0.86	0.87
7	0	0	0	0	0	0	4	0	0	0	0	0	0	0	0	0	0	0	4	68	0	0	100.00	1.00	1.00	1.00	1.00
8	0	0	0	0	0	0	0	4	0	0	0	0	0	0	0	0	0	0	4	67	1	0	98.61	1.00	0.99	0.89	0.99
9	0	0	0	0	0	0	0	1	3	0	0	0	0	0	0	0	0	0	3	68	0	1	98.61	0.75	1.00	0.86	0.87
10	0	0	0	0	0	0	0	0	0	4	0	0	0	0	0	0	0	0	4	67	1	0	98.61	1.00	0.99	0.89	0.99
11	0	0	0	0	0	0	0	0	0	0	4	0	0	0	0	0	0	0	4	67	1	0	98.61	1.00	0.99	0.89	0.99
12	0	0	0	0	0	0	0	0	0	0	1	3	0	0	0	0	0	0	3	68	0	1	98.61	0.75	1.00	0.86	0.87
13	0	0	0	0	0	0	0	0	0	0	0	0	4	0	0	0	0	0	4	68	0	0	100.00	1.00	1.00	1.00	1.00
14	0	0	0	0	0	0	0	0	0	0	0	0	0	4	0	0	0	0	4	68	0	0	100.00	1.00	1.00	1.00	1.00
15	0	0	0	0	0	0	0	0	0	0	0	0	0	0	4	0	0	0	4	68	0	0	100.00	1.00	1.00	1.00	1.00
16	0	0	0	0	0	0	0	0	0	1	0	0	0	0	0	3	0	0	3	68	0	1	98.61	0.75	1.00	0.86	0.87
17	0	0	0	0	0	0	0	0	0	0	0	0	0	0	0	0	4	0	4	68	0	0	100.00	1.00	1.00	1.00	1.00
18	0	0	0	0	0	0	0	0	0	0	0	0	0	0	0	0	0	4	4	68	0	0	100.00	1.00	1.00	1.00	1.00

Table S41. Confusion matrixes of the calibration set and validation set of SG-Ven-MIR- SVM

Calibration set																												
**Class**	**1**	**2**	**3**	**4**	**5**	**6**	**7**	**8**	**9**	**10**	**11**	**12**	**13**	**14**	**15**	**16**	**17**	**18**	**TP**	**TN**	**FP**	**FN**	**ACC**	**SE**	**SP**	**MCC**	**EFF**
1	6	0	0	0	0	0	0	0	0	0	0	0	0	0	0	0	0	0	6	102	0	0	100.00	1.00	1.00	1.00	1.00
2	0	6	0	0	0	0	0	0	0	0	0	0	0	0	0	0	0	0	6	102	0	0	100.00	1.00	1.00	1.00	1.00
3	0	0	6	0	0	0	0	0	0	0	0	0	0	0	0	0	0	0	6	102	0	0	100.00	1.00	1.00	1.00	1.00
4	0	0	0	6	0	0	0	0	0	0	0	0	0	0	0	0	0	0	6	102	0	0	100.00	1.00	1.00	1.00	1.00
5	0	0	0	0	6	0	0	0	0	0	0	0	0	0	0	0	0	0	6	102	0	0	100.00	1.00	1.00	1.00	1.00
6	0	0	0	0	0	6	0	0	0	0	0	0	0	0	0	0	0	0	6	102	0	0	100.00	1.00	1.00	1.00	1.00
7	0	0	0	0	0	0	6	0	0	0	0	0	0	0	0	0	0	0	6	102	0	0	100.00	1.00	1.00	1.00	1.00
8	0	0	0	0	0	0	0	6	0	0	0	0	0	0	0	0	0	0	6	102	0	0	100.00	1.00	1.00	1.00	1.00
9	0	0	0	0	0	0	0	0	6	0	0	0	0	0	0	0	0	0	6	102	0	0	100.00	1.00	1.00	1.00	1.00
10	0	0	0	0	0	0	0	0	0	6	0	0	0	0	0	0	0	0	6	102	0	0	100.00	1.00	1.00	1.00	1.00
11	0	0	0	0	0	0	0	0	0	0	6	0	0	0	0	0	0	0	6	102	0	0	100.00	1.00	1.00	1.00	1.00
12	0	0	0	0	0	0	0	0	0	0	0	6	0	0	0	0	0	0	6	102	0	0	100.00	1.00	1.00	1.00	1.00
13	0	0	0	0	0	0	0	0	0	0	0	0	6	0	0	0	0	0	6	102	0	0	100.00	1.00	1.00	1.00	1.00
14	0	0	0	0	0	0	0	0	0	0	0	0	0	6	0	0	0	0	6	102	0	0	100.00	1.00	1.00	1.00	1.00
15	0	0	0	0	0	0	0	0	0	0	0	0	0	0	6	0	0	0	6	102	0	0	100.00	1.00	1.00	1.00	1.00
16	0	0	0	0	0	0	0	0	0	0	0	0	0	0	0	6	0	0	6	102	0	0	100.00	1.00	1.00	1.00	1.00
17	0	0	0	0	0	0	0	0	0	0	0	0	0	0	0	0	6	0	6	102	0	0	100.00	1.00	1.00	1.00	1.00
18	0	0	0	0	0	0	0	0	0	0	0	0	0	0	0	0	0	6	6	102	0	0	100.00	1.00	1.00	1.00	1.00

Validation set

Class	1	2	3	4	5	6	7	8	9	10	11	12	13	14	15	16	17	18	TP	TN	FP	FN	ACC	SE	SP	MCC	EFF
1	4	0	0	0	0	0	0	0	0	0	0	0	0	0	0	0	0	0	4	68	0	0	100.00	1.00	1.00	1.00	1.00
2	0	4	0	0	0	0	0	0	0	0	0	0	0	0	0	0	0	0	4	68	0	0	100.00	1.00	1.00	1.00	1.00
3	0	0	4	0	0	0	0	0	0	0	0	0	0	0	0	0	0	0	4	68	0	0	100.00	1.00	1.00	1.00	1.00
4	0	0	0	4	0	0	0	0	0	0	0	0	0	0	0	0	0	0	4	68	0	0	100.00	1.00	1.00	1.00	1.00
5	0	0	0	0	4	0	0	0	0	0	0	0	0	0	0	0	0	0	4	68	0	0	100.00	1.00	1.00	1.00	1.00
6	0	0	0	0	0	4	0	0	0	0	0	0	0	0	0	0	0	0	4	68	0	0	100.00	1.00	1.00	1.00	1.00
7	0	0	0	0	0	0	4	0	0	0	0	0	0	0	0	0	0	0	4	68	0	0	100.00	1.00	1.00	1.00	1.00
8	0	0	0	0	0	0	0	4	0	0	0	0	0	0	0	0	0	0	4	68	0	0	100.00	1.00	1.00	1.00	1.00
9	0	0	0	0	0	0	0	0	4	0	0	0	0	0	0	0	0	0	4	68	0	0	100.00	1.00	1.00	1.00	1.00
10	0	0	0	0	0	0	0	0	0	4	0	0	0	0	0	0	0	0	4	68	0	0	100.00	1.00	1.00	1.00	1.00
11	0	0	0	0	0	0	0	0	0	0	4	0	0	0	0	0	0	0	4	68	0	0	100.00	1.00	1.00	1.00	1.00
12	0	0	0	0	0	0	0	0	0	0	0	4	0	0	0	0	0	0	4	68	0	0	100.00	1.00	1.00	1.00	1.00
13	0	0	0	0	0	0	0	0	0	0	0	0	4	0	0	0	0	0	4	68	0	0	100.00	1.00	1.00	1.00	1.00
14	0	0	0	0	0	0	0	0	0	0	0	0	0	4	0	0	0	0	4	68	0	0	100.00	1.00	1.00	1.00	1.00
15	0	0	0	0	0	0	0	0	0	0	0	0	0	0	4	0	0	0	4	68	0	0	100.00	1.00	1.00	1.00	1.00
16	0	0	0	0	0	0	0	0	0	0	0	0	0	0	0	4	0	0	4	68	0	0	100.00	1.00	1.00	1.00	1.00
17	0	0	0	0	0	0	0	0	0	0	0	0	0	0	0	0	4	0	4	68	0	0	100.00	1.00	1.00	1.00	1.00
18	0	0	0	0	0	0	0	0	0	0	0	0	0	0	0	0	0	4	4	68	0	0	100.00	1.00	1.00	1.00	1.00

Table S42. Confusion matrixes of the calibration set and validation set of SG-Ven-MIR- KNN

Calibration set																												
**Class**	**1**	**2**	**3**	**4**	**5**	**6**	**7**	**8**	**9**	**10**	**11**	**12**	**13**	**14**	**15**	**16**	**17**	**18**	**TP**	**TN**	**FP**	**FN**	**ACC**	**SE**	**SP**	**MCC**	**EFF**
1	6	0	0	0	0	0	0	0	0	0	0	0	0	0	0	0	0	0	6	102	0	0	100.00	1.00	1.00	1.00	1.00
2	0	6	0	0	0	0	0	0	0	0	0	0	0	0	0	0	0	0	6	102	0	0	100.00	1.00	1.00	1.00	1.00
3	0	0	6	0	0	0	0	0	0	0	0	0	0	0	0	0	0	0	6	102	0	0	100.00	1.00	1.00	1.00	1.00
4	0	0	0	6	0	0	0	0	0	0	0	0	0	0	0	0	0	0	6	102	0	0	100.00	1.00	1.00	1.00	1.00
5	0	0	0	0	6	0	0	0	0	0	0	0	0	0	0	0	0	0	6	102	0	0	100.00	1.00	1.00	1.00	1.00
6	0	0	0	0	0	6	0	0	0	0	0	0	0	0	0	0	0	0	6	102	0	0	100.00	1.00	1.00	1.00	1.00
7	0	0	0	0	0	0	6	0	0	0	0	0	0	0	0	0	0	0	6	102	0	0	100.00	1.00	1.00	1.00	1.00
8	0	0	0	0	0	0	0	6	0	0	0	0	0	0	0	0	0	0	6	102	0	0	100.00	1.00	1.00	1.00	1.00
9	0	0	0	0	0	0	0	0	6	0	0	0	0	0	0	0	0	0	6	102	0	0	100.00	1.00	1.00	1.00	1.00
10	0	0	0	0	0	0	0	0	0	6	0	0	0	0	0	0	0	0	6	102	0	0	100.00	1.00	1.00	1.00	1.00
11	0	0	0	0	0	0	0	0	0	0	6	0	0	0	0	0	0	0	6	102	0	0	100.00	1.00	1.00	1.00	1.00
12	0	0	0	0	0	0	0	0	0	0	0	6	0	0	0	0	0	0	6	102	0	0	100.00	1.00	1.00	1.00	1.00
13	0	0	0	0	0	0	0	0	0	0	0	0	6	0	0	0	0	0	6	102	0	0	100.00	1.00	1.00	1.00	1.00
14	0	0	0	0	0	0	0	0	0	0	0	0	0	6	0	0	0	0	6	102	0	0	100.00	1.00	1.00	1.00	1.00
15	0	0	0	0	0	0	0	0	0	0	0	0	0	0	6	0	0	0	6	102	0	0	100.00	1.00	1.00	1.00	1.00
16	0	0	0	0	0	0	0	0	0	0	0	0	0	0	0	6	0	0	6	102	0	0	100.00	1.00	1.00	1.00	1.00
17	0	0	0	0	0	0	0	0	0	0	0	0	0	0	0	0	6	0	6	102	0	0	100.00	1.00	1.00	1.00	1.00
18	0	0	0	0	0	0	0	0	0	0	0	0	0	0	0	0	0	6	6	102	0	0	100.00	1.00	1.00	1.00	1.00

Validation set																												
**Class**	**1**	**2**	**3**	**4**	**5**	**6**	**7**	**8**	**9**	**10**	**11**	**12**	**13**	**14**	**15**	**16**	**17**	**18**	**TP**	**TN**	**FP**	**FN**	**ACC**	**SE**	**SP**	**MCC**	**EFF**
1	4	0	0	0	0	0	0	0	0	0	0	0	0	0	0	0	0	0	4	67	1	0	98.61	1.00	0.99	0.89	0.99
2	0	4	0	0	0	0	0	0	0	0	0	0	0	0	0	0	0	0	4	68	0	0	100.00	1.00	1.00	1.00	1.00
3	0	0	4	0	0	0	0	0	0	0	0	0	0	0	0	0	0	0	4	68	0	0	100.00	1.00	1.00	1.00	1.00
4	0	0	0	4	0	0	0	0	0	0	0	0	0	0	0	0	0	0	4	68	0	0	100.00	1.00	1.00	1.00	1.00
5	0	0	0	0	4	0	0	0	0	0	0	0	0	0	0	0	0	0	4	68	0	0	100.00	1.00	1.00	1.00	1.00
6	1	0	0	0	0	3	0	0	0	0	0	0	0	0	0	0	0	0	3	68	0	1	98.61	0.75	1.00	0.86	0.87
7	0	0	0	0	0	0	4	0	0	0	0	0	0	0	0	0	0	0	4	68	0	0	100.00	1.00	1.00	1.00	1.00
8	0	0	0	0	0	0	0	4	0	0	0	0	0	0	0	0	0	0	4	66	2	0	97.22	1.00	0.97	0.80	0.99
9	0	0	0	0	0	0	0	2	2	0	0	0	0	0	0	0	0	0	2	68	0	2	97.22	0.50	1.00	0.70	0.71
10	0	0	0	0	0	0	0	0	0	4	0	0	0	0	0	0	0	0	4	68	0	0	100.00	1.00	1.00	1.00	1.00
11	0	0	0	0	0	0	0	0	0	0	4	0	0	0	0	0	0	0	4	66	2	0	97.22	1.00	0.97	0.80	0.99
12	0	0	0	0	0	0	0	0	0	0	1	3	0	0	0	0	0	0	3	68	0	1	98.61	0.75	1.00	0.86	0.87
13	0	0	0	0	0	0	0	0	0	0	0	0	4	0	0	0	0	0	4	68	0	0	100.00	1.00	1.00	1.00	1.00
14	0	0	0	0	0	0	0	0	0	0	0	0	0	4	0	0	0	0	4	68	0	0	100.00	1.00	1.00	1.00	1.00
15	0	0	0	0	0	0	0	0	0	0	0	0	0	0	4	0	0	0	4	67	1	0	98.61	1.00	0.99	0.89	0.99
16	0	0	0	0	0	0	0	0	0	0	1	0	0	0	1	1	0	1	1	68	0	3	95.83	0.25	1.00	0.49	0.50
17	0	0	0	0	0	0	0	0	0	0	0	0	0	0	0	0	4	0	4	68	0	0	100.00	1.00	1.00	1.00	1.00
18	0	0	0	0	0	0	0	0	0	0	0	0	0	0	0	0	0	4	4	67	1	0	98.61	1.00	0.99	0.89	0.99

Table S43. Confusion matrixes of the calibration set and validation set of Low-RF

Calibration set																												
**Class**	**1**	**2**	**3**	**4**	**5**	**6**	**7**	**8**	**9**	**10**	**11**	**12**	**13**	**14**	**15**	**16**	**17**	**18**	**TP**	**TN**	**FP**	**FN**	**ACC**	**SE**	**SP**	**MCC**	**EFF**
1	6	0	0	0	0	0	0	0	0	0	0	0	0	0	0	0	0	0	6	102	0	0	100.00	1.00	1.00	1.00	1.00
2	0	6	0	0	0	0	0	0	0	0	0	0	0	0	0	0	0	0	6	102	0	0	100.00	1.00	1.00	1.00	1.00
3	0	0	6	0	0	0	0	0	0	0	0	0	0	0	0	0	0	0	6	102	0	0	100.00	1.00	1.00	1.00	1.00
4	0	0	0	6	0	0	0	0	0	0	0	0	0	0	0	0	0	0	6	102	0	0	100.00	1.00	1.00	1.00	1.00
5	0	0	0	0	6	0	0	0	0	0	0	0	0	0	0	0	0	0	6	102	0	0	100.00	1.00	1.00	1.00	1.00
6	0	0	0	0	0	6	0	0	0	0	0	0	0	0	0	0	0	0	6	102	0	0	100.00	1.00	1.00	1.00	1.00
7	0	0	0	0	0	0	6	0	0	0	0	0	0	0	0	0	0	0	6	102	0	0	100.00	1.00	1.00	1.00	1.00
8	0	0	0	0	0	0	0	6	0	0	0	0	0	0	0	0	0	0	6	102	0	0	100.00	1.00	1.00	1.00	1.00
9	0	0	0	0	0	0	0	0	6	0	0	0	0	0	0	0	0	0	6	102	0	0	100.00	1.00	1.00	1.00	1.00
10	0	0	0	0	0	0	0	0	0	6	0	0	0	0	0	0	0	0	6	102	0	0	100.00	1.00	1.00	1.00	1.00
11	0	0	0	0	0	0	0	0	0	0	6	0	0	0	0	0	0	0	6	102	0	0	100.00	1.00	1.00	1.00	1.00
12	0	0	0	0	0	0	0	0	0	0	0	6	0	0	0	0	0	0	6	102	0	0	100.00	1.00	1.00	1.00	1.00
13	0	0	0	0	0	0	0	0	0	0	0	0	6	0	0	0	0	0	6	102	0	0	100.00	1.00	1.00	1.00	1.00
14	0	0	0	0	0	0	0	0	0	0	0	0	0	6	0	0	0	0	6	102	0	0	100.00	1.00	1.00	1.00	1.00
15	0	0	0	0	0	0	0	0	0	0	0	0	0	0	6	0	0	0	6	102	0	0	100.00	1.00	1.00	1.00	1.00
16	0	0	0	0	0	0	0	0	0	0	0	0	0	0	0	6	0	0	6	102	0	0	100.00	1.00	1.00	1.00	1.00
17	0	0	0	0	0	0	0	0	0	0	0	0	0	0	0	0	6	0	6	102	0	0	100.00	1.00	1.00	1.00	1.00
18	0	0	0	0	0	0	0	0	0	0	0	0	0	0	0	0	0	6	6	102	0	0	100.00	1.00	1.00	1.00	1.00

Validation set																											
**Class**	**1**	**2**	**3**	**4**	**5**	**6**	**7**	**8**	**9**	**10**	**11**	**12**	**13**	**14**	**15**	**16**	**17**	**18**	**TP**	**TN**	**FP**	**FN**	**ACC**	**SE**	**SP**	**MCC**	**EFF**
1	4	0	0	0	0	0	0	0	0	0	0	0	0	0	0	0	0	0	4	68	0	0	100.00	1.00	1.00	1.00	1.00
2	0	4	0	0	0	0	0	0	0	0	0	0	0	0	0	0	0	0	4	68	0	0	100.00	1.00	1.00	1.00	1.00
3	0	0	4	0	0	0	0	0	0	0	0	0	0	0	0	0	0	0	4	67	1	0	98.61	1.00	0.99	0.89	0.99
4	0	0	0	4	0	0	0	0	0	0	0	0	0	0	0	0	0	0	4	68	0	0	100.00	1.00	1.00	1.00	1.00
5	0	0	0	0	4	0	0	0	0	0	0	0	0	0	0	0	0	0	4	68	0	0	100.00	1.00	1.00	1.00	1.00
6	0	0	0	0	0	4	0	0	0	0	0	0	0	0	0	0	0	0	4	68	0	0	100.00	1.00	1.00	1.00	1.00
7	0	0	0	0	0	0	4	0	0	0	0	0	0	0	0	0	0	0	4	68	0	0	100.00	1.00	1.00	1.00	1.00
8	0	0	0	0	0	0	0	3	1	0	0	0	0	0	0	0	0	0	3	68	0	1	98.61	0.75	1.00	0.86	0.87
9	0	0	0	0	0	0	0	0	4	0	0	0	0	0	0	0	0	0	4	67	1	0	98.61	1.00	0.99	0.89	0.99
10	0	0	0	0	0	0	0	0	0	4	0	0	0	0	0	0	0	0	4	68	0	0	100.00	1.00	1.00	1.00	1.00
11	0	0	0	0	0	0	0	0	0	0	4	0	0	0	0	0	0	0	4	68	0	0	100.00	1.00	1.00	1.00	1.00
12	0	0	0	0	0	0	0	0	0	0	0	4	0	0	0	0	0	0	4	68	0	0	100.00	1.00	1.00	1.00	1.00
13	0	0	0	0	0	0	0	0	0	0	0	0	4	0	0	0	0	0	4	68	0	0	100.00	1.00	1.00	1.00	1.00
14	0	0	0	0	0	0	0	0	0	0	0	0	0	4	0	0	0	0	4	68	0	0	100.00	1.00	1.00	1.00	1.00
15	0	0	0	0	0	0	0	0	0	0	0	0	0	0	4	0	0	0	4	68	0	0	100.00	1.00	1.00	1.00	1.00
16	0	0	0	0	0	0	0	0	0	0	0	0	0	0	0	4	0	0	4	68	0	0	100.00	1.00	1.00	1.00	1.00
17	0	0	0	0	0	0	0	0	0	0	0	0	0	0	0	0	4	0	4	68	0	0	100.00	1.00	1.00	1.00	1.00
18	0	0	1	0	0	0	0	0	0	0	0	0	0	0	0	0	0	3	3	68	0	1	98.61	0.75	1.00	0.86	0.87

Table S44. Confusion matrixes of the calibration set and validation set of Low-SVM

Calibration set																											
**Class**	**1**	**2**	**3**	**4**	**5**	**6**	**7**	**8**	**9**	**10**	**11**	**12**	**13**	**14**	**15**	**16**	**17**	**18**	**TP**	**TN**	**FP**	**FN**	**ACC**	**SE**	**SP**	**MCC**	**EFF**
1	6	0	0	0	0	0	0	0	0	0	0	0	0	0	0	0	0	0	6	102	0	0	100.00	1.00	1.00	1.00	1.00
2	0	6	0	0	0	0	0	0	0	0	0	0	0	0	0	0	0	0	6	102	0	0	100.00	1.00	1.00	1.00	1.00
3	0	0	6	0	0	0	0	0	0	0	0	0	0	0	0	0	0	0	6	102	0	0	100.00	1.00	1.00	1.00	1.00
4	0	0	0	6	0	0	0	0	0	0	0	0	0	0	0	0	0	0	6	102	0	0	100.00	1.00	1.00	1.00	1.00
5	0	0	0	0	6	0	0	0	0	0	0	0	0	0	0	0	0	0	6	102	0	0	100.00	1.00	1.00	1.00	1.00
6	0	0	0	0	0	6	0	0	0	0	0	0	0	0	0	0	0	0	6	102	0	0	100.00	1.00	1.00	1.00	1.00
7	0	0	0	0	0	0	6	0	0	0	0	0	0	0	0	0	0	0	6	102	0	0	100.00	1.00	1.00	1.00	1.00
8	0	0	0	0	0	0	0	6	0	0	0	0	0	0	0	0	0	0	6	102	0	0	100.00	1.00	1.00	1.00	1.00
9	0	0	0	0	0	0	0	0	6	0	0	0	0	0	0	0	0	0	6	102	0	0	100.00	1.00	1.00	1.00	1.00
10	0	0	0	0	0	0	0	0	0	6	0	0	0	0	0	0	0	0	6	102	0	0	100.00	1.00	1.00	1.00	1.00
11	0	0	0	0	0	0	0	0	0	0	6	0	0	0	0	0	0	0	6	102	0	0	100.00	1.00	1.00	1.00	1.00
12	0	0	0	0	0	0	0	0	0	0	0	6	0	0	0	0	0	0	6	102	0	0	100.00	1.00	1.00	1.00	1.00
13	0	0	0	0	0	0	0	0	0	0	0	0	6	0	0	0	0	0	6	102	0	0	100.00	1.00	1.00	1.00	1.00
14	0	0	0	0	0	0	0	0	0	0	0	0	0	6	0	0	0	0	6	102	0	0	100.00	1.00	1.00	1.00	1.00
15	0	0	0	0	0	0	0	0	0	0	0	0	0	0	6	0	0	0	6	102	0	0	100.00	1.00	1.00	1.00	1.00
16	0	0	0	0	0	0	0	0	0	0	0	0	0	0	0	6	0	0	6	102	0	0	100.00	1.00	1.00	1.00	1.00
17	0	0	0	0	0	0	0	0	0	0	0	0	0	0	0	0	6	0	6	102	0	0	100.00	1.00	1.00	1.00	1.00
18	0	0	0	0	0	0	0	0	0	0	0	0	0	0	0	0	0	6	6	102	0	0	100.00	1.00	1.00	1.00	1.00

Validation set																												
**Class**	**1**	**2**	**3**	**4**	**5**	**6**	**7**	**8**	**9**	**10**	**11**	**12**	**13**	**14**	**15**	**16**	**17**	**18**	**TP**	**TN**	**FP**	**FN**	**ACC**	**SE**	**SP**	**MCC**	**EFF**
1	4	0	0	0	0	0	0	0	0	0	0	0	0	0	0	0	0	0	4	68	0	0	100.00	1.00	1.00	1.00	1.00
2	0	4	0	0	0	0	0	0	0	0	0	0	0	0	0	0	0	0	4	68	0	0	100.00	1.00	1.00	1.00	1.00
3	0	0	4	0	0	0	0	0	0	0	0	0	0	0	0	0	0	0	4	68	0	0	100.00	1.00	1.00	1.00	1.00
4	0	0	0	4	0	0	0	0	0	0	0	0	0	0	0	0	0	0	4	68	0	0	100.00	1.00	1.00	1.00	1.00
5	0	0	0	0	4	0	0	0	0	0	0	0	0	0	0	0	0	0	4	68	0	0	100.00	1.00	1.00	1.00	1.00
6	0	0	0	0	0	4	0	0	0	0	0	0	0	0	0	0	0	0	4	68	0	0	100.00	1.00	1.00	1.00	1.00
7	0	0	0	0	0	0	4	0	0	0	0	0	0	0	0	0	0	0	4	68	0	0	100.00	1.00	1.00	1.00	1.00
8	0	0	0	0	0	0	0	4	0	0	0	0	0	0	0	0	0	0	4	68	0	0	100.00	1.00	1.00	1.00	1.00
9	0	0	0	0	0	0	0	0	4	0	0	0	0	0	0	0	0	0	4	68	0	0	100.00	1.00	1.00	1.00	1.00
10	0	0	0	0	0	0	0	0	0	4	0	0	0	0	0	0	0	0	4	68	0	0	100.00	1.00	1.00	1.00	1.00
11	0	0	0	0	0	0	0	0	0	0	4	0	0	0	0	0	0	0	4	68	0	0	100.00	1.00	1.00	1.00	1.00
12	0	0	0	0	0	0	0	0	0	0	0	4	0	0	0	0	0	0	4	68	0	0	100.00	1.00	1.00	1.00	1.00
13	0	0	0	0	0	0	0	0	0	0	0	0	4	0	0	0	0	0	4	68	0	0	100.00	1.00	1.00	1.00	1.00
14	0	0	0	0	0	0	0	0	0	0	0	0	0	4	0	0	0	0	4	68	0	0	100.00	1.00	1.00	1.00	1.00
15	0	0	0	0	0	0	0	0	0	0	0	0	0	0	4	0	0	0	4	68	0	0	100.00	1.00	1.00	1.00	1.00
16	0	0	0	0	0	0	0	0	0	0	0	0	0	0	0	4	0	0	4	68	0	0	100.00	1.00	1.00	1.00	1.00
17	0	0	0	0	0	0	0	0	0	0	0	0	0	0	0	0	4	0	4	68	0	0	100.00	1.00	1.00	1.00	1.00
18	0	0	0	0	0	0	0	0	0	0	0	0	0	0	0	0	0	4	4	68	0	0	100.00	1.00	1.00	1.00	1.00

Table S45. Confusion matrixes of the calibration set and validation set of Low-KNN

Calibration set																												
**Class**	**1**	**2**	**3**	**4**	**5**	**6**	**7**	**8**	**9**	**10**	**11**	**12**	**13**	**14**	**15**	**16**	**17**	**18**	**TP**	**TN**	**FP**	**FN**	**ACC**	**SE**	**SP**	**MCC**	**EFF**
1	6	0	0	0	0	0	0	0	0	0	0	0	0	0	0	0	0	0	6	102	0	0	100.00	1.00	1.00	1.00	1.00
2	0	6	0	0	0	0	0	0	0	0	0	0	0	0	0	0	0	0	6	102	0	0	100.00	1.00	1.00	1.00	1.00
3	0	0	6	0	0	0	0	0	0	0	0	0	0	0	0	0	0	0	6	102	0	0	100.00	1.00	1.00	1.00	1.00
4	0	0	0	6	0	0	0	0	0	0	0	0	0	0	0	0	0	0	6	102	0	0	100.00	1.00	1.00	1.00	1.00
5	0	0	0	0	6	0	0	0	0	0	0	0	0	0	0	0	0	0	6	102	0	0	100.00	1.00	1.00	1.00	1.00
6	0	0	0	0	0	6	0	0	0	0	0	0	0	0	0	0	0	0	6	102	0	0	100.00	1.00	1.00	1.00	1.00
7	0	0	0	0	0	0	6	0	0	0	0	0	0	0	0	0	0	0	6	102	0	0	100.00	1.00	1.00	1.00	1.00
8	0	0	0	0	0	0	0	6	0	0	0	0	0	0	0	0	0	0	6	102	0	0	100.00	1.00	1.00	1.00	1.00
9	0	0	0	0	0	0	0	0	6	0	0	0	0	0	0	0	0	0	6	102	0	0	100.00	1.00	1.00	1.00	1.00
10	0	0	0	0	0	0	0	0	0	6	0	0	0	0	0	0	0	0	6	102	0	0	100.00	1.00	1.00	1.00	1.00
11	0	0	0	0	0	0	0	0	0	0	6	0	0	0	0	0	0	0	6	102	0	0	100.00	1.00	1.00	1.00	1.00
12	0	0	0	0	0	0	0	0	0	0	0	6	0	0	0	0	0	0	6	102	0	0	100.00	1.00	1.00	1.00	1.00
13	0	0	0	0	0	0	0	0	0	0	0	0	6	0	0	0	0	0	6	102	0	0	100.00	1.00	1.00	1.00	1.00
14	0	0	0	0	0	0	0	0	0	0	0	0	0	6	0	0	0	0	6	102	0	0	100.00	1.00	1.00	1.00	1.00
15	0	0	0	0	0	0	0	0	0	0	0	0	0	0	6	0	0	0	6	102	0	0	100.00	1.00	1.00	1.00	1.00
16	0	0	0	0	0	0	0	0	0	0	0	0	0	0	0	6	0	0	6	102	0	0	100.00	1.00	1.00	1.00	1.00
17	0	0	0	0	0	0	0	0	0	0	0	0	0	0	0	0	6	0	6	102	0	0	100.00	1.00	1.00	1.00	1.00
18	0	0	0	0	0	0	0	0	0	0	0	0	0	0	0	0	0	6	6	102	0	0	100.00	1.00	1.00	1.00	1.00

Validation set																											
**Class**	**1**	**2**	**3**	**4**	**5**	**6**	**7**	**8**	**9**	**10**	**11**	**12**	**13**	**14**	**15**	**16**	**17**	**18**	**TP**	**TN**	**FP**	**FN**	**ACC**	**SE**	**SP**	**MCC**	**EFF**
1	4	0	0	0	0	0	0	0	0	0	0	0	0	0	0	0	0	0	4	68	0	0	100.00	1.00	1.00	1.00	1.00
2	0	4	0	0	0	0	0	0	0	0	0	0	0	0	0	0	0	0	4	67	1	0	98.61	1.00	0.99	0.89	0.99
3	0	0	4	0	0	0	0	0	0	0	0	0	0	0	0	0	0	0	4	68	0	0	100.00	1.00	1.00	1.00	1.00
4	0	0	0	4	0	0	0	0	0	0	0	0	0	0	0	0	0	0	4	68	0	0	100.00	1.00	1.00	1.00	1.00
5	0	0	0	0	4	0	0	0	0	0	0	0	0	0	0	0	0	0	4	68	0	0	100.00	1.00	1.00	1.00	1.00
6	0	0	0	0	0	4	0	0	0	0	0	0	0	0	0	0	0	0	4	68	0	0	100.00	1.00	1.00	1.00	1.00
7	0	0	0	0	0	0	4	0	0	0	0	0	0	0	0	0	0	0	4	68	0	0	100.00	1.00	1.00	1.00	1.00
8	0	0	0	0	0	0	0	4	0	0	0	0	0	0	0	0	0	0	4	68	0	0	100.00	1.00	1.00	1.00	1.00
9	0	0	0	0	0	0	0	0	4	0	0	0	0	0	0	0	0	0	4	68	0	0	100.00	1.00	1.00	1.00	1.00
10	0	1	0	0	0	0	0	0	0	3	0	0	0	0	0	0	0	0	3	67	1	1	97.22	0.75	0.99	0.74	0.86
11	0	0	0	0	0	0	0	0	0	0	4	0	0	0	0	0	0	0	4	68	0	0	100.00	1.00	1.00	1.00	1.00
12	0	0	0	0	0	0	0	0	0	0	0	4	0	0	0	0	0	0	4	68	0	0	100.00	1.00	1.00	1.00	1.00
13	0	0	0	0	0	0	0	0	0	0	0	0	4	0	0	0	0	0	4	68	0	0	100.00	1.00	1.00	1.00	1.00
14	0	0	0	0	0	0	0	0	0	0	0	0	0	4	0	0	0	0	4	68	0	0	100.00	1.00	1.00	1.00	1.00
15	0	0	0	0	0	0	0	0	0	0	0	0	0	0	4	0	0	0	4	68	0	0	100.00	1.00	1.00	1.00	1.00
16	0	0	0	0	0	0	0	0	0	0	0	0	0	0	0	4	0	0	4	68	0	0	100.00	1.00	1.00	1.00	1.00
17	0	0	0	0	0	0	0	0	0	0	0	0	0	0	0	0	4	0	4	68	0	0	100.00	1.00	1.00	1.00	1.00
18	0	0	0	0	0	0	0	0	0	1	0	0	0	0	0	0	0	3	3	68	0	1	98.61	0.75	1.00	0.86	0.87

Table S46. Confusion matrixes of the calibration set and validation set of Mid-RF

Calibration set																											
**Class**	**1**	**2**	**3**	**4**	**5**	**6**	**7**	**8**	**9**	**10**	**11**	**12**	**13**	**14**	**15**	**16**	**17**	**18**	**TP**	**TN**	**FP**	**FN**	**ACC**	**SE**	**SP**	**MCC**	**EFF**
1	6	0	0	0	0	0	0	0	0	0	0	0	0	0	0	0	0	0	6	102	0	0	100.00	1.00	1.00	1.00	1.00
2	0	6	0	0	0	0	0	0	0	0	0	0	0	0	0	0	0	0	6	102	0	0	100.00	1.00	1.00	1.00	1.00
3	0	0	6	0	0	0	0	0	0	0	0	0	0	0	0	0	0	0	6	102	0	0	100.00	1.00	1.00	1.00	1.00
4	0	0	0	6	0	0	0	0	0	0	0	0	0	0	0	0	0	0	6	102	0	0	100.00	1.00	1.00	1.00	1.00
5	0	0	0	0	6	0	0	0	0	0	0	0	0	0	0	0	0	0	6	102	0	0	100.00	1.00	1.00	1.00	1.00
6	0	0	0	0	0	6	0	0	0	0	0	0	0	0	0	0	0	0	6	102	0	0	100.00	1.00	1.00	1.00	1.00
7	0	0	0	0	0	0	6	0	0	0	0	0	0	0	0	0	0	0	6	102	0	0	100.00	1.00	1.00	1.00	1.00
8	0	0	0	0	0	0	0	6	0	0	0	0	0	0	0	0	0	0	6	102	0	0	100.00	1.00	1.00	1.00	1.00
9	0	0	0	0	0	0	0	0	6	0	0	0	0	0	0	0	0	0	6	102	0	0	100.00	1.00	1.00	1.00	1.00
10	0	0	0	0	0	0	0	0	0	6	0	0	0	0	0	0	0	0	6	102	0	0	100.00	1.00	1.00	1.00	1.00
11	0	0	0	0	0	0	0	0	0	0	6	0	0	0	0	0	0	0	6	102	0	0	100.00	1.00	1.00	1.00	1.00
12	0	0	0	0	0	0	0	0	0	0	0	6	0	0	0	0	0	0	6	102	0	0	100.00	1.00	1.00	1.00	1.00
13	0	0	0	0	0	0	0	0	0	0	0	0	6	0	0	0	0	0	6	102	0	0	100.00	1.00	1.00	1.00	1.00
14	0	0	0	0	0	0	0	0	0	0	0	0	0	6	0	0	0	0	6	102	0	0	100.00	1.00	1.00	1.00	1.00
15	0	0	0	0	0	0	0	0	0	0	0	0	0	0	6	0	0	0	6	102	0	0	100.00	1.00	1.00	1.00	1.00
16	0	0	0	0	0	0	0	0	0	0	0	0	0	0	0	6	0	0	6	102	0	0	100.00	1.00	1.00	1.00	1.00
17	0	0	0	0	0	0	0	0	0	0	0	0	0	0	0	0	6	0	6	102	0	0	100.00	1.00	1.00	1.00	1.00
18	0	0	0	0	0	0	0	0	0	0	0	0	0	0	0	0	0	6	6	102	0	0	100.00	1.00	1.00	1.00	1.00

Validation set																											
**Class**	**1**	**2**	**3**	**4**	**5**	**6**	**7**	**8**	**9**	**10**	**11**	**12**	**13**	**14**	**15**	**16**	**17**	**18**	**TP**	**TN**	**FP**	**FN**	**ACC**	**SE**	**SP**	**MCC**	**EFF**
1	4	0	0	0	0	0	0	0	0	0	0	0	0	0	0	0	0	0	4	68	0	0	100.00	1.00	1.00	1.00	1.00
2	0	4	0	0	0	0	0	0	0	0	0	0	0	0	0	0	0	0	4	68	0	0	100.00	1.00	1.00	1.00	1.00
3	0	0	4	0	0	0	0	0	0	0	0	0	0	0	0	0	0	0	4	68	0	0	100.00	1.00	1.00	1.00	1.00
4	0	0	0	4	0	0	0	0	0	0	0	0	0	0	0	0	0	0	4	68	0	0	100.00	1.00	1.00	1.00	1.00
5	0	0	0	0	4	0	0	0	0	0	0	0	0	0	0	0	0	0	4	68	0	0	100.00	1.00	1.00	1.00	1.00
6	0	0	0	0	0	4	0	0	0	0	0	0	0	0	0	0	0	0	4	68	0	0	100.00	1.00	1.00	1.00	1.00
7	0	0	0	0	0	0	4	0	0	0	0	0	0	0	0	0	0	0	4	68	0	0	100.00	1.00	1.00	1.00	1.00
8	0	0	0	0	0	0	0	4	0	0	0	0	0	0	0	0	0	0	4	68	0	0	100.00	1.00	1.00	1.00	1.00
9	0	0	0	0	0	0	0	0	4	0	0	0	0	0	0	0	0	0	4	68	0	0	100.00	1.00	1.00	1.00	1.00
10	0	0	0	0	0	0	0	0	0	4	0	0	0	0	0	0	0	0	4	68	0	0	100.00	1.00	1.00	1.00	1.00
11	0	0	0	0	0	0	0	0	0	0	4	0	0	0	0	0	0	0	4	68	0	0	100.00	1.00	1.00	1.00	1.00
12	0	0	0	0	0	0	0	0	0	0	0	4	0	0	0	0	0	0	4	68	0	0	100.00	1.00	1.00	1.00	1.00
13	0	0	0	0	0	0	0	0	0	0	0	0	4	0	0	0	0	0	4	68	0	0	100.00	1.00	1.00	1.00	1.00
14	0	0	0	0	0	0	0	0	0	0	0	0	0	4	0	0	0	0	4	68	0	0	100.00	1.00	1.00	1.00	1.00
15	0	0	0	0	0	0	0	0	0	0	0	0	0	0	4	0	0	0	4	68	0	0	100.00	1.00	1.00	1.00	1.00
16	0	0	0	0	0	0	0	0	0	0	0	0	0	0	0	4	0	0	4	68	0	0	100.00	1.00	1.00	1.00	1.00
17	0	0	0	0	0	0	0	0	0	0	0	0	0	0	0	0	4	0	4	68	0	0	100.00	1.00	1.00	1.00	1.00
18	0	0	0	0	0	0	0	0	0	0	0	0	0	0	0	0	0	4	4	68	0	0	100.00	1.00	1.00	1.00	1.00

Table S47. Confusion matrixes of the calibration set and validation set of Mid-SVM

Calibration set																												
**Class**	**1**	**2**	**3**	**4**	**5**	**6**	**7**	**8**	**9**	**10**	**11**	**12**	**13**	**14**	**15**	**16**	**17**	**18**	**TP**	**TN**	**FP**	**FN**	**ACC**	**SE**	**SP**	**MCC**	**EFF**
1	6	0	0	0	0	0	0	0	0	0	0	0	0	0	0	0	0	0	6	102	0	0	100.00	1.00	1.00	1.00	1.00
2	0	6	0	0	0	0	0	0	0	0	0	0	0	0	0	0	0	0	6	102	0	0	100.00	1.00	1.00	1.00	1.00
3	0	0	6	0	0	0	0	0	0	0	0	0	0	0	0	0	0	0	6	102	0	0	100.00	1.00	1.00	1.00	1.00
4	0	0	0	6	0	0	0	0	0	0	0	0	0	0	0	0	0	0	6	102	0	0	100.00	1.00	1.00	1.00	1.00
5	0	0	0	0	6	0	0	0	0	0	0	0	0	0	0	0	0	0	6	102	0	0	100.00	1.00	1.00	1.00	1.00
6	0	0	0	0	0	6	0	0	0	0	0	0	0	0	0	0	0	0	6	102	0	0	100.00	1.00	1.00	1.00	1.00
7	0	0	0	0	0	0	6	0	0	0	0	0	0	0	0	0	0	0	6	102	0	0	100.00	1.00	1.00	1.00	1.00
8	0	0	0	0	0	0	0	6	0	0	0	0	0	0	0	0	0	0	6	102	0	0	100.00	1.00	1.00	1.00	1.00
9	0	0	0	0	0	0	0	0	6	0	0	0	0	0	0	0	0	0	6	102	0	0	100.00	1.00	1.00	1.00	1.00
10	0	0	0	0	0	0	0	0	0	6	0	0	0	0	0	0	0	0	6	102	0	0	100.00	1.00	1.00	1.00	1.00
11	0	0	0	0	0	0	0	0	0	0	6	0	0	0	0	0	0	0	6	102	0	0	100.00	1.00	1.00	1.00	1.00
12	0	0	0	0	0	0	0	0	0	0	0	6	0	0	0	0	0	0	6	102	0	0	100.00	1.00	1.00	1.00	1.00
13	0	0	0	0	0	0	0	0	0	0	0	0	6	0	0	0	0	0	6	102	0	0	100.00	1.00	1.00	1.00	1.00
14	0	0	0	0	0	0	0	0	0	0	0	0	0	6	0	0	0	0	6	102	0	0	100.00	1.00	1.00	1.00	1.00
15	0	0	0	0	0	0	0	0	0	0	0	0	0	0	6	0	0	0	6	102	0	0	100.00	1.00	1.00	1.00	1.00
16	0	0	0	0	0	0	0	0	0	0	0	0	0	0	0	6	0	0	6	102	0	0	100.00	1.00	1.00	1.00	1.00
17	0	0	0	0	0	0	0	0	0	0	0	0	0	0	0	0	6	0	6	102	0	0	100.00	1.00	1.00	1.00	1.00
18	0	0	0	0	0	0	0	0	0	0	0	0	0	0	0	0	0	6	6	102	0	0	100.00	1.00	1.00	1.00	1.00

	Validation set																										
**Class**	**1**	**2**	**3**	**4**	**5**	**6**	**7**	**8**	**9**	**10**	**11**	**12**	**13**	**14**	**15**	**16**	**17**	**18**	**TP**	**TN**	**FP**	**FN**	**ACC**	**SE**	**SP**	**MCC**	**EFF**
1	4	0	0	0	0	0	0	0	0	0	0	0	0	0	0	0	0	0	4	68	0	0	100.00	1.00	1.00	1.00	1.00
2	0	4	0	0	0	0	0	0	0	0	0	0	0	0	0	0	0	0	4	68	0	0	100.00	1.00	1.00	1.00	1.00
3	0	0	4	0	0	0	0	0	0	0	0	0	0	0	0	0	0	0	4	68	0	0	100.00	1.00	1.00	1.00	1.00
4	0	0	0	4	0	0	0	0	0	0	0	0	0	0	0	0	0	0	4	68	0	0	100.00	1.00	1.00	1.00	1.00
5	0	0	0	0	4	0	0	0	0	0	0	0	0	0	0	0	0	0	4	68	0	0	100.00	1.00	1.00	1.00	1.00
6	0	0	0	0	0	4	0	0	0	0	0	0	0	0	0	0	0	0	4	68	0	0	100.00	1.00	1.00	1.00	1.00
7	0	0	0	0	0	0	4	0	0	0	0	0	0	0	0	0	0	0	4	68	0	0	100.00	1.00	1.00	1.00	1.00
8	0	0	0	0	0	0	0	4	0	0	0	0	0	0	0	0	0	0	4	68	0	0	100.00	1.00	1.00	1.00	1.00
9	0	0	0	0	0	0	0	0	4	0	0	0	0	0	0	0	0	0	4	68	0	0	100.00	1.00	1.00	1.00	1.00
10	0	0	0	0	0	0	0	0	0	4	0	0	0	0	0	0	0	0	4	68	0	0	100.00	1.00	1.00	1.00	1.00
11	0	0	0	0	0	0	0	0	0	0	4	0	0	0	0	0	0	0	4	68	0	0	100.00	1.00	1.00	1.00	1.00
12	0	0	0	0	0	0	0	0	0	0	0	4	0	0	0	0	0	0	4	68	0	0	100.00	1.00	1.00	1.00	1.00
13	0	0	0	0	0	0	0	0	0	0	0	0	4	0	0	0	0	0	4	68	0	0	100.00	1.00	1.00	1.00	1.00
14	0	0	0	0	0	0	0	0	0	0	0	0	0	4	0	0	0	0	4	68	0	0	100.00	1.00	1.00	1.00	1.00
15	0	0	0	0	0	0	0	0	0	0	0	0	0	0	4	0	0	0	4	68	0	0	100.00	1.00	1.00	1.00	1.00
16	0	0	0	0	0	0	0	0	0	0	0	0	0	0	0	4	0	0	4	68	0	0	100.00	1.00	1.00	1.00	1.00
17	0	0	0	0	0	0	0	0	0	0	0	0	0	0	0	0	4	0	4	68	0	0	100.00	1.00	1.00	1.00	1.00
18	0	0	0	0	0	0	0	0	0	0	0	0	0	0	0	0	0	4	4	68	0	0	100.00	1.00	1.00	1.00	1.00

Table S48. Confusion matrixes of the calibration set and validation set of Mid-KNN

Calibration set																											
**Class**	**1**	**2**	**3**	**4**	**5**	**6**	**7**	**8**	**9**	**10**	**11**	**12**	**13**	**14**	**15**	**16**	**17**	**18**	**TP**	**TN**	**FP**	**FN**	**ACC**	**SE**	**SP**	**MCC**	**EFF**
1	6	0	0	0	0	0	0	0	0	0	0	0	0	0	0	0	0	0	6	102	0	0	100.00	1.00	1.00	1.00	1.00
2	0	6	0	0	0	0	0	0	0	0	0	0	0	0	0	0	0	0	6	102	0	0	100.00	1.00	1.00	1.00	1.00
3	0	0	6	0	0	0	0	0	0	0	0	0	0	0	0	0	0	0	6	102	0	0	100.00	1.00	1.00	1.00	1.00
4	0	0	0	6	0	0	0	0	0	0	0	0	0	0	0	0	0	0	6	102	0	0	100.00	1.00	1.00	1.00	1.00
5	0	0	0	0	6	0	0	0	0	0	0	0	0	0	0	0	0	0	6	102	0	0	100.00	1.00	1.00	1.00	1.00
6	0	0	0	0	0	6	0	0	0	0	0	0	0	0	0	0	0	0	6	102	0	0	100.00	1.00	1.00	1.00	1.00
7	0	0	0	0	0	0	6	0	0	0	0	0	0	0	0	0	0	0	6	102	0	0	100.00	1.00	1.00	1.00	1.00
8	0	0	0	0	0	0	0	6	0	0	0	0	0	0	0	0	0	0	6	102	0	0	100.00	1.00	1.00	1.00	1.00
9	0	0	0	0	0	0	0	0	6	0	0	0	0	0	0	0	0	0	6	102	0	0	100.00	1.00	1.00	1.00	1.00
10	0	0	0	0	0	0	0	0	0	6	0	0	0	0	0	0	0	0	6	102	0	0	100.00	1.00	1.00	1.00	1.00
11	0	0	0	0	0	0	0	0	0	0	6	0	0	0	0	0	0	0	6	102	0	0	100.00	1.00	1.00	1.00	1.00
12	0	0	0	0	0	0	0	0	0	0	0	6	0	0	0	0	0	0	6	102	0	0	100.00	1.00	1.00	1.00	1.00
13	0	0	0	0	0	0	0	0	0	0	0	0	6	0	0	0	0	0	6	102	0	0	100.00	1.00	1.00	1.00	1.00
14	0	0	0	0	0	0	0	0	0	0	0	0	0	6	0	0	0	0	6	102	0	0	100.00	1.00	1.00	1.00	1.00
15	0	0	0	0	0	0	0	0	0	0	0	0	0	0	6	0	0	0	6	102	0	0	100.00	1.00	1.00	1.00	1.00
16	0	0	0	0	0	0	0	0	0	0	0	0	0	0	0	6	0	0	6	102	0	0	100.00	1.00	1.00	1.00	1.00
17	0	0	0	0	0	0	0	0	0	0	0	0	0	0	0	0	6	0	6	102	0	0	100.00	1.00	1.00	1.00	1.00
18	0	0	0	0	0	0	0	0	0	0	0	0	0	0	0	0	0	6	6	102	0	0	100.00	1.00	1.00	1.00	1.00

Validation set																											
**Class**	**1**	**2**	**3**	**4**	**5**	**6**	**7**	**8**	**9**	**10**	**11**	**12**	**13**	**14**	**15**	**16**	**17**	**18**	**TP**	**TN**	**FP**	**FN**	**ACC**	**SE**	**SP**	**MCC**	**EFF**
1	4	0	0	0	0	0	0	0	0	0	0	0	0	0	0	0	0	0	4	68	0	0	100.00	1.00	1.00	1.00	1.00
2	0	4	0	0	0	0	0	0	0	0	0	0	0	0	0	0	0	0	4	68	0	0	100.00	1.00	1.00	1.00	1.00
3	0	0	4	0	0	0	0	0	0	0	0	0	0	0	0	0	0	0	4	68	0	0	100.00	1.00	1.00	1.00	1.00
4	0	0	0	4	0	0	0	0	0	0	0	0	0	0	0	0	0	0	4	68	0	0	100.00	1.00	1.00	1.00	1.00
5	0	0	0	0	4	0	0	0	0	0	0	0	0	0	0	0	0	0	4	68	0	0	100.00	1.00	1.00	1.00	1.00
6	0	0	0	0	0	4	0	0	0	0	0	0	0	0	0	0	0	0	4	68	0	0	100.00	1.00	1.00	1.00	1.00
7	0	0	0	0	0	0	4	0	0	0	0	0	0	0	0	0	0	0	4	68	0	0	100.00	1.00	1.00	1.00	1.00
8	0	0	0	0	0	0	0	4	0	0	0	0	0	0	0	0	0	0	4	68	0	0	100.00	1.00	1.00	1.00	1.00
9	0	0	0	0	0	0	0	0	4	0	0	0	0	0	0	0	0	0	4	68	0	0	100.00	1.00	1.00	1.00	1.00
10	0	0	0	0	0	0	0	0	0	4	0	0	0	0	0	0	0	0	4	68	0	0	100.00	1.00	1.00	1.00	1.00
11	0	0	0	0	0	0	0	0	0	0	4	0	0	0	0	0	0	0	4	68	0	0	100.00	1.00	1.00	1.00	1.00
12	0	0	0	0	0	0	0	0	0	0	0	4	0	0	0	0	0	0	4	68	0	0	100.00	1.00	1.00	1.00	1.00
13	0	0	0	0	0	0	0	0	0	0	0	0	4	0	0	0	0	0	4	68	0	0	100.00	1.00	1.00	1.00	1.00
14	0	0	0	0	0	0	0	0	0	0	0	0	0	4	0	0	0	0	4	68	0	0	100.00	1.00	1.00	1.00	1.00
15	0	0	0	0	0	0	0	0	0	0	0	0	0	0	4	0	0	0	4	68	0	0	100.00	1.00	1.00	1.00	1.00
16	0	0	0	0	0	0	0	0	0	0	0	0	0	0	0	4	0	0	4	68	0	0	100.00	1.00	1.00	1.00	1.00
17	0	0	0	0	0	0	0	0	0	0	0	0	0	0	0	0	4	0	4	68	0	0	100.00	1.00	1.00	1.00	1.00
18	0	0	0	0	0	0	0	0	0	0	0	0	0	0	0	0	0	4	4	68	0	0	100.00	1.00	1.00	1.00	1.00
